# Predicting genome-wide functional constraints with GPN-Star

**DOI:** 10.1038/s41586-026-11005-5

**Published:** 2026-09-09

**Authors:** Chengzhong Ye, Gonzalo Benegas, Carlos Albors, Jianan Canal Li, Sebastian Prillo, Peter D. Fields, Brian Clarke, Yun S. Song

**Affiliations:** 1 Department of Statistics, University of California, Berkeley, CA USA; 2 Computer Science Division, University of California, Berkeley, CA USA; 3 The Jackson Laboratory, Bar Harbor, ME USA; 4 Division of Computational Genomics and Systems Genetics, German Cancer Research Center (DKFZ), Heidelberg, Germany; 5 Center for Computational Biology, University of California, Berkeley, CA USA; 6 Innovative Genomics Institute, University of California, Berkeley, CA USA

**Keywords:** Machine learning, Evolutionary biology, Genetic variation, Phylogenomics

## Abstract

Genomic language models have emerged as a powerful approach for learning genome-wide functional constraints directly from DNA sequences^1^. However, standard genomic language models adapted from natural language processing often require large model sizes and computational resources, yet still fall short of classical evolutionary models in predictive tasks^2–4^. Here we introduce a genomic pretrained network with species tree and alignment representations (GPN-Star), which is a biologically grounded genomic language model featuring a phylogeny-aware architecture that leverages whole-genome alignments and species trees to model evolutionary relationships explicitly. Trained on alignments spanning vertebrate, mammal and primate evolutionary timescales, GPN-Star achieves state-of-the-art performance across a wide range of variant effect prediction tasks in both coding and non-coding regions of the human genome. Analyses across timescales show task-dependent advantages of modelling more recent versus deeper evolution. To demonstrate its potential to advance human genetics, we show that GPN-Star substantially outperforms previous methods in prioritizing pathogenic and fine-mapped genome-wide association study variants, yields strong enrichments of complex trait heritability and improves power in rare variant association testing^5^. Extending beyond humans, we train GPN-Star for five model organisms—*Mus musculus*, *Gallus gallus*, *Drosophila melanogaster*, *Caenorhabditis elegans* and *Arabidopsis thaliana*—demonstrating the robustness and generalizability of the framework. Taken together, these results position GPN-Star as a scalable, powerful and flexible tool for genome interpretation, well suited to leverage the growing abundance of comparative genomics data.

## Abstract (web summary)

GPN-Star, a genomic language model with a phylogeny-aware architecture for whole-genome alignment data, is shown to be a scalable and flexible tool for genetic variant effect prediction across species.

## Main

A fundamental challenge in biology is understanding the functional significance of genetic variants. Despite tremendous advances in experimental technologies in genomics, determining which variants influence phenotypes or contribute to diseases remains difficult. Evolution provides valuable insights: deleterious mutations tend to be purged by natural selection, although tolerated or advantageous changes may accumulate. Millions of years of evolution therefore provide a genome-wide record of functional constraint for variant interpretation. This idea has deep roots in molecular evolution and comparative sequence analysis^6^. More recently, large comparative genomics studies have quantified evolutionary constraints across the genomes of hundreds of species^7,8^.

Genomic language models (gLMs) have emerged as a promising approach for extracting evolutionary information directly from raw DNA sequences through self-supervised learning (ref. ^1^ and references therein). By predicting masked nucleotides from sequence context, gLMs estimate per-site likelihoods that reflect evolutionary constraint without requiring labelled data. These likelihoods have been shown to be effective predictors of genome-wide variant effects^1,9^. However, gLMs based on standard language modelling frameworks still underperform compared with much simpler classical phylogenetic models on certain variant interpretation tasks—particularly in complex eukaryotic genomes such as in humans and in distal regulatory elements such as enhancers^2^—even with massive model sizes^3,4^.

A long-standing approach to modelling evolutionary data is to construct multiple sequence alignments (MSAs). By algorithmically aligning homologous loci across biological sequences, MSAs show site-specific patterns of conservation and facilitate the inference of evolutionary preferences for variants at specific positions. Protein MSAs have enabled highly successful models, including AlphaFold^10^, MSA Transformer^11^ and EVE^12^, and have recently experienced renewed interest as scaling single-sequence protein language models has shown diminishing returns^13–15^. Extending this concept from proteins to genomes, whole-genome alignments (WGAs) of complete assemblies from tens to hundreds of species enable genome-wide studies of sequence evolution^16,17^. Classical parametric phylogenetic models fitted on WGAs—such as PhastCons^18^ and PhyloP^19^—have long been staples in variant interpretation for humans and other species. Recent large-scale consortia have further expanded high-quality WGA resources^20,21^.

Therefore, combining the gLM framework with WGA data presents a promising direction, and we recently demonstrated this potential with GPN-MSA^22^ trained on a WGA of 90 vertebrate species. Here we present GPN-Star (genomic pretrained network with species tree and alignment representations), a general gLM framework that more effectively exploits multispecies WGAs through a new model architecture with phylogeny-aware designs; see Supplementary Information for key differences between GPN-MSA and GPN-Star. This flexible framework enabled us to apply GPN-Star to three WGAs relative to the human genome, spanning vertebrate, mammal and primate evolutionary timescales. GPN-Star achieves state-of-the-art performance across coding and non-coding variant interpretation tasks. The vertebrate model consistently outperforms GPN-MSA trained on the same alignment, whereas the mammal and primate models excel particularly at predicting non-coding variant effects. We further evaluate its use in human genetics by analysing the heritability of complex traits. Importantly, GPN-Star trained on the primate WGA prioritizes variants with an unprecedented amount of heritability enrichment across more than a hundred complex traits^23^. Also, we find a striking connection between the most informative evolutionary timescale for constraint prediction and the effective polygenicity of traits.

To demonstrate the generality of our approach, we apply GPN-Star to five model organisms with minimal tuning and show its effectiveness in assessing variant effects in these species.

## Alignment- and phylogeny-informed gLMs

**Fig. 1:**
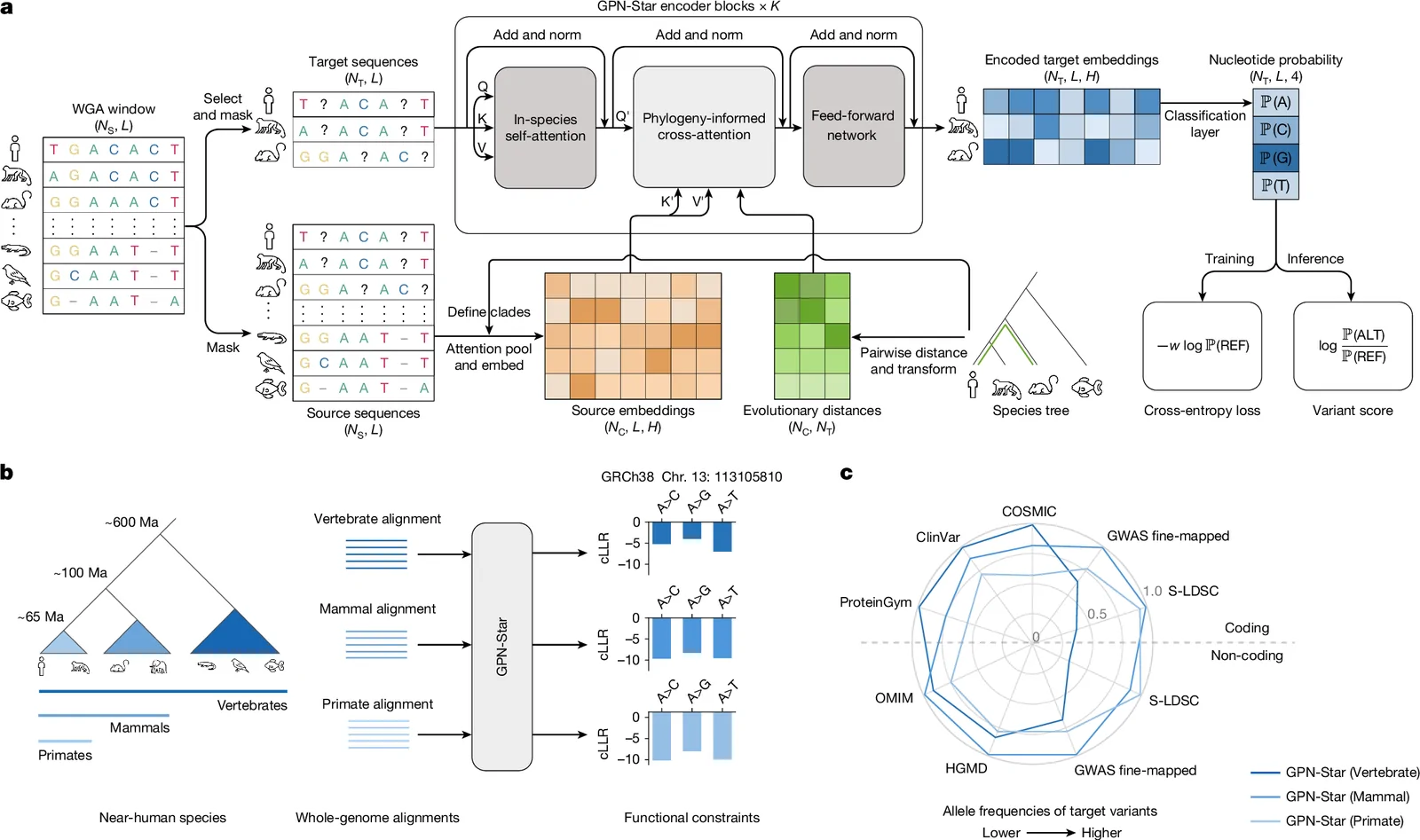
Overview of GPN-Star. **a**, A diagram of the GPN-Star model architecture. The input to the model is an arbitrary multispecies WGA window. The target sequences and source sequences are constructed from the alignment window. The source sequences are compressed into clade-level embeddings using attention pooling following the species tree. The target sequences are encoded through a stack of GPN-Star encoder blocks, where the phylogeny-informed cross-attention module integrates information from the source sequences guided by evolutionary distances between species based on the species tree. Finally, a classification layer transforms the encoded embeddings to nucleotide probabilities at each locus in the target sequences, which are then used to compute the training loss or to make predictions on variant impact. *H*, hidden dimension; *K*, number of encoder blocks; *L*, length of the alignment block; *N*_C_, number of clades; *N*_S_, number of species; *N*_T_, number of target species; *V*, size of the nucleotide vocabulary; *w*, loss weight; *ℙ*, nucleotide probability. Q, K and V (and Q', K' and V') in the encoder blocks refer to the components of an attention module (query, key and value). A full description of the model is provided in Methods. **b**, Application of GPN-Star to the human genome. Three models were trained on vertebrate, mammal and primate alignments, respectively, learning functional constraints at different evolutionary timescales (Methods). **c**, A summary of performance across the downstream tasks of the vertebrate, mammal and primate GPN-Star models. For ClinVar, COSMIC, OMIM, HGMD and GWAS fine-mapped datasets, the performance metric was AUPRC. For ProteinGym, the metric was the mean Spearman’s *ρ* across assays. For S-LDSC, the metric was heritability enrichment. The performance metrics were scaled linearly to the range of 0 to 1; in each task 0 was defined as the lowest metric among the three GPN-Star models and PhyloP and PhastCons fitted at the same three evolutionary timescales, and 1 was defined as the highest metric. cLLR, calibrated log-likelihood ratio; Ma, million years ago. Animal icons in **a**,**b** created by Berkahicon are from Flaticon (www.flaticon.com).

GPN-Star learns functional constraints by leveraging evolutionary signals embedded in multispecies phylogeny and WGAs. Inspired by classical evolutionary models, it characterizes how genomic positions evolve across species. To achieve this, we introduce a specialized transformer architecture that integrates sequence context and evolutionary information (Fig. 1a).

GPN-Star uses an encoder-only architecture trained with a masked language modelling (MLM) objective. Each input consists of a WGA window and its species tree. The alignment is partitioned into target and source sequences and the model predicts masked nucleotides in the targets conditioned on both their sequence context and the evolutionary information in the sources. This is accomplished through a stack of encoder blocks, each comprising a sequence-wise self-attention module to encode intrasequence context, a phylogeny-informed cross-attention module to encode evolutionary context from the source sequences and a feed-forward network to integrate the information. To efficiently represent phylogenetic structure, we group closely related source species according to their evolutionary distances and pool their sequences into clade-level representations. The cross-attention module adaptively weights source sequence contributions using evolutionary distances derived from the species tree using an attention mechanism. The resulting embeddings for the target sequences are then passed to an MLM head for nucleotide probability prediction. Full architectural details are provided in Methods.

Another methodological innovation of this work is that it accounts for mutation rate variation in gLMs. Because GPN-Star is trained on genomic sequences observed in nature, its predictions reflect both mutation and selection. To disentangle these signals and isolate selective constraints, we normalized the prediction by the model of each variant against its background mutation effect estimated from high-confidence neutral sites in the genome (Methods). Unless otherwise specified, all analyses in this study use these calibrated scores to capture functional constraints with minimal mutation rate biases.

The GPN-Star framework is general and flexible, designed to work with any alignment data from any species, requiring minimal hyperparameter tuning to achieve robust performance (Methods). We first applied it to the human genome, training three separate GPN-Star models using the largest available vertebrate, mammalian and primate WGAs (Fig. 1b; Methods). Throughout this study, we denote these models as GPN-Star (V), GPN-Star (M) and GPN-Star (P), respectively. We have experimented with different model sizes, but we focus here on the largest models (200 million parameters), trained for several days on 8 NVIDIA A100 GPUs (Supplementary Table 1). Existing single-sequence gLMs such as Nucleotide Transformer (trained on 128 A100 GPUs for a month)^3^ and Evo 2 (trained on over 2,000 H100 GPUs for several months)^4^ require substantial computational resources to implicitly learn evolutionary constraints, in exchange for flexibility and broader applications. We show that GPN-Star achieves superior constraint prediction with a much smaller resource footprint by leveraging the explicit evolutionary context provided by alignment data.

Unlike previous gLMs trained across extremely broad evolutionary distances (for example, spanning prokaryotes to humans), GPN-Star focuses on narrower, more recent phylogenetic distances closely related to humans (Fig. 1b). As we demonstrate below, modelling longer evolutionary histories is not always helpful. Instead, capturing recent evolutionary constraints proves especially advantageous for interpreting certain classes of genetic variants (Fig. 1c).

## Pathogenic coding variants

A critical challenge in human genetics is understanding the impact of genetic variants on disease susceptibility—an essential step towards improving the diagnosis of genetic disorders, identifying drug targets and realizing the promise of precision medicine. Many computational models have been developed to predict variant pathogenicity in the human genome. Here we systematically evaluate the performance of GPN-Star across a comprehensive set of benchmarks.

**Fig. 2:**
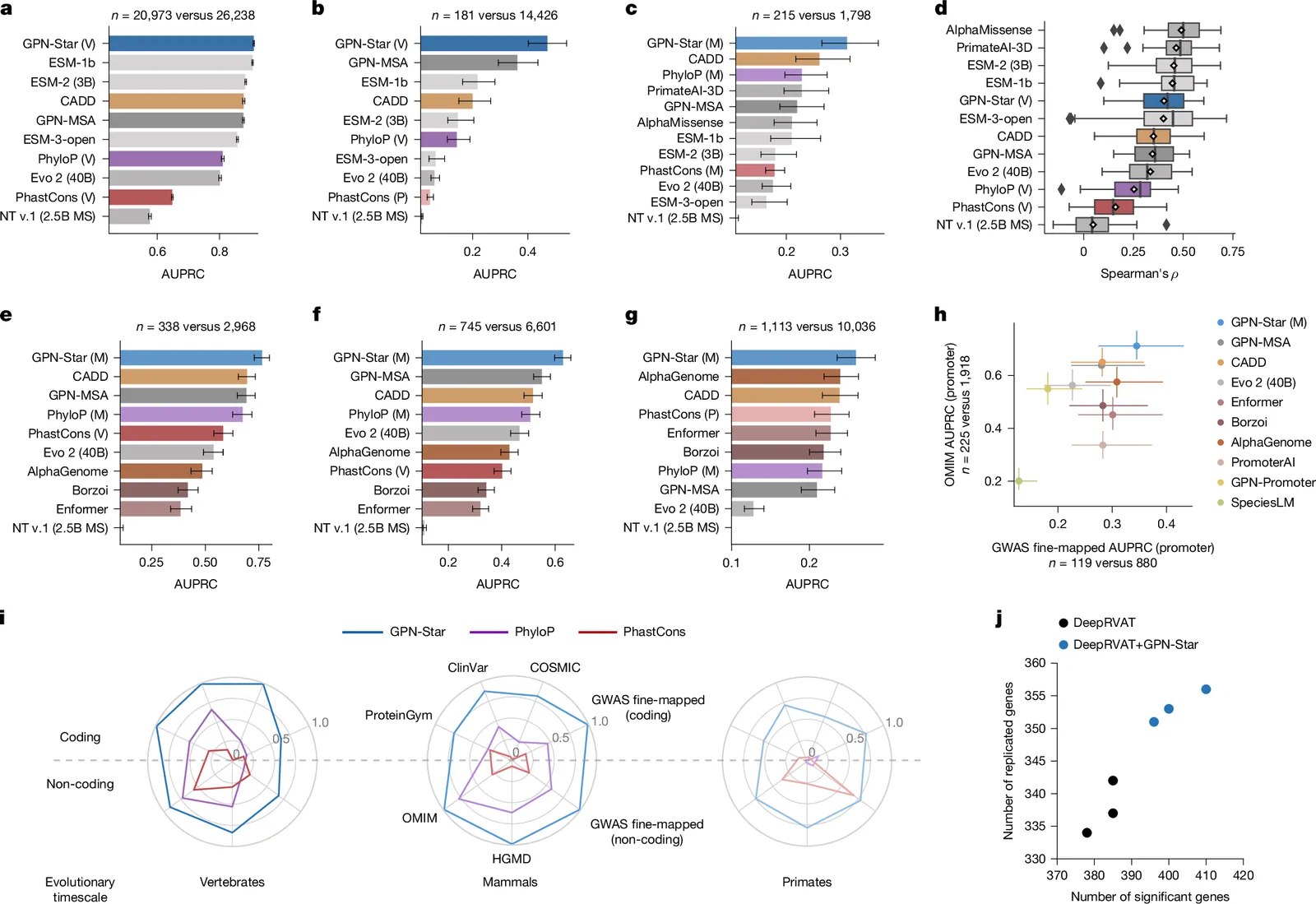
Performance of GPN-Star on human genome-wide variant effect prediction. Classification performance measured by AUPRC is shown in **a**–**c** and **e**–**h**; bar heights and dot positions indicate the AUPRC computed on the complete benchmark set and the error bars are 95% confidence intervals from 1,000 class-stratified bootstrap resamples. Sample sizes (positive versus negative class) are shown in each panel heading. **a**, ClinVar pathogenic versus benign missense variants. **b**, COSMIC high-frequency (>0.1%) versus gnomAD v.3 common missense variants. **c**, GWAS fine-mapped putatively causal (PIP > 0.9) versus non-causal (PIP < 0.01) missense variants across 65 UK Biobank traits. PIP, posterior inclusion probability. **d**, Spearman correlations with deep mutational scanning fitness scores for 31 human–protein assays in ProteinGym; each point is one assay. In each box, the centre line is the median, the diamond is the mean, the bounds are the 25th and 75th percentiles and the whiskers are the most extreme values within 1.5 times the interquartile range. **e**, OMIM pathogenic versus gnomAD v.3 common non-coding variants. **f**, HGMD pathogenic versus gnomAD v.3 common non-coding variants. **g**, GWAS fine-mapped putatively causal versus non-causal non-coding variants across 83 UK Biobank traits. **h**, OMIM pathogenic versus gnomAD v.3 common (*y* axis) and GWAS fine-mapped causal versus non-causal (*x* axis) promoter variants. **i**, Radar plots comparing GPN-Star with PhyloP and PhastCons at the vertebrate, mammal and primate timescales across the seven pathogenicity benchmarks; metrics were scaled linearly to 0–1, defined by the lowest and highest values across the nine models. **j**, RVAT on 34 UK Biobank quantitative traits (WES; 161,822 unrelated individuals of European ancestry), comparing DeepRVAT with and without the three GPN-Star predictions (V, M and P): number of significant genes (*x* axis) versus the number replicating previous larger studies (*y* axis). Each set of three same-coloured points denotes three random initializations.

**Extended Data Fig. 1:**
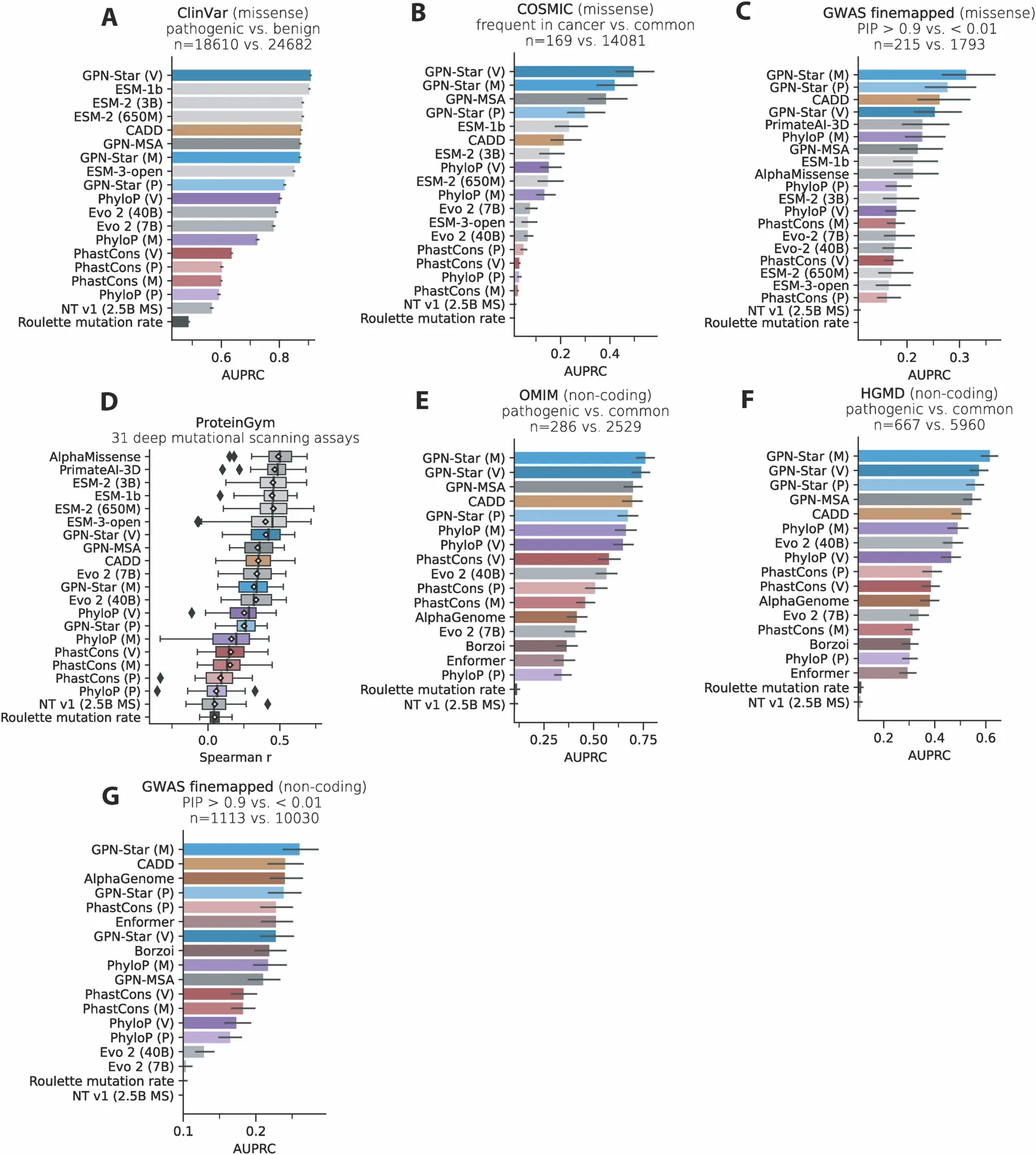
Variant effect prediction benchmarks with all competing models. The benchmarks are the same as in Fig. 2, while including GPN-Star, PhyloP and PhastCons with all three evolutionary timescales, Evo 2 with two parameter sizes and Roulette mutation rate estimates in the comparison. In (**A**)-(**C**) and (**E**)-(**G**), the bar height is the AUPRC computed on the complete benchmark set and the error bars in the barplots represent the 95% confidence intervals based on 1,000 class-stratified bootstrap resamples. Sample sizes (positive vs. negative class) are shown in the heading of each panel. In each box of the boxplot (**D**), the center line is the median, the center diamond is the mean, the box bounds are the 25th and 75th percentiles, and the whiskers extend to the most extreme values within 1.5 times the interquartile range.

Our evaluation of coding variants focused on missense variants, the most prevalent class. We first considered classifying pathogenic versus benign variants in ClinVar, a widely used clinical variant database containing expert-curated pathogenicity labels^24^. We compared our models with major genome-wide variant effect predictors, including classical evolutionary methods (PhyloP and PhastCons) that also use WGA, the ensemble model CADD^25^, recent single-sequence gLMs (Nucleotide Transformer 2.5B multispecies and Evo 2 40B) and GPN-MSA. GPN-Star (V) achieved the highest area under the precision-recall curve (AUPRC), matching the performance of the protein language model ESM-1b^26^ while outperforming ESM-2 (ref. ^27^) and ESM-3-open^28^ (Fig. 2a). For ease of visualization, only the best-performing GPN-Star model is shown in Fig. 2; results for all three versions are available in Extended Data Fig. 1.

We next considered somatic cancer variants from the COSMIC database^29^, constructing a benchmark to distinguish missense variants frequently observed in tumours from common missense variants in the general population (gnomAD^30^). As shown in Fig. 2b, GPN-Star (V) substantially outperformed all competing models, demonstrating strong predictive capability for pathogenicity beyond germline variants.

Beyond clinical variants, we also assessed performance on deep mutational scanning (DMS) data, functional assays that measure the fitness effects of all possible missense mutations in a given protein. Across 31 human DMS datasets from ProteinGym^31^, GPN-Star (V) outperformed all genome-wide models, although it slightly lagged behind the protein-specific models (Fig. 2d).

We also compared GPN-Star with two recent missense variant effect predictors, AlphaMissense^32^ and PrimateAI-3D^33^. These models were supervised on population allele frequency data, a highly informative signal for pathogenicity prediction, which is also used to define labels in databases such as ClinVar^34^. Interestingly, although both AlphaMissense and PrimateAI-3D achieved top performance on ProteinGym and ClinVar, a simple post hoc adjustment of GPN-Star (V) predictions using gnomAD allele frequencies boosted its performance on ClinVar to surpass PrimateAI-3D and approach that of AlphaMissense (Fig. 2d and Supplementary Fig. 1).

## Pathogenic non-coding variants

We next considered tasks involving non-coding variants, which are known to be especially challenging for predictive modelling. Here we demonstrate that GPN-Star is a powerful tool for identifying pathogenic non-coding variants in the human genome.

In addition to previous genome-wide variant effect prediction (VEP) models, we included three prominent sequence-to-function models—Enformer^35^, Borzoi^36^ and AlphaGenome^37^—which are trained on extensive functional genomics data and have been widely applied to non-coding variant interpretation. We evaluated the models on classifying non-coding pathogenic variants from the OMIM^38^ and HGMD^39^ databases, both of which contain expert-curated annotations of human disease-associated variants. GPN-Star (M) achieved the best performance on both benchmarks (Fig. 2e,f). Notably, the sequence-to-function models performed substantially worse than alignment-based gLMs and phylogenetic models on this task, consistent with recent findings^2,37^. Stratified analysis indicated that GPN-Star consistently achieves top performance across variant types, with the largest advantage over sequence-to-function models in distal enhancers (Supplementary Figs. 2, 3).

Given the critical role of promoter regions in transcription initiation and gene regulation, many specialized models have been developed to predict the impact of promoter variants. We therefore evaluated GPN-Star on promoter variants in OMIM and compared its performance with other methods, including three promoter-specific models: PromoterAI^40^, SpeciesLM^41^ and GPN-Promoter^2^. As shown in Fig. 2h, GPN-Star (M) demonstrated superior predictive performance compared with all competing models. In particular, the margin of improvement over the promoter-specific models is substantial.

## Fine-mapped variants in GWAS

Genome-wide association studies (GWAS) have been instrumental in identifying variants that contribute to genetic disease susceptibility. To further assess the use of GPN-Star, we evaluated its performance in classifying putatively causal versus non-causal missense variants resulting from fine-mapping of GWAS variants across 65 traits from the UK Biobank^42^. Among all competing models, GPN-Star (M) achieved the highest predictive performance on these fine-mapped missense variants. Notably, despite leveraging population allele frequency information, both AlphaMissense and PrimateAI-3D were substantially outperformed by GPN-Star in this task (Fig. 2c).

We next evaluated the models on fine-mapped non-coding GWAS variants across 83 traits from the UK Biobank^42^, again assessing their ability to distinguish putatively causal versus non-causal variants. GPN-Star (M) continued to outperform all other models on this benchmark (Fig. 2g). Although sequence-to-function models (Enformer, Borzoi and AlphaGenome) exhibited moderate performance, Evo 2 showed relatively limited predictive value, as was previously observed^2^. Stratified analysis showed robust performance of GPN-Star across variant types (Supplementary Fig. 4). For fine-mapped variants located in promoter regions, GPN-Star (M) again outperformed all models, including the promoter-specific models PromoterAI, SpeciesLM and GPN-Promoter (Fig. 2h).

## Rare variant association testing

Having observed the strong performance of GPN-Star in predicting pathogenic and fine-mapped variants, we explored its use in rare variant association testing (RVAT), an important but challenging task in statistical genetics. The great interest in RVAT stems from the fact that most large-effect variants tend to be rare, as they are subject to negative selection pressure^43^. To overcome statistical power issues with low-frequency variants, RVAT is typically carried out at the gene level by aggregating variants in each gene^44^. These testing procedures often rely on variant annotations that reflect functional importance to prioritize variants in the aggregation. DeepRVAT^5^ is a recent method that uses a deep set network to integrate such variant annotations for RVAT. By combining an expressive deep learning framework with a powerful set of variant annotations, it demonstrated improved statistical power and computational efficiency compared with previous methods in extensive evaluations on UK Biobank whole-exome sequencing (WES) data^5^.

We conducted RVAT experiments using DeepRVAT, enhancing the annotations used in the published version with all three GPN-Star predictions during both the training and association testing phases. Following the published benchmarking procedure^5^, we performed RVAT experiments using DeepRVAT on 34 quantitative traits in UK Biobank with the WES data from 161,822 unrelated individuals of European ancestry, retaining variants with minor allele frequency (MAF) <0.1% (Methods). To account for differences in stochastic model initialization, this procedure was run three times with different random seeds. As shown in Fig. 2j, adding the predictions from the GPN-Star models as variant annotations into DeepRVAT resulted in an increase in the number of discovered genes at a family-wise error rate <0.05 (on average across runs, 402 genes compared with 383 from the original method). Among the significant discoveries, there were also more gene–phenotype associations that replicated in conventional RVAT studies on UK Biobank with larger sample sizes^45,46^ (Methods) (on average 353 compared with 338), indicating high robustness of the further discoveries. Notably, the original DeepRVAT already includes state-of-the-art missense variant effect predictors such as AlphaMissense and PrimateAI, as well as the sequence-to-function model DeepSEA^47^, in the annotations. Nonetheless, GPN-Star seemed to offer complementary information and boosted the power of the tests.

## Complex trait heritability

To investigate the prediction of causal variants for complex trait GWAS, we turned to stratified linkage disequilibrium score regression (S-LDSC)^48^. S-LDSC is a principled way to estimate the informativeness of an annotation for complex trait heritability while leveraging the signal from all single nucleotide polymorphisms (SNPs), including those not confidently fine-mapped or genome-wide significant. It also serves as a backbone for functionally informed fine-mapping^49^ and polygenic risk scores^50^. Because S-LDSC requires scoring about 10 million variants, it was feasible to evaluate only the most scalable models (or those with precomputed scores). We binarized model scores to select a specific fraction of common variants. In our main analysis, we used the top 0.1% most constrained variants. We ran S-LDSC separately for each model score while conditioning on 96 baseline features and meta-analysed the results across 106 independent traits^23^.

**Fig. 3:**
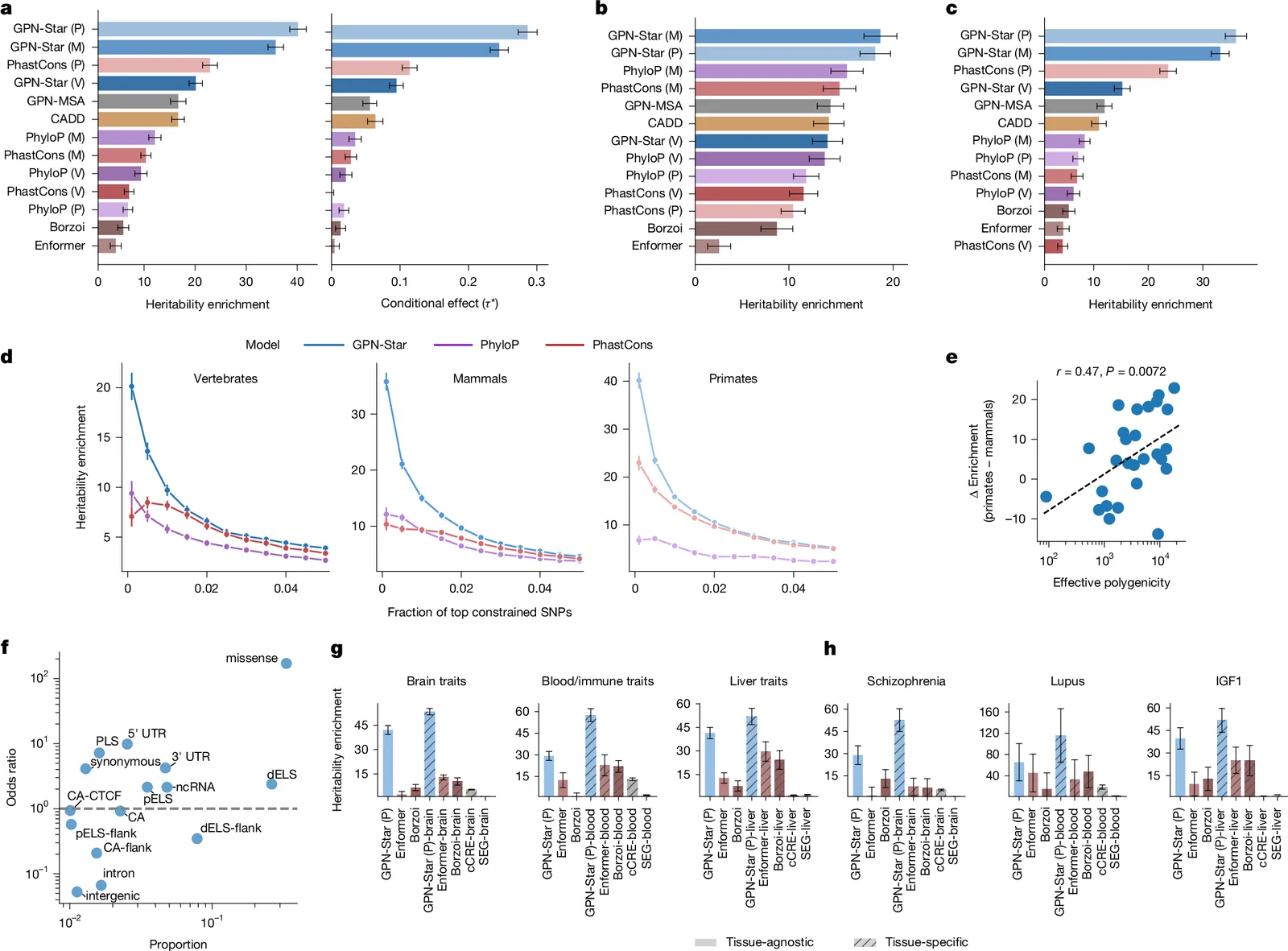
SNP heritability analyses in human complex traits. **a**, Informativeness of different models for complex trait heritability. We binarized model scores to select the top 0.1% most constrained common variants. We ran S-LDSC separately for each model annotation while conditioning on 96 baseline features and meta-analysed the results across 106 independent traits. Heritability enrichment is the proportion of heritability explained by an annotation divided by the size of the annotation. The conditional effect (${\tau }^{\star }$) measures the unique contribution to heritability that is not explained by existing annotations. **b**, Performance restricting model annotations to coding regions. **c**, Performance restricting model annotations to non-coding regions. **d**, Comparison varying the fraction of top constrained SNPs. **e**, Difference in enrichment between GPN-Star (P) and (M) as a function of estimated effective polygenicity across 27 traits. The *P *value is from a one-sided *t*-test on the Pearson correlation (d.f. = 25). Dashed line is an ordinary least squares fit. **f**, Types of common variants prioritized by GPN-Star (P). Variants are annotated by a combination of Ensembl consequences and ENCODE SCREEN candidate *cis*-regulatory elements. Only types with a proportion above 1% are shown. Odds ratio is given with respect to the bottom 99.9% of common variants. **g**, Comparison of tissue-agnostic and tissue-specific adaptations of GPN-Star (P), Enformer, Borzoi and tissue-specific baselines across selected trait groups (brain, 30 traits; blood/immune, 15 traits; liver, 15 traits). **h**, Comparison of tissue-agnostic and tissue-specific adaptations of GPN-Star (P), Enformer, Borzoi and tissue-specific baselines across selected individual traits. In **a**–**d**,**g** and **h**, annotations are defined on 9,997,231 reference SNPs, of which 5,961,159 are common. The bar and line heights are point estimates from S-LDSC. The error bars represent standard errors estimated from block jackknife over 200 genomic windows.

Since the early work on S-LDSC, conservation scores have been found to be the most enriched annotation for complex trait heritability^48^. More recently, primate-specific conservation—particularly PhastCons (P)—has emerged as the state-of-the-art for complex trait heritability enrichment^7,8^. Notably, GPN-Star (P) substantially improves on this result, followed by GPN-Star (M) (Fig. 3a). These improvements are even more striking when examining the heritability coefficient ${\tau }^{\star }$ (Fig. 3a), which quantifies the unique contribution of an annotation to heritability after adjusting for baseline features (Methods). Although per-trait enrichment estimates are noisier, GPN-Star (P) or GPN-Star (M) consistently rank at the top (Supplementary Fig. 5). This advance is particularly notable given the long-standing role of complex trait heritability enrichment as a meaningful benchmark in human genetics.

**Extended Data Fig. 2:**
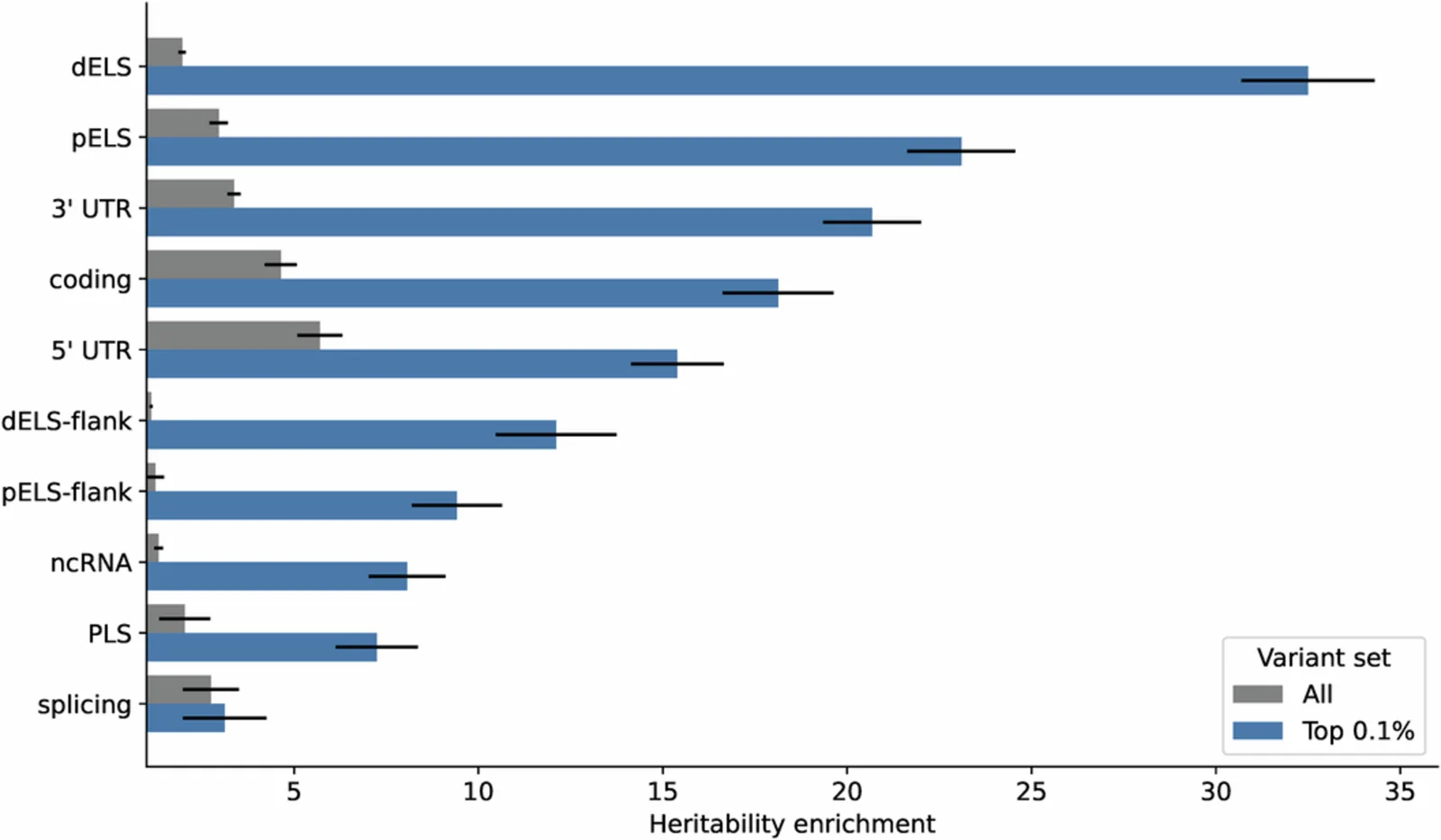
Heritability enrichment across 106 GWAS stratified by genomic functional regions. Same as in Fig. 3, here we show the enrichment among the top 0.1% most constrained variants by GPN-Star (P) scores restricted to different functional regions of the genome (blue bars), compared with all variants in each functional region (gray bars). The bar and line heights are point estimates from S-LDSC. The error bars represent standard errors estimated from block jackknife over 200 genomic windows. dELS: distal enhancer-like signatures; pELS: proximal enhancer-like signatures; PLS: promoter-like signatures. These annotations were obtained from ENCODE cCRE v4. The splicing category encompasses all types of splicing variants.

The improvement of GPN-Star persists when restricting to top-ranked variants in coding and non-coding regions separately (Fig. 3b,c), with stronger gains in non-coding regions. We further performed an analysis of the best-performing model GPN-Star (P), stratifying the non-coding region by functional category, and found particularly strong improvements in enhancer regions (Extended Data Fig. 2). Moreover, across a range of binarization thresholds and all three evolutionary timescales, GPN-Star consistently outperforms previous conservation scores (Fig. 3d).

## Relevant evolutionary timescales

**Extended Data Fig. 3:**
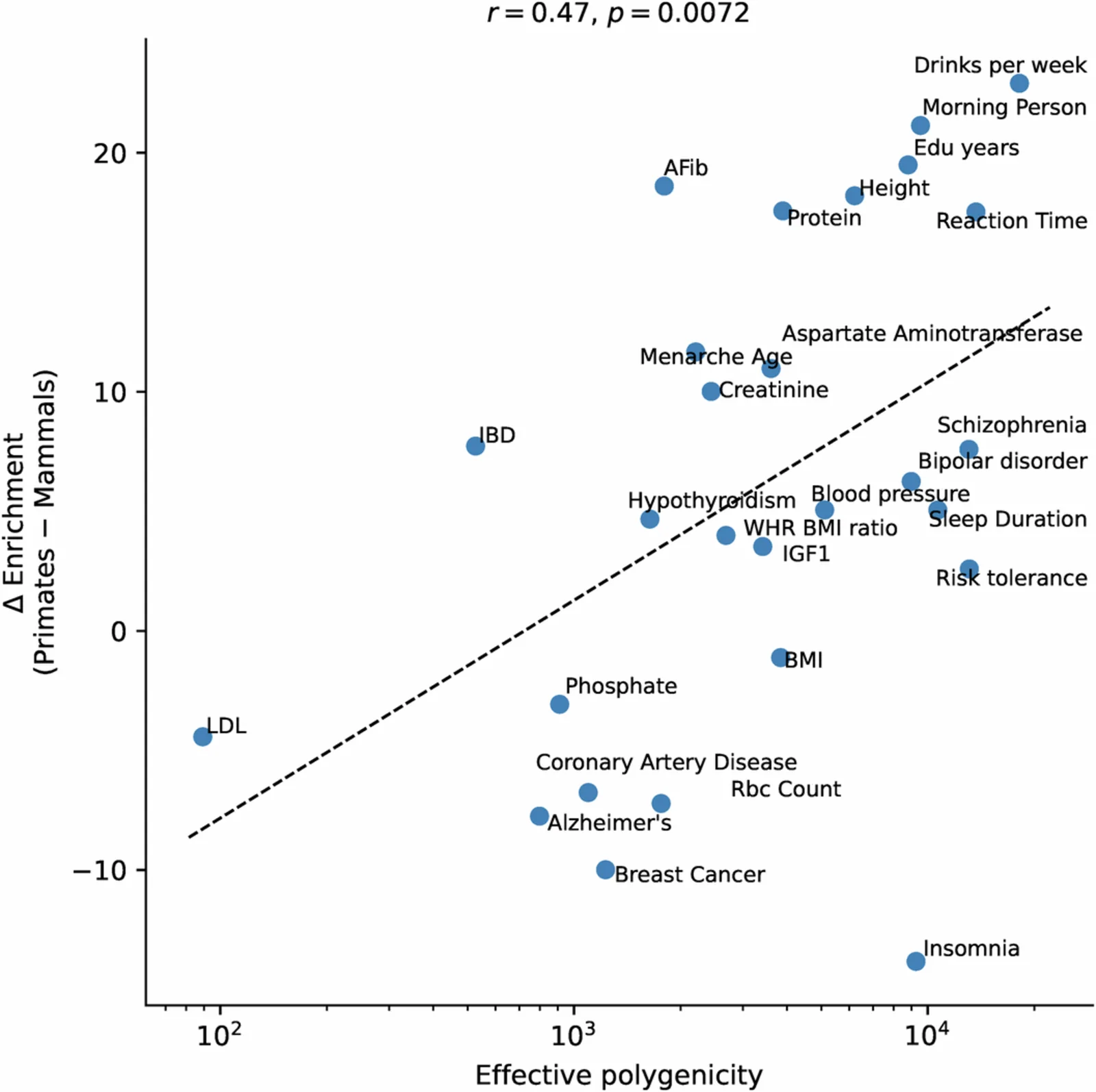
Difference in enrichment between GPN-Star (P) and (M) as a function of estimated effective polygenicity with every trait labeled. GPN-Star (M) attains greater enrichment for LDL cholesterol (effective polygenicity =89), whereas GPN-Star (P) performs better for schizophrenia (effective polygenicity =13,069). These results suggest that the evolutionary timescale most informative for functional constraint depends on the genetic architecture of the trait, with recent primate evolution playing an especially important role for highly polygenic traits. The p-value is from a one-sided *t*-test on the Pearson correlation (df = 25). The dashed line is an ordinary least squares fit.

The VEP results summarized in Fig. 2i underscore the importance of the timescale represented in the training data of evolutionary models. We further investigated how evolutionary timescale shapes the variants prioritized by GPN-Star models for complex trait heritability. We found that GPN-Star (P) preferentially captures variants contributing to highly polygenic traits^51^ (Extended Data Fig. 3), whereas GPN-Star (M) is relatively more informative for less polygenic traits, showing a connection between evolutionary timescale and the genetic architecture of complex traits (Fig. 3e). We also characterized the functional classes of variants prioritized by the top-performing model GPN-Star (P), finding large proportions of missense variants (33%, 170-fold enriched), followed by variants in regions with distal enhancer elements (26%, 2.4-fold enriched) and their flanks (8%, 0.35-fold depleted) (Fig. 3f). The importance of distal enhancer variants for complex trait heritability should not be understated, as they remain a major weakness of present sequence-to-function models^52^ (including the latest AlphaGenome^37^), as well as alignment-free gLMs (including the largest Evo 2; ref. ^2^). More analyses of model overlap and variant consequence enrichments are presented in Supplementary Information.

## Tissue-specific heritability signals

**Extended Data Fig. 4:**
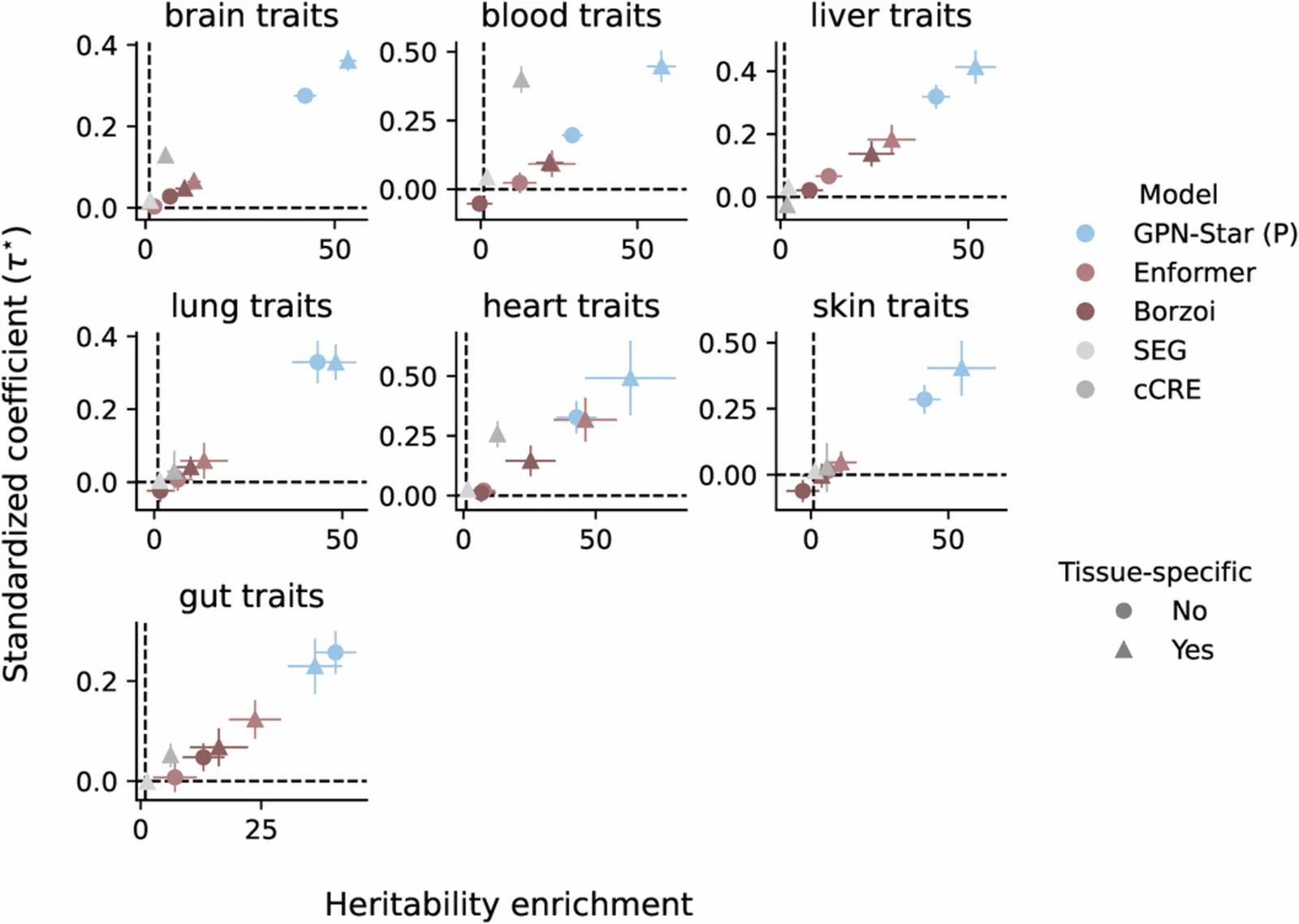
Heritability enrichment and standardized coefficient (${{\boldsymbol{\tau }}}^{{\boldsymbol{\star }}}$) by S-LDSC analysis for tissue-specific and tissue-agnostic annotations. Results are shown for all seven tissues and corresponding trait groups (brain: 30 traits; blood/immune: 15 traits; liver: 15 traits; gut: 13 traits; heart: 7 traits; skin: 7 traits; lung: 6 traits). Annotations are defined on 9,997,231 reference SNPs, of which 5,961,159 are common. The dots represent point estimates from S-LDSC. The error bars represent standard errors estimated from block jackknife over 200 genomic windows.

As the final analysis of complex trait heritability, we investigated the performance of tissue-specific scores. Sequence-to-function models such as Enformer^35^ and Borzoi^36^ do not provide a single variant effect score but instead predict changes in activity across thousands of functional genomics tracks representing different assays and tissues. In our comparisons, we used Enformer and Borzoi scores aggregated across all tracks (tissue agnostic) as well as in seven specific tissues, following procedures from ref. ^53^ Given the low enrichment of tissue-agnostic Enformer and Borzoi scores when meta-analysed across all traits (Fig. 3a), we investigated the performance of tissue-specific scores. We meta-analysed these scores only across traits where we expected the tissue to be relevant (Supplementary Table 2). Tissue-specific Enformer and Borzoi scores consistently outperform their tissue-agnostic scores (Fig. 3g, Extended Data Fig. 4 and Supplementary Fig. 6).

On the other hand, GPN-Star trained solely on DNA sequences is intrinsically tissue agnostic. Motivated by this observation, we devised a simple approach to incorporate tissue specificity into GPN-Star by only considering top-scoring variants located near tissue-specifically expressed genes (SEG) (inspired by LDSC-SEG^54^) or in tissue-specific candidate *cis*-regulatory elements (cCREs) (obtained from ENCODE v.4^53^). Although this improved performance for most tissues—such as brain and blood/immune—it did not consistently surpass the performance of tissue-agnostic GPN-Star annotations, potentially because the present approach to incorporating tissue specificity remains suboptimal (Fig. 3g and Extended Data Fig. 4). Nevertheless, GPN-Star outperformed Enformer and Borzoi across all seven tissues. We also verified that the annotation by SEG and cCRE alone without model scores exhibited much lower heritability enrichment, confirming that the observed performance is still largely attributed to GPN-Star (Fig. 3g,h and Supplementary Fig. 6). Zooming in on example traits, brain-specific GPN-Star showed the highest enrichment for schizophrenia, blood/immune-specific GPN-Star performed best for lupus, and both tissue-agnostic and liver-specific GPN-Star yielded similar results for IGF1 (Fig. 3h). These findings highlight the importance of incorporating tissue-specific information in variant effect prediction.

## Interpretability of GPN-Star

We examined the representations learned by GPN-Star and found that its embeddings distinguish major genomic functional elements, with stronger separation for evolutionarily conserved regions (Supplementary Information; Supplementary Figs. 7–9). These results suggest that GPN-Star is aware of core functional elements of the genome when making predictions.

**Extended Data Fig. 5:**
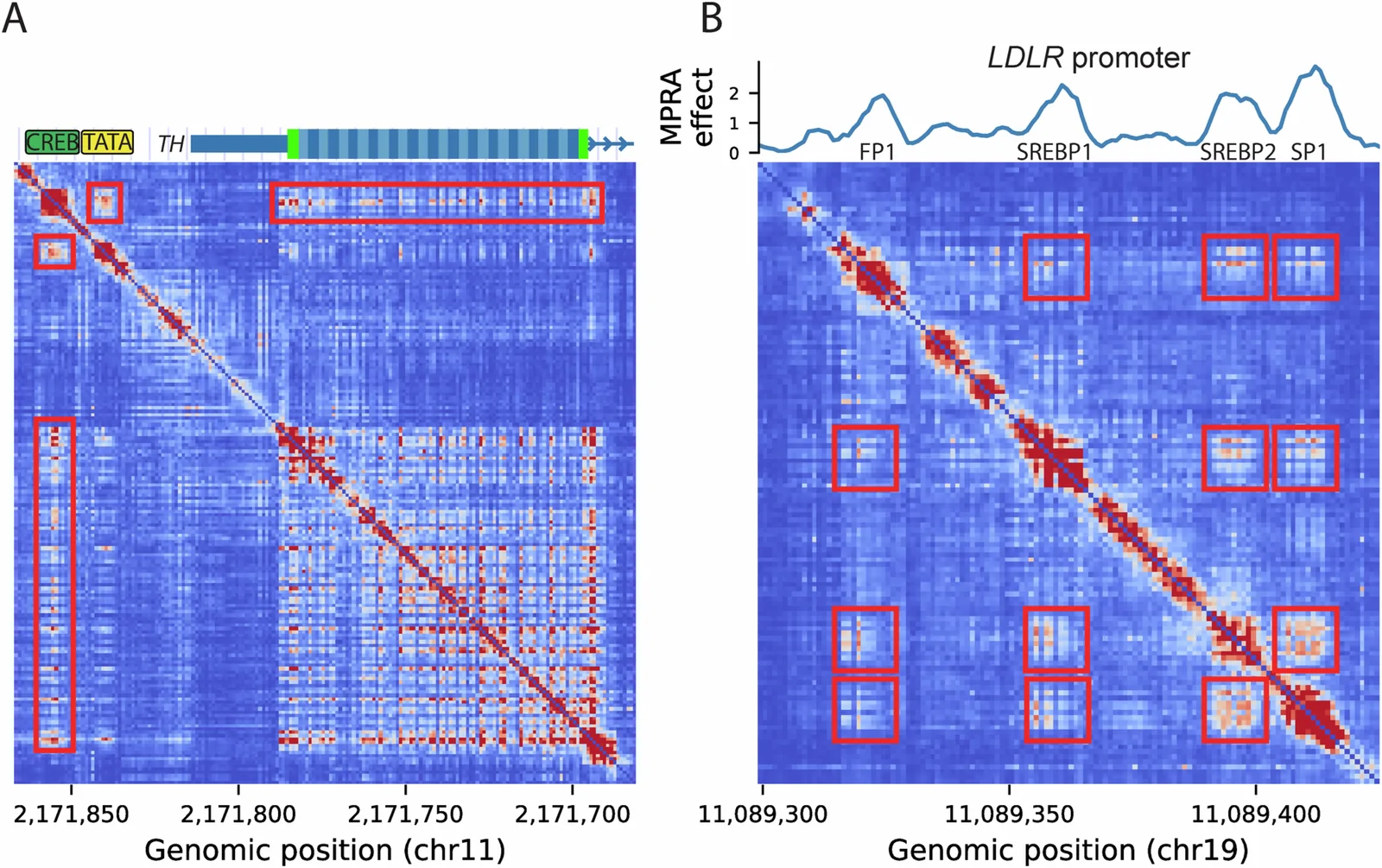
Interpretation of the GPN-Star (M) 85 M-parameter model, revealing functional elements and their dependencies. The intensity of the heatmap at position i,j corresponds to the influence of the nucleotide at position i on the predicted nucleotide probabilities at position j. (**A**) Nucleotide dependency map in the promoter and first exon of *TH*, which encodes the enzyme tyrosine hydroxylase. This analysis revealed a strong interaction block within the coding region and another at a binding site of the transcription factor CREB, where mutations are known to cause tyrosine hydroxylase deficiency and dystonia^40,64^ (also see Supplementary Fig. 10). The model predicts that CREB depends on both the TATA box and, interestingly, the coding region. (**B**) Nucleotide dependency map in the promoter of *LDLR*, implicated in familial hypercholesterolaemia. This promoter contains well-known TFBS and has been studied using massively parallel reporter assays (MPRA)^7,65^. MPRA effect in the figure refers to smoothed absolute log fold change. TFBS locations can be predicted well from the block structure in the nucleotide dependency map (also see Supplementary Fig. 11)^41^. Furthermore, the model also predicts dependencies between TFBS, including the well-known interaction between SREBP2 and SP1^66^.

Site-independent models such as PhyloP cannot, by definition, capture dependencies among nucleotides. By contrast, alignment-free gLMs have been shown to learn dependencies among well-known interacting elements, for example, in a transcription-factor binding site (TFBS) motif or between splice donors and acceptors^41^. To further probe understanding in GPN-Star of genomic syntax, we analysed learned nucleotide dependencies by systematically mutating each position and quantifying the resulting probability changes at other positions in the sequence^41,55^. This analysis showed biologically meaningful co-evolutionary signals, including dependencies among TFBSs, coding regions and splice sites, as well as evidence for primate-specific regulatory constraint. Representative examples are shown in Extended Data Fig. 5, with more case studies presented in Supplementary Figs. 10–13.

**Extended Data Fig. 6:**
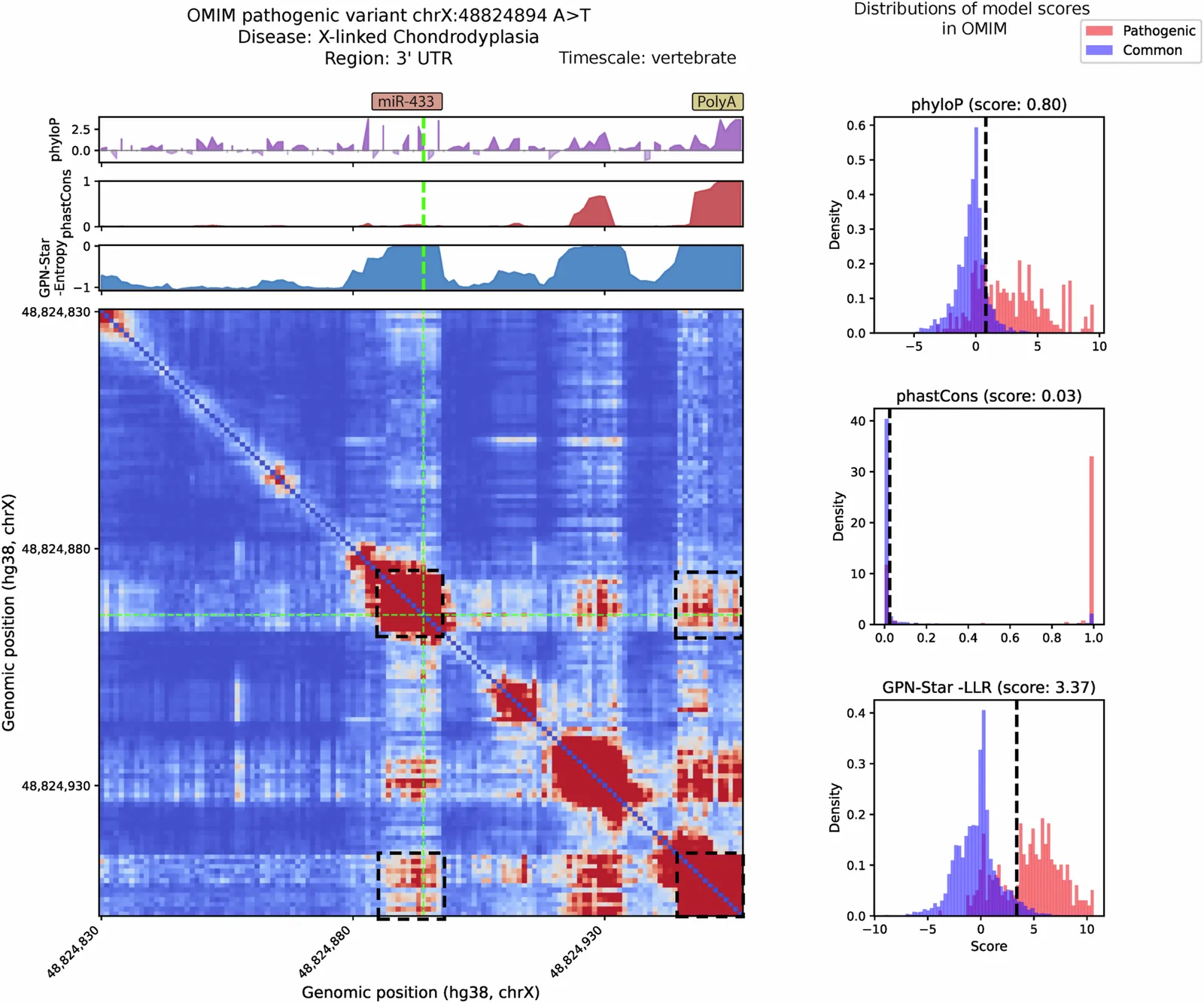
Case study on a pathogenic variant in a 3′ UTR at the vertebrate evolutionary timescale. This is a pathogenic variant in OMIM known to cause X-linked chondrodysplasia. It is correctly classified as deleterious by GPN-Star while PhyloP and PhastCons produce near-neutral scores (right column). The left column shows the nucleotide dependency map, as well as PhyloP, PhastCons, and GPN-Star prediction tracks in the genomic window chrX:48824830-48824958 centered around the variant (green line), overlaid with functional elements identified in an experimental study. The variant is located in a miR-433 binding site and disrupts the seed sequence, thereby increasing mRNA stability. The nucleotide dependency map by GPN-Star identifies the miRNA binding site as a functionally important element, as well as its interdependency with the polyadenylation signal.

**Extended Data Fig. 7:**
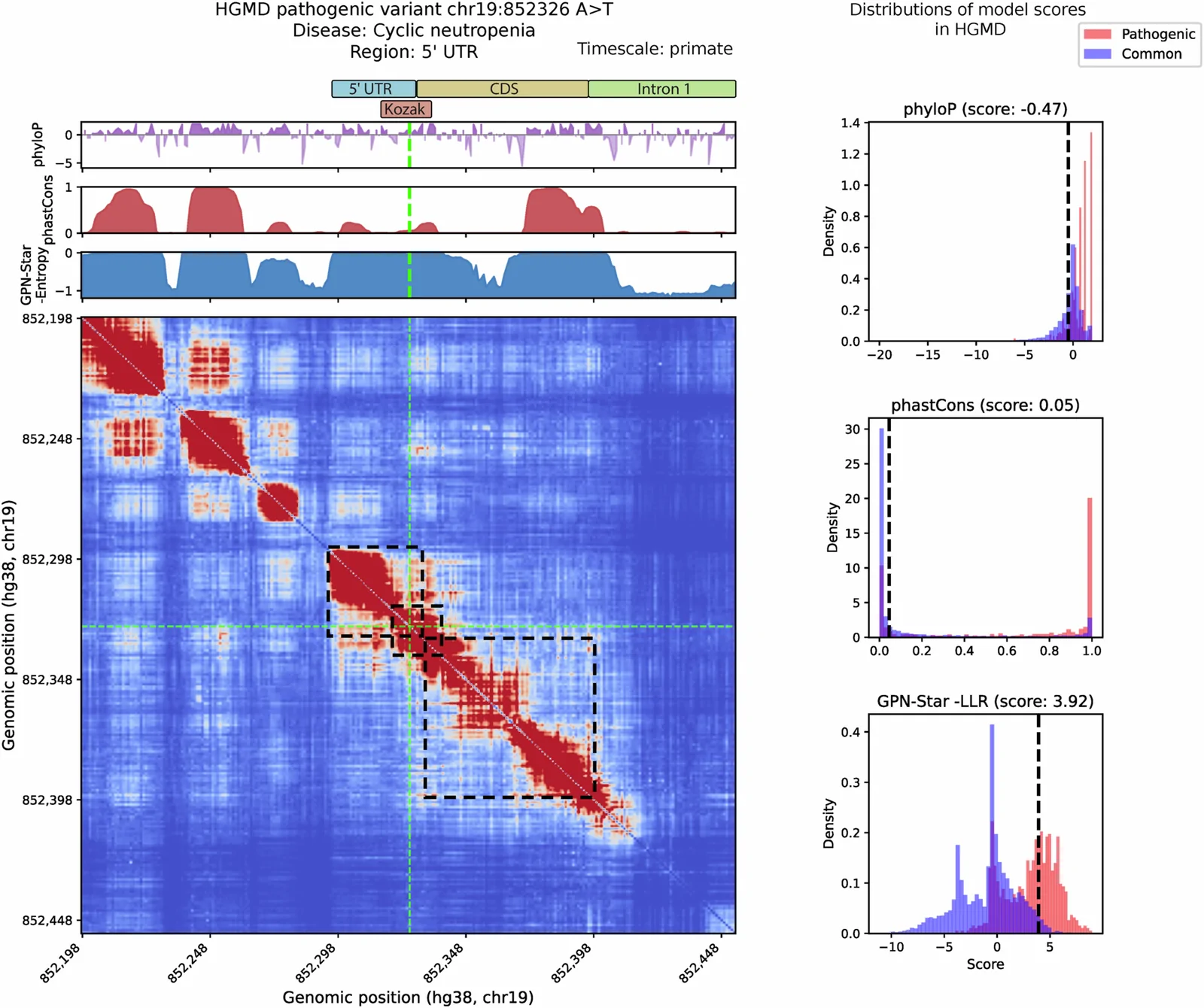
Case study on a pathogenic variant in a 5′ UTR at the primate evolutionary timescale. This is a pathogenic variant in HGMD known to cause cyclic neutropenia. It is correctly classified as deleterious by GPN-Star while PhyloP and PhastCons produce near-neutral scores (right column). The left column shows the nucleotide dependency map, as well as PhyloP, PhastCons, and GPN-Star prediction tracks in the genomic window chr19:852198-852454 centered around the variant (green line), overlaid with functional elements identified in an experimental study. The variant is located in the Kozak sequence and disrupts the critical purine at position −3. Nucleotide dependency map by GPN-Star identifies the Kozak sequence as a functionally important element, as well as its dependencies with the surrounding 5′ UTR and the initial CDS region.

**Extended Data Fig. 8:**
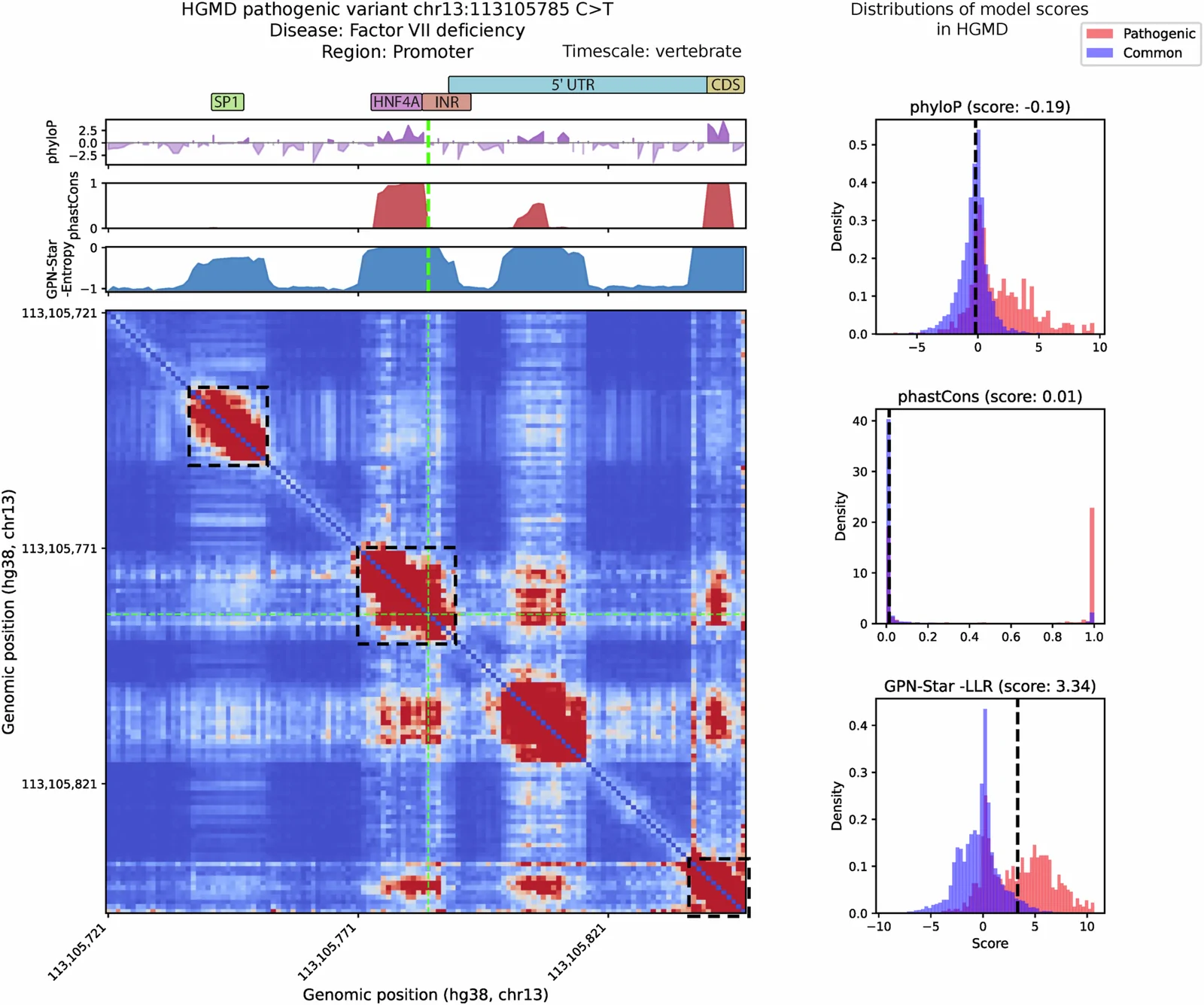
Case study on a pathogenic variant in a promoter region at the vertebrate evolutionary timescale. This is a pathogenic variant in HGMD known to cause factor VII deficiency. It is correctly classified as deleterious by GPN-Star while PhyloP and PhastCons produce near-neutral scores (right column). The left column shows the nucleotide dependency map, as well as PhyloP, PhastCons, and GPN-Star prediction tracks in the genomic window chr13:113105721-113105977 centered around the variant (green line), overlaid with functional elements identified in an experimental study. The variant is located at the boundary of an HNF4A binding site and the initiator element. Nucleotide dependency map by GPN-Star identifies the HNF4A binding site and the initiator element as functionally important elements, as well as their dependencies with a 5′ UTR element and the initial CDS region.

**Extended Data Fig. 9:**
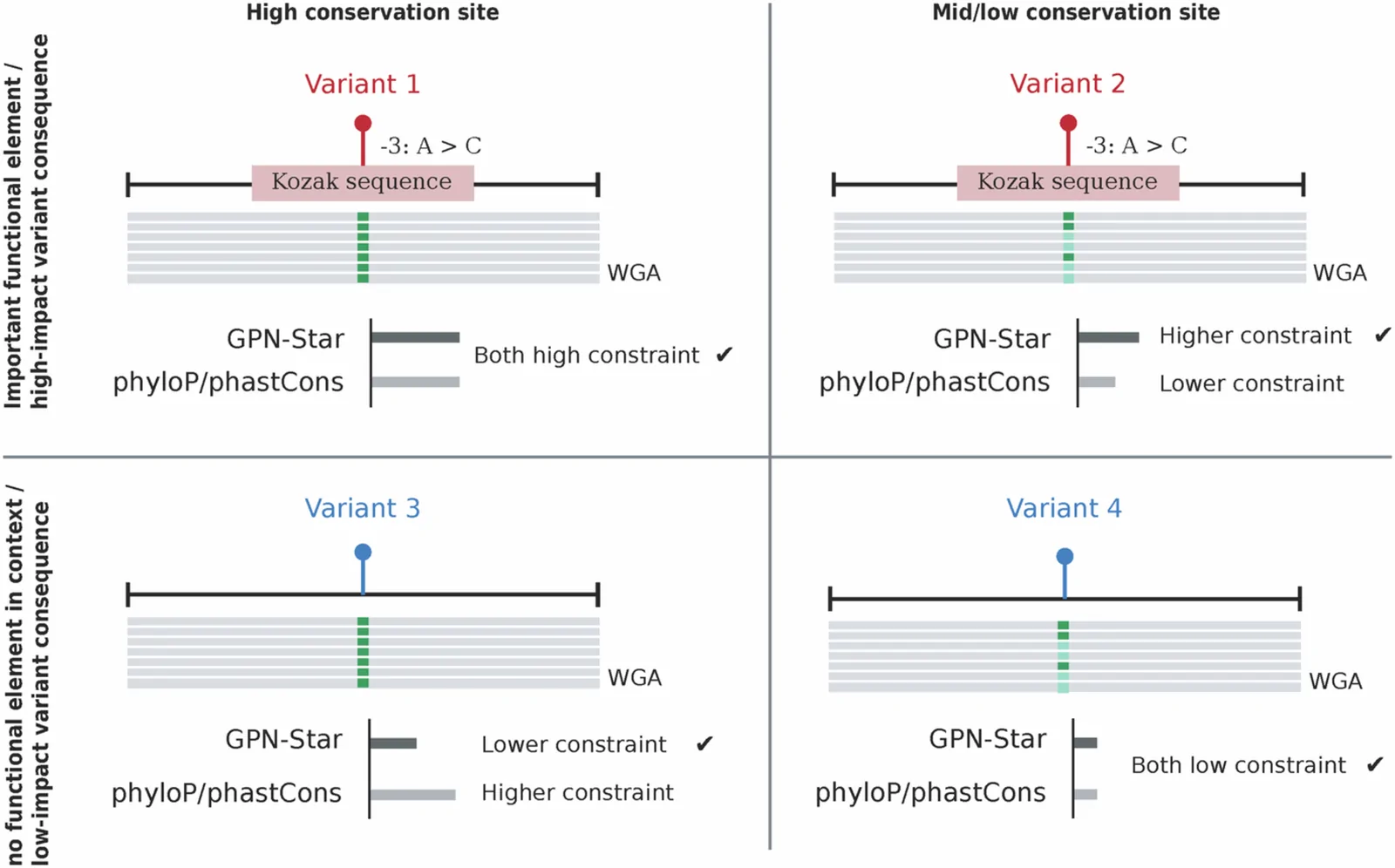
Schematics illustrating representative scenarios in which GPN-Star yields more context-aware constraint predictions than traditional conservation scores. Variants 1 and 2 correspond to high-impact, pathogenic variants, whereas variants 3 and 4 correspond to putatively low-impact, benign variants. Variant 1 lies within a functionally important element and at a strongly conserved site according to the alignment; consequently, both GPN-Star and conservation-based metrics make high constraint predictions. Variant 4 is located outside functional elements and at a weakly conserved site; as such, both GPN-Star and conservation scores produce low constraint predictions. Variant 2 occurs at a weakly conserved site but falls within a functionally important element and has a highly disruptive molecular consequence. In this setting, GPN-Star makes a higher constraint prediction than traditional conservation scores. An empirical instance of this pattern is provided in Extended Data Fig. 7. By contrast, Variant 3 occurs at a strongly conserved site but lacks surrounding functional elements or has low-impact molecular consequences. In such cases, GPN-Star yields lower constraint predictions than the traditional conservation scores. This behavior is illustrated by the distribution of predicted scores at perfectly conserved sites in Supplementary Fig. 14.

These results demonstrate that GPN-Star can leverage co-evolutionary signals embedded in genomic sequence context to learn meaningful nucleotide dependencies that align with known functional dependencies, representing a notable advance over traditional conservation scores. Finally, we used nucleotide dependency to examine a few cases in our disease variant benchmarks where GPN-Star correctly predicts them to be deleterious, whereas PhyloP and PhastCons predict neutral scores (Extended Data Figs. 6, 7 and 8). We found that these variants do not exhibit strong site-wise conservation but reside in functionally important elements with distinct sequence contexts shown by nucleotide dependencies. As schematically depicted in Extended Data Fig. 9, GPN-Star effectively leverages these contextual signals to outperform traditional conservation metrics. For example, in the most extreme case of perfectly conserved sites, traditional conservation scores are unable to distinguish different variant types, whereas GPN-Star tends to predict more deleterious scores for higher impact variants (Supplementary Fig. 14).

## Genome-wide evolutionary constraints

To more directly assess the connection between model predictions and evolutionary constraints in the genome, we leveraged allele frequency data from gnomAD v.3.1.2, which aggregates whole-genome sequencing samples from 76,156 human individuals^30^. Allele frequencies in the human population serve as informative indicators of selective constraint: more deleterious alleles tend to have lower frequencies as a result of purifying selection^9,22,30^.

**Fig. 4:**
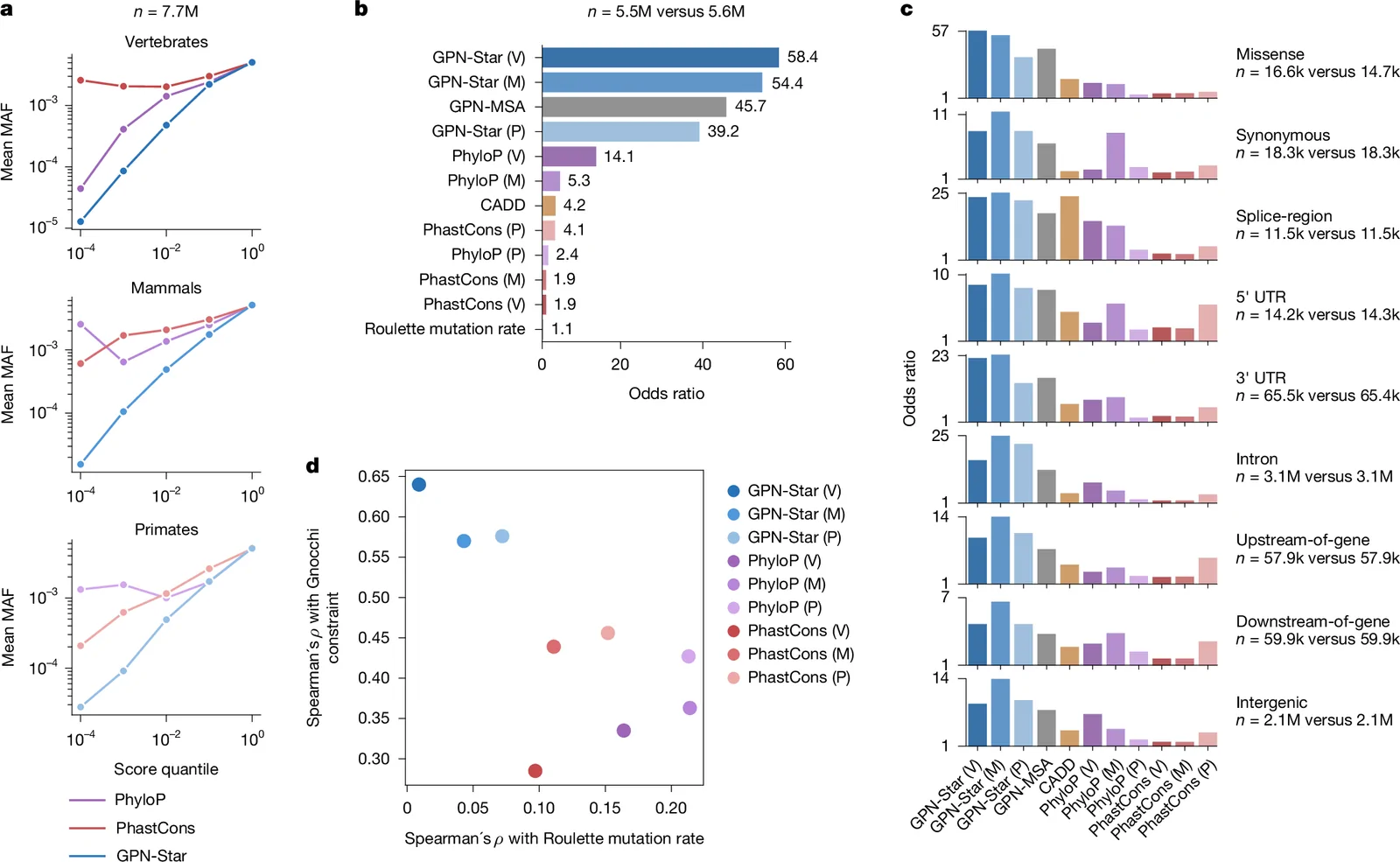
GPN-Star scores reflect evolutionary constraints on the human genome. **a**, Mean MAF for quantile bins ([0, 10^−4^], (10^−4^, 10^−3^], …, (10^−1^, 1]) defined by GPN-Star, PhyloP and PhastCons on the basis of the vertebrate, mammal and primate alignments at the gnomAD biallelic sites on chromosome 22. **b**,**c**, Enrichment of rare (singletons) versus common (MAF > 5%) gnomAD variants in the tail of deleterious scores (the threshold was chosen such that each score yielded 30 false discoveries): genome-wide enrichment (**b**) and enrichments stratified by molecular consequences of the variants (**c**). The rare variants were downsampled to match the number of common variants in each category. **d**, Performance comparison of GPN-Star with PhyloP and PhastCons at the three evolutionary timescales on correlation with Roulette^62^ mutation rate estimates on chromosome 22 (*x* axis) and correlation with Gnocchi^30^ constraint estimates (*y* axis). K, thousand; M, million.

In this evaluation, we focused on comparison with PhyloP and PhastCons, which also learn evolutionary constraints from WGA data. To assess how well each model captures the relationship between allele frequency and constraint, we considered the vertebrate, mammal and primate versions of each model and obtained predictions for all gnomAD v.3 variants on chromosome 22, the held-out chromosome not used in training GPN-Star. We then compared the mean minor allele frequencies in several quantile bins defined by different models. As shown in Fig. 4a, across all three evolutionary timescales, variants in lower GPN-Star quantile bins have consistently lower average allele frequencies compared with those in corresponding PhyloP and PhastCons bins, suggesting that GPN-Star more accurately captures selective constraints in the human genome.

**Extended Data Fig. 10:**
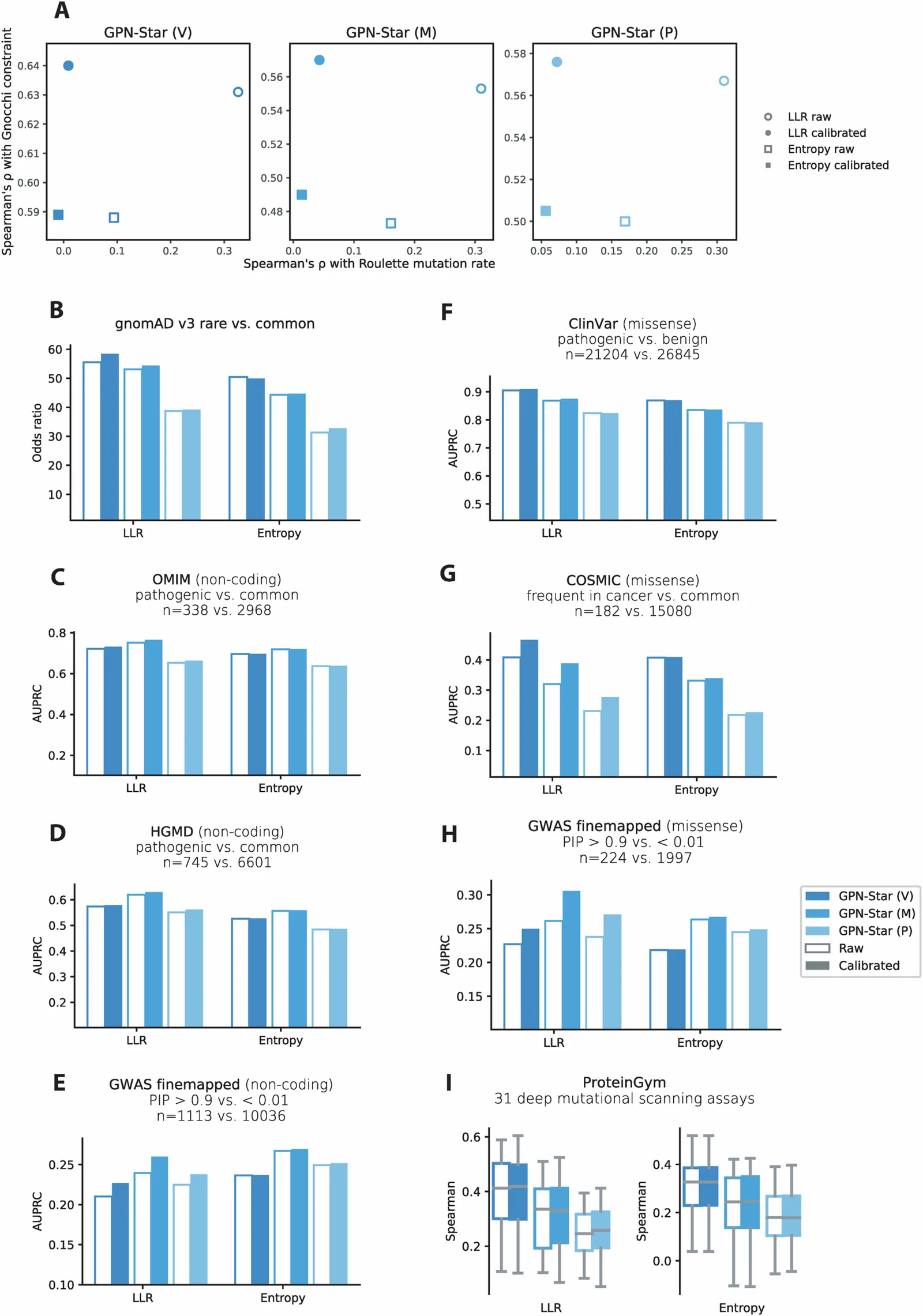
Mutation rate calibration. (**A**) Spearman correlations between GPN-Star predictions and Roulette mutation rate estimates (x axis) and Gnocchi constraint scores (y axis) before and after calibration. (**B**)-(**I**) Performance of GPN-Star predictions on variant effect prediction benchmarks before and after calibration. In (**B**)-(**H**), the bar height is the AUPRC computed on the complete benchmark set and the error bars represent the 95% confidence intervals based on 1,000 class-stratified bootstrap resamples. Sample sizes (positive vs. negative class) are shown in the heading of each panel. In each box of the boxplot (**A**), the center line is the median, the center diamond is the mean, the box bounds are the 25th and 75th percentiles, and the whiskers extend to the most extreme values within 1.5 times the interquartile range.

We next performed a more quantitative evaluation focusing on the most deleterious tails of the model score distributions, a regime especially important in many human genetics applications. We quantified the enrichment of rare variants relative to common variants in the most constrained tail predicted by each model. Here we defined rare variants as singletons and common variants as those with allele frequency >5%. Because rare variants are, on average, more deleterious, a model with more accurate constraint predictions should show higher enrichment of singletons in its most constrained tail. As shown in Fig. 4b and Supplementary Fig. 15a, all three GPN-Star models yielded substantially higher enrichment of rare variants than did PhyloP, PhastCons or CADD. Among the GPN-Star models, the vertebrate model showed the strongest enrichment overall and outperformed GPN-MSA (also trained on vertebrate genomes). When stratifying variants by molecular consequences, GPN-Star again achieved the highest enrichment in every category (Fig. 4c). Notably, GPN-Star (V) performed the best for missense variants, whereas GPN-Star (M) led for the synonymous and non-coding categories, mirroring trends observed in previous benchmarks. Lastly, our procedure for accounting for local mutation rate variation substantially reduced correlations with mutation rate estimates while improving performance across most downstream benchmarks (Fig. 4d and Extended Data Fig. 10; Supplementary Information).

## GPN-Star for model organisms

GPN-Star requires only a multispecies WGA and the corresponding phylogeny to predict variant effects, making it readily applicable to species for which genome-wide variant interpretation has remained challenging. To demonstrate this, we trained GPN-Star models on five important model organisms:* Mus musculus*, *Gallus gallus*, *Drosophila melanogaster*, *Caenorhabditis elegans* and *Arabidopsis thaliana*. In each case, we showed that the learned constraints are highly informative for interpreting genetic variation in that species. For each of the five species, we collected a WGA dataset comprising aligned genomes from 18 to 135 species and applied training procedures similar to those used for the human models, with only minor adjustments (Supplementary Table 1).

**Fig. 5:**
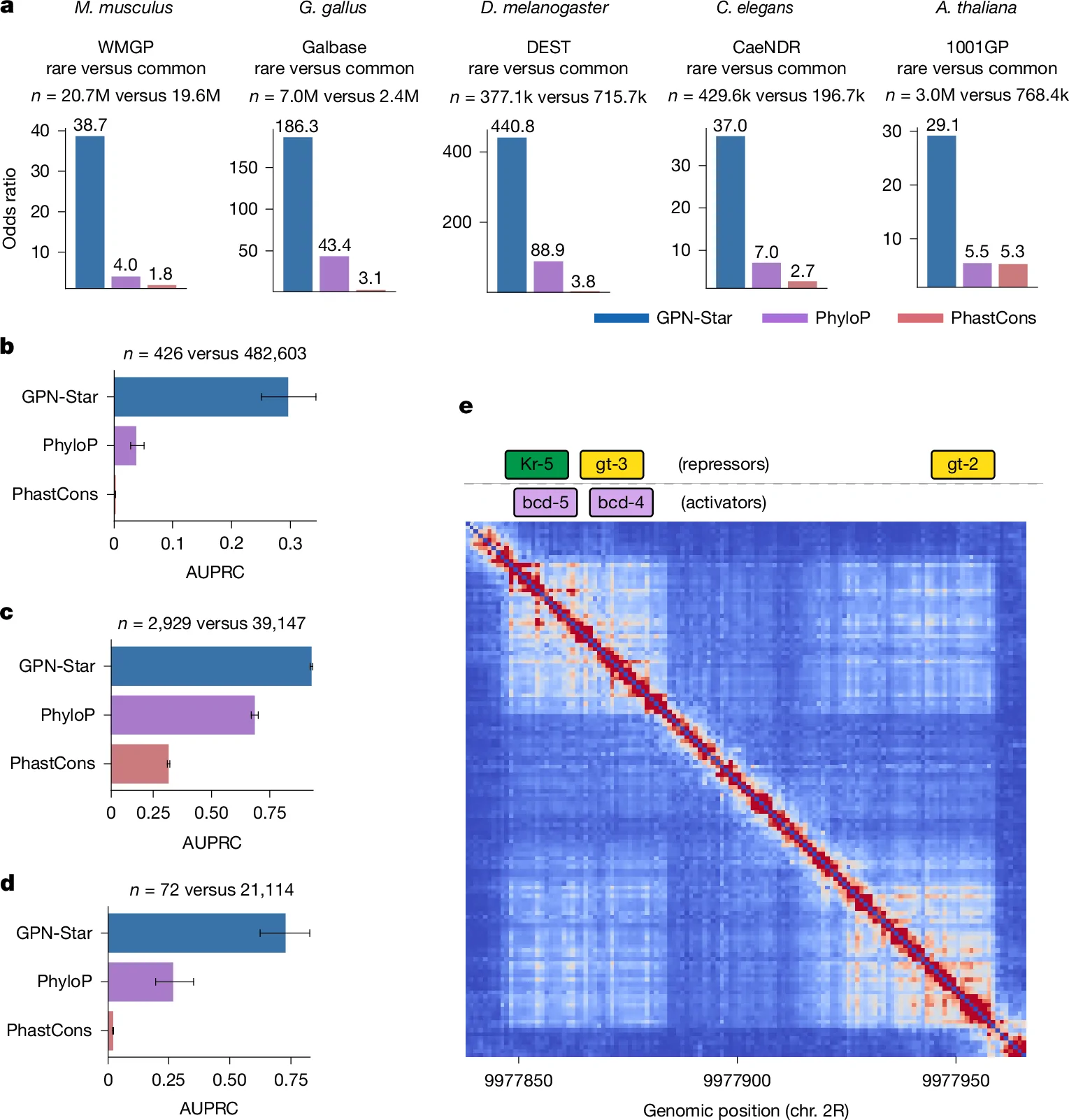
Application of GPN-Star to non-human species. **a**, Evaluation of GPN-Star models for five non-human species on enrichment of rare versus common variants (thresholds in Methods) in five corresponding population genome databases in the tail of deleterious scores (the threshold was chosen such that each score yielded 30 false discoveries), compared with PhyloP and PhastCons fitted to the same alignments. **b**, Classification of MMRdb pathogenic variants versus WMGP common variants in *M. musculus*. **c**, Classification of FlyBase lethal variants versus DEST common variants in *D. melanogaster*. **d**, Classification of *C. elegans* lethal variants versus CaeNDR common variants. The performance metric used in **b**–**d** is the AUPRC. The bar height is the metric computed on the complete benchmark set and the error bars represent the 95% confidence intervals from 1,000 class-stratified bootstrap resamples. **e**, Nucleotide dependency map of the *D. melanogaster* model at a locus in the MSE enhancer annotated with known TFBS.

Owing to the scarcity of large-scale curated variant datasets with functional annotations in non-human species, we used population genetic data for evaluation. Specifically, we gathered population-level variant data for each of the five species (Methods) and examined the enrichment of rare variants in the most deleterious tail of the prediction distributions. Across all five species, GPN-Star scores exhibited substantially higher rare variant enrichment compared with PhyloP and PhastCons (Fig. 5a), indicating more accurate predictions of genome-wide evolutionary constraints. Across different variant categories based on molecular consequences, GPN-Star consistently showed the highest enrichment in almost all cases (Supplementary Figs. 15b–f and 16). In *A. thaliana*, the performance gap between GPN-Star and other models was smaller compared with the results for the other species, which could be due to either the small alignment or the known lower quality of WGAs in plants^56^.

For three of the species, we were able to gather curated pathogenic variants to carry out further evaluation. For the *M. musculus* model, we used pathogenic variants from the MMRdb database (Methods). GPN-Star outperformed both PhyloP and PhastCons in distinguishing these pathogenic variants from common variants in the population, both genome-wide (Fig. 5b) and in each variant category (Supplementary Fig. 16). Similarly, we collected experimentally validated lethal variants for *D. melanogaster* (from FlyBase^57^) and for *C. elegans* (from an experimental study^58^) and found that GPN-Star consistently outperformed the other models on both datasets (Fig. 5c,d and Supplementary Fig. 16). We also evaluated the mouse output head of Enformer^35^ on the non-coding variants in the mouse benchmarks (Supplementary Fig. 17). It was markedly outperformed by GPN-Star, echoing the non-coding VEP results for humans.

Beyond variant prioritization, GPN-Star also enables exploration of learned functional elements and their co-evolution through nucleotide dependency analysis. As an example, we highlight learned nucleotide dependencies between TFBS at the well-characterized MSE enhancer locus in *D. melanogaster* (Fig. 5e). The coordinated activity of these transcription factors drives precise spatial patterning across the fly embryo^59^. This showcases how GPN-Star can serve as an efficient and low-cost tool for investigating functional elements and their dependencies across genomes, complementing and guiding experimental studies.

## Discussion

GPN-Star is a phylogeny-informed genomic language modelling framework that consistently outperforms existing methods for predicting functional constraint and deleterious variants across the human genome. Notably, GPN-Star achieved particularly strong predictive performance for distal enhancer variants, suggesting that evolutionary constraint information derived from cross-species comparisons can provide valuable signals for modelling this historically challenging class of regulatory element^60^. Beyond predictive accuracy, it learns biologically meaningful representations spanning functional elements from enhancers to TFBS and their co-evolutionary dependencies—all without any supervision. Classical WGA-based models, such as PhastCons^18^ and PhyloP^19^, have been indispensable tools to biologists and clinicians for over two decades. Our results indicate that GPN-Star complements and extends these tools, offering improved performance across diverse species and alignments.

A central insight from our study is that the evolutionary timescale represented in the training data strongly influences the constraint learned by gLMs (Figs. 1c and 2i), corroborating and extending previous observations for classical phylogenetic methods^7^. Models trained on deeper timescales better capture coding and other highly constrained regions, whereas shallower timescales better inform rapidly evolving regulatory elements, including those constrained on primate-specific regulation^8^. The evolutionary timescale should therefore be considered an important design consideration for future gLMs.

Like classical conservation scores, GPN-Star predictions quantify evolutionary constraint at specific timescales rather than general variant pathogenicity. Appropriate timescales should therefore be chosen according to the biological question. For integrating or selecting among models in predictive or discovery workflows, we recommend data-driven approaches that learn appropriate weights and transformations from task-specific data, as we demonstrated with DeepRVAT in our RVAT analysis.

GPN-Star achieves state-of-the-art performance in functional constraint prediction with substantially smaller model and context sizes compared with existing single-sequence gLMs. This effectiveness probably stems from the explicit homology information provided by the alignment, which is not readily available to single-sequence models. Although we observed modest performance gains from increased model sizes, improvements from expanding context sizes were relatively small (Supplementary Fig. 18). A plausible explanation is that WGAs are constructed relative to a reference species and often consist of small, highly fragmented synteny blocks. As a result, when context size increases, nucleotides in a window may not be contiguous in the actual genomes of non-reference species, introducing misleading context information to the model. Exploring how to leverage larger context sizes with WGA data is a promising direction for future research. Advancement in model architecture design or alignment data structure is probably necessary.

A practical limitation is that GPN-Star requires WGAs during inference. To facilitate its use, we have released genome-wide predictions through public repositories. Also, the reliance on a fixed alignment format makes the model less suitable for analysing indels and structural variants. On the other hand, single-sequence gLMs offer greater flexibility and applicability to a broad range of tasks and can naturally leverage large context and process indels and structural variants. We envision that future work can bridge these paradigms potentially through flexible homology retrieval mechanisms, allowing models to dynamically incorporate evolutionary information without relying on rigid alignment structures.

Our results on pathogenicity prediction and complex trait heritability constitute a systematic evaluation of GPN-Star in the context of present knowledge of human genetic variation. However, the true value of the model depends on its capacity to enable biological discovery. We expect GPN-Star to hold great potential in human genetics applications by advancing our understanding of causal variants and human traits. Initial experiments demonstrate improved RVAT, and we expect even greater gains for non-coding analyses, in which variant prioritization remains challenging. GPN-Star may also improve functionally informed fine-mapping, polygenic risk prediction and phenotype prediction.

Beyond identifying evolutionary constraints and deleterious variants, an important future direction is to study human-specific adaptations. The present GPN-Star models focus on capturing evolutionary signals across species, which lack the resolution for studying selection in human populations. We anticipate this to be an important direction for future work and would require learning from even more localized evolutionary data, such as human population genomes and archaic human genomes.

It is remarkable how much can be learned from unlabelled DNA sequences alone. Evolutionary and functional genomics provide complementary views of genetic variation. Although evolutionary modelling outperformed present sequence-to-function approaches in our pathogenicity and heritability analyses, functional genomics remains essential for studying molecular phenotypes and tissue-specific regulation. Integrating these complementary sources of information into multimodal gLMs represents a promising direction.

Large-scale efforts to sequence and align genomes across the tree of life are accelerating^21,61^. GPN-Star is well positioned to take advantage of this growth, as it scales readily to new alignments and may gain further capacity from increasingly diverse training data. In this age of rapidly expanding genomic data, we expect GPN-Star to be a valuable tool for leveraging these data to advance our understanding of genetic variation.

## Methods

Supplementary Information provides details of training data, model architecture, training setup, inference, mutation rate calibration, embedding analysis, nucleotide dependency analysis, evaluation benchmarks, heritability enrichment analysis and RVAT.

### Reporting summary

Further information on research design is available in the Nature Portfolio Reporting Summary linked to this article.

## Online content

Any methods, additional references, Nature Portfolio reporting summaries, source data, extended data, supplementary information, acknowledgements, peer review information; details of author contributions and competing interests; and statements of data and code availability are available at https://doi.org/10.1038/s41586-026-11005-5.

## Supplementary information

Supplementary Information Supplementary Notes, Methods, Tables 1–11, Figs. 1–27 and references.

Reporting Summary

Peer Review file

## Data Availability

The pretrained models, training datasets and benchmark datasets are available on Hugging Face (https://huggingface.co/collections/songlab/gpn-star-68c0c055acc2ee51d5c4f129). Precomputed GPN-Star predictions for all possible single nucleotide variants in the human genome and the five model organisms are available on Hugging Face (https://doi.org/10.57967/hf/9849). All data sources used in this study are pre-existing and publicly available; their versions, accessions and URLs are listed in Supplementary Information. No new experimental data were generated in this study that require deposition.

## Code availability

Code to train the model, run inference and reproduce the main analyses is available at GitHub (https://github.com/songlab-cal/gpn) and Zenodo (https://doi.org/10.5281/zenodo.21501177)^63^.

## References

[1] Benegas, G., Ye, C., Albors, C., Li, J. C. & Song, Y. S. Genomic language models: opportunities and challenges. *Trends Genet.* **41**, 286–302 (2025). 10.1016/j.tig.2024.11.013 39753409

[2] Benegas, G., Eraslan, G. & Song, Y. S. Benchmarking DNA sequence models for causal regulatory variant prediction in human genetics. Preprint at *bioRxiv* 10.1101/2025.02.11.637758 (2025).

[3] Dalla-Torre, H. et al. Nucleotide transformer: building and evaluating robust foundation models for human genomics. *Nat. Methods* **22**, 287–297 (2025). 10.1038/s41592-024-02523-z PMC11810778 39609566

[4] Brixi, G. et al. Genome modelling and design across all domains of life with Evo 2. *Nature* **652**, 1349–1361 (2026). 10.1038/s41586-026-10176-5 PMC13128491 41781614

[5] Clarke, B. et al. Integration of variant annotations using deep set networks boosts rare variant association testing. *Nat. Genet.* **56**, 2271–2280 (2024). 10.1038/s41588-024-01919-z PMC11525182 39322779

[6] Dayhoff, M. O., Schwartz, R. M. & Orcutt, B. C. A model of evolutionary change in proteins. *Atlas Protein Seq. Struct.* **5**, 345–352 (1978).

[7] Sullivan, P. F. et al. Leveraging base-pair mammalian constraint to understand genetic variation and human disease. *Science* **380**, eabn2937 (2023). 10.1126/science.abn2937 PMC10259825 37104612

[8] Kuderna, L. F. et al. Identification of constrained sequence elements across 239 primate genomes. *Nature* **625**, 735–742 (2024). 10.1038/s41586-023-06798-8 PMC10808062 38030727

[9] Benegas, G., Batra, S. S. & Song, Y. S. DNA language models are powerful predictors of genome-wide variant effects. *Proc. Natl Acad. Sci. USA* **120**, e2311219120 (2023). 10.1073/pnas.2311219120 PMC10622914 37883436

[10] Jumper, J. et al. Highly accurate protein structure prediction with AlphaFold. *Nature* **596**, 583–589 (2021). 10.1038/s41586-021-03819-2 PMC8371605 34265844

[11] Rao, R. M. et al. MSA Transformer. In *Proc. 38th Int. Conf. on Machine Learning* vol. 139 (eds Meila, M. & Zhang, T.) 8844–8856 (PMLR, 2021).

[12] Frazer, J. et al. Disease variant prediction with deep generative models of evolutionary data. *Nature* **599**, 91–95 (2021). 10.1038/s41586-021-04043-8 34707284

[13] Truong, T. Jr & Bepler, T. PoET: a generative model of protein families as sequences-of-sequences. *Adv. Neural Info. Process. Syst.* **36**, 77379–77415 (2023).

[14] Yang, K. K. et al. The Dayhoff Atlas: scaling sequence diversity for improved protein generation. Preprint at *bioRxiv* 10.1101/2025.07.21.665991 (2025).

[15] Akiyama, Y. et al. Expanding the scope of protein language modeling to protein-protein interactions with MSA pairformer. *Cell* **189**, 4964–4979 (2026). 10.1016/j.cell.2026.06.029 42480528

[16] Blanchette, M. et al. Aligning multiple genomic sequences with the threaded blockset aligner. *Genome Res.* **14**, 708–715 (2004). 10.1101/gr.1933104 PMC383317 15060014

[17] Armstrong, J. et al. Progressive Cactus is a multiple-genome aligner for the thousand-genome era. *Nature* **587**, 246–251 (2020). 10.1038/s41586-020-2871-y PMC7673649 33177663

[18] Siepel, A. et al. Evolutionarily conserved elements in vertebrate, insect, worm, and yeast genomes. *Genome Res.* **15**, 1034–1050 (2005). 10.1101/gr.3715005 PMC1182216 16024819

[19] Pollard, K. S., Hubisz, M. J., Rosenbloom, K. R. & Siepel, A. Detection of nonneutral substitution rates on mammalian phylogenies. *Genome Res.* **20**, 110–121 (2010). 10.1101/gr.097857.109 PMC2798823 19858363

[20] Christmas, M. J. et al. Evolutionary constraint and innovation across hundreds of placental mammals. *Science* **380**, eabn3943 (2023). 10.1126/science.abn3943 PMC10250106 37104599

[21] Rhie, A. et al. Towards complete and error-free genome assemblies of all vertebrate species. *Nature* **592**, 737–746 (2021). 10.1038/s41586-021-03451-0 PMC8081667 33911273

[22] Benegas, G., Albors, C., Aw, A. J., Ye, C. & Song, Y. S. A DNA language model based on multispecies alignment predicts the effects of genome-wide variants. *Nat. Biotechnol.* **43**, 1960–1965 (2025). 10.1038/s41587-024-02511-w 39747647

[23] Kim, A. et al. Identifying independent causal cell types for human diseases and risk variants. *Cell Genom.* 10.1016/j.xgen.2026.101325 (2026). PMC13576728 42600622

[24] Landrum, M. J. et al. ClinVar: public archive of relationships among sequence variation and human phenotype. *Nucleic Acids Res.* **42**, D980–D985 (2014). 10.1093/nar/gkt1113 PMC3965032 24234437

[25] Rentzsch, P., Witten, D., Cooper, G. M., Shendure, J. & Kircher, M. CADD: predicting the deleteriousness of variants throughout the human genome. *Nucleic Acids Res.* **47**, D886–D894 (2019). 10.1093/nar/gky1016 PMC6323892 30371827

[26] Rives, A. et al. Biological structure and function emerge from scaling unsupervised learning to 250 million protein sequences. *Proc. Natl Acad. Sci. USA* **118**, e2016239118 (2021). 10.1073/pnas.2016239118 PMC8053943 33876751

[27] Lin, Z. et al. Evolutionary-scale prediction of atomic-level protein structure with a language model. *Science* **379**, 1123–1130 (2023). 10.1126/science.ade2574 36927031

[28] Hayes, T. et al. Simulating 500 million years of evolution with a language model. *Science* **387**, 850858 (2025). 10.1126/science.ads0018 39818825

[29] Tate, J. G. et al. COSMIC: the catalogue of somatic mutations in cancer. *Nucleic Acids Res.* **47**, D941–D947 (2019). 10.1093/nar/gky1015 PMC6323903 30371878

[30] Chen, S. et al. A genomic mutational constraint map using variation in 76,156 human genomes. *Nature* **625**, 92–100 (2024). 10.1038/s41586-023-06045-0 PMC11629659 38057664

[31] Notin, P. et al. ProteinGym: large-scale benchmarks for protein fitness prediction and design. *Adv. Neural Info. Process. Syst.* **36**, 64331–64379 (2023).

[32] Cheng, J. et al. Accurate proteome-wide missense variant effect prediction with AlphaMissense. *Science* **381**, eadg7492 (2023). 10.1126/science.adg7492 37733863

[33] Gao, H. et al. The landscape of tolerated genetic variation in humans and primates. *Science* **380**, eabn8153 (2023). 10.1126/science.abn8197 PMC10713091 37262156

[34] Ghosh, R. et al. Updated recommendation for the benign stand-alone ACMG/AMP criterion. *Hum. Mutat.* **39**, 1525–1530 (2018). 10.1002/humu.23642 PMC6188666 30311383

[35] Avsec, Ž et al. Effective gene expression prediction from sequence by integrating long-range interactions. *Nat. Methods* **18**, 1196–1203 (2021). 10.1038/s41592-021-01252-x PMC8490152 34608324

[36] Linder, J., Srivastava, D., Yuan, H., Agarwal, V. & Kelley, D. R. Predicting RNA-seq coverage from DNA sequence as a unifying model of gene regulation. *Nat. Genet.* **57**, 949–961 (2025). 10.1038/s41588-024-02053-6 PMC11985352 39779956

[37] Avsec, Ž et al. Advancing regulatory variant effect prediction with AlphaGenome. *Nature* **649**, 1206–1218 (2026). 10.1038/s41586-025-10014-0 PMC12851941 41606153

[38] Amberger, J. S., Bocchini, C. A., Schiettecatte, F., Scott, A. F. & Hamosh, A. OMIM.org: Online Mendelian Inheritance in Man (OMIM), an online catalog of human genes and genetic disorders. *Nucleic Acids Res.* **43**, D789–D798 (2015). 10.1093/nar/gku1205 PMC4383985 25428349

[39] Stenson, P. D. et al. The Human Gene Mutation Database (HGMD): optimizing its use in a clinical diagnostic or research setting. *Hum. Genet.* **139**, 1197–1207 (2020). 10.1007/s00439-020-02199-3 PMC7497289 32596782

[40] Jaganathan, K. et al. Predicting expression-altering promoter mutations with deep learning. *Science* **389**, eads7373 (2025). 10.1126/science.ads7373 40440429

[41] Tomaz da Silva, P. et al. Nucleotide dependency analysis of genomic language models detects functional elements. *Nat. Genet.* **57**, 2589–2602 (2025). 10.1038/s41588-025-02347-3 PMC12513839 41073788

[42] Kanai, M. et al. Insights from complex trait fine-mapping across diverse populations. Preprint at *medRxiv* 10.1101/2021.09.03.21262975 (2021).

[43] Bomba, L., Walter, K. & Soranzo, N. The impact of rare and low-frequency genetic variants in common disease. *Genome Biol.* **18**, 77 (2017). 10.1186/s13059-017-1212-4 PMC5408830 28449691

[44] Lee, S., Abecasis, G. R., Boehnke, M. & Lin, X. Rare-variant association analysis: study designs and statistical tests. *Am. J. Hum. Genet.* **95**, 5–23 (2014). 10.1016/j.ajhg.2014.06.009 PMC4085641 24995866

[45] Backman, J. D. et al. Exome sequencing and analysis of 454,787 UK Biobank participants. *Nature* **599**, 628–634 (2021). 10.1038/s41586-021-04103-z PMC8596853 34662886

[46] Karczewski, K. J. et al. Systematic single-variant and gene-based association testing of thousands of phenotypes in 394,841 UK Biobank exomes. *Cell Genom.* **2**, 100168 (2022). 10.1016/j.xgen.2022.100168 PMC9903662 36778668

[47] Zhou, J. & Troyanskaya, O. G. Predicting effects of noncoding variants with deep learning–based sequence model. *Nat. Methods* **12**, 931–934 (2015). 10.1038/nmeth.3547 PMC4768299 26301843

[48] Finucane, H. K. et al. Partitioning heritability by functional annotation using genome-wide association summary statistics. *Nat. Genet.* **47**, 1228–1235 (2015). 10.1038/ng.3404 PMC4626285 26414678

[49] Weissbrod, O. et al. Functionally informed fine-mapping and polygenic localization of complex trait heritability. *Nat. Genet.* **52**, 1355–1363 (2020). 10.1038/s41588-020-00735-5 PMC7710571 33199916

[50] Márquez-Luna, C. et al. Incorporating functional priors improves polygenic prediction accuracy in UK Biobank and 23andMe data sets. *Nat. Commun.* **12**, 6052 (2021). 10.1038/s41467-021-25171-9 PMC8523709 34663819

[51] O’Connor, L. J. & Sella, G. Principled measures and estimates of trait polygenicity. Preprint at *bioRxiv* 10.1101/2025.07.10.664154 (2025). PMC13231060 42105749

[52] Karollus, A., Mauermeier, T. & Gagneur, J. Current sequence-based models capture gene expression determinants in promoters but mostly ignore distal enhancers. *Genome Biol.* **24**, 56 (2023). 10.1186/s13059-023-02899-9 PMC10045630 36973806

[53] Fabiha, T. et al. A consensus variant-to-function score to functionally prioritize variants for disease. Preprint at *bioRxiv* 10.1101/2024.11.07.622307 (2024).

[54] Finucane, H. K. et al. Heritability enrichment of specifically expressed genes identifies disease-relevant tissues and cell types. *Nat. Genet.* **50**, 621–629 (2018). 10.1038/s41588-018-0081-4 PMC5896795 29632380

[55] Zhang, Z. et al. Protein language models learn evolutionary statistics of interacting sequence motifs. *Proc. Natl Acad. Sci. USA* **121**, e2406285121 (2024). 10.1073/pnas.2406285121 PMC11551344 39467119

[56] Song, B., Buckler, E. S. & Stitzer, M. C. New whole-genome alignment tools are needed for tapping into plant diversity. *Trends Plant Sci.* **29**, 355–369 (2024). 10.1016/j.tplants.2023.08.013 37749022

[57] Öztürk-Çolak, A. et al. FlyBase: updates to the *Drosophila* genes and genomes database. *Genetics* **227**, iyad211 (2024). 10.1093/genetics/iyad211 PMC11075543 38301657

[58] Qin, Z. et al. Genomic identification and functional characterization of essential genes in *Caenorhabditis elegans*. *Genes Genomes Genet.* **8**, 981–997 (2018). 10.1534/g3.117.300338 PMC5844317 29339407

[59] Small, S., Blair, A. & Levine, M. Regulation of even-skipped stripe 2 in the *Drosophila* embryo. *EMBO J.* **11**, 4047–4057 (1992). 10.1002/j.1460-2075.1992.tb05498.x PMC556915 1327756

[60] Wong, E. S. et al. Deep conservation of the enhancer regulatory code in animals. *Science* **370**, eaax8137 (2020). 10.1126/science.aax8137 33154111

[61] Lewin, H. A. et al. Earth BioGenome project: sequencing life for the future of life. *Proc. Natl Acad. Sci. USA* **115**, 4325–4333 (2018). 10.1073/pnas.1720115115 PMC5924910 29686065

[62] Seplyarskiy, V. et al. A mutation rate model at the basepair resolution identifies the mutagenic effect of polymerase III transcription. *Nat. Genet.* **55**, 2235–2242 (2023). 10.1038/s41588-023-01562-0 PMC11348951 38036792

[63] Ye, C., Benegas, G., Albors, C., Li, J. C. & Song, Y. S. GPN-Star model source code. *Zenodo*10.5281/zenodo.21501177 (2026).

[64] Verbeek, M. M. et al. Mutations in the cyclic adenosine monophosphate response element of the tyrosine hydroxylase gene. *Ann. Neurol.* **62**, 422–426 (2007). 10.1002/ana.21199 17696123

[65] Kircher, M. et al. Saturation mutagenesis of twenty disease-associated regulatory elements at single base-pair resolution. *Nat. Commun.* **10**, 3583 (2019). 10.1038/s41467-019-11526-w PMC6687891 31395865

[66] Bennett, M. K., Ngo, T. T., Athanikar, J. N., Rosenfeld, J. M. & Osborne, T. F. Co-stimulation of promoter for low density lipoprotein receptor gene by sterol regulatory element-binding protein and Sp1 is specifically disrupted by the yin yang 1 protein. *J. Biol. Chem.* **274**, 13025–13032 (1999). 10.1074/jbc.274.19.13025 10224053

