# Supplementary material for: Predicting genome-wide functional constraints with GPN-Star
Source: Nature. 2026 Sep 9;659(8139):141–50. doi: 10.1038/s41586-026-11005-5 (PMC13736532; doi:10.1038/s41586-026-11005-5)
Supplement: Supplementary file 1 [file 41586_2026_11005_MOESM1_ESM.pdf]

nature portfolio

https://doi.org/10.1038/s41586-026-11005-5

Supplementary information

Predicting genome-wide functional constraints with GPN-Star

In the format provided by the authors and unedited

Supplementary Text

Key differences between GPN-MSA and GPN-Star

GPN-MSA [22] is a transformer model trained on a WGA of 90 vertebrate species. GPN-MSA achieves strong performance across several variant effect prediction tasks. However, it uses a generic architecture more suitable for unaligned sequences, which limits its ability to fully exploit the rich structure inherent in alignment data, particularly the evolutionary relationship between species. Furthermore, conditioning on species too closely related to humans was empirically shown to deteriorate performance in variant effect prediction, possibly because the model's learned probability distribution becomes overly biased toward their genomes. This prompted us to exclude most primates from the training data – a suboptimal decision, since primates offer valuable insight into recent evolutionary constraints relevant to humans. Lastly, GPN-MSA is highly tuned for a particular alignment, which limits its adaptability to new data.

The GPN-Star framework advances beyond GPN-MSA in three key aspects: First, whereas GPN-MSA masks only the human genome during training, GPN-Star leverages all genomes in the alignment by predicting masked nucleotides across multiple species, thereby substantially increasing both the amount and diversity of training data. Second, GPN-Star explicitly incorporates phylogenetic relationships among species through specialized attention modules, enabling more accurate and biologically grounded modeling. Third, it flexibly accommodates alignments of arbitrary composition and size, eliminating the need for manual curation, such as excluding closely related species, as was required in GPN-MSA [22]. Empirically, we observed noticeable performance gains from each newly introduced architectural component, as well as consistent, substantial improvements over GPN-MSA across extensive evaluations (Supplementary Figure 19, Supplementary Figure 20, Supplementary Table 3).

Relevant evolutionary timescales for different variant effect prediction tasks and the genetic architecture of complex traits

Our analysis underscores the importance of the timescale represented in the training data of evolutionary models. Across VEP benchmarks, we observed a consistent pattern: Coding variants and lower-frequency variants with larger effect sizes tended to be better predicted by models trained on deeper evolutionary timescales. Such variants are enriched in the ClinVar, COSMIC, and ProteinGym benchmarks. In contrast, non-coding variants and higher-frequency variants with smaller effect sizes were, in general, more accurately predicted by models trained on shallower evolutionary timescales, as seen in the OMIM, HGMD, and fine-mapped GWAS benchmarks. Similar timescale-dependent performance patterns have been reported in a previous study [5] with PhyloP and PhastCons. We confirm that this dependency is not unique to classical phylogenetic models but also governs the performance of gLMs across a broader range of predictive tasks. Importantly, GPN-Star outperforms both classical models at each timescale consistently (Figure 2I).

We also analyzed how GPN-Star models trained on different evolutionary timescales prioritize variants in our complex trait heritability analysis. Among common variants prioritized by GPN-Star (P), 39% are shared with GPN-Star (M) and (V). Additionally, 59% are shared with GPN-Star (M), and 36.6% are unique to GPN-Star (P) (Supplementary Figure 21, see Supplementary Figure 22 for all S-LDSC variants). Motivated by the observation that GPN-Star (M) performs better on Mendelian traits while GPN-Star (P) excels on polygenic traits (Figure 1C, Figure 2I), we inspected their relative heritability enrichment performance against a recently estimated spectrum of trait polygenicity [63]. Across 27 traits with available polygenicity estimates, we found a moderate correlation (Pearson's $r = 0.47$) between the (log) effective polygenicity and the

heritability enrichment difference between GPN-Star (P) and GPN-Star (M) (Figure 3E, Extended Data Figure 3). For example, GPN-Star (M) attains a greater enrichment for LDL cholesterol (effective polygenicity = 89), while GPN-Star (P) attains a higher enrichment for schizophrenia (effective polygenicity = 13,069). This suggests a meaningful connection between evolutionary timescale and the genetic architecture of traits, and strengthens the evidence for the special role of primate-specific evolutionary constraint acting on human complex trait variation.

To better understand the kinds of variants prioritized by the top model GPN-Star (P), we analyzed Ensembl variant consequences [67] and ENCODE SCREEN candidate cis-regulatory elements (cCREs, Supplementary Table 4) [68]. Results for common variants are summarized in Figure 3F, focusing on consequence classes each comprising $> 1\%$ of prioritized variants. Full results for common variants and results including low-frequency variants are in Supplementary Tables 5-6 and Supplementary Figures 23 and **??**24. Missense variants represent the majority (33%, 170-fold enriched), followed by variants in regions with distal enhancer-like signatures (dELS) (26%, 2.4-fold enriched) and their flanks (8%, 0.35-fold depleted).

There are interesting differences in the variant types prioritized by the three GPN-Star models. There are only slight differences between GPN-Star (P) and (M), with the former enriched 1.14-fold in missense variants (Supplementary Table 7). Not surprisingly, there are far more differences between GPN-Star (P) and (V) (Supplementary Table 8). GPN-Star (P) is generally enriched in exonic variants, such as missense (1.52-fold) and even synonymous (3.89-fold), while it is depleted in non-exonic variants such as intergenic.

Visualization and analysis of GPN-Star embeddings

Genomics language models can learn powerful sequence representations by predicting masked nucleotides, as local nucleotide distributions in a region are heavily influenced by the region's function. While previous work has shown that gLM embeddings can distinguish genomic regions without supervision [9], it has remained unclear whether alignment-based models could do the same. To investigate this, we conducted a model interpretation analysis of GPN-Star by visualizing embeddings of genomic windows from gene regions, cCREs, and background regions. The resulting embeddings largely segregate by genomic region (Supplementary Figure 7A, Supplementary Figure 8). For example, coding sequences (CDS) and enhancers form distinct clusters. On the other hand, promoter and 5' UTR windows are difficult to distinguish, perhaps because 5' UTR embeddings encode a mix of transcriptional and post-transcriptional signals. Embeddings for conserved windows show stronger clustering by functional region compared to those for non-conserved windows (Supplementary Figure 7B). To quantitatively assess GPN-Star's capacity to distinguish genomic regions, we trained logistic regression classifiers using the model embeddings. The resulting classification accuracy patterns are broadly consistent with the visualization. CDS, enhancers, 3' UTRs, and promoters are classified with good accuracy, whereas 5' UTRs and lncRNAs are relatively more challenging to classify (Supplementary Figure 9). Restricting the analysis to conserved windows led to overall improvements across all classes.

Controlling for mutation rate variation improves constraint prediction

We studied the impact of mutation rate calibration on evolutionary constraint prediction. Before calibration, the raw model scores showed moderate correlations with mutation rate estimates from Roulette [61] (Spearman's $\rho = 0.31$-0.34 across the models for chromosome 22), indicating that the model captures signals from both selective constraints and mutational biases. After calibration, the correlations with mutation rate estimates were reduced to negligible levels, lower than those

observed for PhyloP and PhastCons at the same evolutionary timescales, and improved performance across most downstream benchmarks (Figure 4D, Extended Data Figure 10).

We further validated our calibration procedure by comparing our GPN-Star scores to Gnocchi, a genome-wide constraint score estimated from gnomAD v3 that explicitly accounts for mutation rate variation. Calibrated GPN-Star scores showed higher concordance with Gnocchi than either PhyloP or PhastCons (Figure 4D). These results demonstrate that our calibration procedure effectively removes mutation rate biases in model predictions, thereby better reflecting evolutionary constraints.

As a final sanity check, we confirmed that Roulette mutation rates themselves exhibit negligible enrichment of singletons in their low-rate tails (Figure 4B) and near-baseline performance on pathogenicity prediction tasks (Extended Data Figure 1). This confirms that our rare variant enrichment and pathogenicity benchmarks primarily assess selective constraint rather than mutational bias.

These findings highlight the critical importance of addressing mutation rate variation in future development of gLMs, particularly for applications involving functional constraint prediction.

Supplementary Methods

Training Data

General workflow. The current training workflow requires:

- A multispecies whole-genome alignment (e.g., obtained with MULTIZ [16] or Cactus [17]).
- A phylogenetic tree.
- PhastCons [18] and PhyloP [19] conservation scores.

MULTIZ alignments (MAF format) were first transformed into FASTA format using `maf2fasta` [16]. As in GPN-MSA [22], we remove columns with gaps in the target sequence. Finally, we transform the alignment into Zarr format [69], which allows fast multi-threaded random access to genomic windows.

Similar to Benegas et al. [22], we further select training regions from the target genomes based on conservation. For every model, a sliding window equal to the model's context length is moved across unmasked genomic regions with a stride of half the window size. We define the conservation of a window as the 75th percentile of PhastCons scores in the window. We retain the top 5% most conserved windows and a random 0.1% of the remaining windows as the training data for the model. The windows are split by chromosome into training and validation sets as detailed in Supplementary Table 1. For the selected windows, sequences on both the forward and reverse strands are included in the training data.

Data sources. MULTIZ alignments and associated data are readily available at the UCSC Genome Browser [70] for many of the species covered in this paper (Supplementary Table 9). The human alignments with primates and mammals [6, 71] were done with Cactus [17] and processed by Albors et al. [72]. The MULTIZ alignment for *A. thaliana* (`TAIR10` reference) and 17 other Brassicales, as well as conservation scores, was downloaded from PlantRegMap [73].

Additional phylogenetic tree construction step for Brassicales alignment. Since no phylogenetic tree was publicly available for the species in the Brassicales alignment, we generated one with the PHAST package [74] based on fourfold degenerate sites, as described in the original publication [73]. We used the Ensembl [67] release 60 annotation. Since `msa_view`, part of the pipeline, only works with one chromosome at a time, we picked chromosome 1 arbitrarily.

```
msa_view ../maf/tair10_multiz18way/1.maf --4d --features chr1.gff > 4d-codons.ss
msa_view 4d-codons.ss --in-format SS --out-format SS --tuple-size 1 > 4d-sites.ss
phyloFit --tree "(Carica_papaya,(Tarenaya_hassleriana,(Aethionema_arabicum,
((Arabis_alpina,(Eutrema_salsugineum,(Thellungiella_parvula,(Sisymbrium_irio,
(((Brassica_oleracea,Brassica_napus),Brassica_rapa),Raphanus_sativus))))),
((Boechera_stricta,(Camelina_sativa,(Capsella_grandiflora,Capsella_rubella))),
(Arabidopsis_thaliana,(Arabidopsis_halleri,Arabidopsis_lyrata)))))))"
--msa-format SS --out-root nonconserved-4d --EM --precision MED 4d-sites.ss
```

Model Architecture

The GPN-Star model employs a novel transformer-based architecture operating on blocks of multispecies whole-genome alignments. We denote an input alignment block as $\mathbf{X} \in \mathbb{R}^{N_{\mathcal{S}} \times L \times V}$, where $N_{\mathcal{S}}$ is the number of species and L is the length of the alignment block, and V is the size of the nucleotide vocabulary. Each entry, or token, denoted as $\boldsymbol{x}_j^{(i)}$, is represented as one-hot encoding the nucleotide identity of the sequence of the ith species at position j.

Target and source sequences. The primary objective of GPN-Star is to learn a conditional distribution of nucleotides that models the evolutionary processes between sequences from different species in the alignment. Specifically, for each token in a sequence on which we want to predict the distribution, denoted as a target sequence, GPN-Star learns:

$$\mathbb{P}(\boldsymbol{x}_j^{(i)} \mid \boldsymbol{x}_{-j}^{(i)}, \{\boldsymbol{x}^{(i')}\}_{i' \in \mathcal{S}}, \{\phi_{ii'}\}_{i' \in \mathcal{S}}),$$

where $\boldsymbol{x}_j^{(i)}$ is the token at position j in the target sequence of the species i, $\boldsymbol{x}_{-j}^{(i)}$ denotes the remaining tokens in the sequence i, $\{\boldsymbol{x}^{(i')}\}_{i' \in \mathcal{S}}$ are sequences from a set of species $\mathcal{S}$ that provides evolutionary context, which we call source sequences, and $\{\phi_{ii'}\}_{i' \in \mathcal{S}}$ are the evolutionary distances between the target sequence and each source sequence.

Therefore, as the first step in GPN-Star, we compile two distinct sets of sequences from each alignment block:

- Target sequences, $\{\boldsymbol{x}^{(t)}\}_{t \in \mathcal{T}}$, where $\boldsymbol{x}^{(t)} \in \mathbb{R}^{L \times V}, |\mathcal{T}| = N_{\mathcal{T}}$: sequences used for loss calculation and inference. In training and inference, a subset of $N_{\mathcal{T}}$ species is chosen. The first target species is designated as the primary target species, denoted as t_0, which the whole genome alignment is stitched according to and thus provides contiguous genomic coordinates to anchor positional information. It is also the primary species for making inferences with the trained model.

- Source sequences, $\{\boldsymbol{x}^{(s)}\}_{s \in \mathcal{S}}$, where $\boldsymbol{x}^{(s)} \in \mathbb{R}^{L \times V}, |\mathcal{S}| = N_{\mathcal{S}}$: sequences of all species in the alignment. They provide the evolutionary context for inferring the distribution of the target sequences.

Grouping species into clades. To efficiently represent phylogenetic relationships among species, we group them into discrete clades based on pairwise phylogenetic distances. Concretely, species are partitioned into clades such that any two species belonging to distinct clades have a pairwise phylogenetic distance greater than a predefined model-specific threshold ϕ_{clade}, which is effectively single-linkage clustering. This criterion ensures in-clade phylogenetic coherence while ensuring species in distinct clades are sufficiently distant evolutionarily. The number of clades is denoted by $N_{\mathcal{C}}$.

Embedding target and source sequences. For target sequences, each input token is directly embedded into H-dimension vectors:

$$\boldsymbol{e}_j^{(t)} = f_e(\boldsymbol{x}_j^{(t)}),$$

where $f_e : \mathbb{R}^V \to \mathbb{R}^H$ is a learned embedding function. $\boldsymbol{x}_j^{(t)}$ is the one-hot encoded nucleotide at position j of the target sequence t. This results in target sequence embeddings $\{\boldsymbol{E}^{(t)}\}_{t\in\mathcal{T}}$, where $\boldsymbol{E}^{(t)} \in \mathbb{R}^{L\times H}$.

For embedding source sequences, the sequences of species within each clade are condensed into a single representative embedding per alignment column via an attention pooling module. For each clade c, tokens from member species at column j are combined in each attention head as follows:

$$\text{Attn\_pool}(\boldsymbol{x}_j^{(s)}, s \in c) = \sum_{s\in c} \text{softmax}\left(\boldsymbol{x}_j^{(s)}\boldsymbol{W}_\alpha \, + b(\phi_{c,s})\right) \boldsymbol{x}_j^{(s)}\, \boldsymbol{W}_v,$$

where $\boldsymbol{W}_\alpha \in \mathbb{R}^{V\times 1}$, $\boldsymbol{W}_v \in \mathbb{R}^{V\times D}$ are learnable weight matrices and $b : \mathbb{R} \to \mathbb{R}$ is the evolutionary distance encoding function (see the section Phylogeny-informed cross-attention for detailed description), and $\phi_{c,s}$ is the phylogenetic distance between species s and the Most Recent Common Ancestor (MRCA) of all species in clade c. $\boldsymbol{x}_j^{(s)}$ is the one-hot encoded nucleotide at position j of the source sequence s. The output embeddings across A attention heads are concatenated and then transformed to an H-dimension embedding by a linear layer, which is the final output of the module. This attention pooling procedure aggregates evolutionary signals while reducing computational complexity downstream. We also empirically observed this clade-pooling strategy to improve downstream variant effect prediction performance through an ablation study (Supplementary Figure 19, Supplementary Table 3). If there is only a single species in a clade, the attention pooling module is reduced to a single embedding module, similar to that for the target sequences. This results in clade-level source sequence embeddings $\{\boldsymbol{E}^{(c)}\}_{c\in\mathcal{C}}$, where $\boldsymbol{E}^{(c)} \in \mathbb{R}^{L\times H}$.

The GPN-Star encoder. GPN-Star uses an encoder-only architecture, and the encoder consists of a stack of K encoder blocks. Each block sequentially comprises:

1. A sequence-wise self-attention module.
2. A phylogeny-informed cross-attention module.
3. A feed-forward network.

Sequence embeddings are passed through the encoder blocks sequentially. The first and second attention modules each have A attention heads. Standard residual connections are applied in each module, and the embeddings are normalized with LayerNorm before each module, with dropout (probability 0.1) applied throughout. The third module is the same as in standard transformers. It is a two-layer fully-connected network that accepts the concatenated embeddings from the A

attention heads and outputs an embedding of dimension H, with an intermediate layer of dimension F and the GELU activation. Here, we describe the first two specialized attention modules in further detail.

Sequence-wise self-attention. This module captures the local nucleotide context along the genome and is applied to each of the target sequences. Due to extensive rearrangements and fragmentation among the genomes of the species in the alignments, positional context is most reliably defined in the primary target species. Therefore, queries and keys in the self-attention are calculated exclusively from the embeddings of the primary target species, then the attention score is applied to all the value tensors from the embeddings of all target species. For each target sequence embedding, the output of one attention head is:

$$\mathrm{Attn}_{\mathrm{seq}}(\boldsymbol{E}^{(t)}) = \mathrm{softmax}\left(\frac{(\boldsymbol{E}^{(t_0)}\boldsymbol{W}_Q^{\mathrm{seq}})(\boldsymbol{E}^{(t_0)}\boldsymbol{W}_K^{\mathrm{seq}})^\top}{\sqrt{D}}\right)\boldsymbol{E}^{(t)}\boldsymbol{W}_V^{\mathrm{seq}},$$

where $\boldsymbol{W}_Q^{\mathrm{seq}}, \boldsymbol{W}_K^{\mathrm{seq}}, \boldsymbol{W}_V^{\mathrm{seq}} \in \mathbb{R}^{H\times D}$ are learnable key, query and value weight matrices of the attention head, $\boldsymbol{E}^{(t)} \in \mathbb{R}^{L\times H}$ is the sequence embedding of the target species t from the previous layer, and $\boldsymbol{E}^{(t_0)} \in \mathbb{R}^{L\times H}$ is the sequence embedding of the primary target species t_0 from the previous layer. We employed the Rotary Positional Encoding (RoPE) [75] in the attention module to embed genomic positions with respect to the primary target species genome, which is omitted from the above formula for ease of description. As an additional benefit, sharing a single attention map across the $N_\mathcal{T}$ sequences significantly reduces memory footprint from $O(N_\mathcal{T}L^2)$ to $O(L^2)$, similar to another approach introduced in MSA Transformer [11].

Phylogeny-informed cross-attention. This module explicitly models evolutionary processes from the source sequences to the target sequences, while integrating phylogenetic information directly into attention calculations. Cross-attention is computed between target sequence embeddings (used to compute query tensors) and pooled source clade embeddings (used to compute the key and value tensors). The output of one attention head is:

$$\mathrm{Attn}_{\mathrm{phy}}(\boldsymbol{E}_j^{(\mathcal{T})}, \boldsymbol{E}_j^{(\mathcal{C})}) = \mathrm{softmax}\left(\frac{(\boldsymbol{E}_j^{(\mathcal{T})}\boldsymbol{W}_Q^{\mathrm{phy}})(\boldsymbol{E}_j^{(\mathcal{C})}\boldsymbol{W}_K^{\mathrm{phy}})^\top}{\sqrt{D}} + b(\Phi)\right)\boldsymbol{E}_j^{(\mathcal{C})}\boldsymbol{W}_V^{\mathrm{phy}},$$

where $\boldsymbol{W}_Q^{\mathrm{phy}}, \boldsymbol{W}_K^{\mathrm{phy}}, \boldsymbol{W}_V^{\mathrm{phy}} \in \mathbb{R}^{H\times D}$ are learnable key, query and value weight matrices of the attention head, $\boldsymbol{E}_j^{(\mathcal{T})} \in \mathbb{R}^{N_\mathcal{T}\times H}$ are target embeddings at the jth alignment column from the previous module, $\boldsymbol{E}_j^{(\mathcal{C})} \in \mathbb{R}^{N_\mathcal{C}\times H}$ are the clade-level source embeddings at the jth column, and $\Phi \in \mathbb{R}^{N_\mathcal{T}\times N_\mathcal{C}}$ are the average phylogenetic distances between each target species and the source species in each clade. In order to encode the notion of evolutionary distance between each target and source species, we replaced the positional encoding in conventional attention modules with an evolutionary distance encoding, which is the bias term $b(\Phi)$ in the above formula. We adapted the Functional Interpolation for Relative Positional Encoding (FIRE) method [76] for encoding evolutionary distances:

$$b(\phi) = f_\theta\left(\frac{g(\phi)}{g(\phi_{\max})}\right),$$

where $g : x \mapsto \log(cx+1)$ and $c \in \mathbb{R}^+$ is a learned scaling factor, $\phi_{\max}$ is the maximum distance among all species pairs in the alignment, and $f_\theta : \mathbb{R} \to \mathbb{R}$ is a two-layer perceptron $f_\theta(x) =$

$\boldsymbol{v}_2^\top \sigma(\boldsymbol{v}_1 x)$, where $\theta = \{\boldsymbol{v}_1, \boldsymbol{v}_2 \in \mathbb{R}^R\}$ are learnable parameters and σ is the SiLU activation function. This evolutionary distance encoding module is shared across attention heads in the same layer. To assess the contribution of the evolutionary distance encoding $b(\Phi)$, we performed an ablation study in which it was removed from the attention calculation, and observed consistent performance drops across all variant effect prediction benchmarks (Supplementary Figure 19, Supplementary Table 3).

There are two main reasons for the choice of this distance encoding scheme: first, unlike distances between positions on a sequence, the phylogenetic distances between species follow tree branching paths and are not additive along a single line. Therefore, the relative positional encoding scheme offers much convenience by simply adding bias terms to the attention score based on the pairwise distances; second, we would like the attention scores to have flexible dependencies on the evolutionary distances between species and the adapted FIRE method achieves this purpose by using a two-layer perceptron to learn a flexible mapping from the original evolutionary distance to the bias term in the attention calculation.

As mentioned, species within the same clade are evolutionarily close and have very high sequence similarity. Therefore, for the model to learn from meaningful evolutionary processes, we apply in-clade attention masks in this cross-attention module to prevent tokens in the target species from attending to the clade embeddings that the target species belongs to.

Output layer. Following the stack of K GPN-Star encoder blocks, the output layer is a standard masked language modeling head to convert hidden embeddings into per-token nucleotide probabilities:

$$\hat{p}(x_j^{(t)} \mid \boldsymbol{E}_j^{(t)}) = \mathrm{softmax}\big(\boldsymbol{W}_o \boldsymbol{E}_j^{(t)} + \boldsymbol{b}_o\big),$$

where $\boldsymbol{W}_o \in \mathbb{R}^{V \times H}$ and $b_o \in \mathbb{R}^V$ are the learnable weight matrix and bias term in the module, and $\boldsymbol{E}_j^{(t)}$ is the embedding from the final encoder block for species t at position j. The predicted nucleotide probabilities are then used to compute training loss or make inferences.

Training Setup

The model is trained on a masked language modeling objective. For each training sample, we construct the target sequences from the alignment block as follows. The primary target species will be included in every sample. Then we select 19 target species by stratified random sampling to account for phylogenetic bias: first, we sample 19 clades out of all the clades, with replacement if the number of clades is fewer than 19 and without replacement otherwise; second, we sample one species from each selected clade. This results in 20 target species per training sample. To assess the benefit of multispecies training, we performed an ablation study comparing training on multiple target species versus a single species, and observed consistent performance gains across a range of benchmarks (Supplementary Figure 19, Supplementary Table 3). As for source sequences, all species will be used as aforementioned.

We next apply masking to the input sequences. In each training sample, we randomly select 15% of the non-gap positions in each target sequence, ensuring that the same positions are selected in species that belong to the same clade. We denote the set of indices of the selected tokens as $\mathcal{M}$. Then, randomly, 90% of the selected tokens are replaced with a `[MASK]` token and the remaining 10% are left as is, resulting in a set of masked target sequences $\{\tilde{\boldsymbol{x}}^{(t)}\}_{t \in \mathcal{T}}$. The source sequences are also masked in a way that all sequences from the same clade are masked at the same positions between the target and source sequences. The masked source sequences are denoted as $\{\tilde{\boldsymbol{x}}^{(s)}\}_{s \in \mathcal{S}}$.

The masked sequences are used as input. And the model is trained with a weighted cross-entropy loss on the selected tokens of original target sequences after data augmentation $\acute{x}_j^{(t)}$ (detailed below):

$$\mathcal{L}_{\text{MLM}} = -\frac{1}{|\mathcal{M}|} \sum_{(t,j)\in\mathcal{M}} w_j \log \hat{p}\big(\acute{x}_j^{(t)} | \{\tilde{\boldsymbol{x}}^{(t)}\}_{t\in\mathcal{T}}, \{\tilde{\boldsymbol{x}}^{(s)}\}_{s\in\mathcal{S}}\big),$$

The same loss weighting scheme as Benegas *et al.* is applied [22]. The purpose is to downweight repeats and upweight conserved elements so that incorrect predictions at neutral or non-functional regions are penalized less during training. Repetitive positions were extracted from lowercase letters in the soft-masked Ensembl genome [67], processed with RepeatMasker [77] and Dust [78]. PhyloP and PhastCons are used as conservation metrics to calculate the weights. For PhastCons, we create a smoothed version, PhastCons_M, that takes the maximum PhastCons score in the local 7-bp window. The loss weight of a position is defined as:

$$w \propto (0.1 \times \mathbb{1}\{\text{repeat}\} + \mathbb{1}\{\neg\text{repeat}\}) \times \max(\text{PhyloP}, 1) \times (\text{PhastCons}_M + 0.1).$$

The positions in all the target sequences are defined with respect to the primary target sequence, and there is no explicit species-wise loss weighting applied. However, since we only compute loss on non-gap positions, there is an implicit downweighting of species that are more distant from the primary target, because the fraction of gap tokens tends to increase in the alignment as the species diverges further from the primary target species.

We also applied the data augmentation technique from Benegas *et al.* [22]. Before computing the loss, each position in the original target sequences is replaced by a random nucleotide with a certain probability q:

$$q = 0.5 \times \mathbb{1}\{\text{PhastCons}_M < p_{\text{neutral}}\}.$$

With this procedure, the high-confidence neutral sites defined by PhastCons will be randomly perturbed across training samples, so that the models learn from more diverse plausible sequences. The threshold p_{neutral} is set as 0.1 in all models except for the hg38 primate models, where the fraction of the defined neutral sites is much smaller than the others, and we thus raise the threshold to 0.2 in this regard.

All models are trained on a cluster of 8 NVIDIA A100 GPUs. For each alignment, we select a small fraction of chromosomes as the validation set, and the best model is selected based on the lowest validation loss over a predefined number of training steps. The full training setup of different models is described in Supplementary Table 1. The architecture hyperparameters at different model sizes are listed in Supplementary Table 10.

The hyperparameters for all the pretrained models of the same size are identical except for two: the context size and the clade-defining phylogenetic distance ϕ_{clade}. For these two hyperparameters, we used two sets of choices for the models based on the features of the alignments. Essentially, in alignments where the species are sufficiently distant (where the maximum phylogenetic distance is greater than 1), we use a larger threshold ϕ_{clade} of 0.2 to define clades, and a smaller context size of 128, since these alignments are highly fragmented; in other alignments where the species are all relatively close (where the maximum phylogenetic distance is below 1), we use a smaller ϕ_{clade} of 0.05 to model the more nuanced evolutionary signals, and also a larger context size of 256 because there are less intense rearrangements between the genomes. The models under the first setting include: `hg38-vertebrate`, `mm39`, `galGal6`, `dm6`, and `ce11`. The models under the second setting are: `hg38-mammal`, `hg38-primate`, and `tair10`.

Inference

When making inferences with the trained model, typically, only one target sequence is used as input, i.e., the primary target sequence. And all sequences are used as source sequences. When computing

the entropy or log-likelihood ratio for a given site, we select an alignment window centered around the site with a length equal to the model training context size. The central token is masked in the input target sequence, then the model predicts nucleotide probabilities at the site. We compute the log-likelihood ratio (LLR) for a variant as follows:

$$\mathrm{LLR} = \log \frac{p(\text{alt allele})}{p(\text{ref allele})},$$

and the entropy as follows:

$$H(p) = -\sum_{x \in \{A,C,G,T\}} p(x) \log p(x)$$

Following previous methodologies [2], we used LLR as the scoring function in most of the pathogenicity prediction and the rare variant association testing analysis. In the GWAS fine-mapped datasets, we used the absolute values of LLRs, since the studies prioritized variants based on the absolute effect sizes. For the complex trait heritability analysis, we initially started with absolute LLR, but later found that the entropy was more informative, which is surprising given that it loses allele specificity. One example where the entropy is more informative than the LLR is `rs149514089`, a putative causal variant for mean corpuscular volume and hemoglobin, located near a distal candidate enhancer [68] (Supplementary Figure 25). In this variant, both the reference and alternate allele have low frequencies in the alignment, so the LLR is close to 0.

To get the embedding of a genomic window of the target sequences, the aligned sequences of all species are used as source sequences. The input target sequence is unmasked in this case, and the hidden embeddings from the last encoder block are extracted as the output.

For the variant scores and embeddings, we average predictions from the forward and reverse strands.

Mutation Rate Calibration

Our model is trained on genomic sequences observed in nature. These sequences are shaped by various forces in the highly complex evolutionary processes. Apart from selection, the mutation rate is also an essential determinant of the observed sequences. To more accurately estimate genome-wide selective constraints using our model, we designed a simple yet effective calibration approach to remove mutation rate variation from our model predictions. Similar to conventional phylogenetic models like PhyloP and PhastCons, the basic idea is to estimate neutral model scores at high-confidence neutral sites and then use them to adjust the model scores when making predictions.

Our procedures are described as follows. First, we gather a set of high-confidence neutral sites. For vertebrate genomes, we find ancestral repeats in the target species by lifting over the repeats in the genome of a moderately distant species and overlapping those with the repeats in the target genome. Specifically, for the human models, we lifted over the repeats from the mouse; for the mouse model, we lifted over repeats from the human; and for the chicken model, we lifted over repeats from the Zebra Finch. Next, we further select the most confidently neutral sites by selecting sites within ancestral repeats with a PhyloP score from -0.1 to 0.1, and a PhastCons score of 0. For non-vertebrate species, because the ancestral repeats provide too few sites to reliably estimate the neutral scores, we skipped the first step and directly used genome-wide neutral sites defined by PhyloP (-0.05 to 0.05) and PhastCons (0). Second, we generate the model scores at the neutral sites, including the LLR and entropy. To get baseline model scores that describe site-specific mutation rates, we bin the neutral sites by their central pentanucleotide contexts, as it is known that the central k-mer context is a major determinant of mutation rates [79]. Finally, we

compute the mean model scores of variants with each pentanucleotide context, which is the baseline model score for a particular mutation rate bin determined by the pentanucleotide context and the mutation. We call them the neutral scores (i.e., $\text{LLR}_{neutral}$ and $H_{neutral}$). Then the neutral scores of the corresponding pentanucleotide bin are used to adjust the model predictions as:

$$\text{calibrated LLR} = \text{LLR} - \text{LLR}_{\text{neutral}}(x, \text{alt})$$

$$\text{calibrated entropy} = \frac{H}{H_{\text{neutral}}(x)},$$

where x is the central pentanucleotide context of the target site.

As shown in Extended Data Figure 10 and Supplementary Figure 26, this mutation rate calibration procedure reduced the correlations between the GPN-Star scores and Roulette mutation rate estimates to a negligible level and improved both constraint estimation and the performance on most benchmarks to different extents.

Visualization and Classification of Sequence Embeddings

We sought to visualize embeddings of a relatively balanced set of functional and non-functional genomics regions from human autosomes. We chose a resolution of 100-bp which is close to the median coding exon length in humans. We excluded all repeats from the analysis as these can use up a lot of the representational space [9] and make visualization of the remaining, more interesting, genomic regions difficult. We downloaded RepeatMasker [77] repeat annotation from the UCSC Genome Browser [70]. We utilized the gene annotation from Ensembl release 113 [67] and the cCRE catalog from ENCODE SCREEN v4 [68]. These annotations were also used in the other analyses in this study. Note that in the analysis of perfectly conserved sites, we also used experimentally validated enhancer annotations from VISTA Enhancer Browser [80, 81]. For each genomic element type (e.g. lncRNA or promoter) we filtered out regions overlapping other element types, using Bioframe [82]. We defined background regions as those not overlapping exons nor cCREs. For cCREs we focused on promoters and distal enhancers. We labeled windows as "conserved" when the 75th percentile of primate PhastCons [5] was 100%. For each functional element type, we sampled up to 10,000 "conserved" and 10,000 "non-conserved" regions. We sampled 20,000 background regions at random. Model embeddings of 100-bp windows were obtained by adding flanks up to 256-bp, obtaining embeddings per position, and averaging across the center 100 positions. Embeddings from the forward and reverse strand were averaged and then standardized. UMAP [83] was run with default parameters.

Classification was performed using logistic regression as implemented in scikit-learn [84], with class weights inversely proportional to class frequency and the default L_2 regularization strength. Models were trained on all chromosomes except the held-out chromosome used for prediction, following a leave-one-chromosome-out cross-validation scheme.

Nucleotide Dependency Analysis

Our approach is based on ref. [41]. Namely, we compute the dependency between positions i and j as:

$$e_{i,j} = \left\| \left\{ \log \left[\frac{\mathbb{P}(n_j = k \mid n_i = k_{\text{alt}})}{\mathbb{P}(n_j = k \mid n_i = k_{\text{ref}})} \right] \middle| k, k_{\text{alt}} \in \{\text{A}, \text{C}, \text{G}, \text{T}\}, k_{\text{alt}} \neq k_{\text{ref}} \right\} \right\|_\infty$$

where n_i is the nucleotide at position i. No nucleotides were masked when computing this score. Additionally, we symmetrize by averaging $e_{i,j}$ and $e_{j,i}$.

The LDLR MPRA data [65] was downloaded in processed form from ref. [85]. We applied the most stringent filter described in the paper: variants with at least 10 barcodes and p-value $< 10^{-5}$. To obtain a measure of effect size per position, we averaged the effect size of different alleles. The effect size for positions with no significant alleles was defined as 0. Finally, the effect size was smoothed by averaging over 5 consecutive positions.

Functional annotations and regulatory mechanisms for variants chrX:48824894 A>T, chr19:852326 A>T, and chr13:113105785 C>T were obtained from references [86], [87], and [88], respectively.

Evaluation Benchmarks

Homo sapiens

Datasets

- ClinVar [24]: downloaded release `20220924`. The goal is to classify missense variants labeled as "Pathogenic" vs. "Benign" and the metric is the area under the receiving precision-recall curve (AUPRC).

- COSMIC [29]: downloaded data processed by ref. [22]. Frequent mutations in cancer are defined as more than 0.1% of samples containing the mutation from the COSMIC Mutant-Census v98 dataset, restricting to whole-genome or whole-exome samples. The task is to classify COSMIC frequent vs. gnomAD [30] common missense variants, and the metric is the AUPRC.

- ProteinGym [31]: downloaded the 31 human protein data from ProteinGym v0.1 processed by ref. [25] into genomic coordinates, retaining only amino acid substitutions achievable with a single nucleotide change, and selecting the most deleterious nucleotide change according to CADD among all those that lead to the same amino acid substitution.

- OMIM [38]: downloaded processed Mendelian traits dataset from TraitGym [2]. Pathogenic variants curated from the literature were obtained from [89], and common variants used as controls were obtained from gnomAD [30]. Molecular consequences of variants were obtained with Ensembl VEP annotator [90] with the `--most_severe` flag. Each pathogenic variant was matched with nine control variants—matched by chromosome, consequence and distance to transcription start site (TSS). The task is to classify pathogenic vs. common regulatory variants and the metric is the AUPRC.

- HGMD [39]: downloaded curated data from ref. [91] and processed it similarly to the Mendelian traits dataset in TraitGym [2]. Molecular consequences of variants were obtained with Ensembl VEP annotator [90] with the `--most_severe` flag. Each pathogenic variant was matched with nine control variants—matched by chromosome, consequence and distance to transcription start site (TSS). The goal is to classify pathogenic vs. common regulatory variants and the metric is the AUPRC.

- GWAS fine-mapping [42, 92]: for non-coding variants, we downloaded processed complex traits dataset from TraitGym [2]. Molecular consequences of variants were obtained with Ensembl VEP annotator [90] with the `--most_severe` flag. Each positive variant was matched with nine control variants—matched by chromosome, consequence, distance to transcription start site (TSS), LD score and MAF. We processed coding variants from the same source [42,

92], but only matched MAF, similar to GeneticsGym [93]. The goal is to classify variants with high vs. low posterior inclusion probability (PIP) and the metric is the AUPRC.

- gnomAD [30]. The gnomAD v3.1.2 allele frequency data were downloaded from their official website `https://gnomad.broadinstitute.org/data`. We follow the evaluation protocol of GPN-MSA paper [22]. Enrichment of rare (singletons) vs. common (MAF > 5%) gnomAD variants in the tail of deleterious scores (the threshold defined by allowing a small number of false discoveries, here we use 30). We grouped certain variant consequences with small sample sizes, as follows. "splice-region" groups Ensembl categories `splice_donor`, `splice_acceptor`, `splice_donor_5th_base`, `splice_donor_region`, `splice_region`, and `splice_polypyrimidine_tract`. "start-or-stop" groups Ensembl categories `start_lost`, `stop_gained` or `stop_lost`. To reduce the number of variants for scoring, we subsampled the rare variants in each category to the same number of common variants.

  We also used the Gnocchi [30] constraint scores derived from gnomAD v3 data. The precomputed genome-wide z-scores for 1kb windows that passed quality control were downloaded from the same website. The range of the scores is from -10 to 10. We downsampled the windows to 100 per score bin of length 1, resulting in 2,000 windows. In our analysis, we compared the mean GPN-Star, PhyloP, and PhastCons scores across all possible variants in each window against the Gnocchi z-score.

For each benchmark presented in this paper, we only include variants where all the compared models have available predictions when computing the performance metrics.

Compared models

- AlphaGenome [37]: obtained scores through the official API, following the Enformer/Borzoi protocol in TraitGym [2] but replacing the L2 aggregation across tracks with a max operation, as recommended [37].
- Borzoi [36]: downloaded precomputed scores for OMIM and GWAS fine-mapping datasets [2], and followed a similar approach for HGMD. For LDSC, we downloaded precomputed scores from ref. [94] and performed the same procedures for aggregating the tracks as ref. [52].
- CADD [85]: downloaded precomputed CADD `v1.7` raw scores.
- ESM-1b [26]: log-likelihood ratio (LLR) precomputed by ref. [95] was downloaded in processed form from ref. [22].
- ESM-2 [27]: log-likelihood ratio (LLR) computed with the released models at `https://github.com/facebookresearch/esm`.
- ESM-3-open [28]: log-likelihood ratio (LLR) computed with the released model at `https://huggingface.co/biohub/esm3-sm-open-v1`.
- Enformer [35]: downloaded precomputed scores for OMIM and GWAS fine-mapping datasets [2], and followed a similar approach for HGMD. For LDSC, we downloaded precomputed scores from ref. [52], in particular tissue-agnostic feature `Enformer.Enformer_all_all` and tissue-specific features `Enformer.Enformer_<tissue>_all`.
- Evo 2 [4]: computed LLR using the 40B and 7B models with a context size of 8192-bp as in the paper.

- GPN-MSA [22]: downloaded precomputed LLR from https://huggingface.co/datasets/songlab/gpn-msa-hg38-scores.
- Nucleotide Transformer [3]: computed LLR using the v1 2.5B multispecies model.
- PhastCons (V): based on 100 vertebrates, downloaded from https://hgdownload.soe.ucsc.edu/goldenPath/hg38/phastCons100way/hg38.phastCons100way.bw.
- PhastCons (M): based on 470 mammals, downloaded from https://hgdownload.soe.ucsc.edu/goldenPath/hg38/phastCons470way/hg38.phastCons470way.bw
- PhastCons (P) [5]: based on 43 primates, downloaded from https://cgl.gi.ucsc.edu/data/cactus/zoonomia-2021-track-hub/hg38/phyloPPrimates.bigWig [1].
- PhyloP (V): based on 100 vertebrates, downloaded from https://hgdownload.soe.ucsc.edu/goldenPath/hg38/phyloP100way/hg38.phyloP100way.bw.
- PhyloP (M) [5]: based on 447 mammals, downloaded from https://hgdownload.soe.ucsc.edu/goldenPath/hg38/phyloP447way/hg38.phyloP447way.bw.
- PhyloP (P): based on 243 primates, downloaded from https://hgdownload.soe.ucsc.edu/goldenPath/hg38/phyloP447way/hg38.phyloP447wayPrimates.bw
- AlphaMissense [32]: downloaded precomputed scores for genomic variants on hg38.
- PrimateAI-3D [33]: obtained an academic license to download the scores from Illumina.
- SpeciesLM [41]: computed LLR with the model trained on animal genomes.
- PromoterAI [40]: obtained an academic license to download the scores from Illumina. We first took the absolute value of the score (to capture unsigned changes in expression rather than only over or under-expression). If a variant was predicted to affect multiple genes, we then took the maximum change in expression across all genes.
- GPN-Promoter [2]: computed LLR with the pretrained model from https://github.com/songlab-cal/gpn.
- Roulette [61]: precomputed genome-wide mutation rate estimates were downloaded from the GitHub repository https://github.com/vseplyarskiy/Roulette.

For all scores we computed, we averaged predictions from the forward and reverse strands.

Mus musculus

Datasets

- Wild Mouse Genome Project (WMGP): population allele frequency data were derived from a set of previously published datasets [96–103] under WMGP https://andrewparkermorgan.github.io/wmgp/. Raw data were adapter- and quality-trimmed using fastp [104]. The resultant reads were aligned to a minigraph-cactus [105] constructed pangenome composed of the

[1]The track name is misleading, but the documentation confirms it is indeed PhastCons: https://cgl.gi.ucsc.edu/data/cactus/zoonomia-2021-track-hub/hg38/phyloPPrimates.html

GRCm39 mouse reference and PacBio Continuous Long Read (CLR) assemblies of seven additional founder genomes of the Collaborative Cross mapping panel, plus PacBio CLR assemblies of BALB/cByJ, BALB/cJ, C3H/HeJ, C3H/HeOuJ, C57BL/6NJ, DBA/2J, PWD/PhJ [106] with VG giraffe [107]. The resultant alignments were surjected to the GRCm39 coordinate space in BAM format using VG [108]. Whole-genome variants were identified using DeepVariant [109] with the WGS model for each sample. Individual gVCF files were merged, and joint variant calling was conducted using GLnexus [110]. In our enrichment analysis, we defined singletons as rare variants and variants with allele frequencies above 0.2 as common variants. The molecular consequences were annotated following the same procedure as in the gnomAD dataset. To reduce the number of variants for scoring, we also followed the same procedure to subsample the rare variants.

- Mouse Mutant Resource Database (MMRdb): curated pathogenic variants were gathered from the latest MMRdb. MMRdb includes variant calls from more than 300 laboratory mouse strains with spontaneous disease phenotypes that exhibit Mendelian inheritance under cultivation at the Jackson Laboratory. A set of criteria for identifying likely variants associated with Mendelian disease phenotypes previously described in Fairfield *et al.* [111], based upon analysis of both exome and whole genome data sets, includes the following: variants will be rare in the database (<3%), the observed allele count in the sample will match the expected genotype (one allele for heterozygotes and two for homozygotes), and the chromosomal location of the variant will be consistent with existing linkage data. A subset of variants was derived from an earlier version of MMRdb [112], while the remainder was derived from a v.2.0.0 of the MMRdb, which can be accessed at `https://mmrdb.jax.org/`. All variants in the dataset were recorded in the GRCm38 reference genome coordinate space. In order to be compatible with the GRCm39 reference space used in the present model, we used the UCSC liftOver tool and associated chain files [113]. We filtered out variants that are not SNVs or with reference alleles different from the GRCm39 reference genome. We only retained variants in the following categories where there is a sufficient number of variants: missense, synonymous, nonsense, 3' UTR, 5' UTR and splicing. This curated dataset comprises 470 pathogenic or putatively pathogenic variants in total. Of the total dataset, 89 pathogenic variants have been confirmed via PCR validation (58 in Fairfield et al. 2015; 31 in version 2.0.0 of MMRdb), while the remaining 381 variants are classified as putatively pathogenic. We used the common variants from WMGP in the corresponding categories as the negative control in the benchmark.

Compared models

- PhastCons: based on 35 vertebrates, downloaded from `https://hgdownload.soe.ucsc.edu/goldenPath/mm39/phastCons35way/mm39.phastCons35way.bw`.
- PhyloP: based on 35 vertebrates, downloaded from `https://hgdownload.soe.ucsc.edu/goldenPath/mm39/phyloP35way/mm39.phyloP35way.bw`.

Drosophila melanogaster

Datasets

- Drosophila Evolution in Space and Time (DEST) [114]: downloaded allele frequency data from `https://berglandlab.pods.uvarc.io/vcf/dest.all.PoolSNP.001.50.24Aug202`

4.ann.vcf.gz. In our enrichment analysis, we defined variants with allele frequency below 0.002 as rare variants and those with allele frequency above 0.2 as common variants, as there are no singletons in the dataset. We applied the same procedure as in the gnomAD dataset to annotate the molecular consequences of the variants.

- FlyBase [56]: gathered experimentally validated lethal mutations from FlyBase https://flybase.org/ (FB2025_02, released April 17, 2025). We queried the database with the keywords "lethal", "point_mutation" and "na_change" to obtain annotated SNVs that lead to the lethal phenotypes. We found the vast majority (>99%) of the variants are either missense, nonsense, or splicing; we therefore restricted to these three categories. After matching the reference allele with the dm6 reference genome, we got 2,929 lethal variants in total. We used the common variants from DEST in the corresponding categories as the negative control in the benchmark.

Compared models

- PhastCons: based on 124 insects, downloaded from https://hgdownload.soe.ucsc.edu/goldenPath/dm6/phastCons124way/dm6.phastCons124way.bw.
- PhyloP: based on 124 insects, downloaded from https://hgdownload.soe.ucsc.edu/goldenPath/dm6/phyloP124way/dm6.phyloP124way.bw.

Caenorhabditis elegans

Datasets

- Caenorhabditis elegans Natural Diversity Resource (CaeNDR) [115]: downloaded allele frequency data from https://caendr-open-access-data-bucket.s3.us-east-2.amazonaws.com/dataset_release/c_elegans/20231213/variation/WI.20231213.hard-filter.isotype.vcf.gz. In our enrichment analysis, we defined variants with allele count 2 (one homozygous strain) as rare variants and those with allele frequency above 0.2 as common variants. We applied the same procedure as in the gnomAD dataset to annotate the molecular consequences of the variants.
- Experiment-validated lethal variants: obtained from the Supplementary data of Qin *et al.* [57]. We used the 72 SNVs in this dataset. We used the common variants from CaeNDR in the corresponding categories as the negative control in the benchmark.

Compared models

- PhastCons: based on 135 nematodes, downloaded from https://hgdownload.soe.ucsc.edu/goldenPath/ce11/phastCons135way/ce11.phastCons135way.bw.
- PhyloP: based on 135 nematodes, downloaded from https://hgdownload.soe.ucsc.edu/goldenPath/ce11/phyloP135way/ce11.phyloP135way.bw.

Gallus gallus

Datasets

- Galbase [116]: downloaded allele frequency data from http://animal.omics.pro/code/source/download/Chicken/variation/GRCg6a_SNPs.anno.tab.gz. In our enrichment analysis, we defined variants with allele frequency 0.001 (the minimum value in the dataset) as rare variants and those with allele frequency above 0.2 as common variants. We applied the same procedure as in the gnomAD dataset to annotate the molecular consequences of the variants.

Compared models

- PhastCons: based on 77 vertebrates, downloaded from https://hgdownload.soe.ucsc.edu/goldenPath/galGal6/phastCons77way/galGal6.phastCons77way.bw.
- PhyloP: based on 77 vertebrates, downloaded from https://hgdownload.soe.ucsc.edu/goldenPath/galGal6/phyloP77way/galGal6.phyloP77way.bw.

Arabidopsis thaliana

Datasets

- 1001 Genome Project [117]: downloaded allele frequency data processed in GPN paper [9] from https://huggingface.co/datasets/gonzalobenegas/processed-data-arabidopsis/resolve/main/variants/all/variants.parquet. In our enrichment analysis, we defined singletons as rare variants and those with allele frequency above 0.2 as common variants. We applied the same procedure as in the gnomAD dataset to annotate the molecular consequences of the variants.

Compared models

- PhastCons: based on 18 Brassicaceae, downloaded from PlantRegMap [73] http://plantregmap.gao-lab.org/download_ftp.php?filepath=08-download/Arabidopsis_thaliana/sequence_conservation/Ath_PhastCons.bedGraph.gz.
- PhyloP: based on 18 Brassicales, downloaded from PlantRegMap [73] http://plantregmap.gao-lab.org/download_ftp.php?filepath=08-download/Arabidopsis_thaliana/sequence_conservation/Ath_PhyloP.bedGraph.gz.

Heritability Enrichment Analysis

Background on stratified LD score regression (S-LDSC)

Following is a basic introduction to S-LDSC [48].

Notation. We begin by introducing some notation:

- N – GWAS sample size (individuals).
- M – number of SNPs analysed.
- $\mathbf{X} \in \mathbb{R}^{N \times M}$ – standardised genotype matrix.
- $\mathbf{y} \in \mathbb{R}^{N}$ – standardised phenotype.
- $\boldsymbol{\beta} \in \mathbb{R}^{M}$ – true additive effect (with β_j denoting the effect of SNP j).
- a_{jc} – value of annotation c at SNP j (binary, continuous or probabilistic).
- τ_c – per-SNP contribution of one *unit* of annotation c to heritability, conditional on all other annotations.
- $\boldsymbol{\varepsilon}$ – environmental residual vector with independent $\mathcal{N}(0, \sigma_e^2)$ entries.

Model. Under the additive polygenic assumption the phenotype can be written as:

$$\mathbf{y} \;=\; \mathbf{X}\boldsymbol{\beta} \;+\; \boldsymbol{\varepsilon}.$$

We assume that each SNP effect is an independent draw from a *zero-mean* Gaussian distribution whose variance depends linearly on the functional annotations:

$$\beta_j \sim \mathcal{N}\left(0,\; \sum_{c=1}^{C} a_{jc}\,\tau_c\right).$$

The total SNP heritability follows as:

$$h_g^2 \;=\; \sum_{j=1}^{M} \mathrm{Var}(\beta_j) \;=\; \sum_{c=1}^{C} \tau_c \sum_{j=1}^{M} a_{jc}.$$

Inference. Parameter estimates $\hat{\tau}_c$ are obtained by regressing genome-wide association chi-square statistics on the stratified LD scores using (generalised) least squares. The main statistical difficulty is the strong covariance among SNP-level statistics induced by linkage disequilibrium; S-LDSC sidesteps this by collapsing LD into per-SNP scores and by computing robust standard errors with a block jackknife over genomic windows.

Outputs. For each annotation c, S-LDSC reports two *estimated* statistics that summarize its relationship with heritability:

- Coefficient $\hat{\tau}_c$: The estimated conditional per-SNP contribution to heritability associated with a one-unit increase in the annotation, obtained while adjusting for all other annotations. A scale-free version of the coefficient [118],

$$\hat{\tau}_c^\star = \frac{\hat{\tau}_c \,\mathrm{sd}(a_{\cdot c})}{\hat{h}_g^2/M},$$

  represents the proportionate change in per-SNP heritability produced by a one-standard-deviation increase in the annotation. The normalization by the mean per-SNP heritability facilitates comparison across traits.

- Enrichment (binary annotations only):

$$\widehat{\mathrm{Enrichment}}_c = \frac{\hat{h}_c^2/\hat{h}_g^2}{\sum_{j=1}^{M} a_{jc}/M},$$

  where $\hat{h}_c^2 = \sum_j a_{jc}\,\widehat{\mathrm{Var}}(\beta_j)$ is the heritability at SNPs bearing the annotation (including contributions mediated through any overlap with other annotations). Enrichment compares the *proportion of heritability* to the *proportion of SNPs*; it is defined when $a_{jc} \in \{0, 1\}$.

S-LDSC analysis

Reference files, baseline annotations and summary statistics were downloaded from ref. [118] (latest dataset at ref. [23]). We used `baselineLD_v2.2` as baseline annotations. Of the 107 independent traits with summary statistics, we kept the 106 that were available without restrictions. To evaluate a model, we ran S-LDSC including the model score and also the baseline annotations. Running S-LDSC requires computing scores for around 10M variants, prohibiting this analysis for all but the most scalable models. To be precise, S-LDSC uses 9,997,231 reference variants with allele count ≥ 5 (MAF $\geq 0.52\%$) in 479 European individuals from 1000 Genomes Phase 3 [119]. Of these, 5,961,159 are common (MAF $\geq 5\%$) and have a central role when estimating heritability (please see [48] for further details). We binarized scores based on a quantile threshold. When the number of variants with tied PhastCons scores saturated at 1.0 exceeds the number of variants selected at the 0.1% quantile, we resolved rankings via random sampling. Since enrichment is only calculated on common variants, only these were used to determine the threshold so that all model annotations have the same proportion of variants. We perform a random-effects meta-analysis of enrichments and coefficients across traits. To assess significance, we perform a one-sided Wald test. The tissue-specific analysis reuses annotations from LDSC-SEG [53] (`gs://broad-alkesgroup-public-requester-pays/LDSCORE/LDSC_SEG_ldscores/Multi_tissue_gene_expr_1000Gv3_ldscores.tgz`). In this study, regions specific to each GTEx [120] tissue were defined by first finding the top 10% most differentially over-expressed genes in that tissue and then adding a 100 kb window around the genes. To better match the Enformer tissue aggregation [52], we grouped certain tissues by taking the union over their windows (Supplementary Table 11). Finally, tissue-specific GPN-Star scores were obtained by only picking top variants within tissue-specific regions defined by the union of the tissue-specifically expressed gene regions extended by 100 kb flanking windows [53] and the tissue-specific cCREs from ENCODE v4 [52]. We included seven tissues in our analysis for which both SEG annotations and cCREs are defined in the aforementioned sources. When comparing

tissue-agnostic and tissue-specific GPN-Star annotations, we made sure they both contained the same number of variants. One thing to note is that PhastCons (P) used a much smaller alignment (43 primates) compared to GPN-Star (P) presented in the results (243 primates). We confirmed the advantage of GPN-Star (P) is not mainly driven by the increased number of species by retraining it with only the 36 intersecting species between the two sets (Supplementary Figure 27).

Rare Variant Association Testing with DeepRVAT

Benchmarking of DeepRVAT with the published annotation set and enhanced with GPN-Star was carried out according to the same benchmarking procedure as for Fig. 4a in [7]. We describe the details of this in brief here.

UK Biobank WES data was processed and quality-controlled according to the procedure of the "UKBB 200k unrelated European ancestry dataset" from [7]. This dataset was derived from the UK Biobank 200K interim WES by including only individuals of European genetic ancestry and excluding those related to third degree or closer, resulting in a cohort of 161,822 individuals. This restriction was done for benchmarking in order to remove confounding effects of population stratification. Sequencing data processing, quality control, and annotation was carried out following [7], and variants with MAF $< 1\%$ were retained for training and variants with MAF $< 0.1\%$ were retained for association testing.

DeepRVAT models (package v1.1) with and without GPN-Star variant annotations were trained using stochastic parameter initialization for three different random seeds and the same 21 training phenotypes as in [7]. Burden testing was carried out as in Fig. 4a of [7], using REGENIE with DeepRVAT gene scores on the same 34 quantitative phenotypes (21 used during training and 13 not).

DeepRVAT discoveries were those with a Bonferroni-corrected p-value < 0.05. Replication was carried out by comparing significant gene-phenotype associations to reported discoveries from two studies [45, 46] on larger WES cohorts from UK Biobank (394,841 and 454,787 individuals, respectively).

Supplementary Tables

Supplementary Table 1: A summary of the training setups of all GPN-Star models in this work.

Model name	Genome assembly (size)	Train. chr.	Val. chr.	Held-out test chr.	WGA	aligned species	ϕ_{clade}	num. clades	context size	model size	learning rate	training steps	training time (8× A100)
hg38-vertebrate-200m	hg38 (3.1Gbp)	1-20, X, Y	21	22	multiz100way	100 vertebrates	0.2	45	128bp	200 M	5e-5	200 k	4.5 d
hg38-vertebrate-85m										85 M	1e-4	150 k	1.8 d
hg38-vertebrate-25m										25 M	1e-4	150 k	0.9 d
hg38-mammal-200m					cactus447way	447 mammals	0.05	145	256bp	200 M	5e-5	200 k	6.6 d
hg38-mammal-85m										85 M	1e-4	150 k	2.9 d
hg38-mammal-25m										25 M	1e-4	150 k	1.2 d
hg38-primate-200m						243 primates		21		200 M	5e-5	200 k	4.2 d
hg38-primate-85m										85 M	1e-4	150 k	1.6 d
hg38-primate-25m										25 M	1e-4	150 k	0.8 d
mm39-85m	mm39 (2.7Gbp)	1-17, X, Y	18	19	multiz35way	35 vertebrates	0.2	26	128bp	85 M	1e-4	150 k	1.6 d
mm39-25m										25 M	1e-4	150 k	0.7 d
galGal6-85m	galGal6 (1.1Gbp)	1-24, Z, W	25-28	30-33	multiz77way	77 vertebrates	0.2	29	128bp	85 M	1e-4	150 k	1.7 d
galGal6-25m										25 M	1e-4	150 k	0.7 d
dm6-85m	dm6 (144Mbp)	2L, 3L, 3R, X, Y	2R	4	multiz124way	124 insects	0.2	93	128bp	85 M	1e-4	50 k	0.8 d
dm6-25m										25 M	1e-4	50 k	0.3 d
ce11-85m	ce11 (100Mbp)	II, IV, V, X	I	III	multiz135way	135 nematodes	0.2	92	128bp	85 M	1e-4	50 k	0.8 d
ce11-25m										25 M	1e-4	50 k	0.3 d
tair10-85m	tair10 (120Mbp)	1-3	4	5	multiz18way	18 Brassicaceae	0.05	18	256bp	85 M	1e-4	50 k	0.5 d
tair10-25m										25 M	1e-4	50 k	0.2 d

Supplementary Table 2: Traits and tissues we expect them to be related to.

	brain	blood	liver	gut	heart	skin	lung
ADHD	1	0	0	0	0	0	0
Abdominal aortic aneurysm	0	0	0	0	0	0	0
Acne vulgaris	0	0	0	0	0	1	0
Acute appendicitis	0	0	0	1	0	0	0
Age First Birth	1	0	0	0	0	0	0
Aging Parental Lifespan	0	0	0	0	0	0	0
Alcohol use/AUDIT	1	0	0	0	0	0	0
Alkaline Phosphatase	0	0	1	1	0	0	0
Alzheimer's disease	1	0	0	0	0	0	0
Anorexia nervosa	1	0	0	0	0	0	0
Aspartate Aminotransferase	0	0	1	0	1	0	0
Asthma	0	1	0	0	0	0	1
Atrial Fibrillation	0	0	0	0	1	0	0
Atrial Fibrillation And Flutter	0	0	0	0	1	0	0
Autism Spectrum Disorder	1	0	0	0	0	0	0
BMI	1	0	0	0	0	0	0
Balding	0	0	0	0	0	1	0
Basophil Percentage	0	1	0	0	0	0	0
Bipolar disorder (all cases)	1	0	0	0	0	0	0
Breast Cancer (female)	0	0	0	0	0	0	0
Bronchitis, Emphysema, Asthma, Rhinitis, Eczema, Allergy Diagnosed By Doctor	0	1	0	0	0	1	1
CHIP	0	1	0	0	0	0	0
COPD	0	0	0	0	0	0	1
COPD - FVC	0	0	0	0	0	0	1
Cannabis use disorder	1	0	0	0	0	0	0
Celiac disease	0	1	0	1	0	0	0
Cholesterol	0	0	1	1	0	0	0
Cigarettes Per Day	1	0	0	0	0	0	0
Coronary Artery Disease (Aragam)	0	0	1	0	1	0	0
Covid 19 Vaccination	0	0	0	0	0	0	0
Creatinine	0	0	0	0	0	0	0
Daytime napping	1	0	0	0	0	0	0
Depression	1	0	0	0	0	0	0
Diastolic (Blood Pressure)	0	0	0	0	1	0	0
Diverticulosis And Diverticulitis	0	0	0	1	0	0	0
Drinks Per Week	1	0	0	0	0	0	0
Education Years	1	0	0	0	0	0	0
Endometriosis	0	0	0	0	0	0	0
Eosinophil Percentage	0	1	0	0	0	0	0
Esophageal Cancer	0	0	0	1	0	0	0
Ever Smoked	1	0	0	0	0	0	0
Fetal Birth Weight	0	0	1	0	0	0	0
GGE	1	0	0	0	0	0	0
Gastrointestinal Disease	0	0	0	1	0	0	0
General Risk Tolerance	1	0	0	0	0	0	0
Glaucoma	0	0	0	0	0	0	0
Gout	0	1	0	0	0	0	0
HDL	0	0	1	1	0	0	0
Heart Failure	0	0	0	0	1	0	0
Heel T-score	0	0	0	0	0	0	0
Height	0	0	0	0	0	0	0
Hypothyroidism (Self reported)	0	0	0	0	0	0	0
IBD	0	1	0	1	0	0	0
IGF1	0	0	1	0	0	0	0
Inguinal Hernia	0	0	0	0	0	0	0
Insomnia	1	0	0	0	0	0	0
Intelligence	1	0	0	0	0	0	0
KneeOA	0	0	0	0	0	0	0
LDL	0	0	1	1	0	0	0
Lung cancer	0	0	0	0	0	0	1
Lupus (SLE)	0	1	0	0	0	1	0
Malignant Neoplasms Of Skin	0	0	0	0	0	1	0
Maternal Birth Weight	0	0	0	0	0	0	0
Medication use (drugs used in diabetes)	0	0	0	0	0	0	0
Menarche Age	1	0	0	0	0	0	0
Monocyte Percentage	0	1	0	0	0	0	0
Morning Person	1	0	0	0	0	0	0
Mouth Teeth Dental Problems	0	0	0	0	0	0	0
Number Children Ever Born	1	0	0	0	0	0	0
Parkinson Disease	1	0	0	0	0	0	0
Phosphate	0	0	0	1	0	0	0
Physical Activity	1	0	0	0	0	0	0
Platelet Distribution Width	0	0	0	0	0	0	0
Primary Biliary Cirrhosis	0	1	1	0	0	0	0
Primary open-angle glaucoma	0	0	0	0	0	0	0
Prostate Cancer	0	0	0	0	0	0	0
Rbc Count	0	0	0	0	0	0	0
Rbc Distribution Width	0	0	0	0	0	0	0
Reaction Time	1	0	0	0	0	0	0
Reticulocyte Count	0	0	0	0	0	0	0
Rheumatoid Arthritis (Ishigaki)	0	1	0	0	0	0	0
Rheumatoid Arthritis (Saevarsdottir)	0	1	0	0	0	0	0
SARS-CoV-2 infection (C2)	0	0	0	1	0	0	1
Schizophrenia	1	0	0	0	0	0	0
Sleep Duration	1	0	0	0	0	0	0
Snoring	1	0	0	0	0	0	0
Stroke	0	0	1	0	1	0	0
Structural Connectivity Global (imaging)	1	0	0	0	0	0	0
Sunburn	0	0	0	0	0	1	0
T1D - Type 1 Diabetes	0	1	0	0	0	0	0
Telomere Length	0	0	0	0	0	0	0
Testosterone Male	0	0	0	0	0	0	0
ThumbOA	0	0	0	0	0	0	0
Total Bilirubin	0	0	1	0	0	0	0
Total Hip Replacement	0	0	0	0	0	0	0
Total Protein	0	0	1	0	0	0	0
Tourette Syndrome	1	0	0	0	0	0	0
Type 2 Diabetes	0	0	1	1	0	0	0
Varicose Vein Surgery	0	0	0	0	0	0	0
Varicose veins	0	0	0	0	0	0	0
Venous Thromboembolism (VTE)	0	0	1	0	0	0	0
Visceral Adipose Tissue Volume	0	0	0	0	0	0	0
Vitamin D	0	0	1	0	0	1	0
WHR BMI ratio	0	0	0	0	0	0	0
Wbc Count	0	1	0	0	0	0	0

Supplementary Table 3: Ablation studies. The performance metric is Spearman's ρ for ProteinGym and AUPRC for all the other benchmarks. The numbers outside the parentheses are the mean metrics across 3 random initializations with the same specified architecture, and the numbers inside the parentheses are the max metrics. The best performance in each column is marked in boldface, and the second best is underlined. All compared models were trained with the same dataset (multiz100way with training interval curation) and hyperparameters, including model size (25 million), learning rate (10^{-4}), batch size (512), and number of steps (150k). The MSA Transformer-like architecture neither uses species trees nor distinguishes target and source species. In each encoder block, row-wise self-attention is followed by column-wise self-attention operating on the full alignment.

	ClinVar	COSMIC	ProteinGym	Finemapped coding	OMIM	Finemapped non-coding	HGMD
GPN-Star default	**0.896** (**0.897**)	**0.391** (**0.395**)	**0.383** (**0.384**)	**0.239** (**0.241**)	0.713 (0.716)	**0.222** (**0.223**)	**0.570** (0.571)
only use one target species (human)	0.883 (0.884)	0.353 (0.354)	0.350 (0.353)	0.233 (0.233)	0.699 (0.703)	0.218 (0.220)	0.561 (0.563)
no evol. distance encoding	0.895 (0.895)	0.377 (0.385)	0.375 (0.375)	0.230 (0.234)	0.699 (0.703)	0.214 (0.216)	0.553 (0.557)
no pooling into clades	0.883 (0.886)	0.333 (0.343)	0.365 (0.367)	0.229 (0.236)	**0.720** (**0.725**)	0.209 (0.212)	0.570 (**0.575**)
no species tree (no evol. distances, no clades)	0.880 (0.882)	0.341 (0.353)	0.348 (0.354)	0.222 (0.227)	0.675 (0.681)	0.190 (0.192)	0.530 (0.537)
MSA Transformer-like architecture	0.887 (0.897)	0.351 (0.391)	0.350 (0.357)	0.223 (0.230)	0.696 (0.697)	0.195 (0.199)	0.548 (0.551)
GPN-MSA (exclude 10 closest species)	0.880 (0.881)	0.373 (0.384)	0.345 (0.348)	0.222 (0.226)	0.691 (0.693)	0.211 (0.211)	0.551 (0.553)
GPN-MSA (no species curation)	0.838 (0.844)	0.229 (0.265)	0.271 (0.282)	0.198 (0.203)	0.684 (0.699)	0.200 (0.204)	0.541 (0.553)

Supplementary Table 4: ENCODE SCREEN cCRE classes.

	TSS dist.	DNase/ATAC	H3K4me3	H3K27ac	CTCF	TF
promoter (PLS)	< 200	✓	✓			
proximal enhancer (pELS)	$< 2\,000$	✓		✓		
distal enhancer (dELS)	$> 2\,000$	✓		✓		
chromatin accessibility w/ H3K4me3 (CA-H3K4me3)		✓	✓	×		
chromatin accessibility w/ CTCF (CA-CTCF)		✓	×	×	✓	
chromatin accessibility w/ TF (CA-TF)		✓	×	×	×	✓
chromatin accessibility (CA)		✓	×	×	×	×
transcription factor (TF)		×	×	×	×	✓

Supplementary Table 5: Consequence distribution for the top 0.1% most constrained variants according to GPN-Star (P) (common variants only). Bold marks FDR < 5% from a two-sided Fisher's exact test.

consequence	count	proportion	odds ratio
missense	1983	0.33	**170.27**
dELS	1564	0.26	**2.39**
dELS-flank	468	0.08	**0.35**
ncRNA	287	0.05	**2.14**
3' UTR	280	0.05	**4.23**
pELS	209	0.04	**2.14**
5' UTR	151	0.03	**9.83**
CA	135	0.02	0.92
intron	99	0.02	**0.07**
PLS	96	0.02	**7.18**
CA-flank	92	0.02	**0.21**
synonymous	77	0.01	**4.11**
intergenic	67	0.01	**0.05**
pELS-flank	61	0.01	**0.57**
CA-CTCF	60	0.01	0.94
CA-H3K4me3	49	8e-03	1.07
splice-region	40	7e-03	**7.52**
CA-CTCF-flank	36	6e-03	**0.19**
splice-donor	28	5e-03	**50.38**
splice-donor-region	27	5e-03	**23.04**
TF-flank	24	4e-03	**0.14**
splice-donor-5th-base	22	4e-03	**52.98**
CA-TF	20	3e-03	1.30
splice-polypyrimidine-tract	18	3e-03	**4.19**
CA-H3K4me3-flank	15	3e-03	**0.14**
splice-acceptor	11	2e-03	**30.65**
CA-TF-flank	10	2e-03	**0.26**
stop-gained	10	2e-03	**41.91**
TF	9	2e-03	**0.15**
start-lost	4	7e-04	**68.72**
downstream-gene	3	5e-04	**0.15**
mature-miRNA	3	5e-04	**48.21**
stop-lost	2	3e-04	**22.64**
PLS-flank	1	2e-04	**0.17**

Supplementary Table 6: Consequence distribution for the top 0.1% most constrained variants according to GPN-Star (P) (all S-LDSC variants, common and low-frequency). Bold marks FDR < 5% from a two-sided Fisher's exact test.

consequence	count	proportion	odds ratio
missense	6494	0.40	**193.30**
dELS	3734	0.23	**2.02**
dELS-flank	1092	0.07	**0.30**
3' UTR	798	0.05	**4.25**
ncRNA	654	0.04	**1.76**
pELS	545	0.03	**2.01**
5' UTR	423	0.03	**9.52**
CA	322	0.02	**0.82**
PLS	255	0.02	**6.72**
intron	235	0.01	**0.06**
CA-flank	188	0.01	**0.16**
synonymous	164	0.01	**2.97**
pELS-flank	143	9e-03	**0.50**
intergenic	138	9e-03	**0.04**
CA-CTCF	115	7e-03	**0.67**
CA-H3K4me3	106	7e-03	0.85
splice-region	100	6e-03	**6.63**
splice-donor	94	6e-03	**58.71**
CA-CTCF-flank	79	5e-03	**0.16**
splice-donor-region	60	4e-03	**18.68**
stop-gained	51	3e-03	**56.16**
CA-H3K4me3-flank	51	3e-03	**0.18**
splice-donor-5th-base	44	3e-03	**38.80**
TF-flank	42	3e-03	**0.09**
splice-polypyrimidine-tract	38	2e-03	**3.17**
CA-TF	35	2e-03	0.85
splice-acceptor	30	2e-03	**30.80**
CA-TF-flank	19	1e-03	**0.18**
TF	17	1e-03	**0.10**
start-lost	16	1e-03	**84.74**
PLS-flank	7	4e-04	**0.44**
mature-miRNA	7	4e-04	**38.37**
stop-lost	6	4e-04	**26.35**
downstream-gene	4	2e-04	**0.07**
upstream-gene	2	1e-04	**0.06**

Supplementary Table 7: Consequences significantly enriched at the top common variants according to GPN-Star (P) vs. GPN-Star (M).

consequence	odds ratio	p-value	q-value
`missense`	1.14	7e-04	2e-02

Supplementary Table 8: Consequences significantly enriched at the top common variants according to GPN-Star (P) vs. GPN-Star (V).

consequence	odds ratio	p-value	q-value
`synonymous`	3.89	4e-09	5e-08
`5' UTR`	1.89	4e-06	2e-05
`PLS`	1.76	1e-03	3e-03
`missense`	1.52	5e-25	2e-23
`3' UTR`	1.36	1e-03	4e-03
`dELS-flank`	0.62	7e-15	1e-13
`CA`	0.62	1e-05	6e-05
`CA-flank`	0.56	8e-06	4e-05
`CA-CTCF-flank`	0.55	5e-03	1e-02
`intron`	0.54	1e-06	9e-06
`intergenic`	0.49	2e-06	2e-05
`CA-H3K4me3-flank`	0.34	2e-04	8e-04

Supplementary Table 9: Alignments obtained from the UCSC Genome Browser.

	Reference	Alignment
H. sapiens	`hg38`	`multiz100way` (100 vertebrates)
M. musculus	`mm39`	`multiz35way` (35 vertebrates)
G. gallus	`galGal6`	`multiz77way` (77 vertebrates)
D. melanogaster	`dm6`	`multiz124way` (124 insects)
C. elegans	`ce11`	`multiz135way` (135 nematodes)

Supplementary Table 10: Hyperparameters of the GPN-Star architecture at different model sizes.

Model size	25 M	85 M	200 M
Num encoder blocks (K)	8	12	16
Hidden dim (H)	512	768	1024
Intermediate size (F)	2048	3072	4096
Num self-attn heads (A)	4	6	8
Num cross-attn heads (A)	4	6	8
Attn head dim (D)	64	64	64
Dropout rate	0.1	0.1	0.1

Supplementary Table 11: Grouping of GTEx tissues.

	GTEx tissue
brain	Brain Substantia nigra<br>Brain Spinal cord (cervical c-1)<br>Brain Amygdala<br>Brain Anterior cingulate cortex (BA24)<br>Brain Hippocampus<br>Brain Hypothalamus<br>Brain Putamen (basal ganglia)<br>Brain Cerebellar Hemisphere<br>Brain Frontal Cortex (BA9)<br>Brain Nucleus accumbens (basal ganglia)<br>Brain Cortex<br>Brain Caudate (basal ganglia)<br>Brain Cerebellum
blood/immune	Spleen<br>Cells EBV-transformed lymphocytes<br>Whole Blood
liver	Liver
gut	Small Intestine Terminal Ileum<br>Colon Sigmoid<br>Esophagus Gastroesophageal Junction<br>Stomach<br>Colon Transverse<br>Esophagus Muscularis<br>Esophagus Mucosa
heart	Heart Atrial Appendage<br>Heart Left Ventricle
skin	Skin Not Sun Exposed (Suprapubic)<br>Cells Transformed fibroblasts<br>Skin Sun Exposed (Lower leg)
lung	Lung

Supplementary Figures

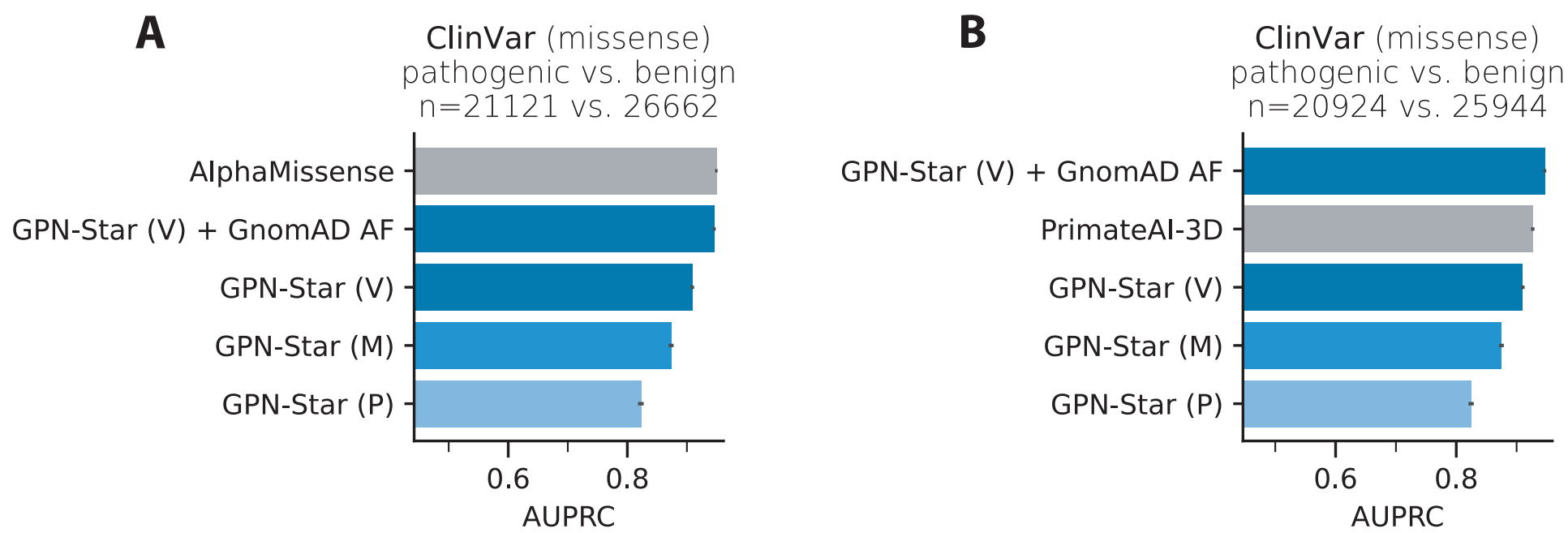

Supplementary Figure 1: Head-to-head comparisons with AlphaMissense and PrimateAI-3D on ClinVar missense variant effect prediction. These two models leverage human population allele frequencies during training and thus gain an advantage in predicting ClinVar variants, as allele frequencies are used to define benign variants. The method GPN-Star (V) + gnomAD AF is adjusting GPN-Star (V) predictions by assigning the most benign score to those variants with allele frequency $> 2 \times 10^{-4}$ in gnomAD v3. The threshold is chosen according to the definition of benign variants in AlphaMissense training data. The error bars represent the 95% confidence intervals based on 1,000 class-stratified bootstrap resamples.

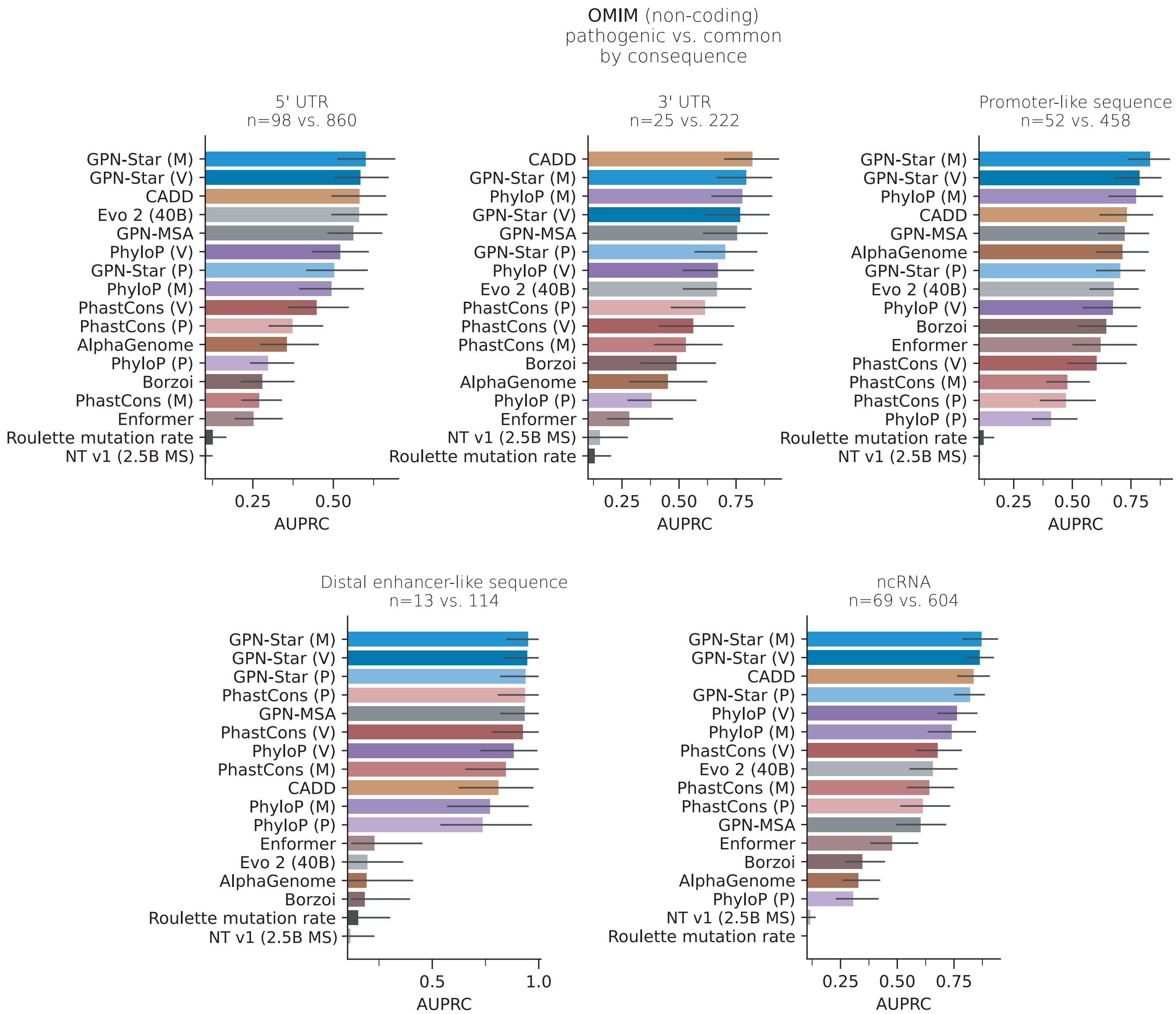

Supplementary Figure 2: Model performances on the OMIM benchmarks stratified by molecular consequences. The datasets and figure setups are the same as Figure 2 otherwise. Only categories with at least 10 positive variants were retained.

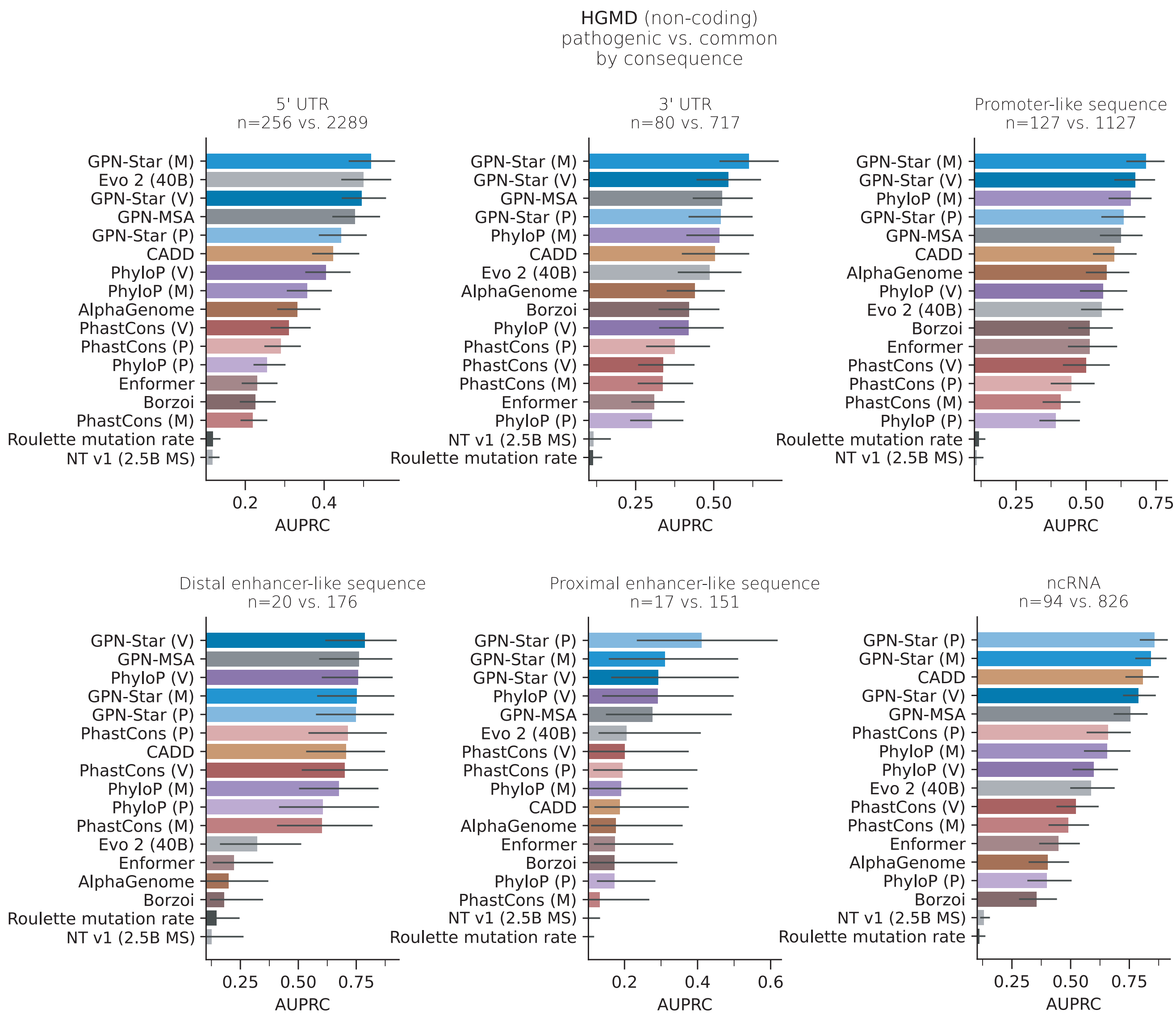

Supplementary Figure 3: Model performances on HGMD benchmark stratified by molecular consequences. The datasets and figure setups are the same as Figure 2 otherwise. Only categories with at least 10 positive variants were retained.

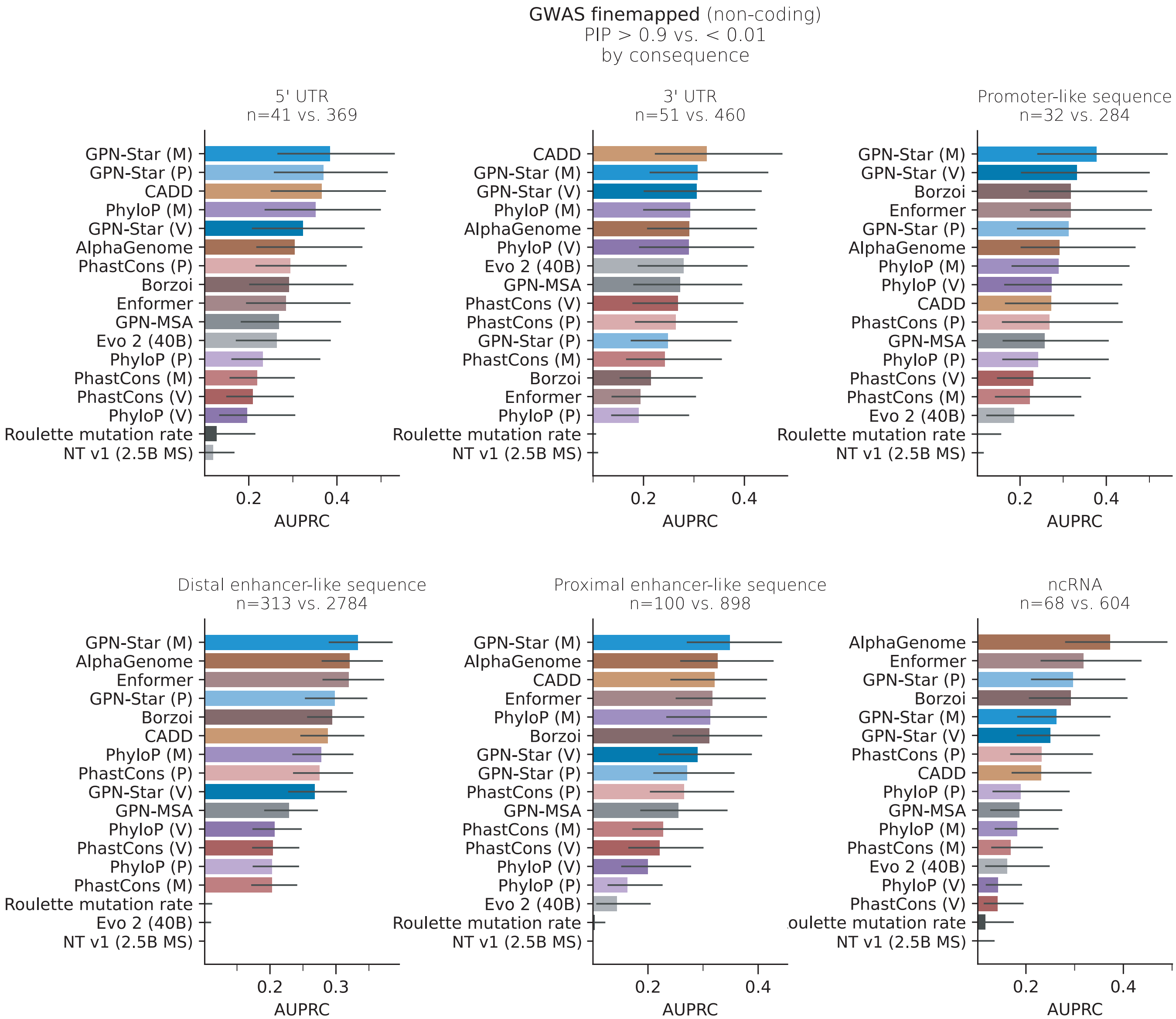

Supplementary Figure 4: Model performances on GWAS fine-mapped non-coding variant benchmark stratified by molecular consequences. The datasets and figure setups are the same as Figure 2 otherwise. Only categories with at least 10 positive variants were retained.

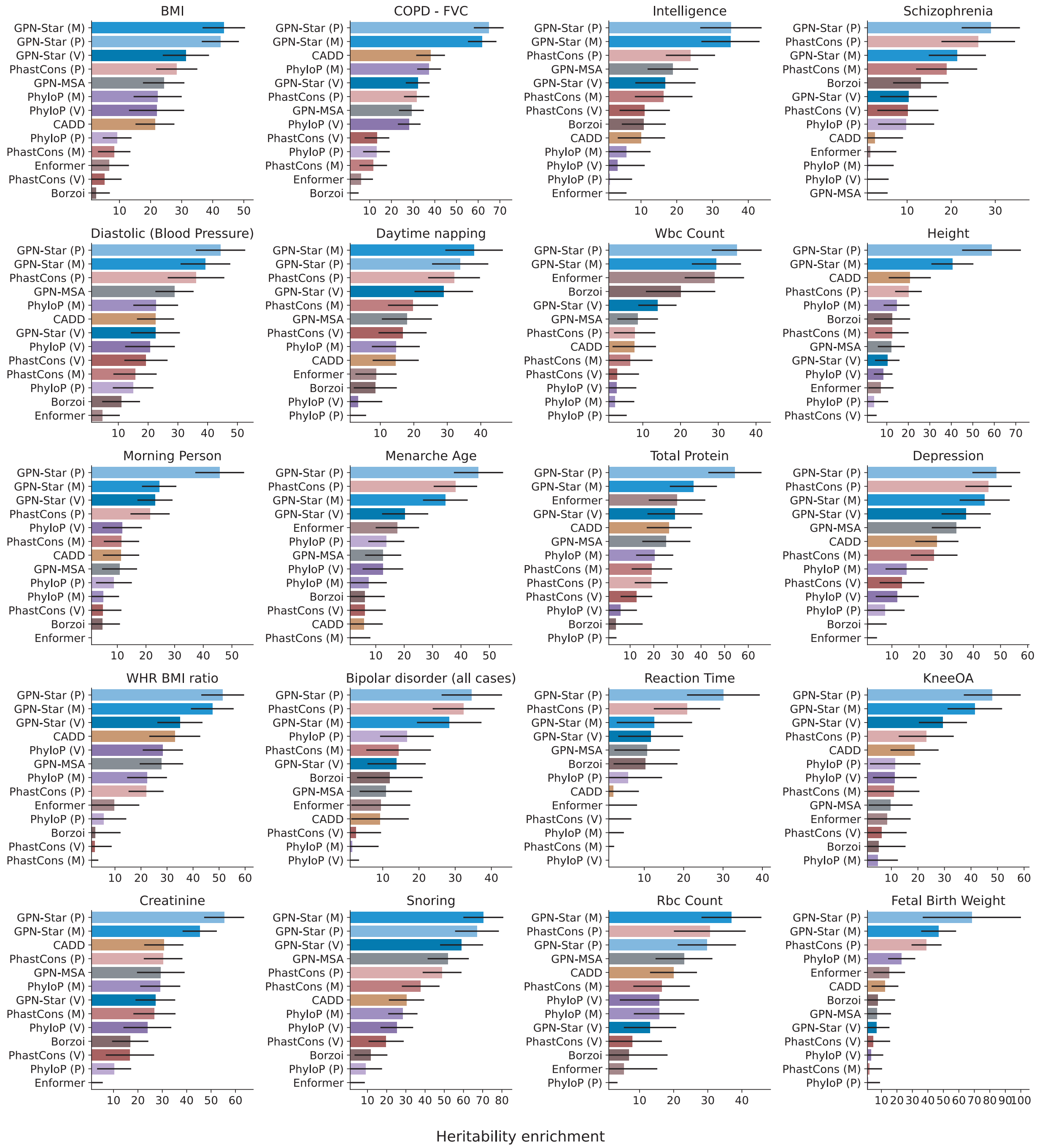

Supplementary Figure 5: Heritability enrichment by model predictions of the 20 most heritable traits. Same as in Figure 3, here we showed the enrichment among the top 0.1% most constrained variants defined by each model.

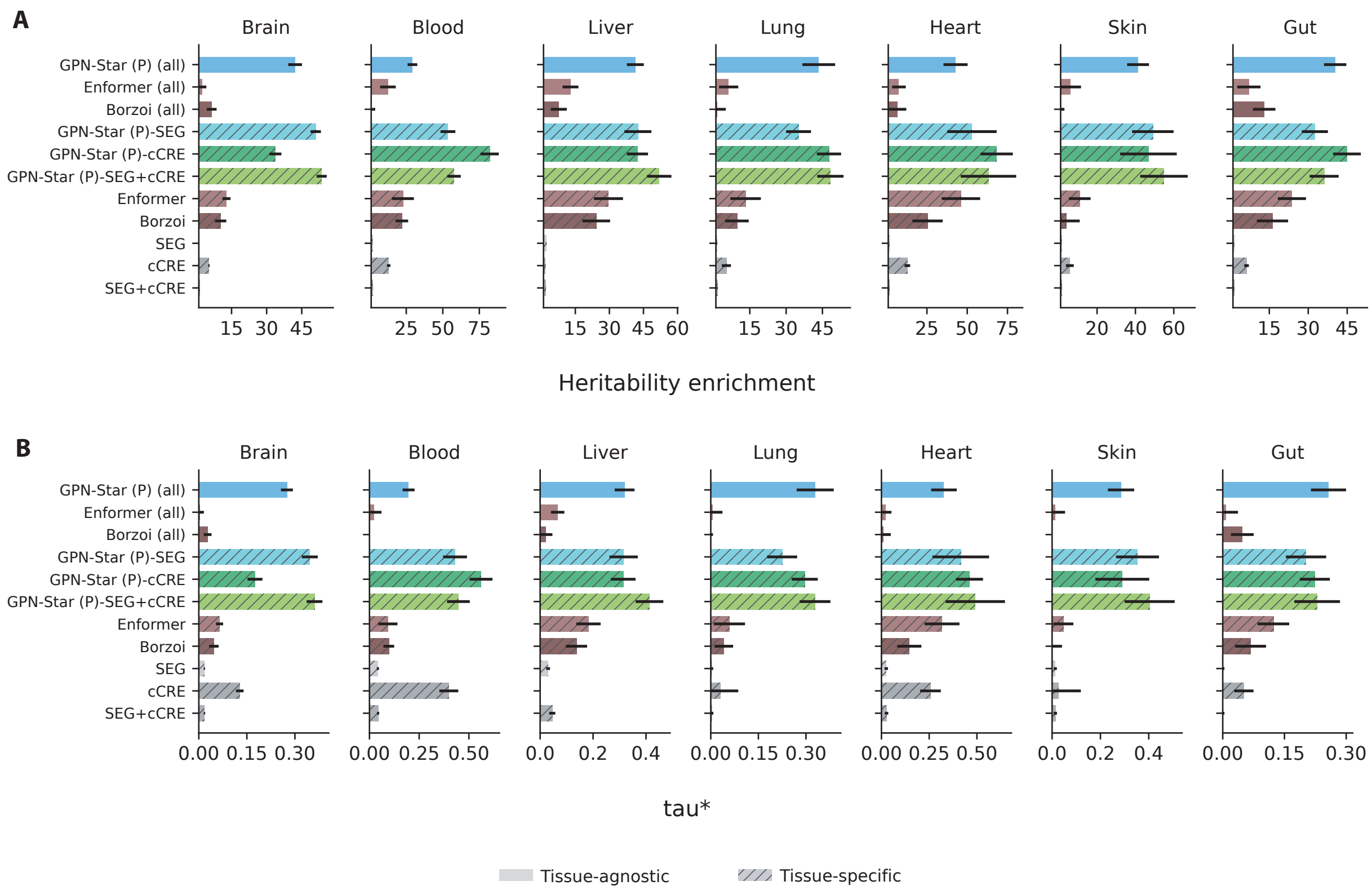

Supplementary Figure 6: Comparisons of different approaches of constructing tissue-specific GPN-Star annotations and different tissue-specific baseline annotations. SEG+cCRE represents taking the union of the two sets of regions.

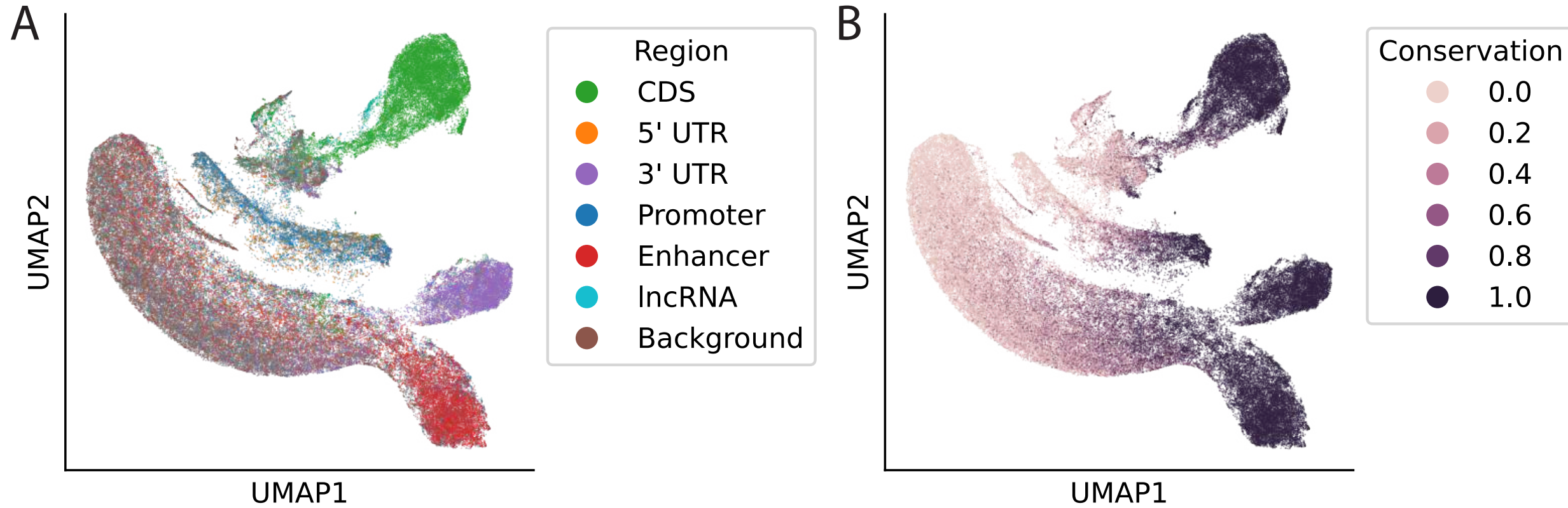

Supplementary Figure 7: Visualization of GPN-Star (M) model with 85 M parameters reveals functional elements. **(A)** Visualization of embeddings of different genomic windows, colored by their annotated region. **(B)** Visualization of embeddings of different genomic windows, colored by conservation, defined as the 75th percentile of PhastCons (P) in the window.

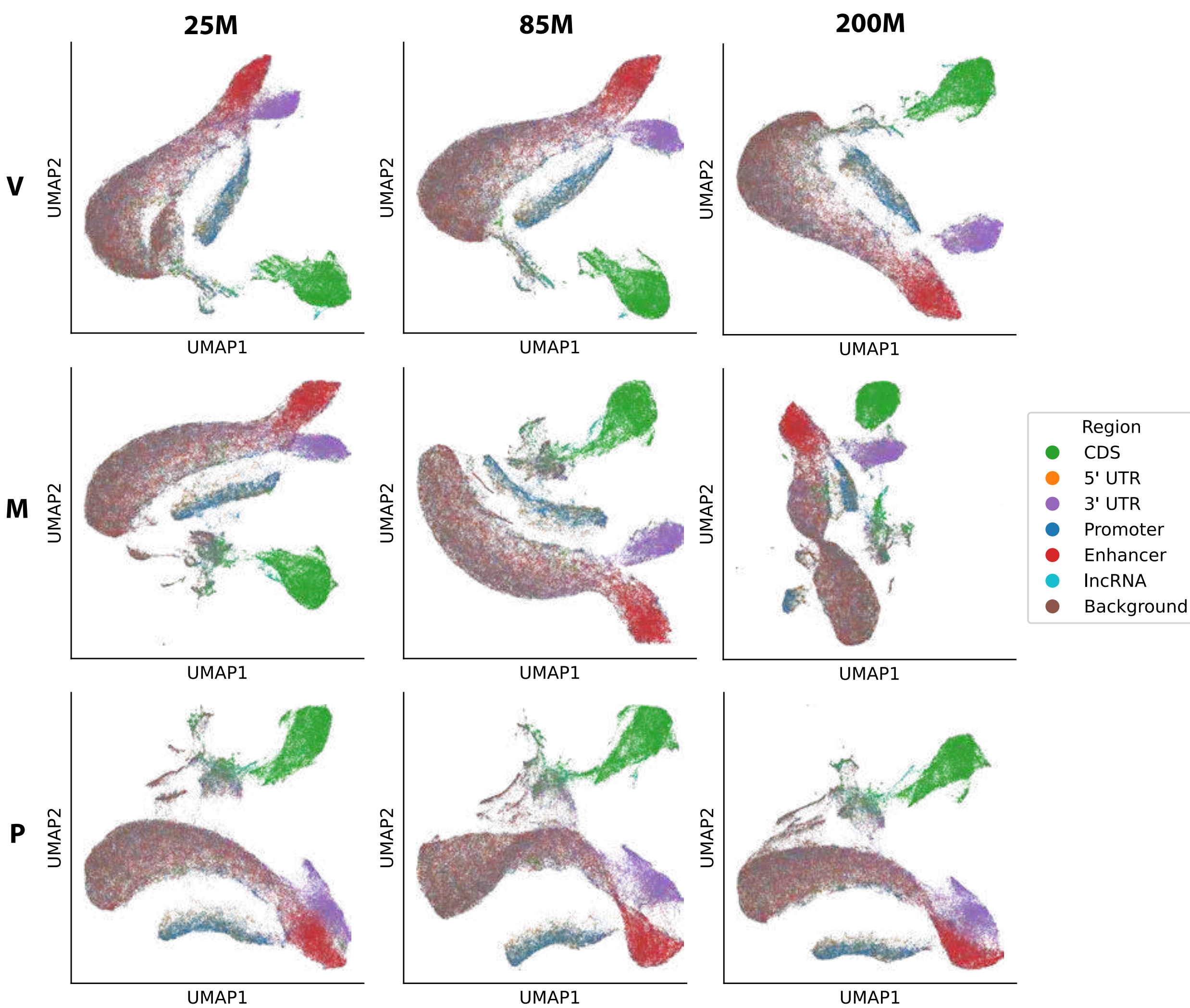

Supplementary Figure 8: Visualization of window embeddings (all models). The rows represent models trained on different evolutionary timescales (V: vertebrates, M: mammals, P: primates) and the columns represent different model sizes (number of parameters).

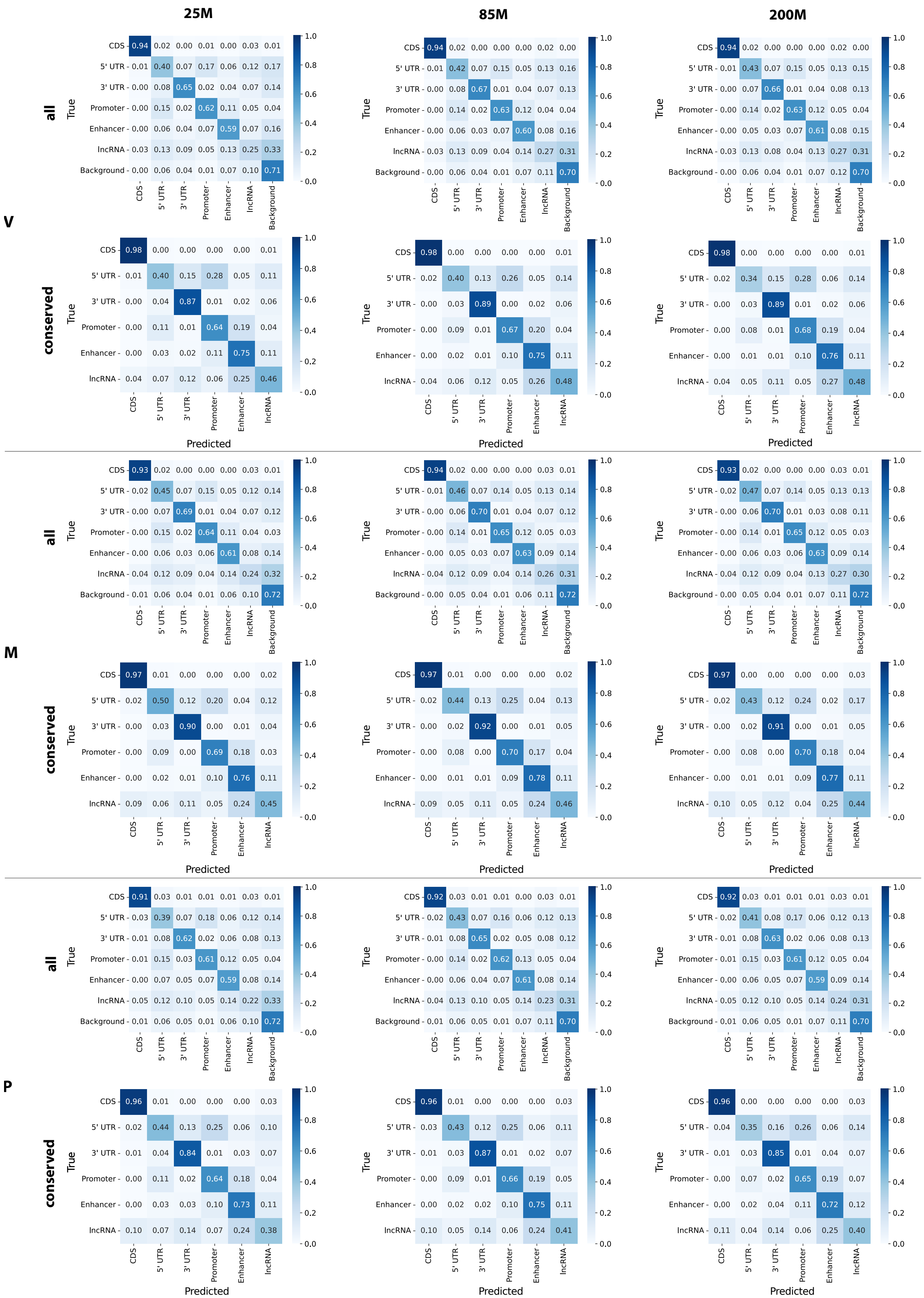
25M
85M
200M
V
M
P
all
conserved
True
Predicted
CDS
5' UTR
3' UTR
Promoter
Enhancer
lncRNA
Background

Supplementary Figure 9: Confusion matrices for the classification of window embeddings by logistic regression for all models. The rows represent models trained on different evolutionary timescales (V: vertebrates, M: mammals, P: primates) and the columns represent different model sizes (number of parameters). Each row contains two subrows corresponding to results for all genomic windows (all) or only conserved windows (conserved).

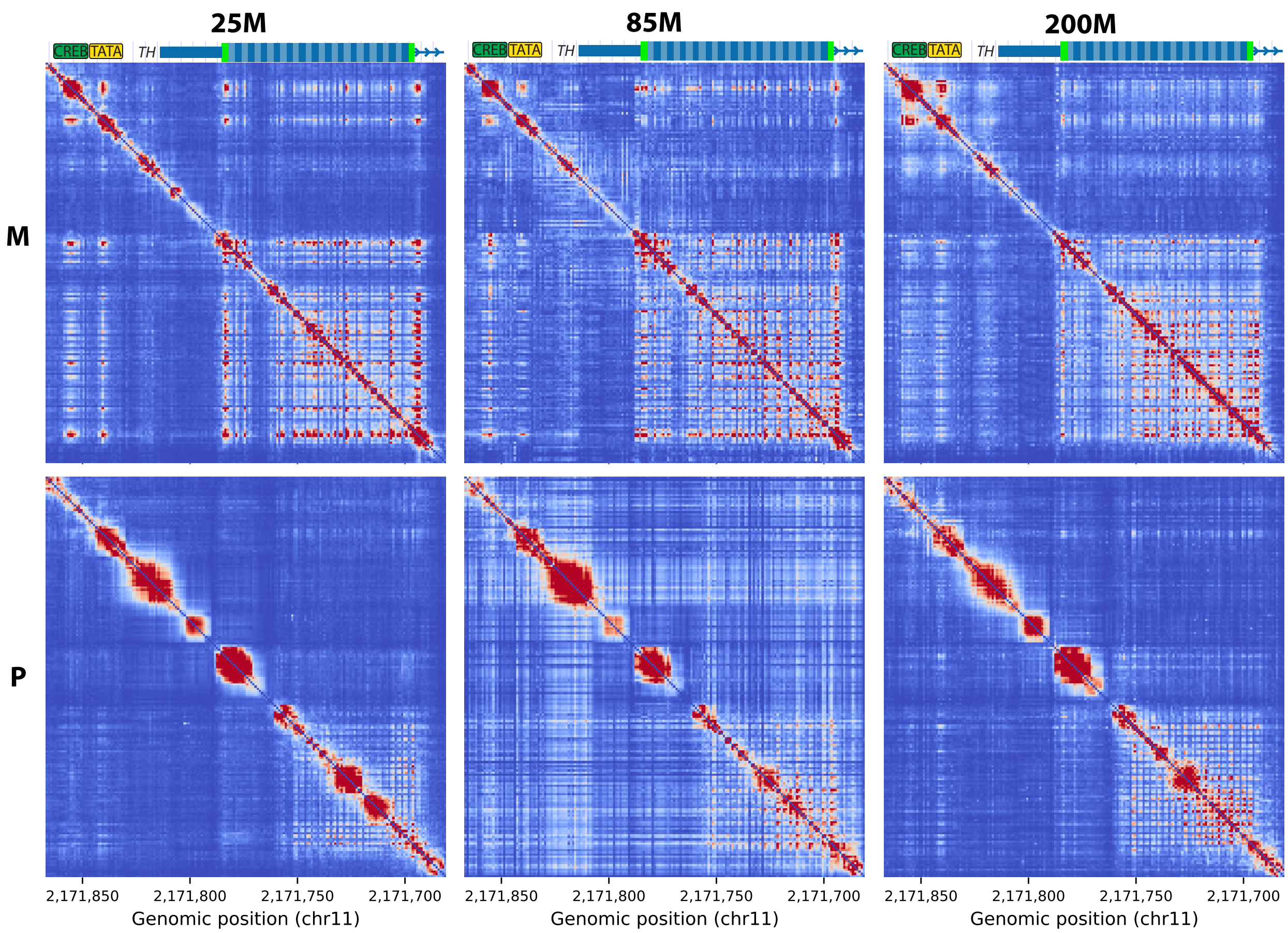

Supplementary Figure 10: Nucleotide dependency map in *TH* promoter (vertebrate models excluded as this region exceeds its context size). The rows represent models trained on different evolutionary timescales (V: vertebrates, M: mammals, P: primates) and the columns represent different model sizes (number of parameters).

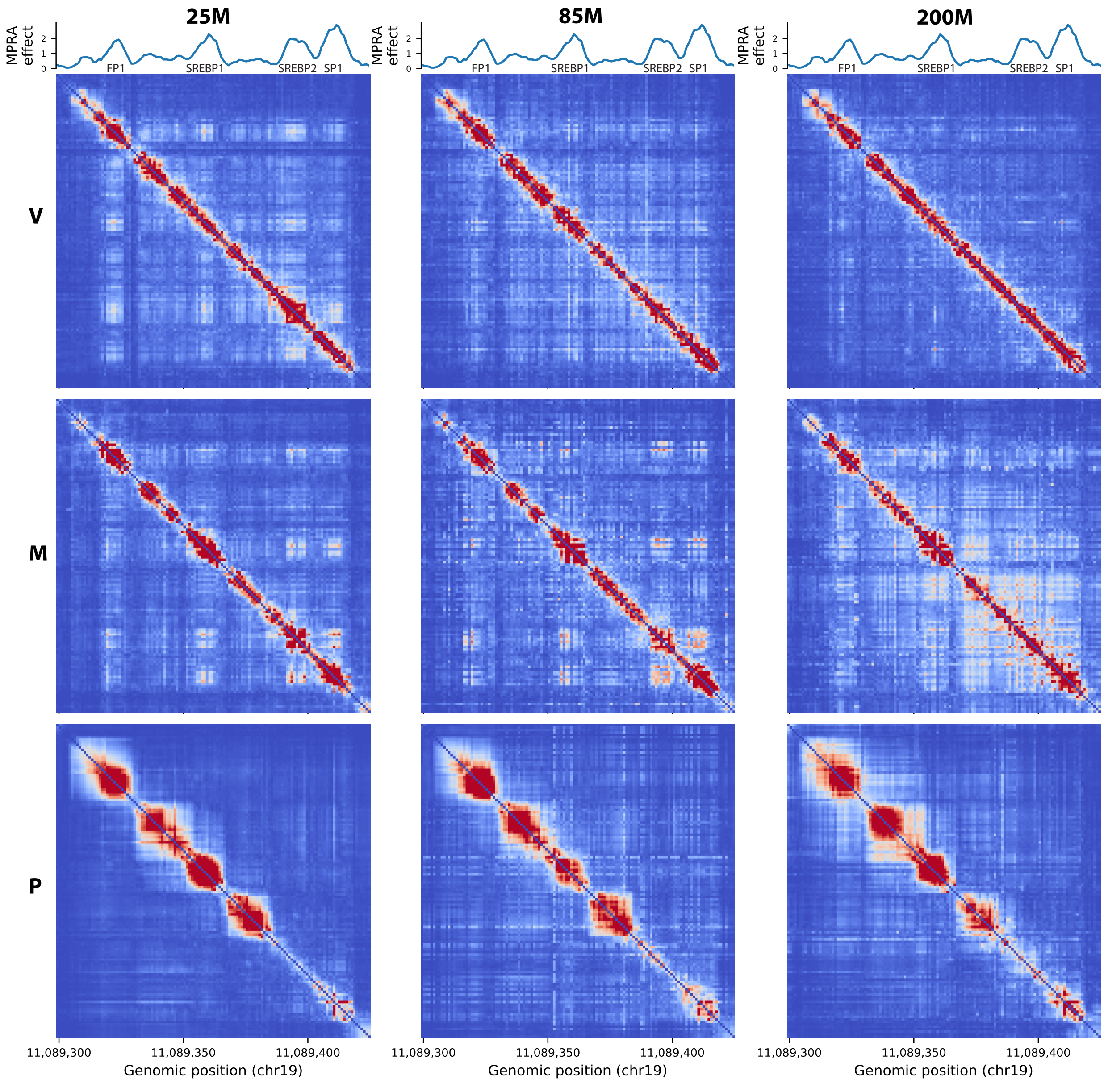

Supplementary Figure 11: Nucleotide dependency maps for *LDLR* promoter (all models). The rows represent models trained on different evolutionary timescales (V: vertebrates, M: mammals, P: primates) and the columns represent different model sizes (number of parameters).

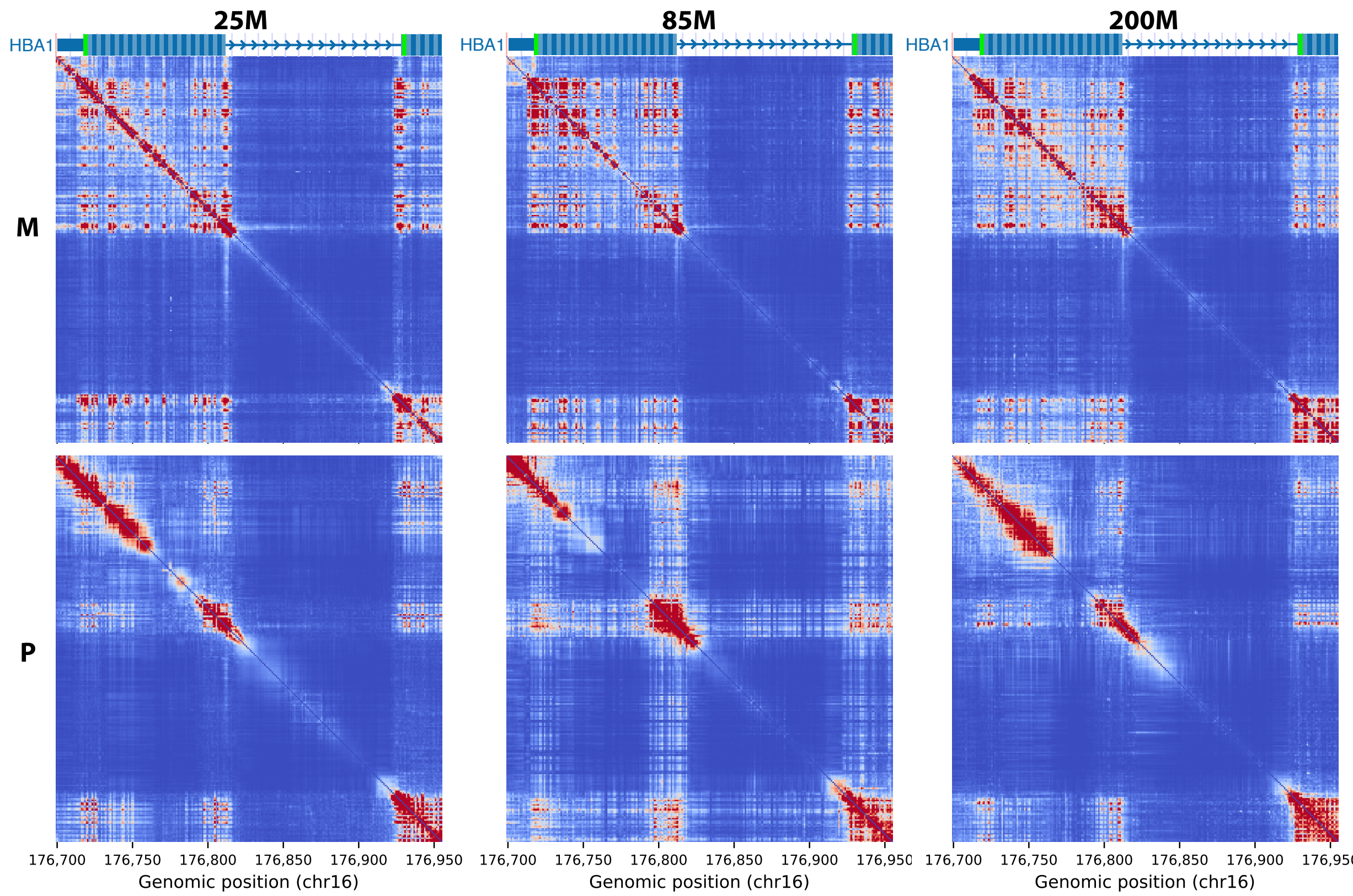

Supplementary Figure 12: Nucleotide dependency maps around the first two exons of *HBA1*, a gene with exceptionally small introns that fit within the model's context size. Vertebrate models excluded as this region exceeds its context size. Notably, dependencies between splice donor and acceptor regions were especially strong, consistent with previous findings [41]. The rows represent models trained on different evolutionary timescales (V: vertebrates, M: mammals, P: primates) and the columns represent different model sizes (number of parameters).

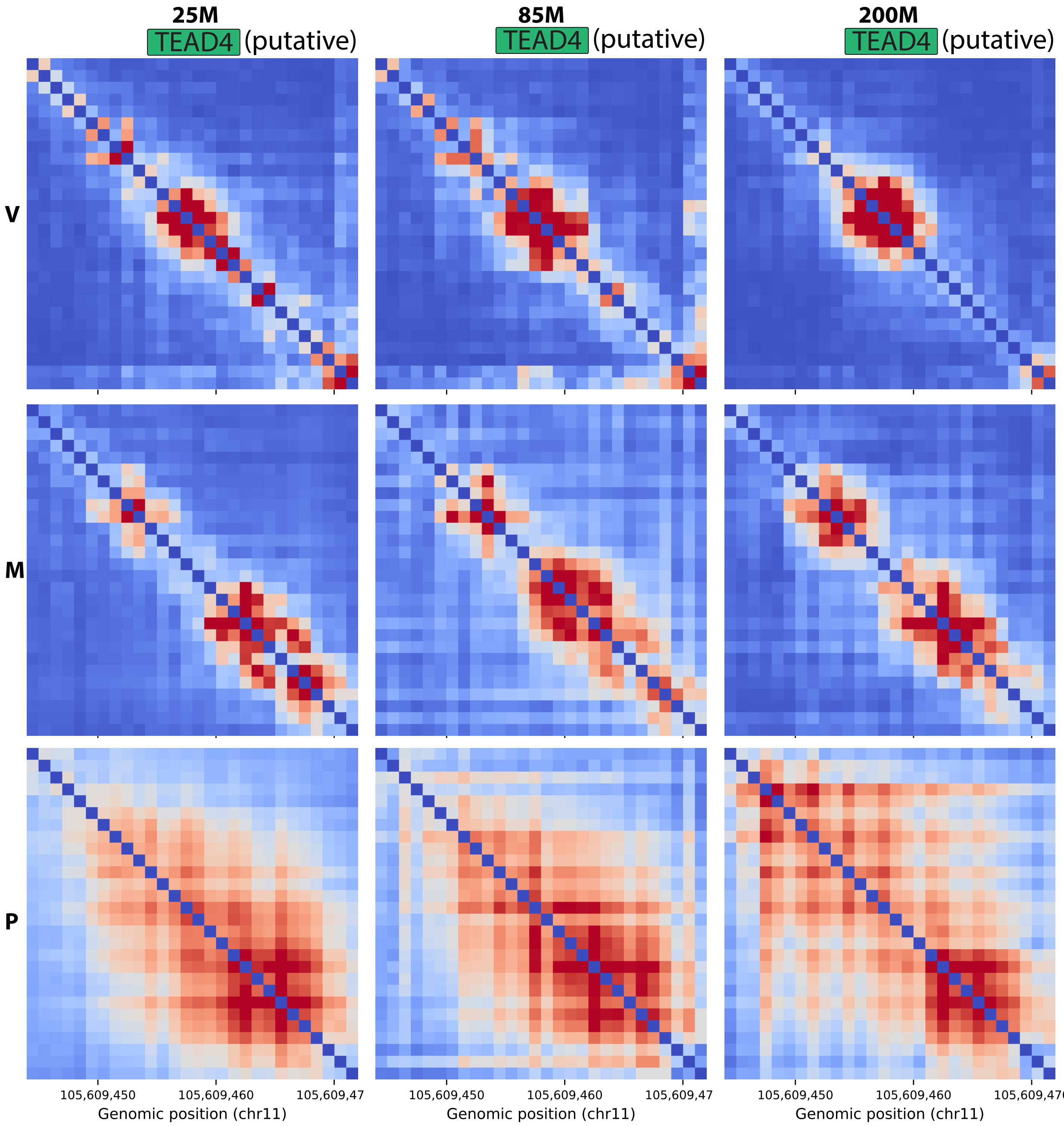

Supplementary Figure 13: Nucleotide dependency maps (all models) for an accessible region near *GRIA4* hypothesized to be under primate-specific constraint [6]. Supporting this hypothesis, the GPN-Star (P) model showed the highest level of dependency around a putative TEAD4 binding site. The rows represent models trained on different evolutionary timescales (V: vertebrates, M: mammals, P: primates) and the columns represent different model sizes (number of parameters).

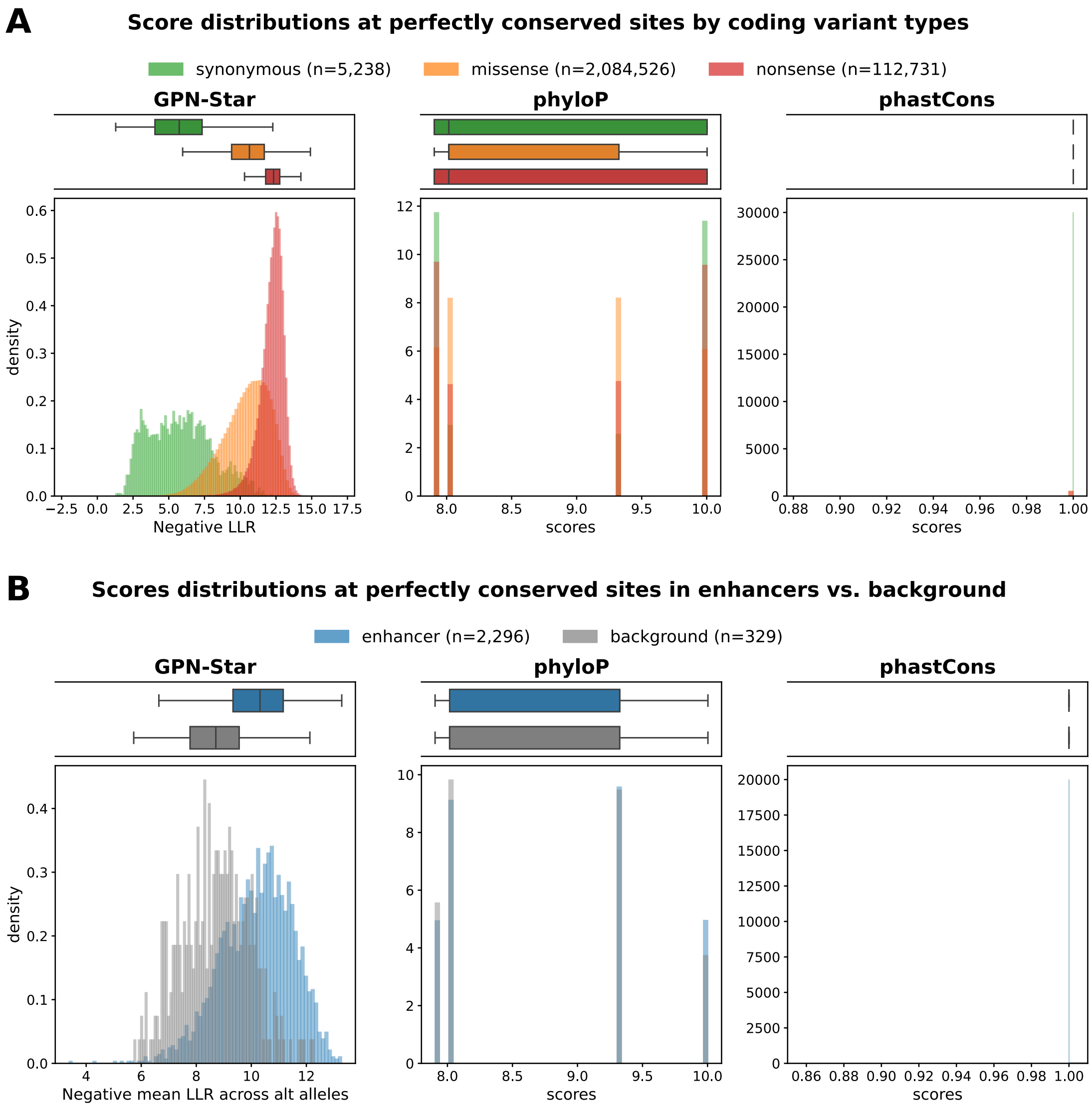

Supplementary Figure 14: Comparison of score distributions generated by GPN-Star, PhyloP, and PhastCons for different variant types at perfectly conserved sites in the human genome. Perfect conservation is defined as the presence of a single, gap-free nucleotide across all species in the multiz100way alignment, and all scores are computed with respect to this same alignment. (A) Nonsense, missense, and synonymous single-nucleotide variants. (B) Enhancer versus background sites. Experimentally validated enhancer annotations are obtained from the VISTA Enhancer Browser, and background sites are selected from as non-N genomic regions that exclude all exons, ENCODE V4 cCREs, and RepeatMasker repeats. The four discrete PhyloP score values observed at these perfectly conserved sites arise from nucleotide-specific neutral substitution rates for the four reference bases.

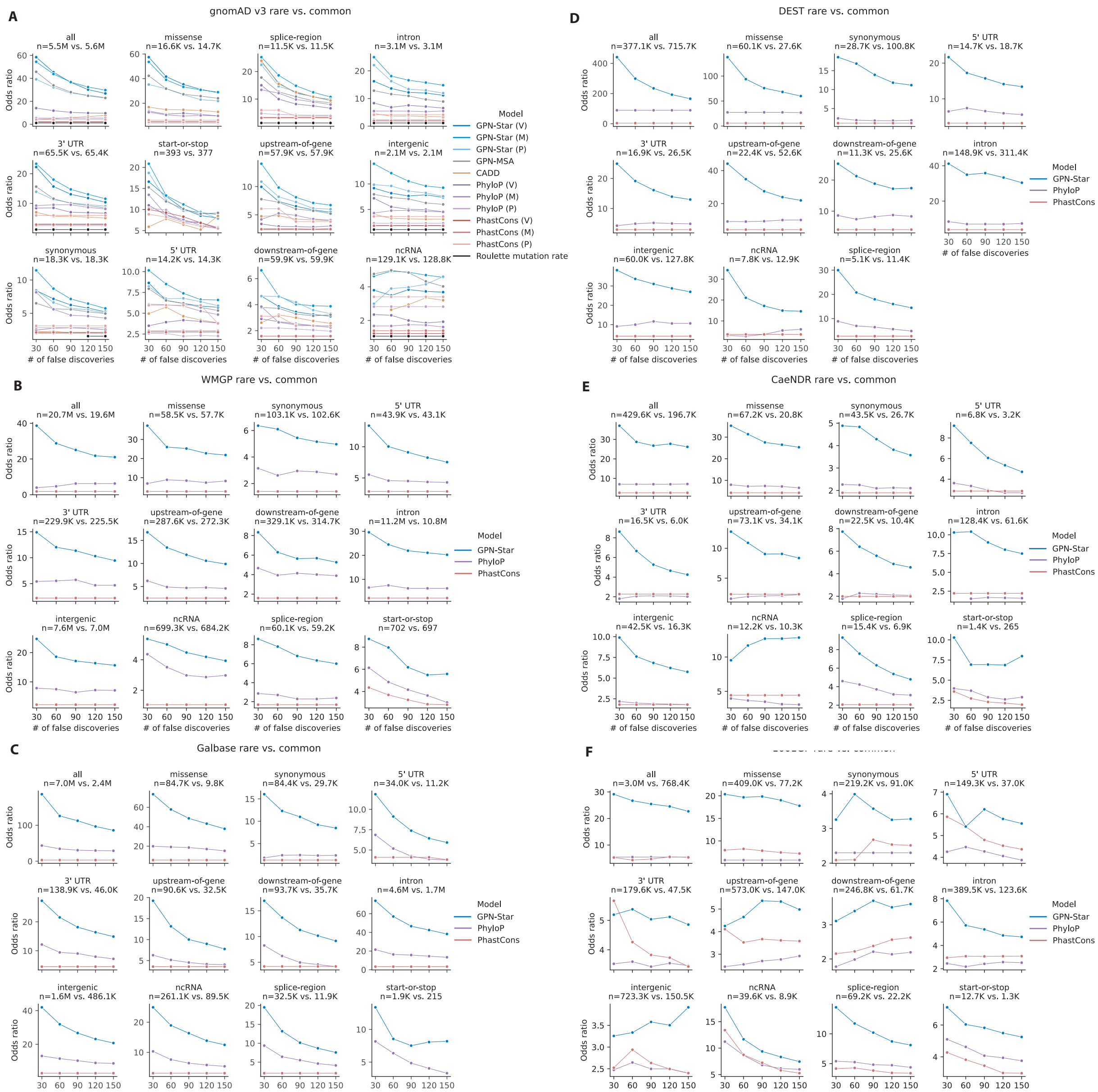

Supplementary Figure 15: Enrichment of rare versus common variants in the tail of deleterious scores in population allele frequency datasets, varying the threshold of deleteriousness from 30 to 150 false discoveries. Data points are absent when enrichment is not significant by Fisher's exact test. (A) gnomAD v3 dataset in human. (B) WMGP dataset in mouse. (C) Galbase dataset in chicken. (D) DEST dataset in *D. melanogaster*. (E) CaeNDR dataset in *C. elegans*. (F) 1001GP dataset in *A. thaliana*.

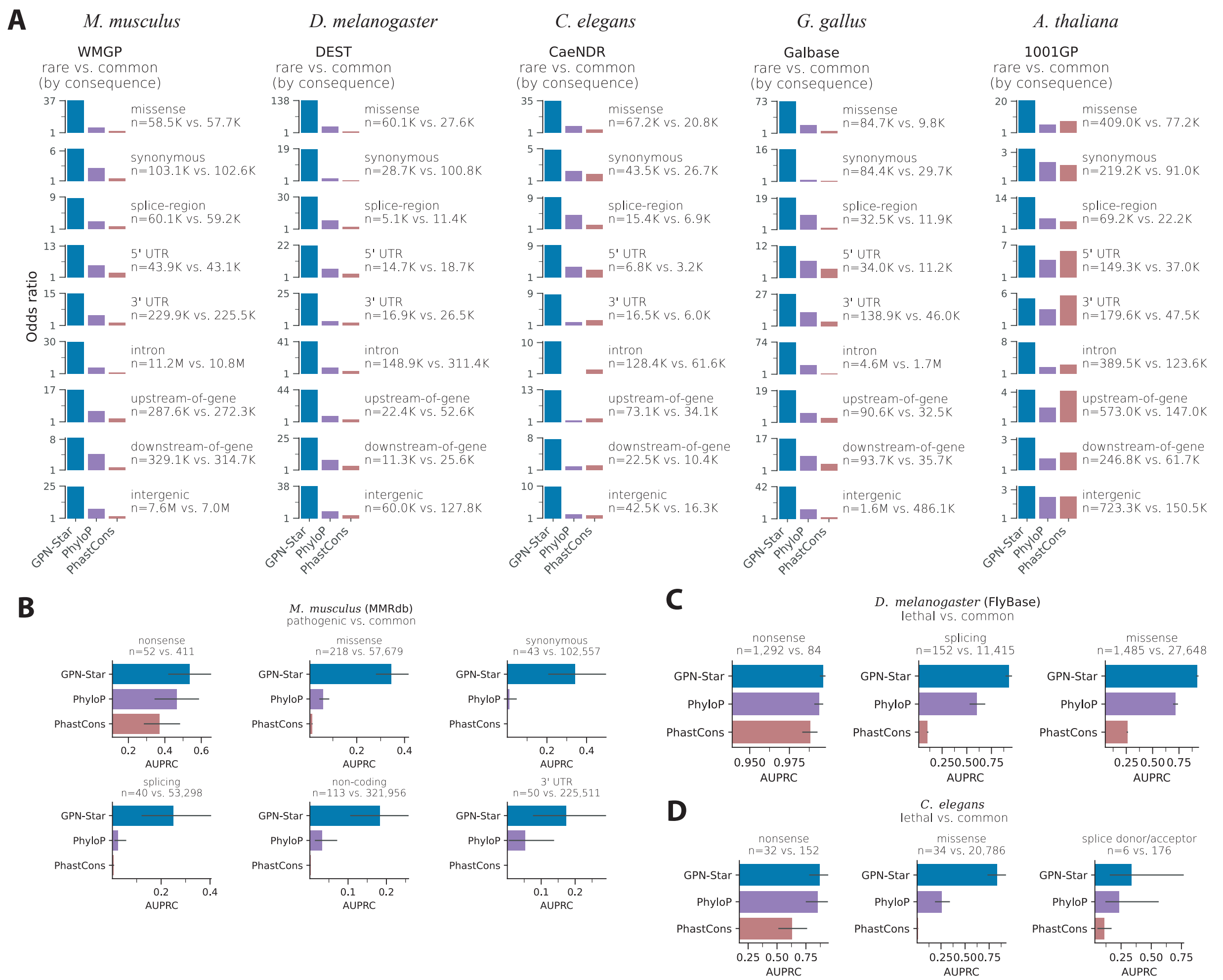

Supplementary Figure 16: Non-human model evaluations stratified by molecular consequences. (A) Enrichment of rare versus common variants from population genome databases in the tail of deleterious scores (the threshold was chosen such that each score yielded 30 false discoveries), compared with PhyloP and PhastCons fitted to the same alignments. (B) Classification of MMRdb pathogenic variants versus WMGP common variants in *M. musculus*. (C) Classification of FlyBase lethal variants versus DEST common variants in *D. melanogaster*. (D) Classification of *C. elegans* lethal variants versus CaeNDR common variants.

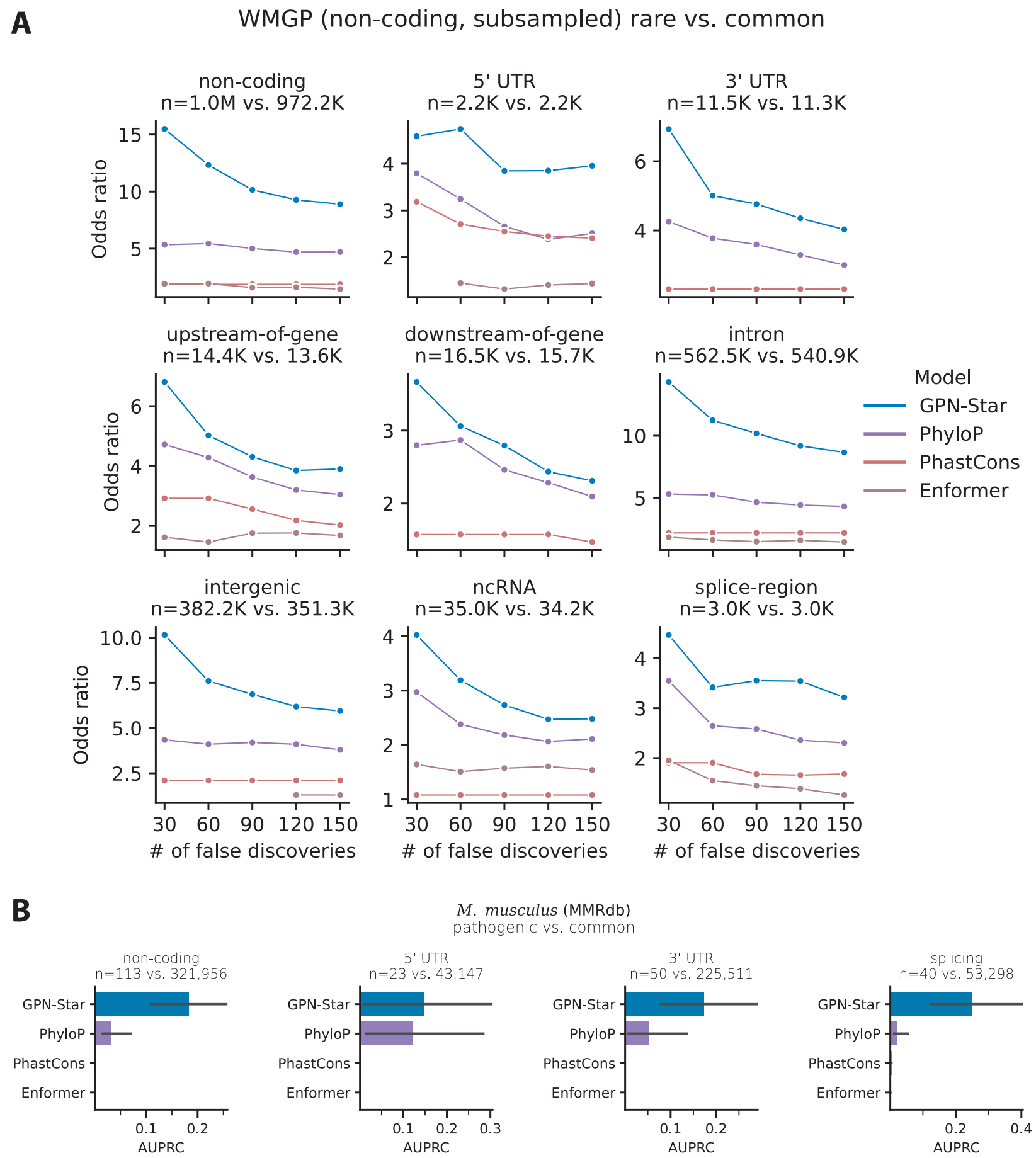

Supplementary Figure 17: Evaluation of Enformer on the non-coding variants in mouse benchmarks. (A) Enrichment of rare vs. common variants in the deleterious tails of model predictions, varying the threshold from 30 to 150 false discoveries. Data points are absent when enrichment is not significant by Fisher's exact test. Only non-coding categories were retained, and the variants are subsampled to 5% to reduce computational burden. (B) AUPRC of classifying non-coding pathogenic MMRdb variants from WMGP common variants.

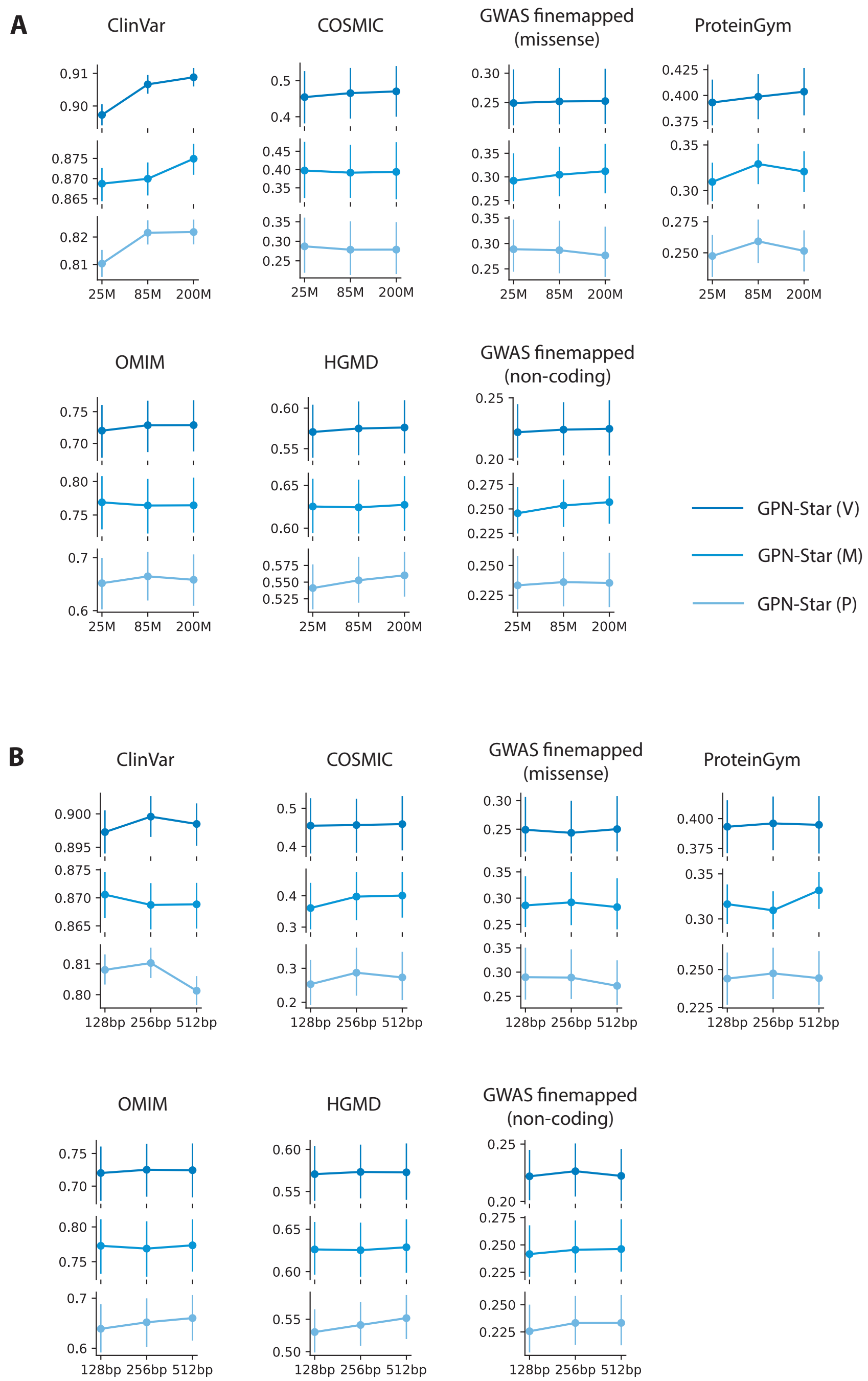

Supplementary Figure 18: Scaling experiments on model size and context size. (A) Performance of GPN-Star models with 25 M, 85 M, and 200 M parameters across benchmarks. (B) Performance of GPN-Star models with 25 M parameters and 128bp, 256bp, and 512bp context sizes across benchmarks. The benchmarks are the same as in Figure 2. For all benchmarks except ProteinGym, the performance metric is the area under precision recall curve (AUPRC) and the error bars represent the 95% confidence intervals based on 1,000 class-stratified bootstrap resamples. For ProteinGym, the dots and error bars represent the mean and standard deviation of Spearman correlations across the DMS assays.

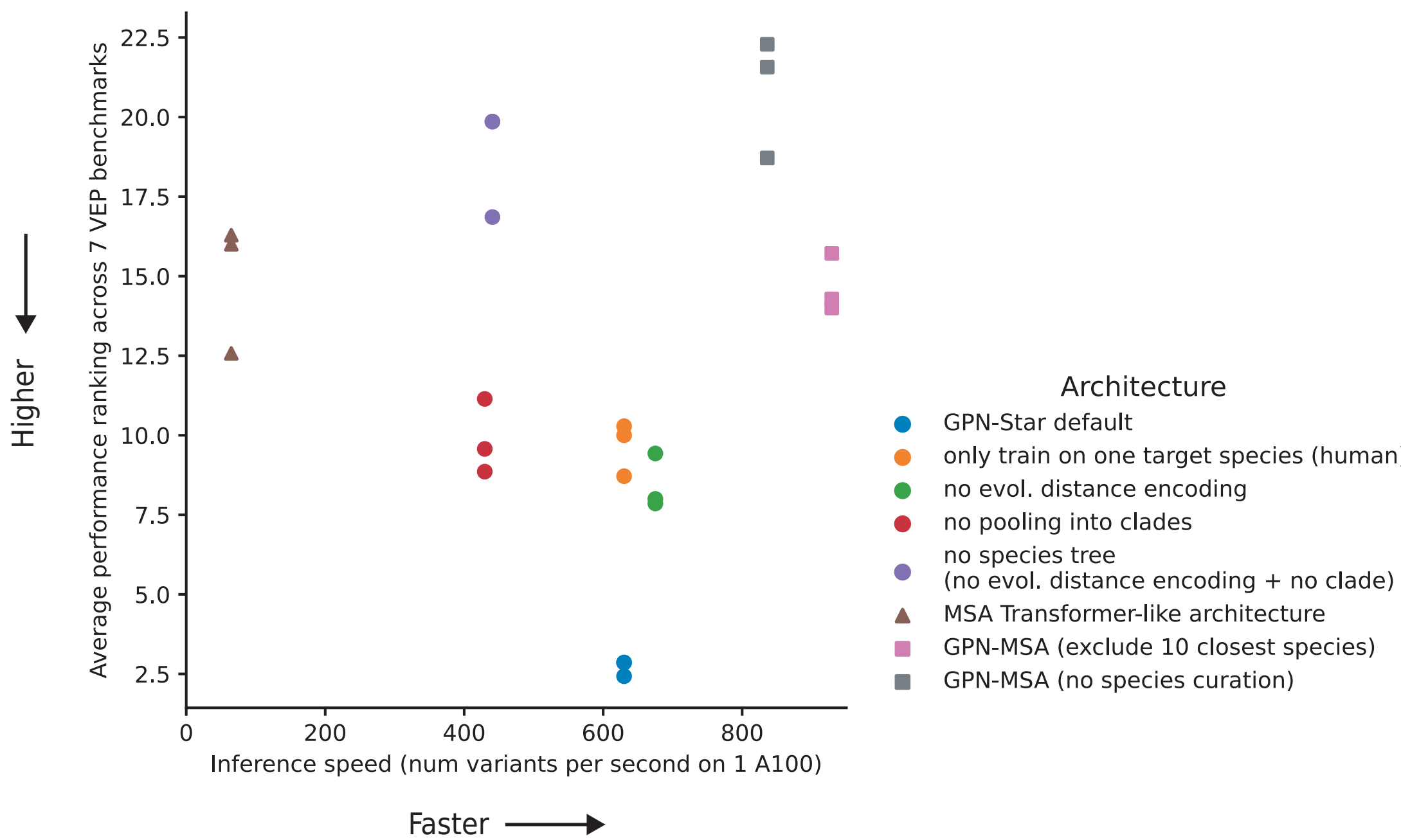

Supplementary Figure 19: Ablation studies. Each set of three dots with the same color represents three random initializations with the same architecture. The average performance ranking is among the models with these 24 architecture-seed combinations. All compared models were trained with the same dataset (multiz100way with training interval curation) and hyperparameters, including model size (25 million), learning rate (1e-4), batch size (512), and number of steps (150k). The MSA Transformer-like architecture neither uses species trees nor distinguishes target and source species. In each encoder block, row-wise self-attention is followed by column-wise self-attention operating on the full alignment.

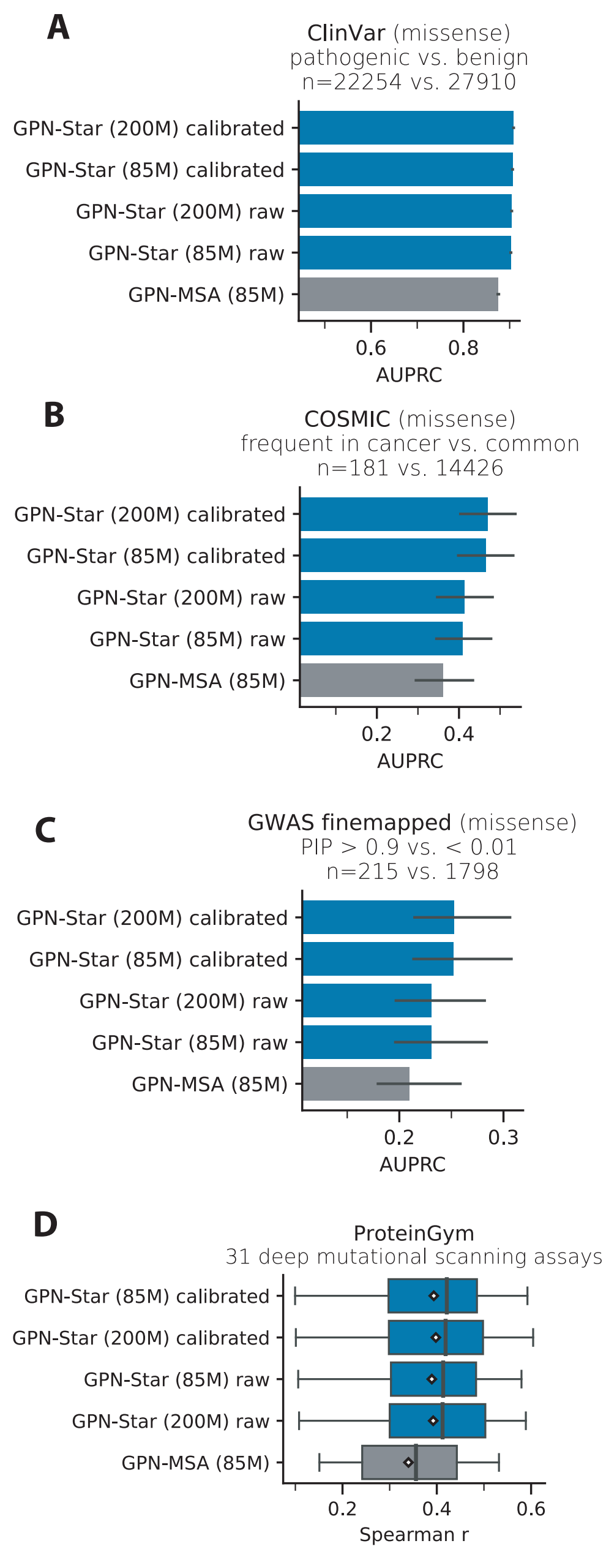

Supplementary Figure 20: Head-to-head comparison with GPN-MSA on variant effect prediction. The benchmarks are the same as in Figure 2. For fair comparison, we showed the performance of GPN-Star with the same alignment (multiz100way), same model size (85 M), and the uncalibrated raw scores. The error bars in the barplots represent the 95% confidence intervals from 1,000 class-stratified bootstrap resamples.

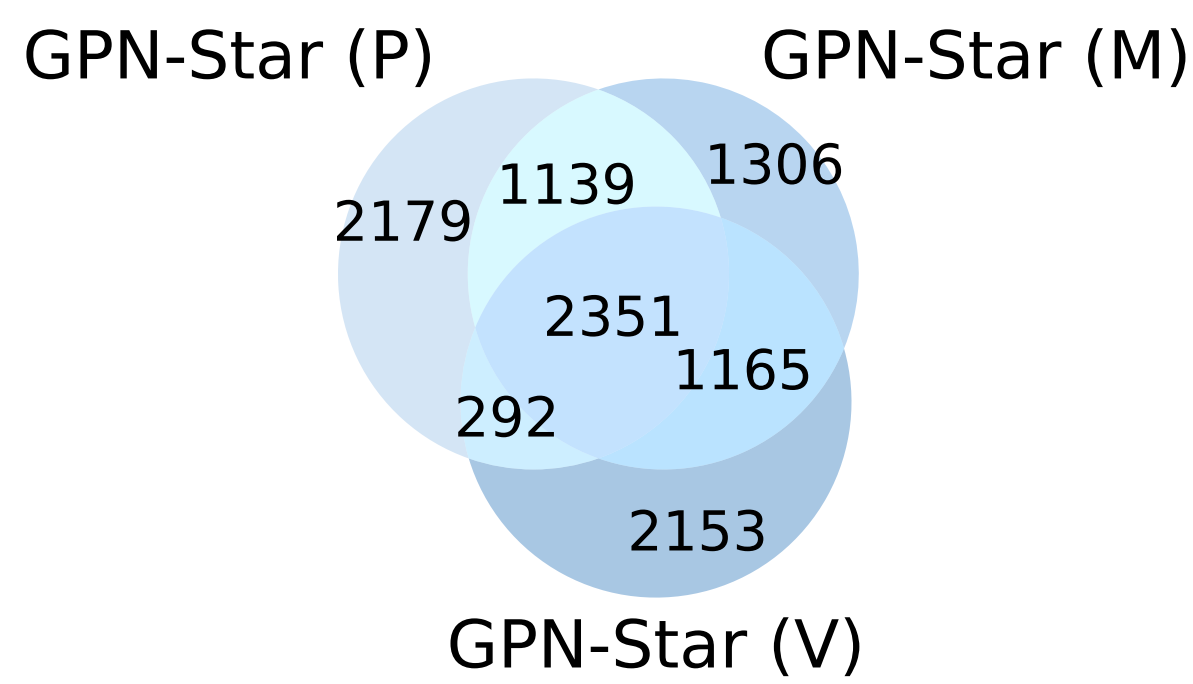

Supplementary Figure 21: Overlap across the three GPN-Star models for the top 0.1% most constrained common variants according to each model.

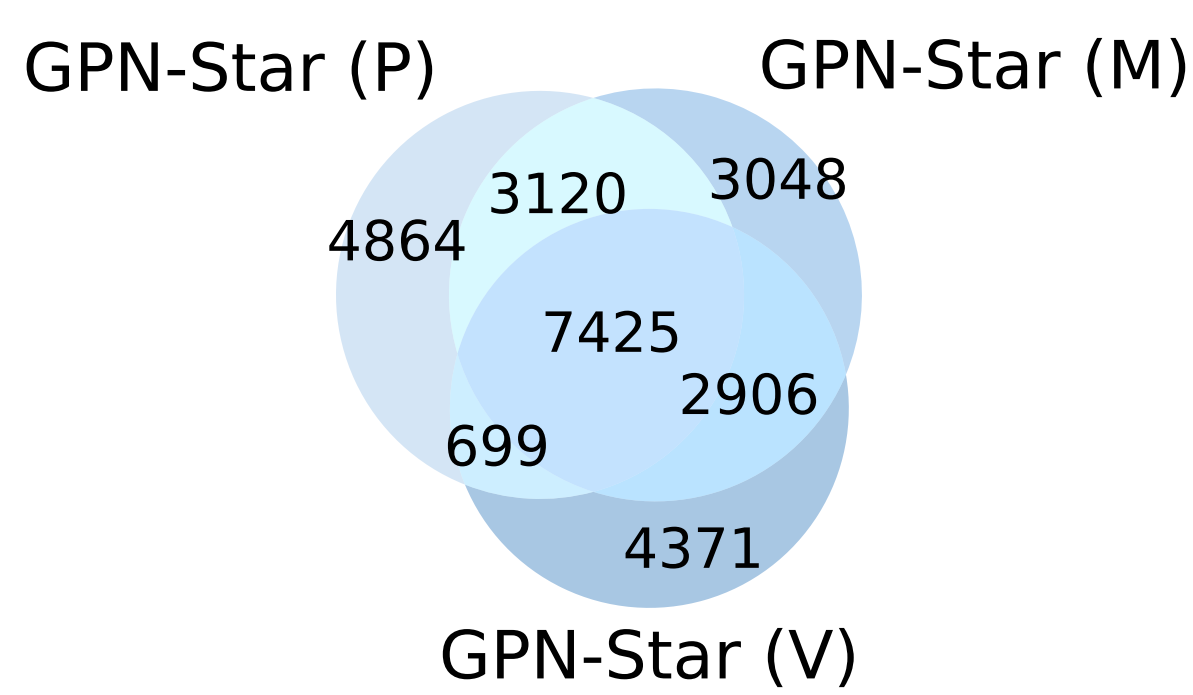

Supplementary Figure 22: Overlap across the three GPN-Star models for highly constrained variants (all S-LDSC variants, including common and low-frequency) with scores more extreme than the top 0.1% threshold (the 0.999 quantile) of common variants according to each model.

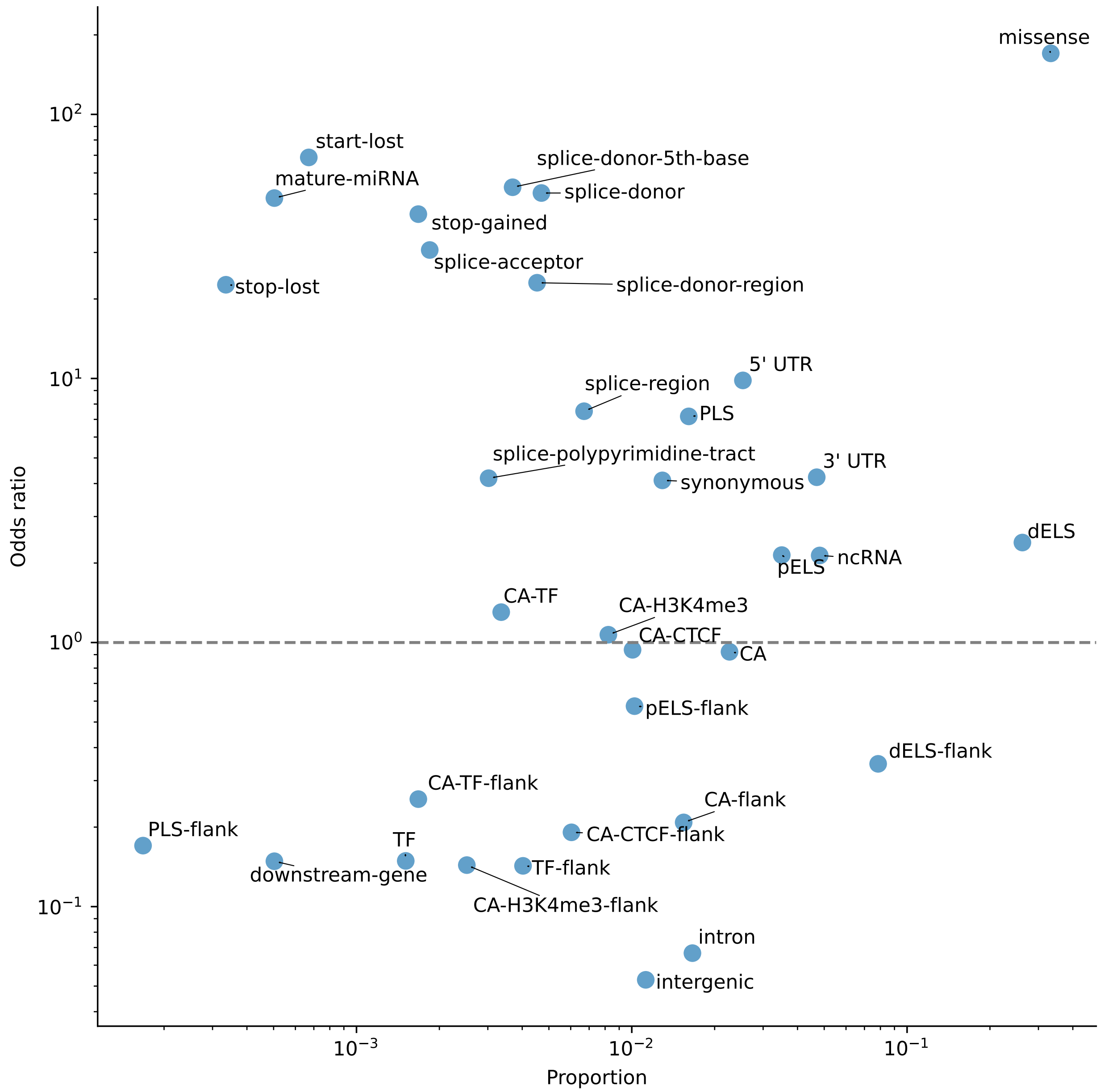

Supplementary Figure 23: Proportion of variant consequence among the top 0.1% most constrained common variants according to GPN-Star (P) and odds ratio with respect to the 99.9% least constrained variants.

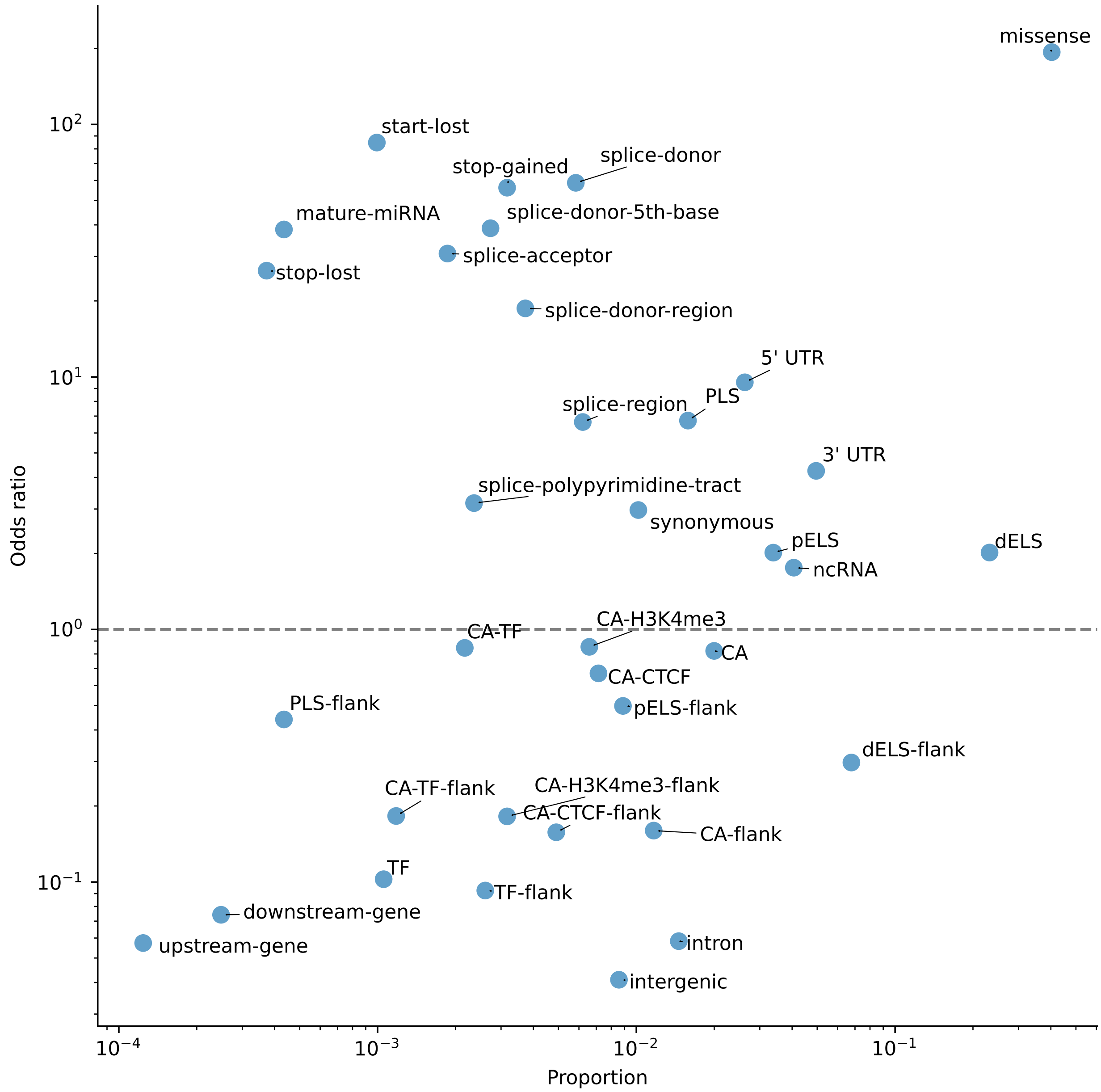

Supplementary Figure 24: Proportion of variant consequence among the highly constrained variants (all S-LDSC variants, common and low-frequency) with scores within the top 0.1% threshold of constrained common variants according to GPN-Star (P) and odds ratio with respect to the rest of the variants.

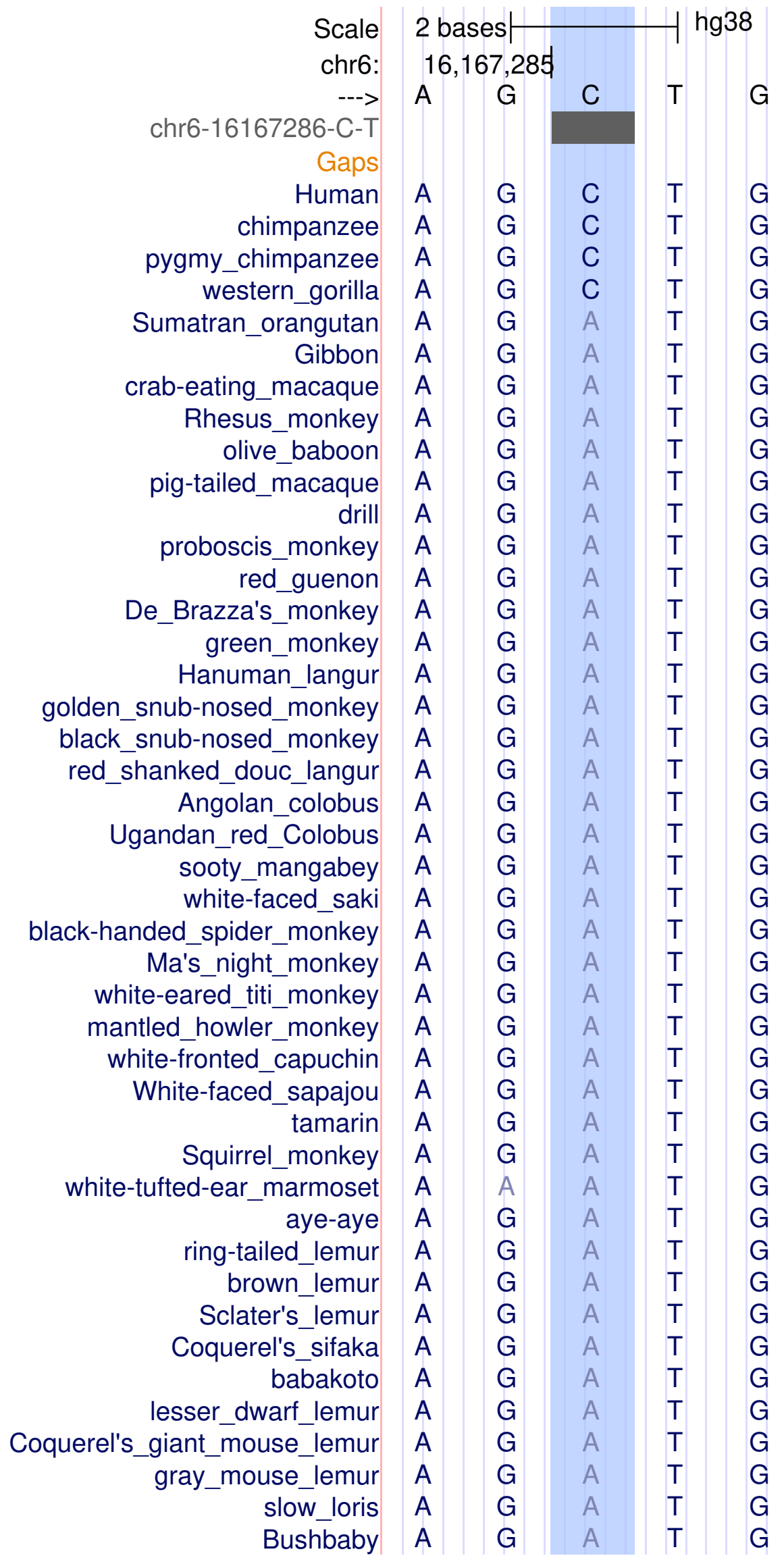

Supplementary Figure 25: Example putative causal variant (rs149514089, `chr6-16167286-C-T`) where entropy is more predictive than LLR. The predicted entropy is very low but neither the reference (C) or alternate (T) allele has a high probability.

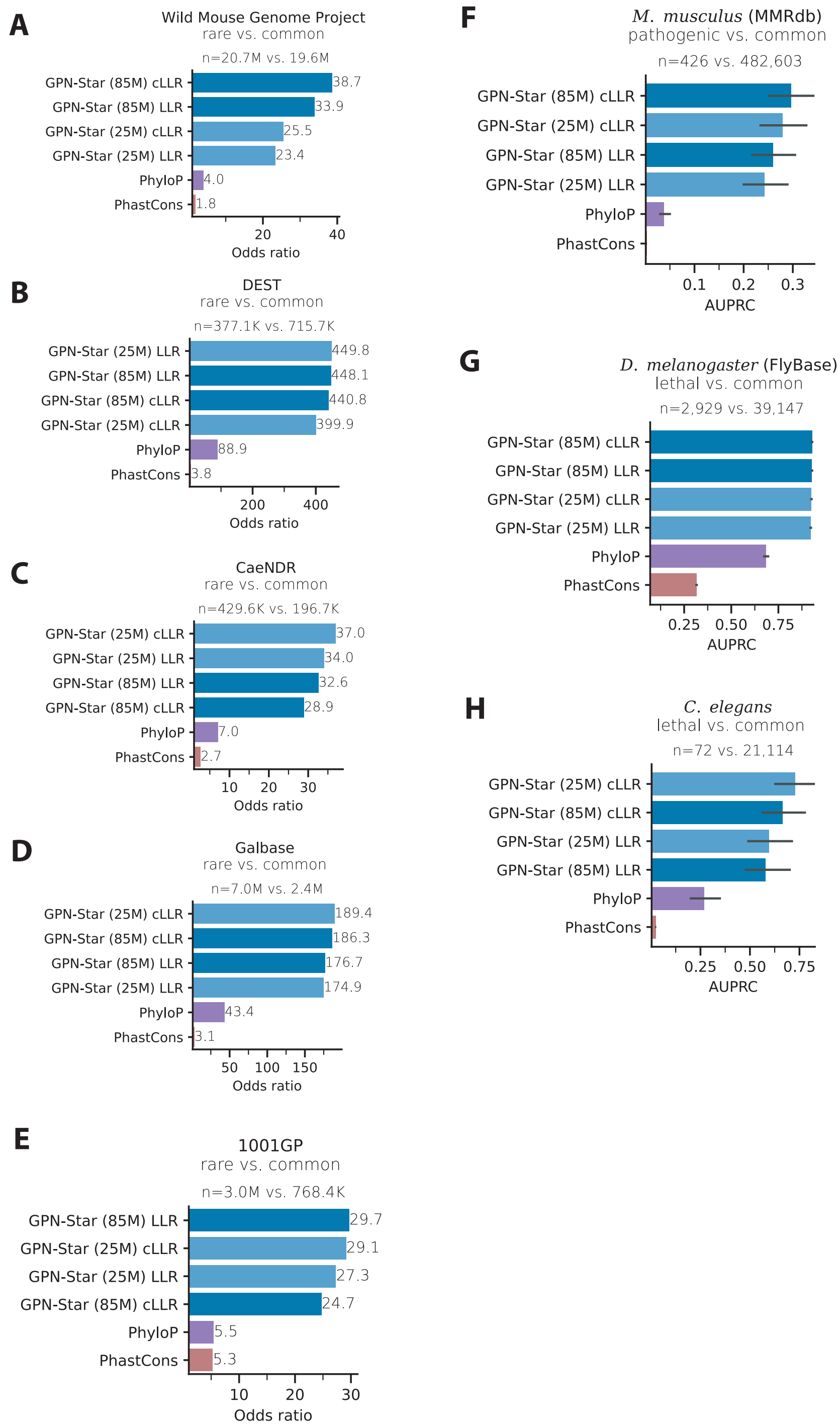

Supplementary Figure 26: Non-human model evaluations before and after mutation rate calibration. (A)-(E) Enrichment of rare versus common variants from population genome databases in the tail of deleterious scores (the threshold was chosen such that each score yielded 30 false discoveries), compared with PhyloP and PhastCons fitted to the same alignments. (F) Classification of MMRdb pathogenic variants versus WMGP common variants in *M. musculus*. (G) Classification of FlyBase lethal variants versus DEST common variants in *D. melanogaster*. (H) Classification of *C. elegans* lethal variants versus CaeNDR common variants. cLLR: calibrated log-likelihood ratio (see Supplementary Methods).

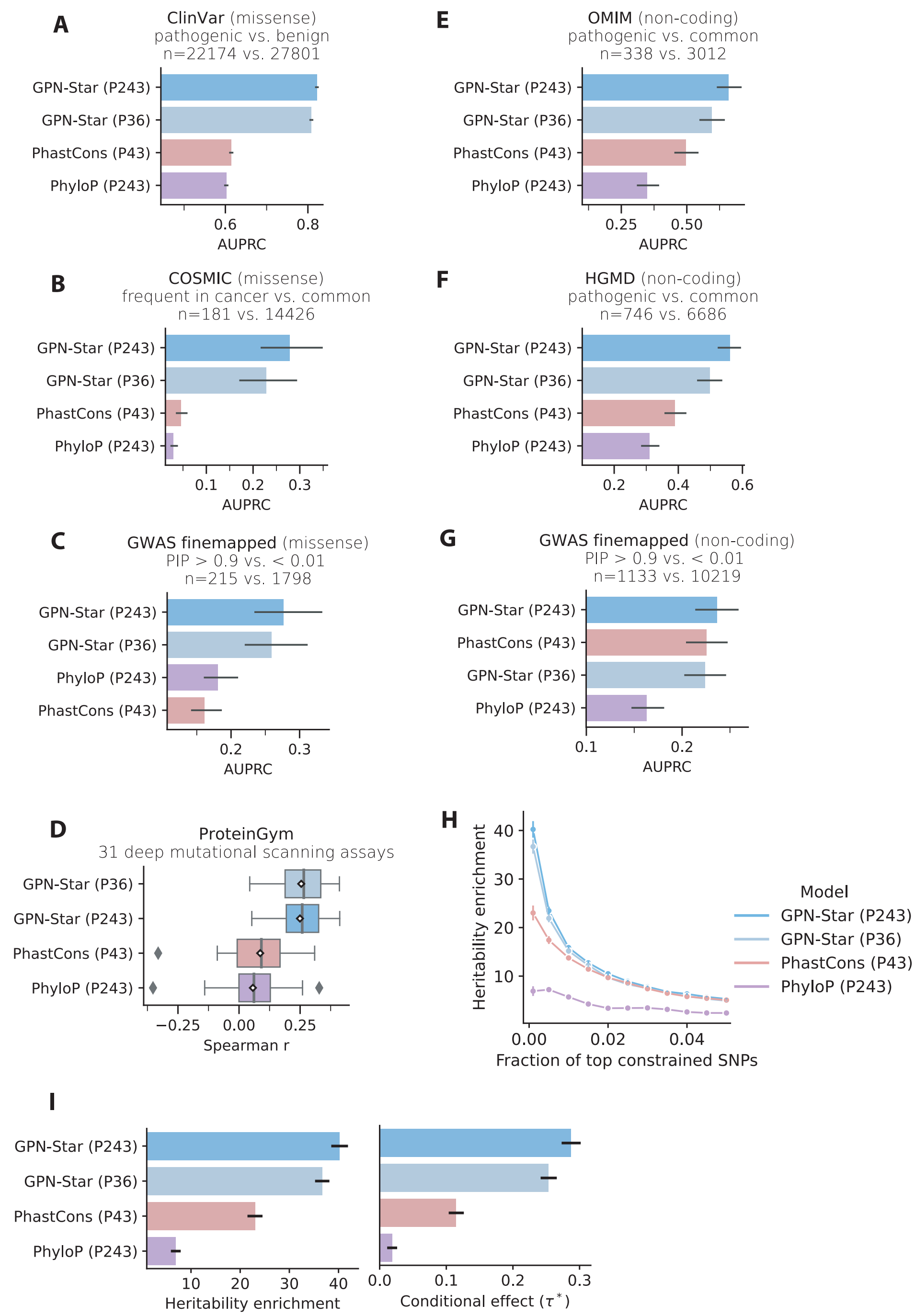

Supplementary Figure 27: Comparison of the primate models trained with WGAs of different numbers of species. The benchmarks include the variant effect prediction tasks in Figure 2 ((A)-(G)) and complex trait heritability analysis in Figure 3 ((H)-(I)). The numbers in the parentheses in the model names represent the number of primate species in the alignment: P243 denotes the 243 species in the full cactus447way alignment; P43 denotes the 43 species in the cactus241way alignment from the initial Zoonomia release; P36 denotes 36 species in the intersection of the two sets above.

Supplementary References

67. Dyer, S. C. *et al.* Ensembl 2025. *Nucleic Acids Research* **53,** D948–D957 (2025).
68. Moore, J. E. *et al.* An Expanded Registry of Candidate cis-Regulatory Elements for Studying Transcriptional Regulation. *bioRxiv* (2024).
69. Miles, A. *et al.* *zarr-developers/zarr-python: v3.0.7* version v3.0.7. Apr. 2025. https://doi.org/10.5281/zenodo.15255977.
70. Kent, W. J. *et al.* The human genome browser at UCSC. *Genome Research* **12,** 996–1006 (2002).
71. Zoonomia Consortium. A comparative genomics multitool for scientific discovery and conservation. *Nature* **587,** 240–245 (2020).
72. Albors, C., Li, J. C., Benegas, G., Ye, C. & Song, Y. S. *A Phylogenetic Approach to Genomic Language Modeling* in *Research in Computational Molecular Biology* (ed Sankararaman, S.) (Springer Nature Switzerland, Cham, 2025), 99–117. ISBN: 978-3-031-90252-9.
73. Tian, F., Yang, D.-C., Meng, Y.-Q., Jin, J. & Gao, G. PlantRegMap: charting functional regulatory maps in plants. *Nucleic Acids Research* **48,** D1104–D1113 (2020).
74. Hubisz, M. J., Pollard, K. S. & Siepel, A. PHAST and RPHAST: phylogenetic analysis with space/time models. *Briefings in Bioinformatics* **12,** 41–51 (2011).
75. Su, J. *et al.* Roformer: Enhanced transformer with rotary position embedding. *arXiv preprint arXiv:2104.09864* (2021).
76. Li, S. *et al.* *Functional Interpolation for Relative Positions improves Long Context Transformers* in *The Twelfth International Conference on Learning Representations* (2024). https://openreview.net/forum?id=rR03qFesqk.
77. Tarailo-Graovac, M. & Chen, N. Using RepeatMasker to Identify Repetitive Elements in Genomic Sequences. *Current Protocols in Bioinformatics* **25,** 4.10.1–4.10.14 (2009).
78. Morgulis, A., Gertz, E. M., Schäffer, A. A. & Agarwala, R. A fast and symmetric DUST implementation to mask low-complexity DNA sequences. *Journal of Computational Biology* **13,** 1028–1040 (2006).
79. Aggarwala, V. & Voight, B. F. An expanded sequence context model broadly explains variability in polymorphism levels across the human genome. *Nature Genetics* **48,** 349–355 (2016).
80. Visel, A., Minovitsky, S., Dubchak, I. & Pennacchio, L. A. VISTA Enhancer Browser—a database of tissue-specific human enhancers. *Nucleic acids research* **35,** D88–D92 (2007).
81. Kosicki, M. *et al.* VISTA Enhancer browser: an updated database of tissue-specific developmental enhancers. *Nucleic Acids Research* **53,** D324–D330 (2025).
82. Open2C *et al.* Bioframe: operations on genomic intervals in Pandas dataframes. *Bioinformatics* **40,** btae088 (2024).
83. McInnes, L., Healy, J. & Melville, J. UMAP: Uniform manifold approximation and projection for dimension reduction. *arXiv preprint arXiv:1802.03426* (2018).
84. Pedregosa, F. *et al.* Scikit-learn: Machine Learning in Python. *Journal of Machine Learning Research* **12,** 2825–2830 (2011).

85. Schubach, M., Maass, T., Nazaretyan, L., Röner, S. & Kircher, M. CADD v1.7: using protein language models, regulatory CNNs and other nucleotide-level scores to improve genome-wide variant predictions. *Nucleic Acids Research* **52,** D1143–D1154 (2024).

86. Simon, D. *et al.* A mutation in the 3′-UTR of the HDAC6 gene abolishing the post-transcriptional regulation mediated by hsa-miR-433 is linked to a new form of dominant X-linked chondrodysplasia. *Human molecular genetics* **19,** 2015–2027 (2010).

87. Tidwell, T. *et al.* Neutropenia-associated ELANE mutations disrupting translation initiation produce novel neutrophil elastase isoforms. *Blood, The Journal of the American Society of Hematology* **123,** 562–569 (2014).

88. Pollak, E. S., Hung, H.-L., Godin, W., Overton, G. C. & High, K. A. Functional characterization of the human factor VII 5′-flanking region (∗). *Journal of Biological Chemistry* **271,** 1738–1747 (1996).

89. Smedley, D. *et al.* A whole-genome analysis framework for effective identification of pathogenic regulatory variants in Mendelian disease. *The American Journal of Human Genetics* **99,** 595–606 (2016).

90. McLaren, W. *et al.* The Ensembl Variant Effect Predictor. *Genome Biology* **17,** 1–14 (2016).

91. Chen, K. M., Wong, A. K., Troyanskaya, O. G. & Zhou, J. A sequence-based global map of regulatory activity for deciphering human genetics. *Nature Genetics* **54,** 940–949 (2022).

92. Bycroft, C. *et al.* The UK Biobank resource with deep phenotyping and genomic data. *Nature* **562,** 203–209 (2018).

93. Finucane, H. K. *et al.* Variant scoring performance across selection regimes depends on variant-to-gene and gene-to-disease components. *bioRxiv,* 2024–09 (2024).

94. Srivastava, D. *et al.* Borzoi-informed fine mapping improves causal variant prioritization in complex trait GWAS. *bioRxiv,* 2025–07 (2025).

95. Brandes, N., Goldman, G., Wang, C. H., Ye, C. J. & Ntranos, V. Genome-wide prediction of disease variant effects with a deep protein language model. *Nature Genetics* **55,** 1512–1522 (2023).

96. Halligan, D. L. *et al.* Contributions of protein-coding and regulatory change to adaptive molecular evolution in murid rodents. *PLoS genetics* **9,** e1003995 (2013).

97. Davies, R. W. *Factors influencing genetic variation in wild mice* PhD thesis (University of Oxford, 2015).

98. Harr, B. *et al.* Genomic resources for wild populations of the house mouse, Mus musculus and its close relative Mus spretus. *Scientific Data* **3,** 1–14 (2016).

99. Phifer-Rixey, M. *et al.* The genomic basis of environmental adaptation in house mice. *PLoS Genetics* **14,** e1007672 (2018).

100. Payseur, B. A. & Jing, P. Genomic targets of positive selection in giant mice from Gough Island. *Molecular Biology and Evolution* **38,** 911–926 (2021).

101. Fujiwara, K. *et al.* Insights into Mus musculus population structure across Eurasia revealed by whole-genome analysis. *Genome biology and evolution* **14,** evac068 (2022).

102. Morgan, A. P. *et al.* Population structure and inbreeding in wild house mice (Mus musculus) at different geographic scales. *Heredity* **129,** 183–194 (2022).

103. Lawal, R. A. *et al.* Taxonomic assessment of two wild house mouse subspecies using whole-genome sequencing. *Scientific reports* **12,** 20866 (2022).

104. Chen, S., Zhou, Y., Chen, Y. & Gu, J. fastp: an ultra-fast all-in-one FASTQ preprocessor. *Bioinformatics* **34,** i884–i890 (2018).

105. Hickey, G. *et al.* Pangenome graph construction from genome alignments with Minigraph-Cactus. *Nature biotechnology* **42,** 663–673 (2024).

106. Ferraj, A. *et al.* Resolution of structural variation in diverse mouse genomes reveals chromatin remodeling due to transposable elements. *Cell Genomics* **3** (2023).

107. Sirén, J. *et al.* Pangenomics enables genotyping of known structural variants in 5202 diverse genomes. *Science* **374,** abg8871 (2021).

108. Garrison, E. *et al.* Variation graph toolkit improves read mapping by representing genetic variation in the reference. *Nature biotechnology* **36,** 875–879 (2018).

109. Poplin, R. *et al.* A universal SNP and small-indel variant caller using deep neural networks. *Nature biotechnology* **36,** 983–987 (2018).

110. Lin, M. F. *et al.* GLnexus: joint variant calling for large cohort sequencing. *bioRxiv,* 343970. https://doi.org/10.1101/343970 (2018).

111. Fairfield, H. *et al.* Mutation discovery in mice by whole exome sequencing. *Genome Biology* **12,** 1–12 (2011).

112. Fairfield, H. *et al.* Exome sequencing reveals pathogenic mutations in 91 strains of mice with Mendelian disorders. *Genome research* **25,** 948–957 (2015).

113. Perez, G. *et al.* The UCSC Genome Browser database: 2025 update. *Nucleic Acids Research* **53,** D1243–D1249 (2025).

114. Nunez, J. C. *et al.* Footprints of worldwide adaptation in structured populations of D. melanogaster through the expanded DEST 2.0 genomic resource. *bioRxiv,* 2024–11 (2024).

115. Crombie, T. A. *et al.* CaeNDR, the Caenorhabditis natural diversity resource. *Nucleic Acids Research* **52,** D850–D858 (2024).

116. Fu, W. *et al.* Galbase: a comprehensive repository for integrating chicken multi-omics data. *BMC genomics* **23,** 364 (2022).

117. Alonso-Blanco, C. *et al.* 1,135 genomes reveal the global pattern of polymorphism in *Arabidopsis thaliana.* *Cell* **166,** 481–491 (2016).

118. Gazal, S. *et al.* Linkage disequilibrium–dependent architecture of human complex traits shows action of negative selection. *Nature Genetics* **49,** 1421–1427 (2017).

119. 1000 Genomes Project Consortium *et al.* A global reference for human genetic variation. *Nature* **526,** 68–74 (2015).

120. Lonsdale, J. *et al.* The genotype-tissue expression (GTEx) project. *Nature Genetics* **45,** 580–585 (2013).
