# Supplementary material for: Predicting genome-wide functional constraints with GPN-Star
Source: Nature. 2026 Sep 9;659(8139):141–50. doi: 10.1038/s41586-026-11005-5 (PMC13736532; doi:10.1038/s41586-026-11005-5)
Supplement: Supplementary file 2 [file 41586_2026_11005_MOESM2_ESM.pdf]

nature portfolio

Corresponding author(s): Yun S. Song

Last updated by author(s): Jul 31, 2026

Reporting Summary

Nature Portfolio wishes to improve the reproducibility of the work that we publish. This form provides structure for consistency and transparency in reporting. For further information on Nature Portfolio policies, see our Editorial Policies and the Editorial Policy Checklist.

Statistics

For all statistical analyses, confirm that the following items are present in the figure legend, table legend, main text, or Methods section.

n/a	Confirmed	
☐	☒	The exact sample size (n) for each experimental group/condition, given as a discrete number and unit of measurement
☒	☐	A statement on whether measurements were taken from distinct samples or whether the same sample was measured repeatedly
☐	☒	The statistical test(s) used AND whether they are one- or two-sided<br>*Only common tests should be described solely by name; describe more complex techniques in the Methods section.*
☒	☐	A description of all covariates tested
☐	☒	A description of any assumptions or corrections, such as tests of normality and adjustment for multiple comparisons
☐	☒	A full description of the statistical parameters including central tendency (e.g. means) or other basic estimates (e.g. regression coefficient) AND variation (e.g. standard deviation) or associated estimates of uncertainty (e.g. confidence intervals)
☐	☒	For null hypothesis testing, the test statistic (e.g. F, t, r) with confidence intervals, effect sizes, degrees of freedom and P value noted<br>*Give P values as exact values whenever suitable.*
☒	☐	For Bayesian analysis, information on the choice of priors and Markov chain Monte Carlo settings
☒	☐	For hierarchical and complex designs, identification of the appropriate level for tests and full reporting of outcomes
☐	☒	Estimates of effect sizes (e.g. Cohen's d, Pearson's r), indicating how they were calculated

Our web collection on statistics for biologists contains articles on many of the points above.

Software and code

Policy information about availability of computer code

Data collection	No software was used for data collection. This study is entirely computational and generated no new experimental measurements; all data analyzed are pre-existing and were obtained from the public repositories listed in the Data availability statement.
Data analysis	Code for training GPN-Star, running inference and reproducing all analyses: https://github.com/songlab-cal/gpn (v0.8), archived at Zenodo (https://doi.org/10.5281/zenodo.21501177), available under the MIT license.<br>Other analysis software: Python 3.10 (https://www.python.org); PyTorch 2.2.0 (https://pytorch.org); Hugging Face Transformers 4.48.1 (https://github.com/huggingface/transformers); Hugging Face Datasets 2.18.0 (https://github.com/huggingface/datasets); NumPy 1.26.4 (https://numpy.org); pandas 2.2.3 (https://pandas.pydata.org); Zarr 2.18.3 (https://zarr.readthedocs.io); SciPy 1.10.0 (https://scipy.org); scikit-learn 1.2.0 (https://scikit-learn.org); statsmodels 0.13.5 (https://www.statsmodels.org); Matplotlib 3.6.2 (https://matplotlib.org); seaborn 0.12.2 (https://seaborn.pydata.org); PHAST 1.5, comprising phyloFit, phyloP and phastCons (http://compgen.cshl.edu/phast/); liftover 1.2.2 (https://pypi.org/project/liftover/); Ensembl VEP release 107, GRCh38 (https://www.ensembl.org/vep); LDSC 1.0.0 with baselineLD v2.2 annotations (https://github.com/bulik/ldsc); DeepRVAT 1.1 (https://github.com/PMBio/deeprvat); and the competing variant effect predictors evaluated for comparison, used as released models or precomputed scores: CADD v1.7 (https://cadd.gs.washington.edu), Evo 2 (https://github.com/ArcInstitute/evo2), Nucleotide Transformer v1 2.5B multispecies (https://github.com/instadeepai/nucleotide-transformer), ESM-1b and ESM-2 (https://github.com/facebookresearch/esm), ESM-3-open (https://huggingface.co/biohub/esm3-sm-open-v1), Enformer (https://github.com/google-deepmind/deepmind-research/tree/master/enformer), Borzoi (https://github.com/calico/borzoi), AlphaGenome (https://github.com/google-deepmind/alphagenome), GPN-MSA (https://github.com/songlab-cal/gpn), AlphaMissense (https://github.com/google-deepmind/alphamissense), PrimateAI-3D (https://github.com/Illumina/PrimateAI-3D), and Roulette mutation rate estimates.

For manuscripts utilizing custom algorithms or software that are central to the research but not yet described in published literature, software must be made available to editors and reviewers. We strongly encourage code deposition in a community repository (e.g. GitHub). See the Nature Portfolio guidelines for submitting code & software for further information.

Data

Policy information about availability of data

All manuscripts must include a data availability statement. This statement should provide the following information, where applicable:

- Accession codes, unique identifiers, or web links for publicly available datasets
- A description of any restrictions on data availability
- For clinical datasets or third party data, please ensure that the statement adheres to our policy

The pretrained models, training datasets and benchmark datasets are available on Hugging Face (https://huggingface.co/collections/songlab/gpn-star-68c0c055acc2ee51d5c4f129). Precomputed GPN-Star predictions for all possible single-nucleotide variants in the human genome and the five model organisms are available on Hugging Face (https://doi.org/10.57967/hf/9849). The source code is archived at Zenodo (https://doi.org/10.5281/zenodo.21501177).
All data sources used in this study are pre-existing and publicly available; their versions, accessions and URLs are listed in the Supplementary Methods. No new experimental data were generated in this study that require deposition.

Research involving human participants, their data, or biological material

Policy information about studies with human participants or human data. See also policy information about sex, gender (identity/presentation), and sexual orientation and race, ethnicity and racism.

Reporting on sex and gender	n/a
Reporting on race, ethnicity, or other socially relevant groupings	n/a
Population characteristics	n/a
Recruitment	n/a
Ethics oversight	n/a

Note that full information on the approval of the study protocol must also be provided in the manuscript.

Field-specific reporting

Please select the one below that is the best fit for your research. If you are not sure, read the appropriate sections before making your selection.

☒ Life sciences ☐ Behavioural & social sciences ☐ Ecological, evolutionary & environmental sciences

For a reference copy of the document with all sections, see nature.com/documents/nr-reporting-summary-flat.pdf

Life sciences study design

All studies must disclose on these points even when the disclosure is negative.

Sample size	No statistical method was used to predetermine sample size and no power calculation was performed, because no sampling was carried out by the investigators: each analysis uses the complete, externally defined dataset for that resource after the pre-specified processing described in Methods.
Data exclusions	No data were excluded other than through pre-specified, dataset-level inclusion criteria described in Methods, all of which were defined before model predictions were examined. These comprise: restriction of ProteinGym to the 31 deep mutational scanning assays on human proteins; restriction of the MMRdb mouse benchmark to single-nucleotide variants whose reference allele matches GRCm39 after liftOver and to consequence categories with sufficient variant counts (missense, synonymous, nonsense, 3' UTR, 5' UTR and splicing); restriction of the FlyBase benchmark to missense, nonsense and splicing variants, which account for more than 99% of the annotated lethal variants. In each benchmark, the comparison is additionally restricted to variants that can be scored by all competing models. No variants, traits, assays or individuals were excluded on the basis of model performance or of any result.
Replication	No wet-laboratory experiments were performed, so biological replication does not apply; reproducibility was addressed in three ways. (1) Stochastic training. For example, the DeepRVAT analysis was run 3 times with independent random initializations, all plotted individually in Fig. 2j, and the conclusion held in every run; the architecture ablations likewise used 3 independent initializations per configuration. (2) Held-out evaluation: models were trained leave-one-chromosome-out, and the allele-frequency and mutation-rate analyses use chromosome 22, never seen in training; training is self-supervised and uses no benchmark labels, so all benchmarks are held out by construction. (3) Independent benchmarks: the central claim was tested on multiple mutually independent human benchmarks and in five non-human species and reproduced in each. All remaining analyses are deterministic given the released weights and data. All attempts at replication were successful.
Randomization	Randomization does not apply: no experimental units were allocated to groups by the investigators. Group membership in every benchmark is a fixed label assigned by an external database before this study (e.g. ClinVar pathogenic vs benign; fine-mapped PIP > 0.9 vs < 0.01).

Randomness enters only as computational procedure - assignment of chromosomes to training, validation and test sets; random sampling of non-conserved training windows; random tie-breaking when PhastCons scores saturate; random down-sampling of rare variants to match common-variant counts; random initialization (3 seeds); and 1,000 bootstrap resamples.

Blinding

Blinding does not apply and no step was susceptible to investigator expectation. Predictions come from a model trained purely by self-supervision on genome alignments that never observes benchmark labels; labels were assigned independently by third-party databases before this study; and all metrics (AUPRC, Spearman's rho, odds ratio, heritability enrichment and tau*) are computed automatically from fixed labels with no manual or subjective scoring.

Reporting for specific materials, systems and methods

We require information from authors about some types of materials, experimental systems and methods used in many studies. Here, indicate whether each material, system or method listed is relevant to your study. If you are not sure if a list item applies to your research, read the appropriate section before selecting a response.

Materials & experimental systems

n/a	Involved in the study
☒	☐ Antibodies
☒	☐ Eukaryotic cell lines
☒	☐ Palaeontology and archaeology
☒	☐ Animals and other organisms
☒	☐ Clinical data
☒	☐ Dual use research of concern
☒	☐ Plants

Methods

n/a	Involved in the study
☒	☐ ChIP-seq
☒	☐ Flow cytometry
☒	☐ MRI-based neuroimaging

Plants

Seed stocks

Report on the source of all seed stocks or other plant material used. If applicable, state the seed stock centre and catalogue number. If plant specimens were collected from the field, describe the collection location, date and sampling procedures.

Novel plant genotypes

Describe the methods by which all novel plant genotypes were produced. This includes those generated by transgenic approaches, gene editing, chemical/radiation-based mutagenesis and hybridization. For transgenic lines, describe the transformation method, the number of independent lines analyzed and the generation upon which experiments were performed. For gene-edited lines, describe the editor used, the endogenous sequence targeted for editing, the targeting guide RNA sequence (if applicable) and how the editor was applied.

Authentication

Describe any authentication procedures for each seed stock used or novel genotype generated. Describe any experiments used to assess the effect of a mutation and, where applicable, how potential secondary effects (e.g. second site T-DNA insertions, mosiacism, off-target gene editing) were examined.
