# Supplementary material for: Predicting genome-wide functional constraints with GPN-Star
Source: Nature. 2026 Sep 9;659(8139):141–50. doi: 10.1038/s41586-026-11005-5 (PMC13736532; doi:10.1038/s41586-026-11005-5)
Supplement: Supplementary file 3 [file 41586_2026_11005_MOESM3_ESM.pdf]

nature portfolio

Peer Review File

Predicting genome-wide functional constraints with GPN-Star

Corresponding Author: Professor Yun Song

Any redactions in this file are there to maintain patient confidentiality, the confidentiality of unpublished data, or to remove third-party material.

This file contains all reviewer reports in order by version, followed by all author rebuttals in order by version.

Version 1:

Reviewer comments:

Referee #2

(Remarks to the Author)
I co-reviewed this manuscript with one of the reviewers who provided the listed reports.

(Remarks on code availability)

Referee #3

(Remarks to the Author)
Summary

In this study, Ye et al. present GPN-Star, a new genomics foundation model that builds upon the authors' previous work on GPN-MSA. The model integrates evolutionary information from whole-genome alignments (WGAs) and species trees through a phylogeny-aware architecture. Trained across vertebrate, mammalian, and primate timescales, GPN-Star is evaluated on a wide range of variant-effect prediction (VEP) tasks and demonstrates state-of-the-art performance in both coding and non-coding regions of the human genome, as well as across several model organisms. The work aims to show that incorporating multispecies evolutionary context leads to more biologically grounded variant predictions.
Conceptually, this direction is valuable and timely—variant interpretation remains a central challenge in human genetics, and using multispecies alignments is biologically intuitive. However, many of the ideas presented here are extensions of prior work, particularly GPN-MSA, and the improvements appear incremental rather than conceptual breakthroughs. While the reported performance gains are impressive, it remains unclear how much of this improvement comes from architectural innovation versus larger model size or dataset differences. Furthermore, practical aspects such as model usability and fair baseline comparisons require more careful analysis. Overall, the paper represents an important but evolutionary (rather than revolutionary) step in alignment-based genomic modeling.

1 — Architecture and model analysis

The new model architecture is central to the manuscript, yet its components are not sufficiently analyzed or justified. The paper would benefit from a systematic exploration of what aspects of the design truly contribute to improved performance.

First, ablation studies are missing. The authors introduce several changes relative to GPN-MSA—such as the use of multiple input species, species-aware attention, and alignment-informed pooling—but do not quantify their individual contributions. Evaluating how each architectural component affects downstream variant-prediction performance would be critical to understanding why GPN-Star works.

Second, the decision to train three separate models (vertebrate, mammal, and primate) is not well justified. It complicates practical use and raises questions about whether a unified approach could capture the same signals. At a minimum, the authors should explain how a user would choose which model to apply to a given problem and whether a single model spanning multiple scales could perform comparably.

2 — Benchmarking and comparison to existing models

The benchmarking section is extensive, but several issues make interpretation difficult.
First, while the model is tested on standard variant-effect prediction benchmarks (ClinVar, COSMIC, GWAS fine-mapping, etc.), the analysis remains mostly limited to these established datasets. For a paper targeting Nature, it would be valuable to include either additional clinically relevant datasets or finer-grained analyses—for example, distinguishing model behavior across genomic contexts (intronic, exonic, promoter, enhancer, splicing, UTR). This would provide a deeper understanding of what types of variants benefit most from the evolutionary context.

Second, the baseline comparisons are not entirely fair or up to date. GPN-Star leverages WGAs, whereas most competing models (e.g., Nucleotide Transformer, Evo, Enformer) operate on single sequences. This gives GPN-Star access to substantially more information, which should be clearly acknowledged. The comparison to ESM-1b is particularly problematic—this protein model is now several years old, and more recent versions (ESM-2 and ESM-3) have significantly improved performance.The authors should also consider including MSA-Transformer as a protein baseline, since it likewise leverages multiple sequence alignments and would provide a more balanced comparison. Similarly, the authors should specify which Nucleotide Transformer model (v1 or v2) and size were used, as results vary widely across versions and scales.

The reported compute comparisons also need clarification. The paper contrasts training budgets between models of very different sizes and goals—Evo (designed primarily for generation), NT (for fine-tuning), and GPN-Star (for VEP). The claim that GPN-Star is more efficient should therefore be treated cautiously unless parameter counts and training data are normalized.
Overall, the benchmarking is impressive in scope, but the comparisons must be fair, transparent, and clearly documented.

3 — Tissue specificity

The tissue-specific analyses are currently exploratory and relatively weak compared to the rest of the paper. The approach—restricting variants to those near tissue-specific genes—provides limited insight into how well the model captures tissue-specific regulatory effects.

For meaningful progress, the authors could incorporate tissue-specific variant-effect datasets (e.g., MPRA or CRISPR screens) or examine tissue-resolved quantitative trait data. Evaluating how GPN-Star predictions correlate with tissue-specific activity would make this section much more compelling.
Furthermore, all comparisons in this part of the paper are performed against Enformer, which, while relevant, is no longer the strongest available model. Including Borzoi, which has been shown to outperform Enformer on RNA-seq prediction and regulatory modeling, or even AlphaGenome would strengthen the analysis and ensure up-to-date baselines.

4 — Beyond variant-effect prediction

Although the paper positions GPN-Star as a “foundation model for genomics,” the experiments focus almost exclusively on variant-effect prediction. This raises questions about whether the learned representations are general enough to transfer to other tasks, such as functional-element annotation or predicting experimental genomics data (e.g., ATAC-seq or ChIP-seq).

The authors should test GPN-Star on additional downstream tasks or, at least, perform probing analyses showing that its representations capture diverse genomic features. For example, linear-probe classification on promoter vs. enhancer vs. intronic sequences could demonstrate that the model encodes meaningful functional structure.

In the interpretation section, the claim that GPN-Star “learns to recognize a wide range of functional elements in the genome” is supported mainly by a UMAP visualization. This is not sufficient evidence. The authors could follow the approaches used in the Nucleotide Transformer and When Berthology Meets Biology papers, where quantitative evaluations (e.g., clustering metrics or classification accuracy) were used to demonstrate functional organization.

Finally, if the authors intend GPN-Star to be used as a general-purpose foundation model, they should provide fine-tuning results on supervised tasks. The current framing implies broad utility, but the presented evidence remains narrow.

5 — Model scaling and context effects

Supplementary Figures 1 and 18 show that increasing model size or context length leads to only modest gains. This observation deserves discussion in the main text, as it has implications for model design and efficiency.

If larger context windows or bigger models do not substantially improve performance, it suggests that most relevant information is captured by shorter evolutionary blocks, possibly due to the fragmented nature of WGAs. Alternatively, the model may already saturate at this scale, or certain architectural constraints (such as shared attention across species) limit the benefits of scaling. Explicitly discussing these possibilities and relating them to the absence of ablations would help the reader interpret the results.

6 — Practicality and computational considerations

Requiring WGAs or MSAs at inference time poses a serious barrier to adoption. In practice, generating and maintaining

genome alignments across hundreds of species is computationally expensive and technically complex. Users would need access to large alignment datasets and consistent reference versions, which limits usability—especially for new genomes or clinical pipelines.

This issue has also been observed in protein modeling: MSA-Transformer outperformed single-sequence models like ESM, yet most users adopted ESM because it is simpler and faster to use. Similarly, recent versions of AlphaFold have deliberately moved away from MSAs for practicality. A discussion of these precedents and their implications for GPN-Star would be valuable.

An experiment training with MSAs but removing them at inference could be particularly informative. If performance remains strong, this would suggest that alignments help primarily during training (by accelerating learning) rather than being strictly required for prediction. Such a finding would make GPN-Star far more practical.

In addition, comparisons of computational cost should control for model size and version. Stating that GPN-Star is “more efficient” than NT or Evo is misleading if the models differ drastically in objective and parameter count. Providing normalized training times (e.g., hours per billion tokens or FLOPs per variant) would allow a fairer assessment.

Finally, from a usability perspective, the need to choose among three separate models trained at different evolutionary depths (vertebrate, mammal, primate) is impractical. The authors could consider unifying these within a single scalable framework or providing clear guidance on model selection for end users.

Overall assessment

The manuscript presents a strong and technically competent piece of work that meaningfully extends the authors’ previous GPN-MSA framework. The idea of leveraging phylogenetic information for genome modeling is sound and well-motivated, and the reported results are impressive across many benchmarks. However, the advance is primarily incremental, and several aspects—particularly architectural justification, fairness of comparisons, and practical usability—require further clarification before the paper reaches Nature standards.
Improving the clarity of presentation, strengthening the benchmarking fairness, and expanding the scope of evaluation (especially regarding generality and tissue specificity) would substantially enhance the paper’s impact.

Figure presentation. Figures summarizing model comparisons (notably Fig. 2) should be redesigned for consistency and visual polish. Axis labels, fonts, and color schemes currently vary between panels, giving an under-finished impression.

(Remarks on code availability)

Referee #4

(Remarks to the Author)
I co-reviewed this manuscript with one of the reviewers who provided the listed reports.

(Remarks on code availability)

Referee #5

(Remarks to the Author)
In this paper, Ye*, Benagas* et al. propose GPN-Star, a new efficient and powerful algorithm to estimate evolutionary constraint. The method leverages Genomic language models and explicitly accounts for confounders such as mutation rate and repeat elements. They provide 3 estimates across vertebrates, mammals and primates. These scores are evaluated through rigorous analyses using an impressive number of disease datasets, spanning rare or common variants, and coding and non-coding variants. GPN-Star is shown to significantly outperform existing evolutionary constraint scores (i.e., phastcons and phylop) as well as other methods predicting deleterious effects.

I am overall very impressed by the results of GPN-Star, although I do not have the expertise to fully understand the methodology. PhyloP and PhastCons scores have been widely used in genetics but have been developed more than a decade ago (2005 and 2010). I anticipate GPN-Star scores to become the gold standard for constraint scores and to be widely used to prioritize disease variants.
I have nevertheless some concerns on the manuscript.

My main critic is the limited biological and evolutionary insights presented in this paper. The main conclusion outcome from this paper is that the timescale of the constraint score matters when prioritizing variants. However, this observation was already described by the Zoonomia consortium in Sullivan et al. 2023 Science, who shown that primate constraint scores were more informative than mammal constraint scores in heritability analyses of non-coding variants in GWAS, while mammal constraint scores were more informative than primate constraint scores in analyses of coding and/or low-frequency variants.

Clear examples of disease variants and disease genes prioritized by GPN-Star but not by other constraint scores would be a plus.
Similarly, leveraging evolutionary constraint to prioritize genetic variants is not new. I would cite recent works from the Zoonomia consortium and from Illumina (e.g. Kuderna et al. 2024 Nature) in the first paragraph of the Introduction.

The GitHub page associated to GPN-Star (https://github.com/songlab-cal/gpn) is not well documented. In addition, prediction scores are not available yet. For a method paper focusing a generating a new tool and new scores for the community, this lack of usability and accessibility is a weakness.

I have also some technical questions and presentation suggestions:
- What is the recommended GPN-Star to prioritize variants in GWAS? GPN-Star (M) outperforms GPN-Star (P) in GWAS fine-mapped variants, but GPN-Star (P) outperforms GPN-Star (M) in heritability analyses.
- Fig 2, 3 & 6: The authors should consider using 95% confidence intervals instead of standard to better represent the uncertainty in the estimates and to avoid potentially overselling the significance of the observed differences.
- From my personal experience, a large proportion of the genome (at least 1%) saturate at a phastcons score of 1 for mammals and primates. I am thus wondering how the authors selected the top 0.1% of phastcons variants?
- Fig 2G: The authors should clarify how the controls have been selected.
- Fig 2J: Could the authors label V, M and P on the Figure?
- The link section for phastcons (P) is https://cgl.gi.ucsc.edu/data/cactus/zoonomia-2021-track-hub/hg38/phyloPPrimates.bigWig. - is it a typo, or do Zoonomia PhastCons scores have been released in a file called phyloP?

(Remarks on code availability)
The GitHub page associated to GPN-Star (https://github.com/songlab-cal/gpn) is not well documented. In addition, prediction scores are not available yet. For a method paper focusing a generating a new tool and new scores for the community, this lack of usability and accessibility is a weakness.

Version 2:

Reviewer comments:

Referee #1

(Remarks to the Author)
We believe the paper is improved and now provides a more complete picture of the conceptual and applicable advances of the modelling approach. We consider the conceptual and technical contributions presented in this manuscript to be timely, innovative, and important. The broader discovery impact remains to be seen; however, we believe this will be a model broadly embraced by the community, and we therefore endorse the publication of the current version.

(Remarks on code availability)
Codebase appropriately annotated and reusable by the community.

Referee #2

(Remarks to the Author)
I co-reviewed this manuscript with one of the reviewers who provided the listed reports.

(Remarks on code availability)

Referee #3

(Remarks to the Author)
The authors have performed a comprehensive review of the manuscript and addressed most points satisfactorily. The points below require final consideration:

2 — Benchmarking and comparison to existing models

This point was not addressed: "Second, the baseline comparisons are not entirely fair or up to date. GPN-Star leverages WGAs, whereas most competing models (e.g., Nucleotide Transformer, Evo, Enformer) operate on single sequences. This gives GPN-Star access to substantially more information, which should be clearly acknowledged."

4 — Beyond variant-effect prediction

The authors performed classification analysis of different genomic regions with GPN-Star embeddings to quantitatively evaluate the capacity of our model to learn functional elements. However, there is no comparison to other genomics models.

This should be done for completeness and a full picture on the quality of GPN-Star embeddings.

6 — Practicality and computational considerations

The authors mention that in their experiments they observed a drop in model performance if the alignment data is not available during inference. This result should be briefly mentioned in the manuscript as it’s a useful insight for the community.

Other comments

As discussed by different reviewers, it is very important that the authors provide genome-wide predictions for all the models and the precomputed variant scores upon publication to facilitate the use of the model by the community.

(Remarks on code availability)

Referee #4

(Remarks to the Author)
I co-reviewed this manuscript with one of the reviewers who provided the listed reports.

(Remarks on code availability)

Referee #5

(Remarks to the Author)
The authors have addressed most of my previous comments satisfactorily.

My main concern was the limited biological and evolutionary insight. While this aspect remains somewhat limited, I consider it acceptable given the primary contribution of the manuscript, namely the development of improved constraint scores. The addition of three case studies is valuable and convincing, illustrating cases where GPN-Star identifies variants as deleterious while PhyloP and PhastCons assign near-neutral scores.

I have one remaining request. The conceptual distinction between GPN-Star and classical conservation scores (PhyloP/PhastCons) is not explicit, and clarifying this point would substantially improve interpretability.
I suggest including a schematic figure illustrating contrasting scenarios, for example:
(1) bases predicted as constrained by GPN-Star but not by PhyloP/PhastCons
(2) bases predicted as constrained by PhyloP/PhastCons but not by GPN-Star
In case (1), is the signal driven by weak or noisy conservation across species, or by sequence-level constraints (e.g. motifs or context) that are not captured by site-wise conservation?
In case (2), does this reflect mutation-rate effects, alignment or phylogenetic artifacts, or regions that are evolutionarily conserved but not strongly supported by sequence context?
Making these distinctions explicit would strengthen the manuscript and help position GPN-Star relative to established conservation metrics.

(Remarks on code availability)

Version 3:

Reviewer comments:

Referee #3

(Remarks to the Author)
The authors have addressed my previous comments satisfactorily.

(Remarks on code availability)

Referee #4

(Remarks to the Author)
I co-reviewed this manuscript with one of the reviewers who provided the listed reports.

(Remarks on code availability)

Referee #5

(Remarks to the Author)
The authors have perfectly addressed my last requests.

(Remarks on code availability)

Open Access This Peer Review File is licensed under a Creative Commons Attribution 4.0 International License, which permits use, sharing, adaptation, distribution and reproduction in any medium or format, as long as you give appropriate credit to the original author(s) and the source, provide a link to the Creative Commons license, and indicate if changes were made.
In cases where reviewers are anonymous, credit should be given to 'Anonymous Referee' and the source.
The images or other third party material in this Peer Review File are included in the article's Creative Commons license, unless indicated otherwise in a credit line to the material. If material is not included in the article's Creative Commons license and your intended use is not permitted by statutory regulation or exceeds the permitted use, you will need to obtain permission directly from the copyright holder.
To view a copy of this license, visit https://creativecommons.org/licenses/by/4.0/

Point-by-Point Response to Reviews

Manuscript ID: 2025-09-23375
Title: Predicting functional constraints across evolutionary timescales with phylogeny-informed genomic language models

We thank the editor and all the reviewers for their careful reading and comprehensive evaluation of our manuscript. In response to their constructive comments, we have incorporated several new analyses and substantially revised the manuscript. Below is a summary of the major updates:

1. **Expanded and updated benchmarks**
   We have compared with more models in our evaluations. In coding variant effect prediction (Figure 2A-D), we have added ESM-2 (3B and 650M) and ESM-3-open. We moved AlphaMissense and PrimateAI-3D to the main figure where there is no clear data leakage for those models (Figure 2C, D). We added Borzoi to all the heritability enrichment analyses (Figure 3A-C, G, H). We added Enformer to the mouse benchmarks (Supplementary Figure 23).

2. **Systematic ablations studies**
   We performed a set of ablation studies, verified the individual contributions of each phylogenetic module in the model architecture, and demonstrated improved performance over GPN-MSA and MSA Transformer in a direct architectural comparison (Extended Data Figure 1, Supplementary Table 1).

3. **Case studies on disease variants**
   We carefully examined examples in which GPN-Star correctly predicts pathogenic variants while classical conservation scores fail, and reported three concrete case studies that reveal connections between the evolutionary signals learned by the model and functional elements identified by previous experiments (Extended Data Figures 7, 8, 9).

4. **Improved tissue-specific heritability enrichment analysis**
   We refined our approach to constructing tissue-specific GPN-Star annotations by considering regulatory elements. We also added tissue-specific genes and regulatory elements as additional baselines in the analyses (Figure 3G, H, Supplementary Figure 11).

5. **Stratified analyses by functional category**
   We performed stratified analyses of our non-coding variant effect prediction benchmarks (OMIM, HGMD, and GWAS fine-mapped variants) by functional category (Supplementary Figures 3, 4, 5). We further stratified our heritability enrichment analysis

by genomic region and observed a notable improvement in enhancers (Extended Data Figure 9).

6. **Classification of genomic regions with model embeddings**
   To quantitatively assess the model’s ability to distinguish different genomic regions, we performed classification analyses using logistic regression on the model embeddings and observed patterns similar to those in the existing visualization results (Extended Data Figure 6, Supplementary Figure 13).

7. **Clarification of model utilization, limitations, and efficiency arguments**
   We have added new paragraphs on the model's anticipated utility and limitations. We clarified the statements about model efficiency. We have clearly limited the scope of this work to functional constraint prediction of genetic variants. Furthermore, we have committed to publicly releasing precomputed genome-wide model predictions to improve accessibility.

We are grateful for all the valuable feedback, which we think has fundamentally strengthened this manuscript. Please find our detailed point-by-point responses to each specific comment below. Please note that the figure numbers have changed significantly from the previous version. In this file, we always refer to the numbers in the updated version.

Reviewer #1

Summary of the key results

Chengzhong and Benegas et al. describe a novel method, GPN-Star, to model genomes that enables quantifying the likelihood of each nucleotide given a multiple sequence alignment. This builds on the group's previous work on GPN-MSA to build a new genome language model which outperforms all other genome models at a suite of tasks, at a fraction of computational cost. To achieve this, the authors improve GPN-MSA by accounting for the phylogenetic structure of the training data, with an attention module which adaptively weighs each genome. They also account for local mutation rate biases, with a simple average over putatively neutral regions. The resulting model outperforms other genome models at identifying coding and non-coding pathogenic and functional variants, and also shows utility to improve rare variant association testing and identification of the genetics of complex traits. Through explorative analysis, the authors find that model embeddings and co-dependencies qualitatively correlate with annotated genomic regions. Finally, since their model is based on alignments from different species, the authors also benchmark it with available data on non-human model organisms to explore its applicability.

Originality and significance

This work constitutes a great example of the power of biologically-informed model building. The biologically-informed decisions in GPN-Star's architecture, training, and inference schemes drive the improvement in model performance, and constitute a refreshing approach. The biggest advance of this model is on variant effect prediction of non-coding regions, which in itself has the potential for a broad range of applications.

Importantly, the authors put effort into not only publicly sharing their model but also their analyses and predictions, facilitating reproducibility and benchmarking in future studies.

Data & methodology

The benchmarks and methodology are reasonable, and in line with most of the literature. Though see comments below for some clarifications.

Appropriate use of statistics and treatment of uncertainties

The use of statistics and uncertainties is appropriate, though see some questions below.

Conclusions

Overall, this manuscript is an important step forward in the field of genomic modeling. It builds on the group's previous work on GPN-MSA by accounting for the inherent biological nature of the training data, namely its phylogenetic structure and local mutation rate biases. Both

strategies are simple but powerful – the article shows clearly how this model outperforms other genome language models (GLMs) while much more computationally efficient. This work is a great example of how accounting for the biological structure of the data, even with simple approaches, delivers better results than blindly scaling up model size (which so often seems to be the paradigm in parts of ML for biology).

The weakest aspect of this work is that while it demonstrates a new state-of-the-art GLM in various benchmarks, it does not demonstrate its ability, or discuss its limitations, to address biological problems, and it is therefore not totally clear when such a GLM would in effect be useful. In other words, the work would benefit from a deeper exploration of the limitations of GPN-Star, to offer better guidance and interpretation of its outputs in the context of application to biological problems. For example: the biggest advance of this model is on variant effect prediction of non-coding regions. While the current analysis shows an improvement at identifying finemapped GWAS variants, the AUPRCs are still very low, and while there is an enrichment of heritability for constrained non-coding snps (in what is one of the most interesting results of the paper), it fails to demonstrate if and when this would translate into a better understanding of complex traits.

Response: Thank you for acknowledging the significance of our work, and for your comprehensive and thoughtful evaluation. We have carefully incorporated your feedback, adding several new analyses and revising the manuscript accordingly. Specifically, we have performed systematic ablation studies to quantify the contribution of each phylogenetic module, refined our tissue-specific heritability enrichment analysis, and included more comprehensive comparisons in the benchmarks.

We agree that clearly articulating the biological utility and limitations of GPN-Star is important, and we have revised the manuscript substantially in response. We would first like to offer some broader context. GPN-Star is a useful scoring tool, analogous to PhyloP and PhastCons, which have been indispensable to biologists and clinicians for over two decades, not because they directly solve biological problems, but because they provide principled, genome-wide measures of evolutionary constraint that underpin a wide range of downstream analyses. Our goal is to provide a substantially improved version of this type of tool, and we believe the benchmarks demonstrate convincingly that GPN-Star achieves this. We have added a paragraph to the Discussion that clarifies this framing and the anticipated uses of GPN-Star scores.

"We anticipate GPN-Star predictions to be used in ways analogous to classical conservation scores. The model scores carry a specific interpretation: they reflect evolutionary constraint at particular timescales rather than serving as general-purpose variant effect predictions. When interpreting individual variants, we recommend choosing an appropriate timescale based on the patterns described above. For integrating or selecting among models within predictive or discovery workflows, we recommend data-driven approaches that learn appropriate weights and transformations from task-specific data, as we demonstrated with DeepRVAT in our rare variant association testing analysis."

We have also added a paragraph to discuss the limitations of the model:

"Configuring a data pipeline for large alignment datasets can be laborious. GPN-Star requires the availability of the full alignment data at inference time, which can be a practical limitation. To improve accessibility, we are committed to releasing precomputed genome-wide model predictions through major public repositories. Also, the reliance on a fixed alignment format makes the model less suitable for analyzing indels and structural variants. On the other hand, single-sequence gLMs offer greater flexibility and applicability to a broad range of tasks, and can naturally leverage large context and process indels and structural variants. We envision that future work can bridge these paradigms potentially through flexible homology retrieval mechanisms, allowing models to dynamically incorporate evolutionary information without relying on rigid alignment structures."

Regarding the specific concern that low AUPRC on GWAS fine-mapping limits the model's practical utility: we note that this difficulty is not unique to GPN-Star; it reflects a fundamental challenge in the field, as fine-mapped GWAS variants are often only moderately constrained and are difficult to distinguish from background even with the best available tools. Importantly, GPN-Star achieves the highest AUPRC of any method evaluated on this benchmark, and the improvement over the prior state-of-the-art is substantial and consistent across traits.

On the question of whether heritability enrichment translates into a better understanding of complex traits: we believe heritability analysis already includes two concrete examples. First, GPN-Star (P) prioritizes variants with unprecedented heritability enrichment, and the tissue-specific analyses show that brain-constrained variants are most enriched for schizophrenia heritability, blood/immune-constrained variants for lupus, and liver-constrained variants for IGF1; these results align closely with known disease biology and therefore validate that the constraint signal captured by GPN-Star is biologically meaningful. Second, our analysis in Figure 3E relating our model predictions with the polygenicity of traits has revealed important insights about the timescales of evolutionary constraints and the genetic architecture of complex traits. That said, we agree that the true value of our model would be best shown by discovering novel driver genes and causal variants, but such analyses would take significant effort and we think are out of the scope of this study. We are very interested in exploring this in future research. We have modfied relevant paragraph in the Discussion to clearly acknowledge this.

"Our results on pathogenicity prediction and complex trait heritability constitute a systematic evaluation of GPN-Star in the context of current knowledge of human genetic variation. However, the true value of the model depends on its capacity to enable novel biological discovery. We expect GPN-Star to hold great potential in human genetics applications by advancing our understanding of causal variants and human traits. For example, our initial experiments in rare variant association testing showed noticeable improvements, though current analyses were limited to exome data. We anticipate even greater benefits in non-coding regions, where variant prioritization has historically been more difficult. GPN-Star predictions could also enhance other downstream tasks such as functionally informed fine-mapping,

polygenic risk scoring, and phenotype prediction. We are actively exploring these directions in collaboration with domain experts."

Finally, to provide more direct insight into the biology underlying GPN-Star's superior performance, we have added three concrete case studies in the new Extended Data Figures 7, 8, and 9. In each case, GPN-Star uniquely identifies a pathogenic non-coding variant that classical conservation scores miss, and nucleotide dependency analysis reveals the specific functional element — a miRNA binding site, a Kozak sequence, and a promoter initiator element, respectively — that the model has learned to recognize. These examples illustrate both what GPN-Star can do and where it derives its advantage: it leverages sequence context and co-evolutionary signals that site-wise conservation scores cannot capture.

Suggested improvements

• For coherence, and considering that all results shown and described come from calibrated scores, the description of the mutation rate calibration method should come at the beginning of the results section.

Response: Thank you for the suggestion. We have added a description of the mutation rate calibration procedure to the first subsection of the Results section. We retained the correlation analysis with mutation rate and constraint estimates in its original location, given its relevance to evolutionary constraint estimation and to preserve the current figure order.

• Modeling
• The phylogenetic module seems an important piece for achieving high performance, so it would be good to see an ablation study to evaluate the impact on predictive power.

Response: Thank you for raising this important point. We have performed a systematic ablation study of various model components, which have shown that the phylogenetic information is key to improved performance. The results are presented in the new Extended Data Figure 1 and Supplementary Table 1, reproduced below for the ease of reference. The green, red, and purple dots represent ablations of the phylogenetic components to different extents, and we observed that the default GPN-Star architecture consistently outperformed them.

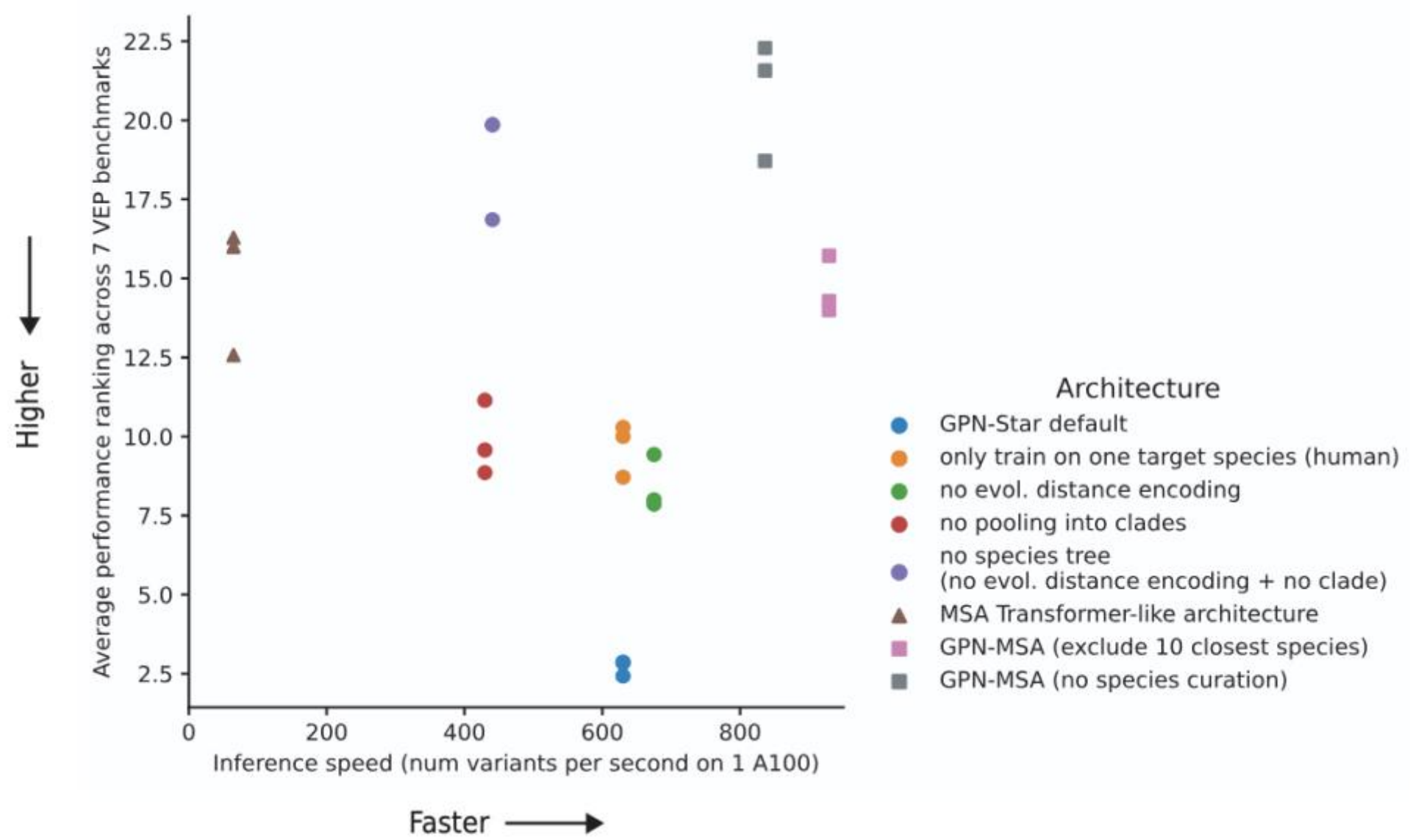

Extended Data Figure 1: Ablation studies. Each set of three dots with the same color represents three random initializations with the same architecture. The average performance ranking is among the models with these 24 architecture-seed combinations. All compared models were trained with the same dataset (multiz100way with training interval curation) and hyperparameters, including model size (25 million), learning rate (1e-4), batch size (512), and number of steps (150k). The MSA Transformer-like architecture neither uses species trees nor distinguishes target and source species. In each encoder block, row-wise self-attention is followed by column-wise self-attention operating on the full alignment.

Supplementary Table 1: Ablation studies. The performance metric is Spearman's ρ for ProteinGym and AUPRC for all the other benchmarks. The numbers outside the parentheses are the mean metrics across 3 random initializations with the same specified architecture, and the numbers inside the parentheses are the max metrics. The best performance in each column is marked in boldface, and the second best is underlined. All compared models were trained with the same dataset (multiz100way with training interval curation) and hyperparameters, including model size (25 million), learning rate (10^{-4}), batch size (512), and number of steps (150k). The MSA Transformer-like architecture neither uses species trees nor distinguishes target and source species. In each encoder block, row-wise self-attention is followed by column-wise self-attention operating on the full alignment.

	ClinVar	COSMIC	ProteinGym	Finemapped coding	OMIM	Finemapped non-coding	HGMD
GPN-Star default	**0.896 (0.897)**	**0.391 (0.395)**	**0.383 (0.384)**	**0.239 (0.241)**	0.713 (0.716)	**0.222 (0.223)**	**0.570** (0.571)
only use one target species (human)	0.883 (0.884)	0.353 (0.354)	0.350 (0.353)	0.233 (0.233)	0.699 (0.703)	0.218 (0.220)	0.561 (0.563)
no evol. distance encoding	0.895 (0.895)	0.377 (0.385)	0.375 (0.375)	0.230 (0.234)	0.699 (0.703)	0.214 (0.216)	0.553 (0.557)
no pooling into clades	0.883 (0.886)	0.333 (0.343)	0.365 (0.367)	0.229 (0.236)	**0.720 (0.725)**	0.209 (0.212)	0.570 (**0.575**)
no species tree (no evol. distances, no clades)	0.880 (0.882)	0.341 (0.353)	0.348 (0.354)	0.222 (0.227)	0.675 (0.681)	0.190 (0.192)	0.530 (0.537)
MSA Transformer-like architecture	0.887 (0.897)	0.351 (0.391)	0.350 (0.357)	0.223 (0.230)	0.696 (0.697)	0.195 (0.199)	0.548 (0.551)
GPN-MSA (exclude 10 closest species)	0.880 (0.881)	0.373 (0.384)	0.345 (0.348)	0.222 (0.226)	0.691 (0.693)	0.211 (0.211)	0.551 (0.553)
GPN-MSA (no species curation)	0.838 (0.844)	0.229 (0.265)	0.271 (0.282)	0.198 (0.203)	0.684 (0.699)	0.200 (0.204)	0.541 (0.553)

• It is striking how much information the model can extract from alignments in such a small window of nucleotides. How reliable are predictions for larger windows for your pretrained models? Can GPN-Star detect long-range interactions compared to other models? You discuss extending context size, but with your models, given the amount of information that the alignment has, can they just work for wider predictions? How bad are they?

Response: As shown in paragraph 5 of the Discussion section and Supplementary Figure 20, increasing context size did not result in improved performance. This is likely because the alignment data are highly fragmented; consequently, using a larger context size introduces increasingly distorted context for non-reference species. In some sense, we are trading long

sequence context for explicit evolutionary context, which, as you pointed out, appears to be vastly beneficial for variant constraint prediction.

Following your suggestion, we further investigated making predictions with longer context lengths up to 1,536 bp (the maximum supported by the positional encoding of our trained models). As shown below, we evaluated the GPN-Star (M) model with varying context sizes on the OMIM and GWAS fine-mapped benchmarks and observed significant performance degradation as context size increased. We therefore do not recommend using the model beyond its trained context size. As noted in the Discussion, extending the context size of alignment-based models remains an important direction for future research.

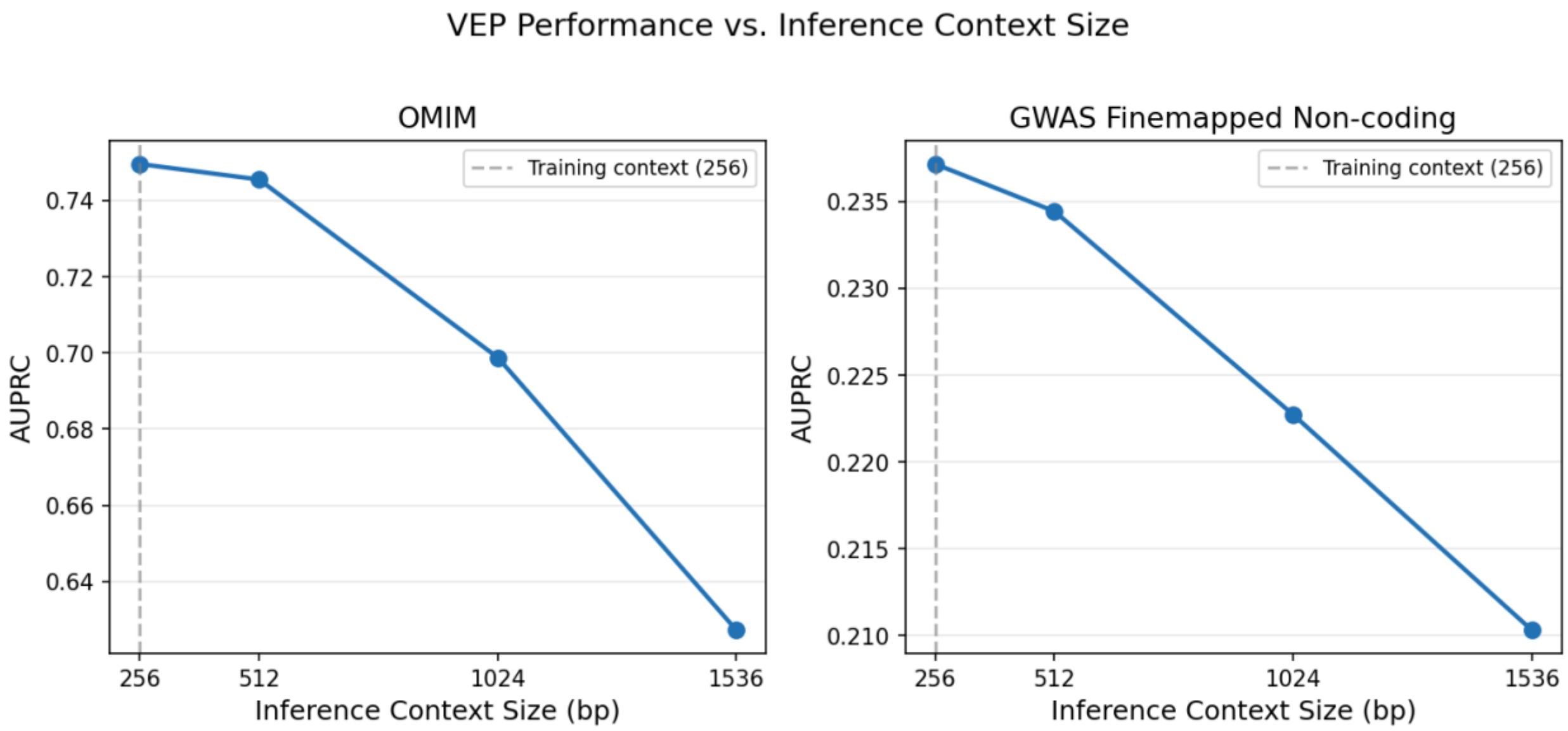

• Latent representations of sequences can be useful to perform linear probing or get a rich summary of a genomic region of interest. Can you assess the utility of GPN-Star's embeddings more quantitatively? For example, the authors could extend their analysis on using embeddings to classify genomic regions, by comparing with embeddings of the same regions produced by other models to quantify their informativeness for different types of genomic annotations. This would highlight the utility of GPN-Star for genome annotation or downstream use for semi-supervised settings. Also, related to the previous one, assess the utility of the model's embeddings for regions longer than the window used for training, e.g., embedding whole genes and predicting their function, essentiality, or other features the authors consider relevant.

Response: Thank you for the suggestion. We have performed a classification analysis of different genomic regions using GPN-Star embeddings to quantitatively evaluate our model's capacity to learn functional elements. In the new Extended Data Figure 6 reproduced below, we show confusion matrices for classification with the 85M GPN-Star (M) model embeddings considering all windows (left) and only conserved windows (right). The classification accuracies of different genomic regions largely agree with the visual patterns observed in the UMAP plots. When considering all genomic windows, some non-conserved windows, particularly among

lncRNA windows, are often indistinguishable from the background. However, when focusing only on conserved windows, the embeddings provide better classification accuracy for each class of genomic region. A moderate fraction of 5’ UTR windows tends to be classified as promoters, consistent with our UMAP observations.

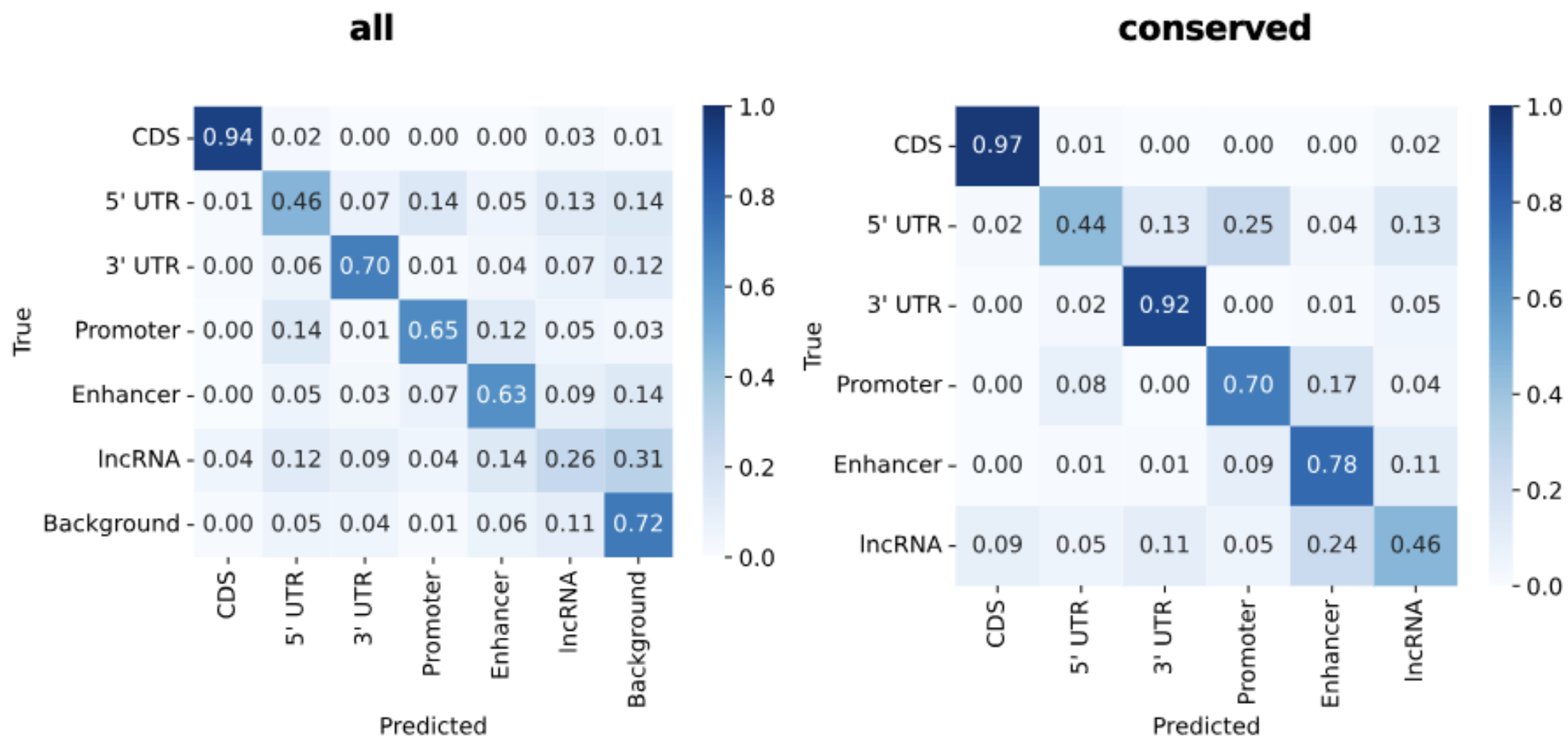

Extended Data Figure 6: Confusion matrices for the classification of window embeddings by logistic regression. The left panel shows results for all genomic windows mixing conserved and non-conserved windows (all). The right panel shows results considering only the conserved windows (conserved).

We show the confusion matrices for all the other models in the new Supplementary Figure 13 and the results follow similar patterns.

We would like to clarify that this study focuses on the functional constraint prediction of genetic variants, which is the primary objective of the model. The visualization of embeddings from different genomic regions is intended as model interpretation rather than a direct application. We have rephrased the corresponding sentence in the Results section to make this point explicit:

“*...we conducted a model interpretation analysis of GPN-Star by visualizing embeddings of genomic windows from gene regions, cCREs, and background regions.*”

We do not expect GPN-Star or current alignment-based gLMs to be the best tool for genome annotation. A clear limitation is the context size. Similarly, embedding sequences the length of entire genes is beyond the capabilities of this model, as indicated in our previous results. We expect the gene function prediction task to be better suited for long-context single-sequence gLMs or protein language models.

• Regarding coding regions, esm1b is treated as SOTA at predicting ClinVar variants, DMS from ProteinGym, etc. This is not a representative picture of the field. In particular, while it is true that PrimateAI and AlphaMissense use population information data and therefore their performance

at predicting ClinVar variants suffers from data leakage, they are unbiased predictors of functional assays (makes no sense to exclude them from the main results plot). Furthermore, esm1b is not SOTA amongst those models which do not use population frequencies.

Response: We have now moved the AlphaMissense and PrimateAI-3D results on ProteinGym and fine-mapped coding variants to the main Figure 2, where there is no apparent data leakage in the datasets. We had originally kept the same set of compared models across all panels to avoid potential confusion. However, we agree that the updated arrangement provides a more representative picture of the field. We have also added the newer ESM-2 (3B and 650M) and ESM-3-open (1.4B) models to the four coding benchmarks. They somewhat outperform ESM-1b on ProteinGym, but underperform on clinical and genetics datasets, consistent with the results reported in the recent Evo 2 paper (Brixi et al., 2026, *Nature*).

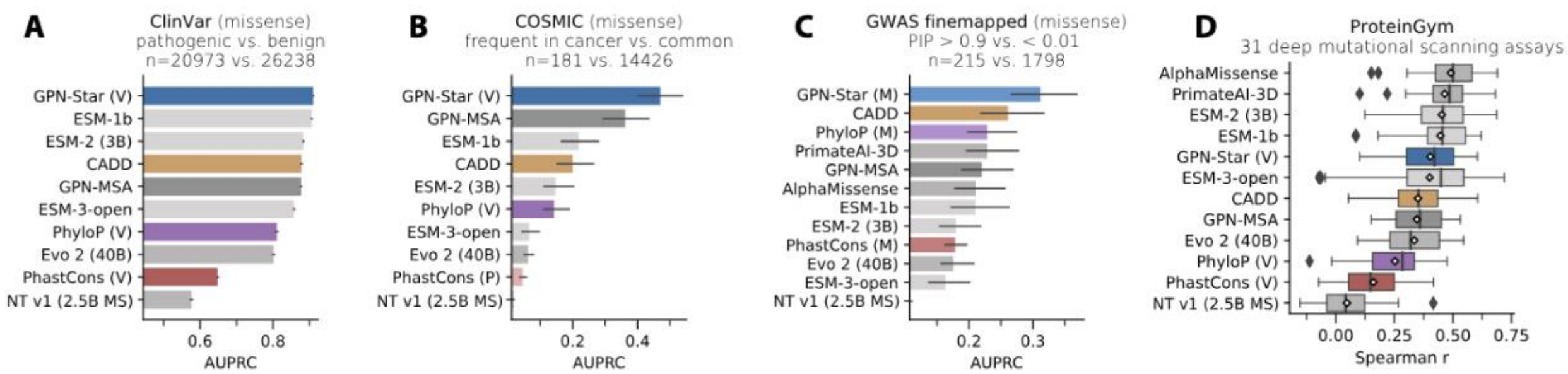

• How are error bars generated in Fig 2D? The variability in results is highly correlated across models (it is more a representation of the specific DMSs, rather than model performance) so if these error bars are generated by randomising assays, they are a bit misleading.

Response: We apologize for the confusion. Unlike the rest of the plots in Figure 2, panel D is a standard boxplot in which each data point represents an assay, and each box spans 31 assays. The displayed intervals are therefore box whiskers rather than error bars. . We have added a clarifying description to the figure caption.

" *Each datapoint represents an assay or protein.*"

• The COSMIC results are very curious. It is a striking result, with the caveat of the extremely unbalanced dataset. The first question is why is this unbalance not reflected in the error bars? (in other words, what is the meaning of these error bars?) And also, why do you think your model is so much better than other protein models? You seem to be implying that it is related to the somatic nature of these variants, but it is hard to see how a GLM would capture better somatic constraints than a PLM for example. Could it be related to the fact that somatic variants are more mildly constrained than those in ClinVar? Do other models suffer from a large number of false positives, or false negatives?

Response: Thank you for your interest in our COSMIC results and for raising the insightful questions. Regarding the error bars, we use stratified bootstrapping throughout the paper to address class imbalance following standard statistical practice (Davison & Hinkley, 1997,

Cambridge University Press). We realized this was not clearly stated in the paper. We have now added clear descriptions in the texts whenever bootstrap is used. For example:

"The performance metric used in (A)-(C) and (E)-(G) is the area under the precision-recall curve (AUPRC). The error bars represent 95% confidence intervals from 1000 class-stratified bootstrap resamples."

Regarding why GPN-Star outperformed pLMs on this task, we propose the following hypothesis: The notion of cancer-causing mutation may only make sense in complex eukaryotic organisms. For single-cell organisms, such mutations are likely not harmful or even beneficial, as they facilitate cell proliferation. Therefore, the pLMs whose training data span a very long evolutionary timescale may learn contradictory selection signals that confuse the model. In contrast, GPN-Star training data are drawn from vertebrate species only, in which the selective pressure is clearly against these mutations. However, this remains a hypothesis. To properly verify it, one could train a pLM with protein data restricted to multicellular species, which is beyond our capability and the scope of this work.

• It would be good to say which kind of (pathogenic) variants are in the OMIM/HGMD non-coding datasets. It seems like two thirds of the OMIM variants are in promoters? Are all other splice disrupting?

Response: We have added fully stratified results on OMIM, HGMD, and the fine-mapped non-coding variant benchmarks by different variant types. The number of variants in each category (with number of positive variants > 10) is available in the new Supplementary Figures 3, 4, 5. You may notice that the number of "promoter variants" in the new stratified results is different from that in Figure 2H. This is because the same variant can belong to different categories. In the previous promoter-specific results, we included all variants where PromoterAI has made predictions on, which are supposedly all variants that can be categorized into the promoter region. In the new stratified results, we used Ensembl VEP with the "--most-severe" flag to assign each variant to the most severe one consequence, therefore some of those promoter variants are assigned to higher impact classes in their hierarchy. We decided to keep the two categorizations, as the two analyses serve different purposes. But as shown, the ranking of models is stable, regardless of the annotation approach.

• Do you understand what is going on with PromoterAI? Why is its performance so much worse than, say, CADD? Isn't this contradicting their main claims?

Response: PromoterAI is a sequence-to-expression model and was primarily evaluated on regulatory genomics tasks in their paper, such as predicting gene expression, protein abundance, and transcription factor binding, whereas our analysis focuses on predicting disease-causing variants and trait-altering variants.

The discrepancy in performance compared to models like CADD is likely driven by the distinct training objectives. CADD is explicitly trained to score variant deleteriousness by contrasting

observed human variation with simulated mutations, making it highly optimized for the pathogenicity benchmarks used in our study. In the one benchmark on disease variants (GEL rare disease) in the PromoterAI paper, they did not compare with any other model, and focused on showing the additional contribution on top of a set of existing variant annotations. Hence, there is no direct conflict between their results and ours. That dataset is not conveniently available for public access, and we are not able to directly evaluate on the same analysis.

• How are functional models AlphaGenome, Borzoi, and Enformer used for predicting pathogenic variants? Does using the effect on transcription or accessibility (where applicable) only as a proxy to compute L2 scores change the result?

Response: For the three models, we first obtain the "L2" score aggregating log fold changes in activity within a single track, as proposed in Borzoi paper (Linder et al., 2025, *Nat Genet*). We then aggregate across all tracks. In the case of Enformer/Borzoi, we use the L2 norm, as proposed in TraitGym (Benegas et al., 2025, *bioRxiv*). In the case of AlphaGenome, we aggregate instead with a max operation, which the authors recommend instead (Avsec et al., 2026, *Nature*). The results indeed change depending on which modalities are included in the aggregation (Benegas et al., 2025, *bioRxiv*). Overall, the idea of obtaining a zero-shot pathogenicity score from a sequence-to-function model is relatively new. We here adopt simple approaches that have been used in the past, making no assumptions over the specific tissue or molecular mechanism.

• How does GPN-Star perform at predicting insertions and deletions?

Response: We would like to clarify that GPN-Star only focuses on predicting the effects of SNVs. Due to the structure of the alignment data, predicting indels is not straightforward, as they create gaps in the alignment. Also, the masked language modeling setup is not ideal for assessing likelihood with variable input length. Overall, we think this task would be better tackled with generative single-sequence gLMs. We have added a sentence in the Discussion section to acknowledge this.

"Also, the reliance on a fixed alignment format makes the model less suitable for analyzing indels and structural variants. On the other hand, single-sequence gLMs offer greater flexibility and applicability to a broad range of tasks, and can naturally leverage large context and process indels and structural variants."

• It is suggested the heritability enrichment might be led by distal enhancer-like signatures (dELS), since these constitute the vast majority of the "prioritised" non-coding variants by GPN-Star -- is this the case? These dELS are only 2.4-fold enriched compared to the bottom unconstrained common variants, could they be driving the heritability result? It would be interesting to follow the origin of this result, to try to gain an intuition about the genetic origin of the traits studied.

Response: Thank you for the insightful comments and the suggestion. To verify this, we have performed S-LDSC analysis with stratified GPN-Star (P) annotations by different functional regions in the genome, and compared with all variants in each category. As shown in the new Extended Data Figure 3, we confirm particular strong heritability enrichment in the dELS variants with top GPN-Star (P) scores, and the improvement over the baseline dELS annotation with all variants is also particularly pronounced among all categories. The improvements by GPN-Star (P) are substantial in most categories, especially in enhancer-flanking regions and ncRNA. The improvement in the splicing category is relatively weaker, which might be due to splicing variants having relatively fixed functional impacts, e.g. splice donor and acceptor variants are almost certainly high-impact, and splice region variants are generally low-impact; thus, further priortization by GPN-Star offers limited additional improvement.

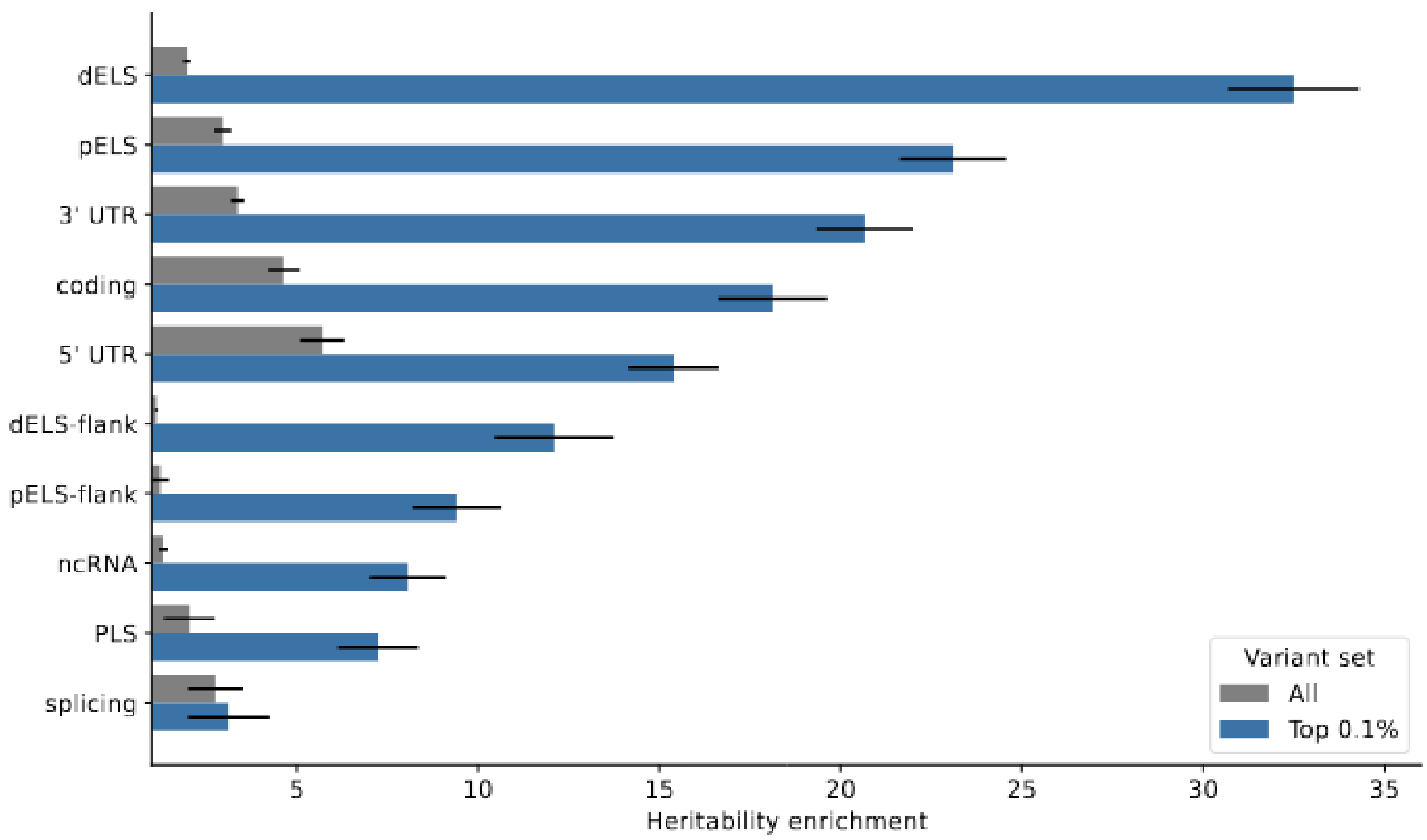

Extended Data Figure 3: Heritability enrichment across 106 GWASs stratified by genomic functional regions. Same as in Figure 3, here we showed the enrichment among the top 0.1% most constrained variants by GPN-Star (P) scores restricted to different functional regions of the genome (blue bars), compared with all variants in each functional regions (gray bars). dELS: distal enhancer-like signatures; pELS: proximal enhancer-like signatures; PLS: promoter-like signatures. These annotations were obtained from ENCODE cCRE v3. The splicing category encompasses all types of splicing variants.

• In the tissue-specific heritability enrichment, you could also consider mutations in regulatory elements that control the expression of tissue-specific genes, as tissue-specific regions in your benchmark.

Response: Thank you for the suggestion. We have made two updates to the tissue-specific heritability enrichment analysis, which are described below. We have also included a figure below to illustrate these results.

1. We refined our approach for creating tissue-specific GPN-Star annotations by incorporating regions spanned by tissue-specific cis-regulatory elements (cCREs) from ENCODE v4 alongside tissue-specifically expressed gene (SEG) regions. This further improved heritability enrichments in GPN-Star annotations across trait groups. In the figure below, the previous approach is denoted as GPN-Star (P)-SEG (cyan), and we added two new approaches denoted as GPN-Star (P)-cCRE (dark green) and GPN-Star (P)-SEG+cCRE (light green). We decided to present the GPN-Star (P)-SEG+cCRE approach in the main figure as it provides the most robust performance and consistently improves over GPN-Star (P)-SEG.

2. We compared our model against three additional baselines: tissue-specific genes with their flanking regions (SEG), tissue-specific cCREs, and SEG combined with cCREs (SEG+cCRE). As shown in the figure below, these baselines exhibit much lower heritability enrichment than the model-based annotations, demonstrating that the model-based constraint signal is key to the performance.

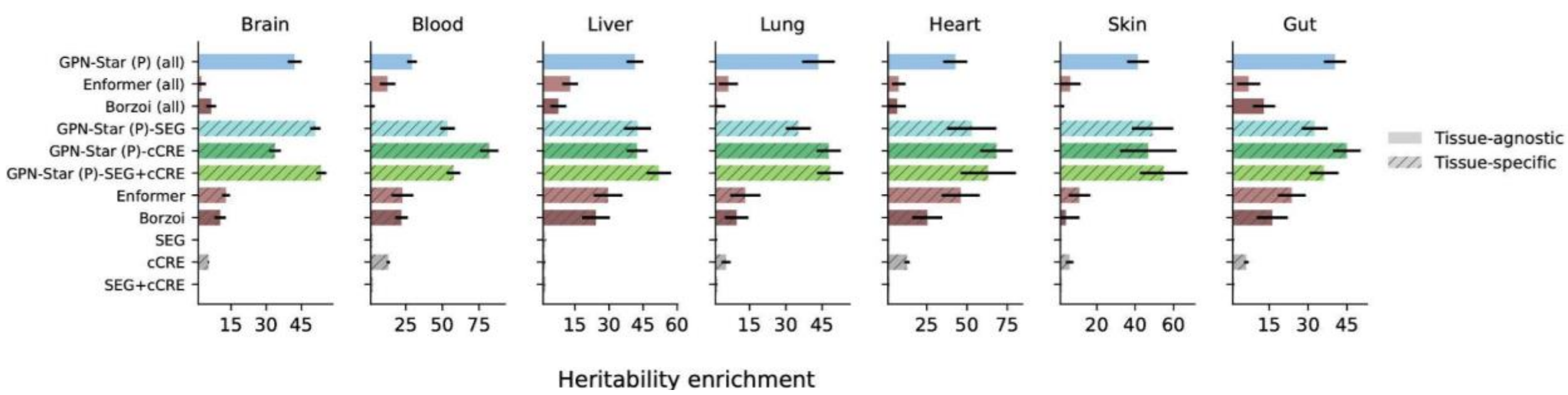

Based on these results, we updated the tissue-specific analysis in Figure 3G and H with the new approach and added the new SEG and cCRE baseline.

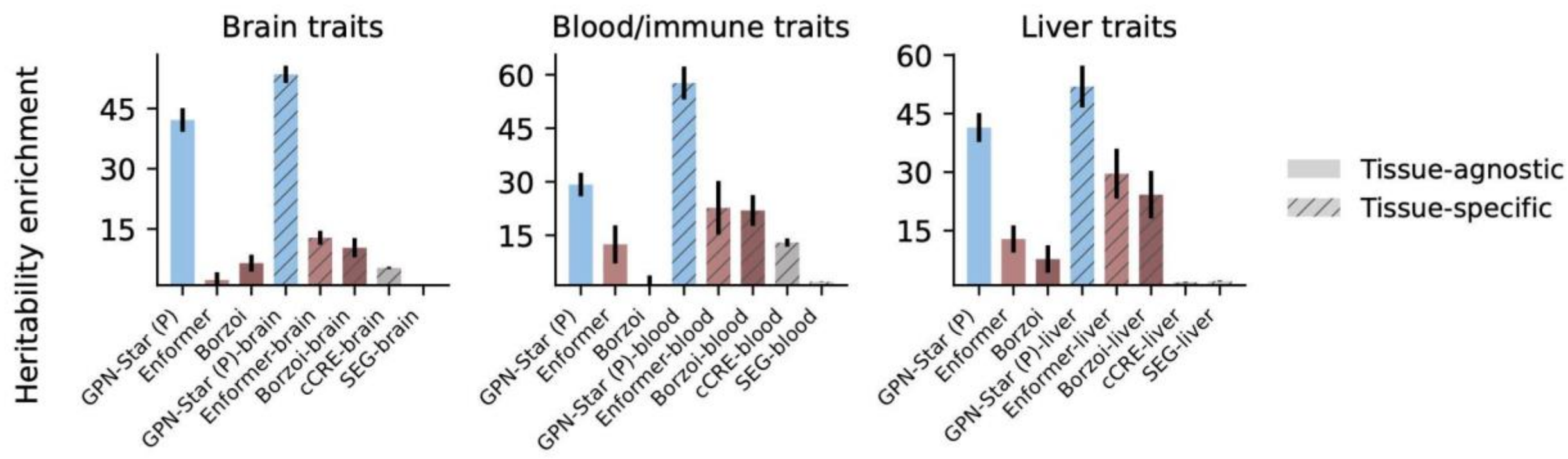

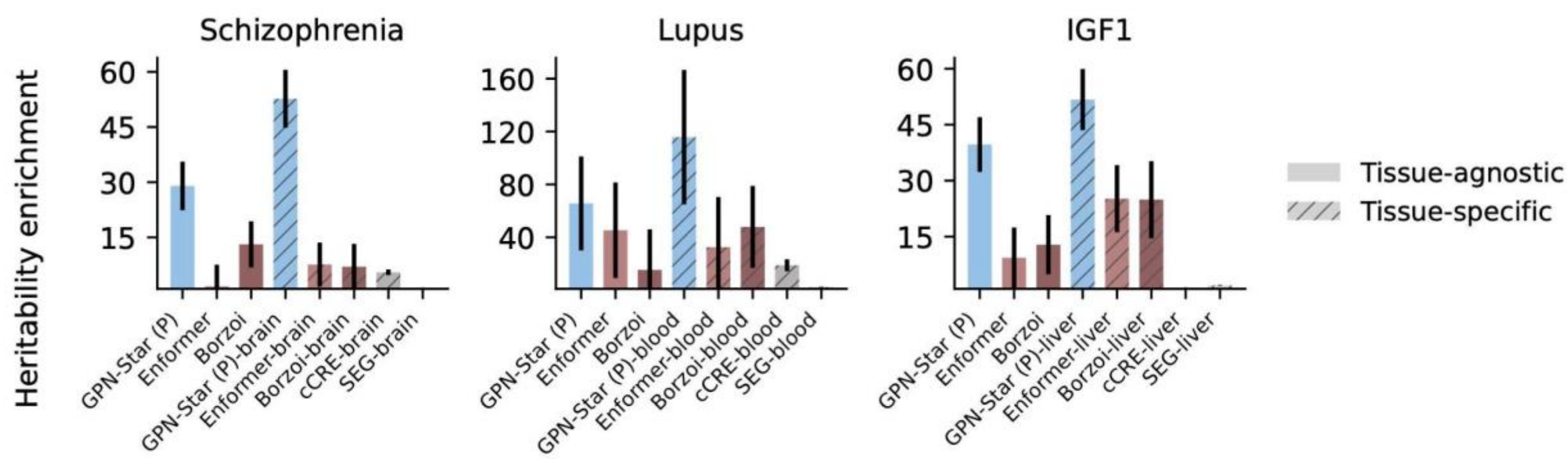

• How do the results of 5B and C (and 6 A) look like as AUPRC or AUC, as in, independently of this threshold of 30 false discoveries?

Response: Thanks for your suggestion to evaluate performance independently of the threshold. However, we think that the global AUROC or AUPRC metrics are not the ideal choice in this specific case. Since a large fraction of the rare variants can still be neutral, a global classification metric effectively penalizes models for failing to distinguish neutral singletons from neutral common variants, which is not desirable. Therefore, we have chosen the enrichment metric to focus on the deleterious tails of the variants.

To address your concern, we performed analyses varying the threshold from 30 to 150 false discoveries. As shown in Supplementary Figure 18, our models' advantage on the odds ratio is robust to different thresholds across the 6 datasets and all variant types. Results on the gnomAD dataset are shown below as a representative example.

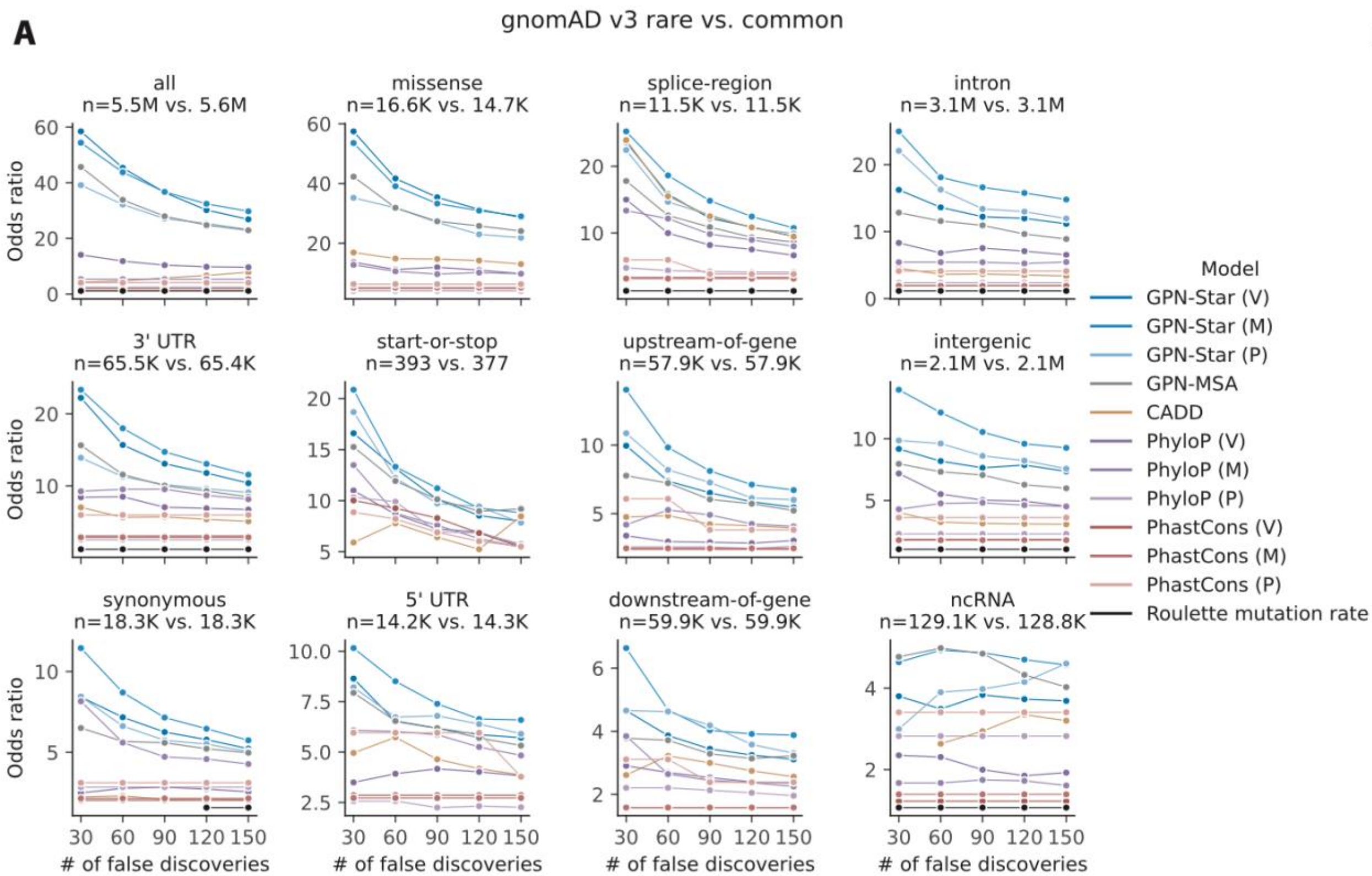

• Benchmarks with other species: Could you include models like AlphaGenome, Borzoi and Enformer with mouse output tracks, following the approach to compare L2 norm of REF and ALT output tracks, similarly as described in TraitGym in the benchmarks for Mus musculus?

Response: We have evaluated the performance of Enformer on non-coding variants in our mouse benchmarks, as shown in the new Supplementary Figure 23. In the rare vs. common variant enrichment analysis, Enformer showed worse performance than GPN-Star, PhyloP, and PhastCons in most cases. Note that we subsampled the variants to reduce computational cost. This agrees with the results we previously reported that Enformer is not effective at predicting evolutionary constraints in the human genome (Benegas et al., 2025, *Nat Biotech*). On the MMrdb benchmark, it also showed poor performance. One possible explanation is that the dataset contains only UTR and splicing variants, which are not the main targets of the model (i.e., promoters and enhancers). Additionally, these are mostly rare Mendelian trait variants, a category where we have observed poor performance in human benchmarks for sequence-to-function models.

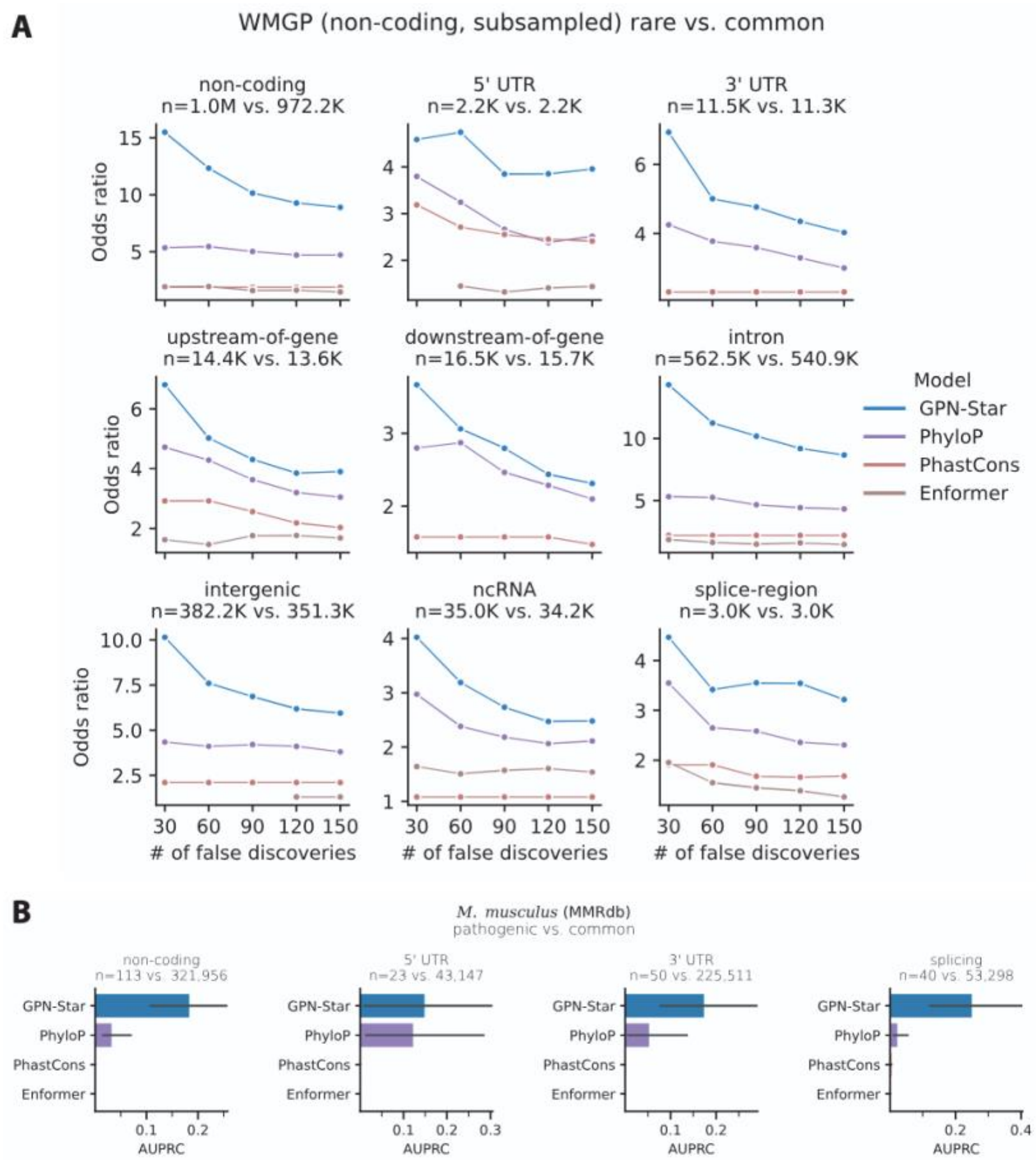

Supplementary Figure 23: Evaluation of Enformer on the non-coding variants in mouse benchmarks. (A) Enrichment of rare vs. common variants in the deleterious tails of model predictions, varying the threshold from 30 to 150 false discoveries. Data points are absent when enrichment is not significant by Fisher's exact test. Only non-coding categories were retained, and the variants are subsampled to 5% to reduce computational burden. (B) AUPRC of classifying non-coding pathogenic variants from MMRdb from WMGP common variants.

Running inference with such models is computationally intensive given their long context sizes and large numbers of output tracks. Running Enformer on these 1.5 million variants took us one week on four A100 GPUs. Borzoi and AlphaGenome are expected to be several times slower than Enformer, and evaluating them at this scale is beyond our current computational capacity.

• Methods

• There are multiple sections where the authors mention their data preprocessing was performed as in a reference, without explaining what was done. The manuscript should be more self-contained. Please include a brief description of the methods followed in this particular project, at least for the reader to have an intuition of what is going on.

Thank you for the suggestion. We have added more self-contained descriptions for the datasets of COSMIC, ProteinGym, OMIM, HGMD, and GWAS fine-mapped variants.

• Genome "repeats" are mentioned across the methods, but never defined. Are they always those from "RepeatMasker" mentioned in "Visualizing Sequence Embeddings" section? Please, clarify.

We have added a sentence where repeats are first mentioned in the Methods section:
"*Repetitive positions were extracted from lowercase letters in the soft-masked Ensembl genome \cite{ensembl}, processed with RepeatMasker \cite{repeatmasker} and Dust \cite{dust}.* "
In the sequence embedding visualization, we directly used the RepeatMasker annotation, as currently described.

• Minor
• "Alignment- and phylogeny-informed genomic language models": please introduce the concept of clade that is used in the model architecture.

Response: We have added a sentence in the corresponding paragraph:

"*To efficiently represent phylogenetic structure in our model, we group closely related source species into clades according to their evolutionary distances and pool their sequences into clade-level representations.*"

• "Relevant evolutionary timescales for different variant effect prediction tasks": If different timescales are useful for different tasks, it would be helpful to explicitly discuss how the user should approach this. Should one make the prediction with all models? What if the predictions differ a lot? How much should we trust each?

Response: Thank you for raising this important practicality issue. For model application, we draw an analogy between GPN-Star predictions and classical conservation scores, such as phyloP and phastCons. GPN-Star scores should not be interpreted as general predictions of variant effects, but rather as measures of functional constraint specific to the evolutionary timescale represented in the alignment data. For instance, a high phyloP score derived from a vertebrate alignment carries the specific interpretation that the variant is highly constrained at the vertebrate evolutionary timescale. We envision GPN-Star scores to be used and interpreted in the same way.

Regarding model selection for applications, we have reported general patterns in the Results and Discussion sections: models trained on longer evolutionary timescales perform better on larger-effect variants, while those on shorter timescales excel with smaller-effect variants. Given the limited evaluation datasets, providing more concrete guidelines than this would be premature. We recommend employing data-driven approaches specific to the downstream task to select or integrate models trained at different evolutionary timescales. For example, future research could develop ensemble deleteriousness predictors, similar to CADD, that integrate GPN-Star with other variant scores. Alternatively, in statistical genetics, methods typically integrate multiple variant annotations within a statistical framework, as demonstrated in our

analysis with DeepRVAT. We have added a paragraph to the Discussion to clarify anticipated model usage:

"*We anticipate GPN-Star predictions to be used in ways analogous to classical conservation scores. The model scores carry a specific interpretation: they reflect evolutionary constraint at particular timescales rather than serving as general-purpose variant effect predictions. When interpreting individual variants, we recommend choosing an appropriate timescale based on the patterns described above. For integrating or selecting among models within predictive or discovery workflows, we recommend data-driven approaches that learn appropriate weights and transformations from task-specific data, as we demonstrated with DeepRVAT in our rare variant association testing analysis.*"

References: appropriate credit to previous work?

Yes, though some SOTA methods are missing from benchmarks.

Clarity and context: lucidity of abstract/summary, appropriateness of abstract, introduction, and conclusions

In general, appropriate. Refer to the "suggested improvements" for specific comments.

Reviewer #3

Summary

In this study, Ye et al. present GPN-Star, a new genomics foundation model that builds upon the authors' previous work on GPN-MSA. The model integrates evolutionary information from whole-genome alignments (WGAs) and species trees through a phylogeny-aware architecture. Trained across vertebrate, mammalian, and primate timescales, GPN-Star is evaluated on a wide range of variant-effect prediction (VEP) tasks and demonstrates state-of-the-art performance in both coding and non-coding regions of the human genome, as well as across several model organisms. The work aims to show that incorporating multispecies evolutionary context leads to more biologically grounded variant predictions.

Conceptually, this direction is valuable and timely—variant interpretation remains a central challenge in human genetics, and using multispecies alignments is biologically intuitive. However, many of the ideas presented here are extensions of prior work, particularly GPN-MSA, and the improvements appear incremental rather than conceptual breakthroughs. While the reported performance gains are impressive, it remains unclear how much of this improvement comes from architectural innovation versus larger model size or dataset differences. Furthermore, practical aspects such as model usability and fair baseline comparisons require more careful analysis. Overall, the paper represents an important but evolutionary (rather than revolutionary) step in alignment-based genomic modeling.

Response: Thank you for finding our study valuable, and for your careful reading and constructive critiques. We have incorporated several new analyses and thoroughly revised the manuscript in response, including systematic ablation studies to quantify the individual contributions of each architectural component, updated benchmarks with the latest competing models, and refined clarity and scope of our core claims. We have also expanded the discussions of practical model usage and have committed to releasing precomputed whole-genome model scores to improve accessibility to the broader scientific community.

We would like to explain why we believe GPN-Star is not an incremental advance. We agree that science progresses through cumulative contributions, and we do not claim GPN-Star is a revolution. However, we believe the specific innovations introduced here represent meaningful conceptual departures from GPN-MSA and from the broader field of genomic language modeling, and that the resulting performance gains, particularly in non-coding variant interpretation, are substantial enough to matter for real applications in human genetics.

Regarding architectural novelty, GPN-Star introduces a phylogeny-informed cross-attention mechanism that explicitly encodes evolutionary distances between species directly into the attention calculation. This is not a straightforward adaptation of existing transformer designs: it requires a new formulation of the attention bias term as a learned function of phylogenetic distance, clade-level pooling of source sequences guided by the species tree, and a shared attention map across target species to maintain tractable memory. Together, these components

allow the model to reason about why a position is conserved, distinguishing deep constraint from shallow, in a way that no prior genomic language model has done. The newly added ablation results (Extended Data Figure 1, Supplementary Table 1) confirm that each component contributes independently to performance, and that the full architecture outperforms GPN-MSA by a substantial margin even when fully controlling for model size, dataset, and training setup.

Our mutation rate calibration procedure is a conceptually distinct contribution that has not previously been applied to genomic language models. Genomic sequences observed in nature reflect both selection and mutation, and failing to disentangle the two conflates constraint with mutability. Our approach, which bins neutral sites by pentanucleotide context and uses them to subtract mutation-rate baselines from model scores, reduces the correlation with Roulette mutation rate estimates to near zero, measurably improving downstream performance across most benchmarks (Extended Data Figure 10). This correction is simple in implementation but important in principle, and we expect it to conceptually advance the field of variant effect prediction with gLMs.

Regarding the source of performance gains, we can answer this directly with our updated set of analyses. Model size contributes modestly: performance gains from 25M to 200M parameters are real but small (Supplementary Figure 20). Dataset differences contribute meaningfully, as we extensively discussed in the paper that the choice of alignment data should be tailored to the task. But we also note that our architectural innovations provide the flexibility to train on the diverse datasets: GPN-MSA required manual exclusion of closely related species and could not accommodate heterogeneous alignment compositions or larger alignment sizes, whereas GPN-Star handles these naturally. The ablation results (Extended Data Figure 1, Supplementary Table 1) show that removing the phylogenetic distance encoding, the clade pooling, or the multi-species training objective each degrades performance, confirming that the architecture itself is also an important contributor to the enhanced performance.

Finally, we think achieving state-of-the-art empirical performance on realistic functional constraint prediction tasks with genomic language modeling is a meaningful advancement on its own. In non-coding variant interpretation, which is the most important frontier, GPN-Star outperforms every competing method across OMIM, HGMD, and GWAS fine-mapping benchmarks, including large sequence-to-function models trained on thoudsands of functional genomics tracks. The heritability enrichment results are particularly striking: GPN-Star (P) achieves enrichments that surpass the previous state-of-the-art (PhastCons Primate) by a factor of roughly two, and the conditional coefficient τ^* demonstrates that this enrichment reflects genuinely new information not captured by any existing baseline annotation. For a community that has relied on PhyloP and PhastCons for two decades, we believe these gains are scientifically significant.

We are, of course, open to the view that different readers will draw different lines between "incremental" and "substantial." What we can say with confidence is that the specific problems GPN-Star solves — how to incorporate phylogenetic structure into a genomic language model, how to train on diverse alignments without manual curation, and how to disentangle functional

constraint from mutability — are problems the field has recognized for years, and that our solutions to them are principled, empirically validated, and immediately useful.

1 — Architecture and model analysis

The new model architecture is central to the manuscript, yet its components are not sufficiently analyzed or justified. The paper would benefit from a systematic exploration of what aspects of the design truly contribute to improved performance.

First, ablation studies are missing. The authors introduce several changes relative to GPN-MSA—such as the use of multiple input species, species-aware attention, and alignment-informed pooling—but do not quantify their individual contributions. Evaluating how each architectural component affects downstream variant-prediction performance would be critical to understanding why GPN-Star works.

Response: Thank you for raising this important point. We have performed a systematic ablation study of various model components, including the multi-species training loss, the species-aware attention, and the clade-pooling. We found that each of these components clearly improves the model performance on variant prediction benchmarks, demonstrating that each component is individually beneficial. Also, GPN-Star clearly outperforms GPN-MSA in direct architectural comparisons. The default GPN-Star architecture achieved the best performance in nearly every benchmark compared with the alternative architectures. The results are presented in the new Extended Data Figure 1 and Supplementary Table 1, reproduced below for the ease of reference.

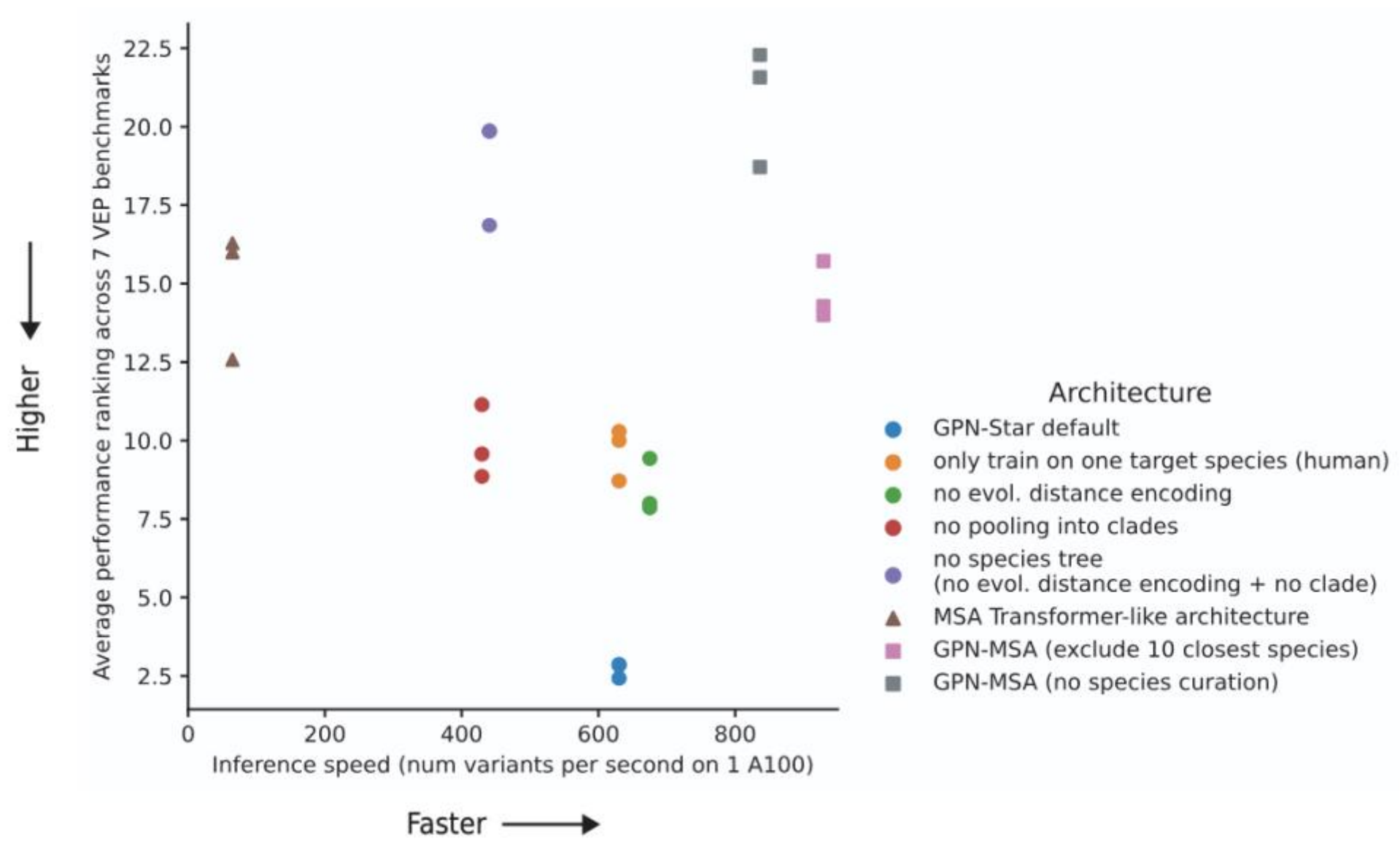

Extended Data Figure 1: Ablation studies. Each set of three dots with the same color represents three random initializations with the same architecture. The average performance ranking is among the models with these 24 architecture-seed combinations. All compared models were trained with the same dataset (multiz100way with training interval curation) and hyperparameters, including model size (25 million), learning rate (1e-4), batch size (512), and number of steps (150k). The MSA Transformer-like architecture neither uses species trees nor distinguishes target and source species. In each encoder block, row-wise self-attention is followed by column-wise self-attention operating on the full alignment.

Supplementary Table 1: Ablation studies. The performance metric is Spearman's ρ for ProteinGym and AUPRC for all the other benchmarks. The numbers outside the parentheses are the mean metrics across 3 random initializations with the same specified architecture, and the numbers inside the parentheses are the max metrics. The best performance in each column is marked in boldface, and the second best is underlined. All compared models were trained with the same dataset (multiz100way with training interval curation) and hyperparameters, including model size (25 million), learning rate (10^{-4}), batch size (512), and number of steps (150k). The MSA Transformer-like architecture neither uses species trees nor distinguishes target and source species. In each encoder block, row-wise self-attention is followed by column-wise self-attention operating on the full alignment.

	ClinVar	COSMIC	ProteinGym	Finemapped coding	OMIM	Finemapped non-coding	HGMD
GPN-Star default	**0.896** (**0.897**)	**0.391** (**0.395**)	**0.383** (**0.384**)	**0.239** (**0.241**)	0.713 (0.716)	**0.222** (**0.223**)	**0.570** (0.571)
only use one target species (human)	0.883 (0.884)	0.353 (0.354)	0.350 (0.353)	0.233 (0.233)	0.699 (0.703)	0.218 (0.220)	0.561 (0.563)
no evol. distance encoding	0.895 (0.895)	0.377 (0.385)	0.375 (0.375)	0.230 (0.234)	0.699 (0.703)	0.214 (0.216)	0.553 (0.557)
no pooling into clades	0.883 (0.886)	0.333 (0.343)	0.365 (0.367)	0.229 (0.236)	**0.720** (**0.725**)	0.209 (0.212)	0.570 (**0.575**)
no species tree (no evol. distances, no clades)	0.880 (0.882)	0.341 (0.353)	0.348 (0.354)	0.222 (0.227)	0.675 (0.681)	0.190 (0.192)	0.530 (0.537)
MSA Transformer-like architecture	0.887 (0.897)	0.351 (0.391)	0.350 (0.357)	0.223 (0.230)	0.696 (0.697)	0.195 (0.199)	0.548 (0.551)
GPN-MSA (exclude 10 closest species)	0.880 (0.881)	0.373 (0.384)	0.345 (0.348)	0.222 (0.226)	0.691 (0.693)	0.211 (0.211)	0.551 (0.553)
GPN-MSA (no species curation)	0.838 (0.844)	0.229 (0.265)	0.271 (0.282)	0.198 (0.203)	0.684 (0.699)	0.200 (0.204)	0.541 (0.553)

Second, the decision to train three separate models (vertebrate, mammal, and primate) is not well justified. It complicates practical use and raises questions about whether a unified approach could capture the same signals. At a minimum, the authors should explain how a user would choose which model to apply to a given problem and whether a single model spanning multiple scales could perform comparably.

Response: Thank you for raising this important practicality issue. For model application, we draw an analogy between GPN-Star predictions and classical conservation scores, such as phyloP and phastCons. GPN-Star scores should not be interpreted as general predictions of variant effects, but rather as measures of functional constraint specific to the evolutionary timescale represented in the alignment data. For instance, a high phyloP score derived from a vertebrate alignment carries the specific interpretation that the variant is highly constrained at

the vertebrate evolutionary timescale. We envision GPN-Star scores to be used and interpreted in the same manner.

Regarding model selection for applications, we have reported general patterns in the Results and Discussion sections: models trained on longer evolutionary timescales perform better on larger-effect-size variants, while those on shorter timescales excel with smaller-effect-size variants. Given the limited evaluation datasets, providing more concrete guidelines than this would be premature. We recommend employing data-driven approaches specific to the downstream task to select or integrate models trained at different evolutionary timescales. For example, future research could develop ensemble deleteriousness predictors, similar to CADD, that integrate GPN-Star with other variant scores. Also, in statistical genetics, methods typically integrate multiple variant annotations within a statistical framework, as demonstrated in our analysis with DeepRVAT. We have added a paragraph to the Discussion to clarify the anticipated model usage:

"*We anticipate GPN-Star predictions to be used in ways analogous to classical conservation scores. The model scores carry a specific interpretation: they reflect evolutionary constraint at particular timescales rather than serving as general-purpose variant effect predictions. When interpreting individual variants, we recommend choosing an appropriate timescale based on the patterns described above. For integrating or selecting among models within predictive or discovery workflows, we recommend data-driven approaches that learn appropriate weights and transformations from task-specific data, as we demonstrated with DeepRVAT in our rare variant association testing analysis.*"

The idea of a unified model spanning all evolutionary timescales is certainly appealing. However, our results suggest that focusing on the appropriate timescale remains key to optimal performance. In some sense, the vertebrate model already encompasses all three timescales, as it includes mammals and primates; yet it does not consistently achieve the best performance across all tasks. A unified model that could automatically determine the optimal timescale from sequence context and other inputs would be highly desirable, but this remains a substantial challenge for future research.

2 — Benchmarking and comparison to existing models

The benchmarking section is extensive, but several issues make interpretation difficult.
First, while the model is tested on standard variant-effect prediction benchmarks (ClinVar, COSMIC, GWAS fine-mapping, etc.), the analysis remains mostly limited to these established datasets. For a paper targeting Nature, it would be valuable to include either additional clinically relevant datasets or finer-grained analyses—for example, distinguishing model behavior across genomic contexts (intronic, exonic, promoter, enhancer, splicing, UTR). This would provide a deeper understanding of what types of variants benefit most from the evolutionary context.

Response: Thank you for this suggestion. We have now included fully stratified results on the disease variant (OMIM, HGMD), and the fine-mapped non-coding variant datasets by different

molecular consequences (Supplementary Figures 3, 4, 5). Due to the small sample size in most of the strata, the results should be interpreted with care. Still, we observed the following pattern:

1. GPN-Star shows robust performance in most cases, topping 14 out of 17 consequence-dataset combinations. In the remaining 3 cases, the performances are also comparable with the best models.
2. The models with the evolutionary context (alignments) showed the largest advantage in disease variants in enhancers. Large gaps were observed between them and the other single-sequence models.
3. Sequence-to-function models perform robustly on promoter variants while less so on UTR variants.
4. Evo 2 performs decently in near-gene regions while poorly in distal enhancers, as we previously reported (Benegas et al, 2025, *bioRxiv*).

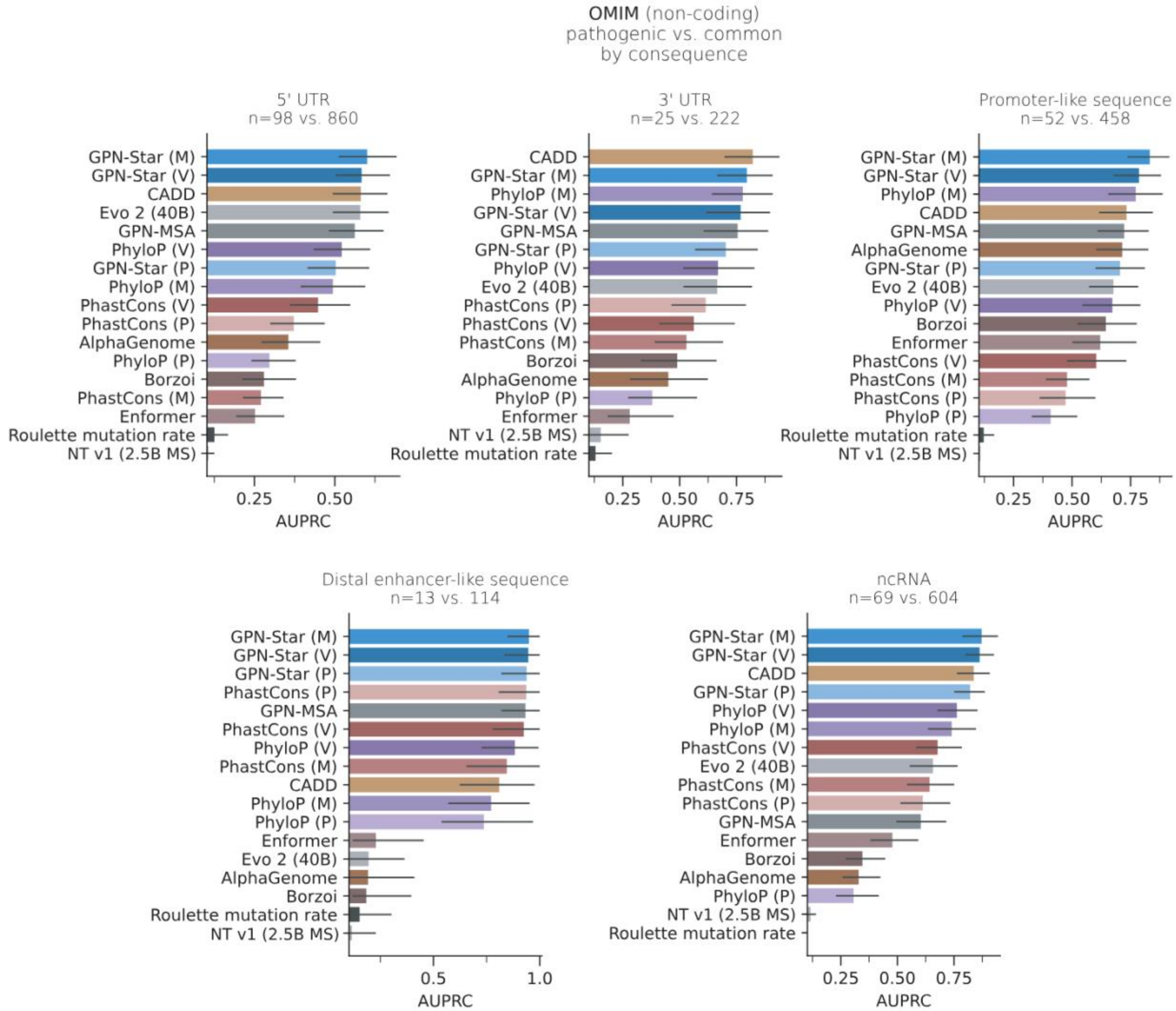

Supplementary Figure 3: Model performances on the OMIM benchmarks stratified by molecular consequences. The datasets and figure setups are the same as Figure 2 otherwise. Only categories with at least 10 positive variants were retained.

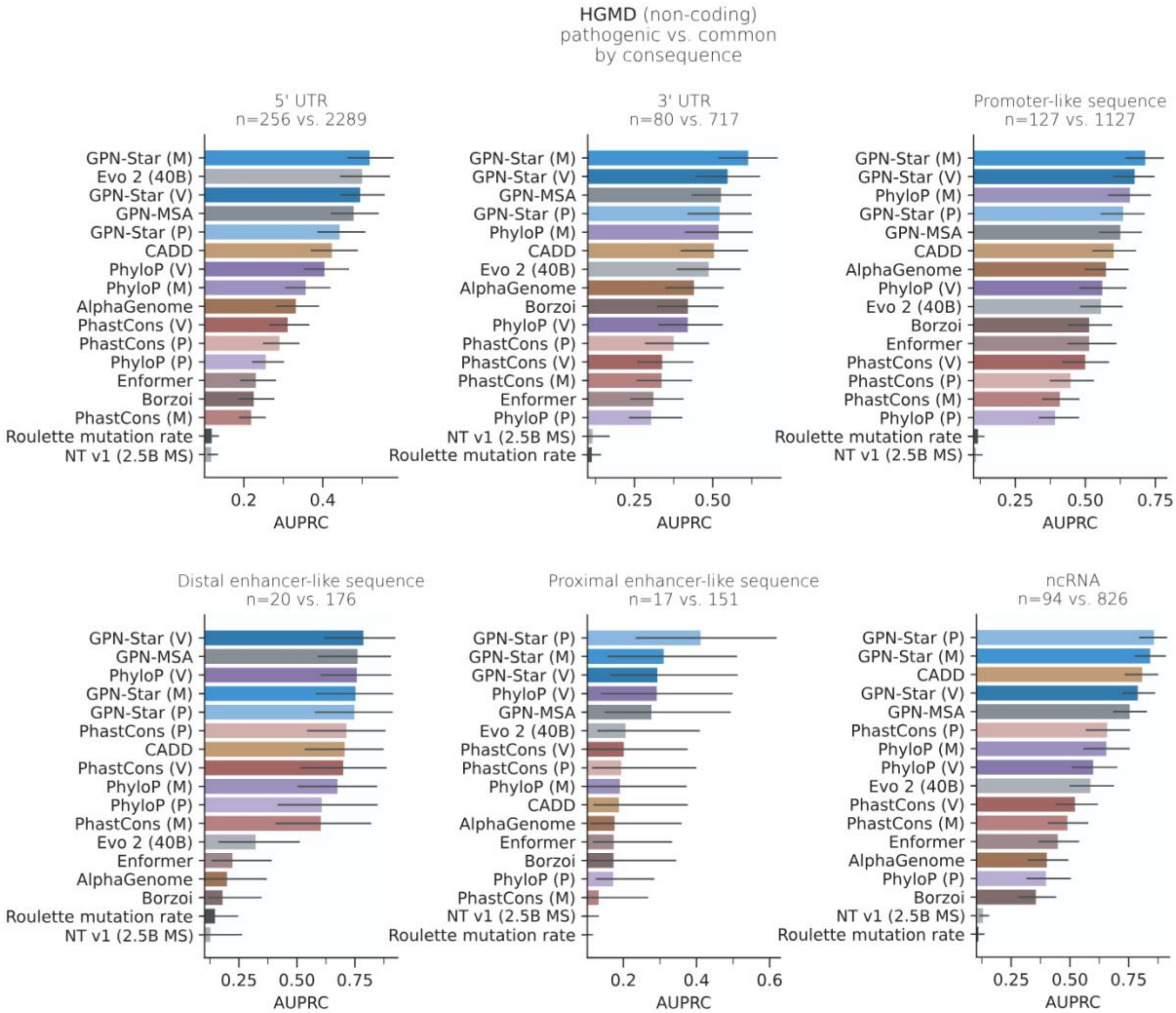

Supplementary Figure 4: Model performances on HGMD benchmark stratified by molecular consequences. The datasets and figure setups are the same as Figure 2 otherwise. Only categories with at least 10 positive variants were retained.

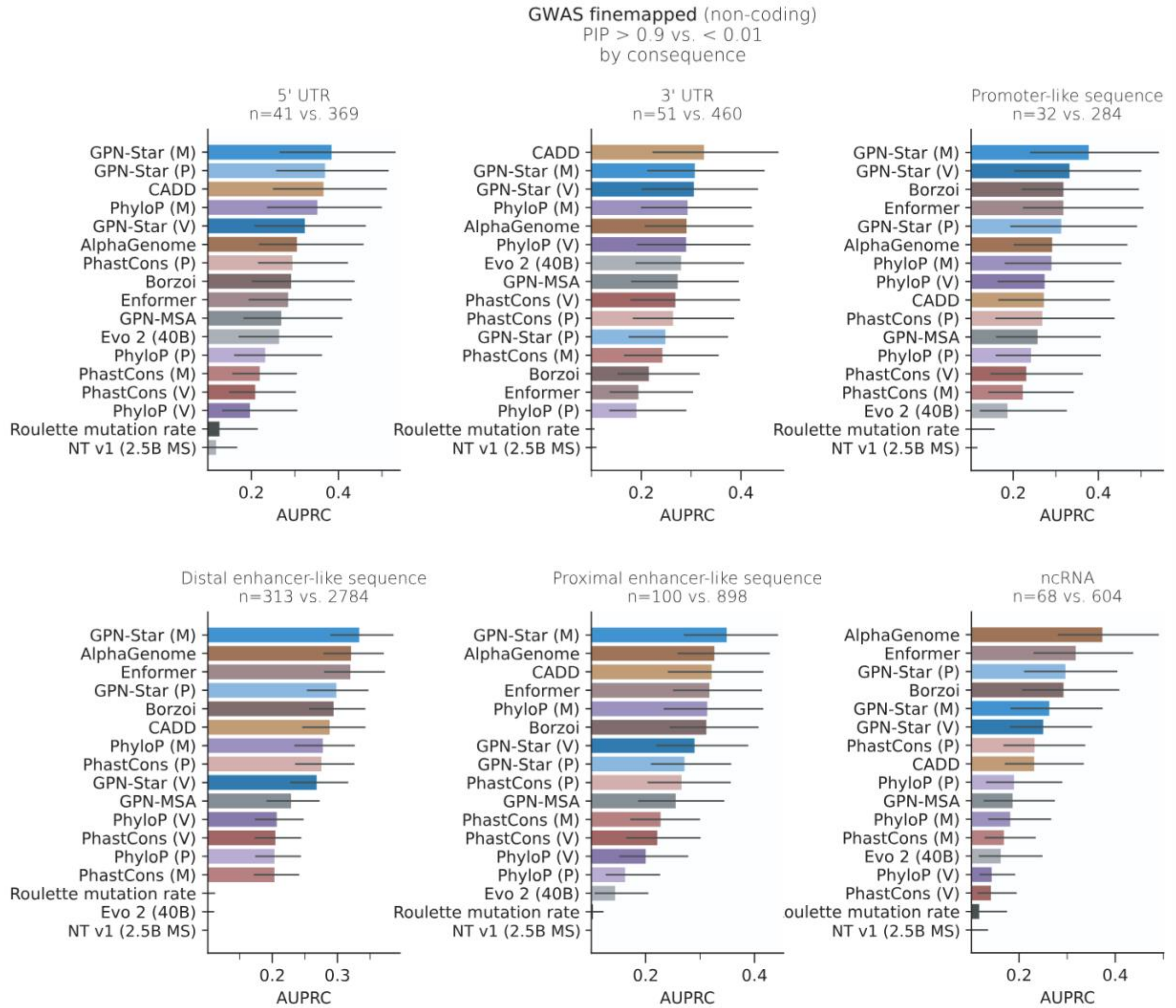

Supplementary Figure 5: Model performances on GWAS fine-mapped non-coding variant benchmark stratified by molecular consequences. The datasets and figure setups are the same as Figure 2 otherwise. Only categories with at least 10 positive variants were retained.

Second, the baseline comparisons are not entirely fair or up to date. GPN-Star leverages WGAs, whereas most competing models (e.g., Nucleotide Transformer, Evo, Enformer) operate on single sequences. This gives GPN-Star access to substantially more information, which should be clearly acknowledged. The comparison to ESM-1b is particularly problematic—this protein model is now several years old, and more recent versions (ESM-2 and ESM-3) have significantly improved performance.The authors should also consider including MSA-Transformer as a protein baseline, since it likewise leverages multiple sequence alignments and would provide a more balanced comparison. Similarly, the authors should specify which Nucleotide Transformer model (v1 or v2) and size were used, as results vary widely across versions and scales.

Response: Thank you for the suggestion. We have added ESM-2 (both 3B and 650M versions, showing the larger model in the main Figure 2) and ESM-3-open (1.4B) to all our coding variant benchmarks. They somewhat outperform ESM-1b on ProteinGym, but not on the other clinical and genetic datasets, such as ClinVar. Similar results were also reported in the Evo 2 paper (Brixi et al., 2026, *Nature*). MSA Transformer has never been applied to clinical variant prediction, and doing so would require substantial additional effort. For example, one would need to explore curating alignment datasets of appropriate depth and composition to achieve good performance on these variants. We consider such work beyond the scope of this study.

Additionally, to reflect a more representative picture of the field, we decided to move the AlphaMissense and PrimateAI-3D results to the main Figure 2 for the two benchmarks with no apparent data leakage by allele frequency (i.e., ProteinGym and fine-mapped coding variants).

Finally, we clarify that we used the largest Nucleotide Transformer model (v1 2.5B multispecies) in the comparison. We have now included all model versions and sizes in the labels of the figures to make it more transparent.

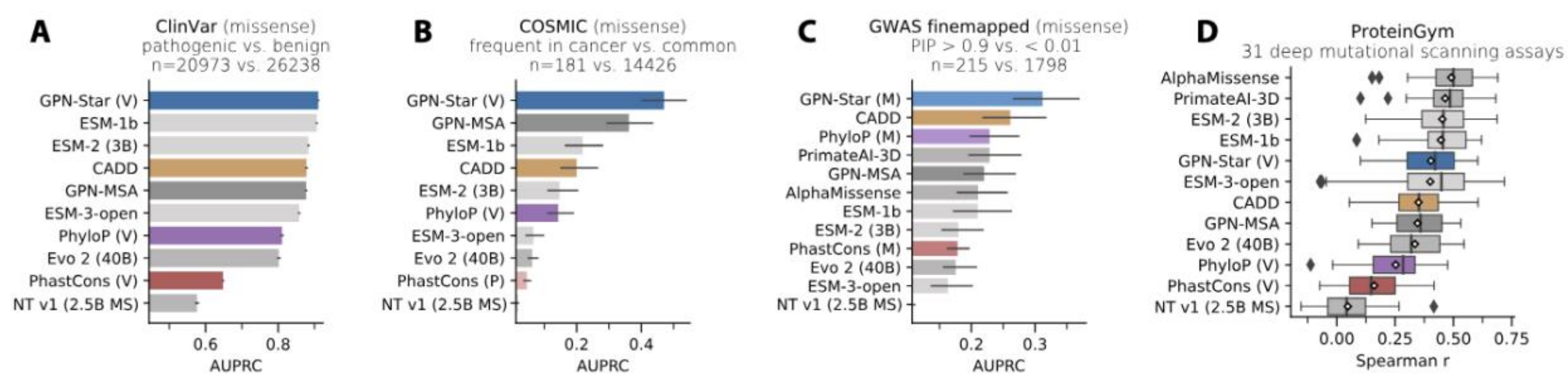

The reported compute comparisons also need clarification. The paper contrasts training budgets between models of very different sizes and goals—Evo (designed primarily for generation), NT (for fine-tuning), and GPN-Star (for VEP). The claim that GPN-Star is more efficient should therefore be treated cautiously unless parameter counts and training data are normalized. Overall, the benchmarking is impressive in scope, but the comparisons must be fair, transparent, and clearly documented.

Response: We apologize for the imprecise wording. We fully acknowledge that single-sequence gLMs like Evo 2 and NT target different primary goals, such as sequence generation, and that zero-shot variant effect prediction is only one component of their broad capabilities.

We have revised the manuscript to clarify that our comparison focuses strictly on the specific task of functional constraint prediction. In this context, we also define "efficiency" at the system level, encompassing both the data format and the model architecture. Because GPN-Star explicitly leverages the evolutionary information present in alignment data, it can achieve state-of-the-art zero-shot variant effect prediction performance with smaller model sizes and training budgets. On the other hand, single-sequence models must implicitly learn these constraints from only sequence context with more parameters, in exchange for flexibility and broader applicability.

We have updated the text noting that these systems are intrinsically different and that the efficiency gains are due to this fundamental difference in data modality rather than a direct architectural superiority.

"*Existing single-sequence gLMs such as Nucleotide Transformer (trained on 128 A100 GPUs for a month) (dallatorre2024nucleotide) and Evo-2 (trained on over 2,000 H200 GPUs for several months) (brixi2025genome) require significantly larger model sizes and computational resources to implicitly learn evolutionary constraints from only sequence context, in exchange for flexibility and broader applicability. For the specific task of constraint prediction, GPN-Star achieves superior performance with a substantially smaller resource footprint by leveraging the explicit evolutionary context provided by alignment data.*"

3 — Tissue specificity

The tissue-specific analyses are currently exploratory and relatively weak compared to the rest of the paper. The approach—restricting variants to those near tissue-specific genes—provides limited insight into how well the model captures tissue-specific regulatory effects.

For meaningful progress, the authors could incorporate tissue-specific variant-effect datasets (e.g., MPRA or CRISPR screens) or examine tissue-resolved quantitative trait data. Evaluating how GPN-Star predictions correlate with tissue-specific activity would make this section much more compelling.

Response: We would like to clarify that we do not claim GPN-Star learns tissue-specific variant effects. Since our model is trained solely on DNA sequences, it is intrinsically tissue-agnostic. The tissue-specific analyses represent an effort to improve annotation by combining GPN-Star predictions with functional genomics data, where the tissue-specific signal comes from the latter. Therefore, direct correlation of GPN-Star predictions with tissue-specific assays is not our primary evaluation target. We have added a sentence in the corresponding subsection in the Results to clarify this point

"*On the other hand, GPN-Star trained solely on DNA sequences is intrinsically tissue-agnostic.*"

Still, inspired by your comment that our previous approach doesn't consider tissue-specific regulatory effects, we refined our approach for creating tissue-specific GPN-Star annotations by incorporating regions spanned by tissue-specific cis-regulatory elements (cCREs) from ENCODE v4 alongside tissue-specifically expressed gene (SEG) regions. This further improved heritability enrichments in GPN-Star annotations across trait groups. In the figure below, the previous approach is denoted as GPN-Star (P)-SEG (cyan), and we added two new approaches denoted as GPN-Star (P)-cCRE (dark green) and GPN-Star (P)-SEG+cCRE (light green). We decided to present the SEG+cCRE approach in the main figure as it provides the most robust performance and consistently improves over GPN-Star (P)-SEG.

Additionally, we have compared our model against three new baselines as suggested by another reviewer: tissue-specific genes with their flanking regions (SEG), tissue-specific cCREs, and SEG combined with cCREs (SEG+cCRE). As shown in the figure below, these baselines exhibit much lower heritability enrichment than the model-based annotations, demonstrating that the model-based constraint signal is key to the performance.

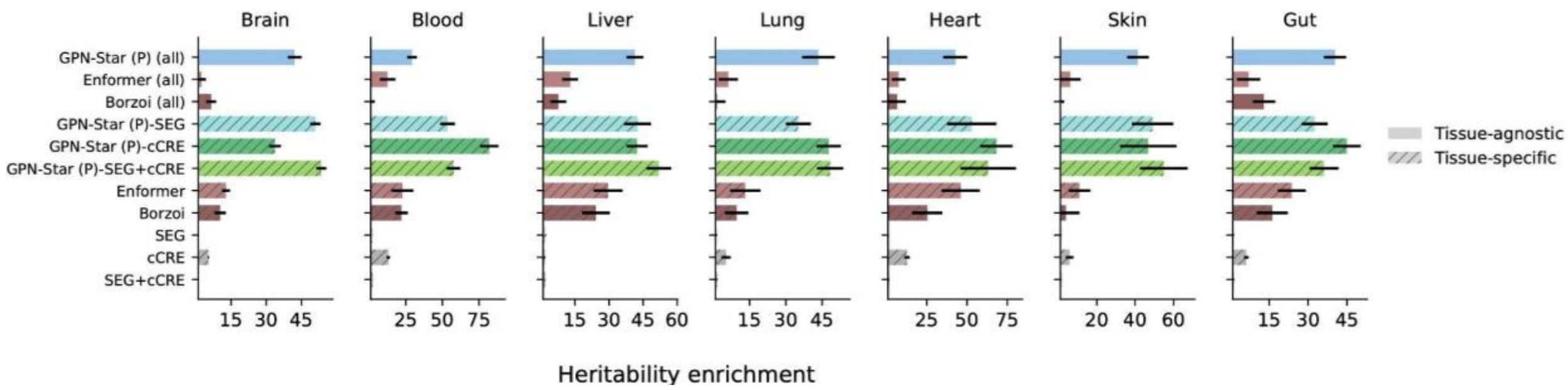

Based on these results, we updated the tissue-specific analysis in Figure 3G and H with the new approach and added the new SEG and cCRE baseline.

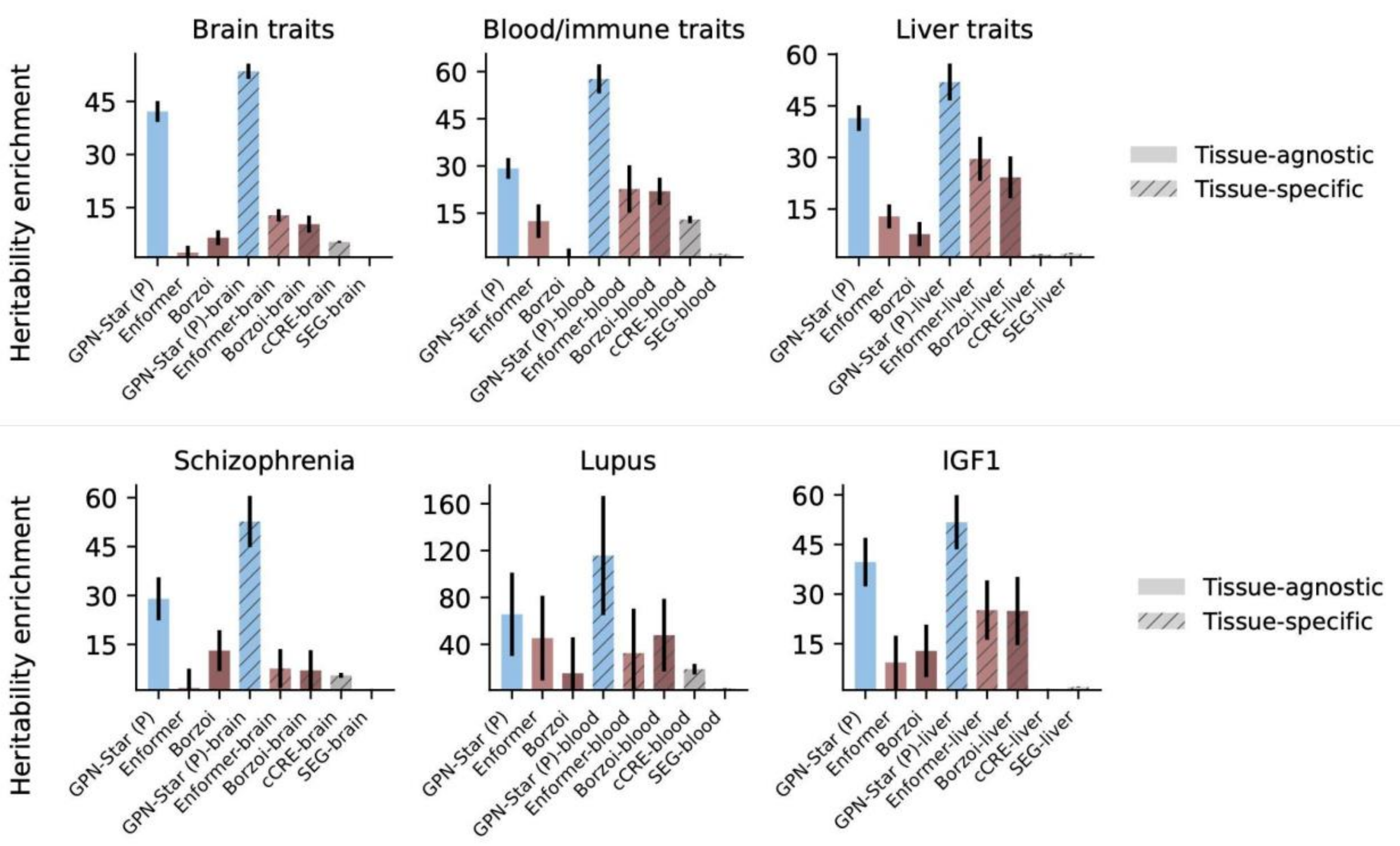

Furthermore, all comparisons in this part of the paper are performed against Enformer, which, while relevant, is no longer the strongest available model. Including Borzoi, which has been shown to outperform Enformer on RNA-seq prediction and regulatory modeling, or even AlphaGenome would strengthen the analysis and ensure up-to-date baselines.

Response: We have added Borzoi to all the S-LDSC analyses alongside Enformer (Figure 3). Overall, Borzoi showed slightly better performance than Enformer. AlphaGenome recently released its model weights, but generating the predictions for the ~10 million variants required in this analysis for such models is currently beyond our computational capacity. Enformer predictions were computed and provided by the cV2F team (Fabiha et al., 2024, *bioRxiv*), while

Borzoi predictions for these variants were recently released by the authors alongside the Sniff preprint (Srivastava et al., 2025, *bioRxiv*), making it possible to compare with these two models.

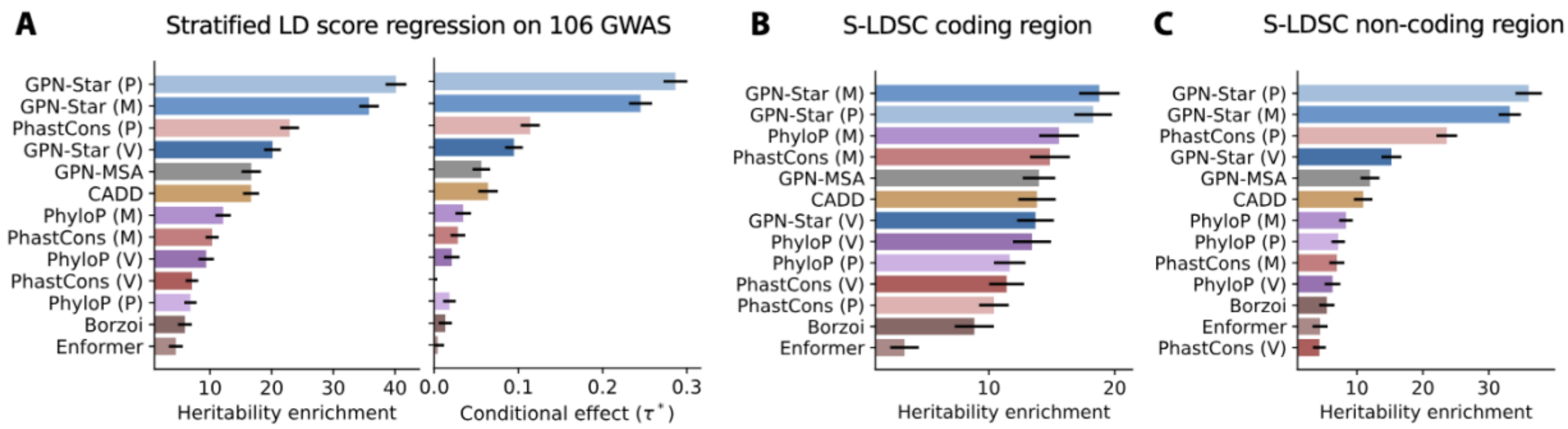

4 — Beyond variant-effect prediction

Although the paper positions GPN-Star as a “foundation model for genomics,” the experiments focus almost exclusively on variant-effect prediction. This raises questions about whether the learned representations are general enough to transfer to other tasks, such as functional-element annotation or predicting experimental genomics data (e.g., ATAC-seq or ChIP-seq).

The authors should test GPN-Star on additional downstream tasks or, at least, perform probing analyses showing that its representations capture diverse genomic features. For example, linear-probe classification on promoter vs. enhancer vs. intronic sequences could demonstrate that the model encodes meaningful functional structure.

In the interpretation section, the claim that GPN-Star “learns to recognize a wide range of functional elements in the genome” is supported mainly by a UMAP visualization. This is not sufficient evidence. The authors could follow the approaches used in the Nucleotide Transformer and When Berthology Meets Biology papers, where quantitative evaluations (e.g., clustering metrics or classification accuracy) were used to demonstrate functional organization.

Finally, if the authors intend GPN-Star to be used as a general-purpose foundation model, they should provide fine-tuning results on supervised tasks. The current framing implies broad utility, but the presented evidence remains narrow.

Response: Thank you for the suggestion. We have performed a classification analysis of different genomic regions with GPN-Star embeddings to quantitatively evaluate the capacity of our model to learn functional elements. In the new Extended Data Figure 6, we show confusion matrices for classification with the 85M GPN-Star (M) model embeddings considering all windows (left) and only conserved windows (right). The classification accuracies of different genomic regions largely agree with the visual patterns observed in the UMAP plots. When considering all genomic windows, some non-conserved windows, particularly among lncRNA windows, are often indistinguishable from the background. However, when focusing only on conserved windows, the embeddings provide better classification accuracy for each class of

genomic region. A moderate fraction of 5' UTR windows tends to be classified as promoters, consistent with our UMAP observations.

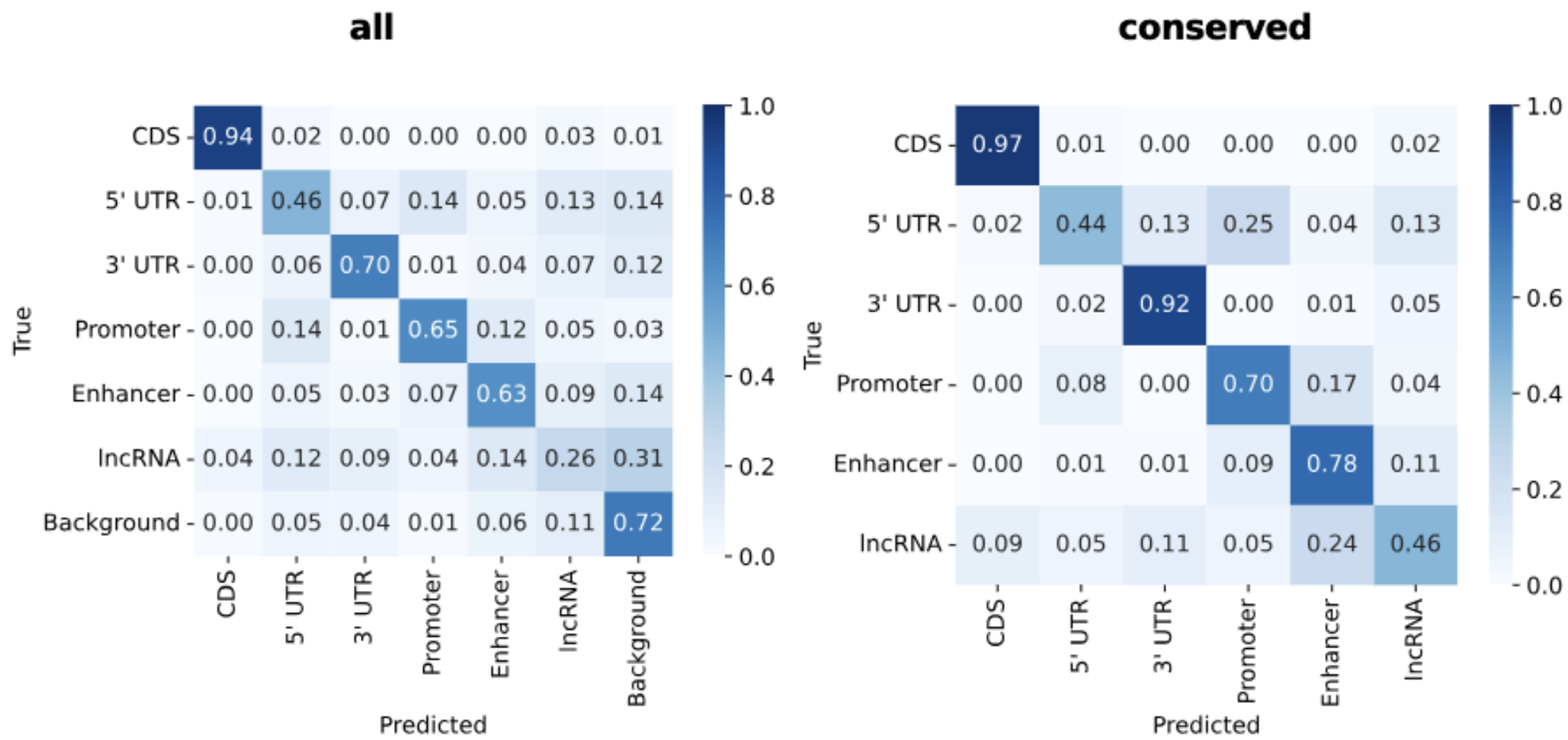

Extended Data Figure 6: Confusion matrices for the classification of window embeddings by logistic regression. The left panel shows results for all genomic windows mixing conserved and non-conserved windows (all). The right panel shows results considering only the conserved windows (conserved).

We show the confusion matrices for all the other models in the new Supplementary Figure 13 and the results follow similar patterns.

We would like to clarify that this study focuses on functional constraint prediction of genetic variants, and we have been careful not to overclaim GPN-Star as a "foundational model" that excels across all genomic tasks. The visualization of embeddings from different genomic regions is intended as model interpretation rather than as a demonstration of broad applicability. We have rephrased the corresponding sentence in the Results to make this point explict.

"...we conducted a model interpretation analysis of GPN-Star by visualizing embeddings of genomic windows from gene regions, cCREs, and background regions."

In our manuscript, we only mentioned GPN-Star's potential for transfer learning tasks in the Discussion as a direction for future work, which we think still requires substantial research to obtain convincing evidence. To clearly limit the scope of this work, we have deleted this part from the Discussion.

5 — Model scaling and context effects

Supplementary Figures 1 and 18 show that increasing model size or context length leads to only modest gains. This observation deserves discussion in the main text, as it has implications for model design and efficiency.

If larger context windows or bigger models do not substantially improve performance, it suggests that most relevant information is captured by shorter evolutionary blocks, possibly due to the fragmented nature of WGAs. Alternatively, the model may already saturate at this scale, or certain architectural constraints (such as shared attention across species) limit the benefits of scaling. Explicitly discussing these possibilities and relating them to the absence of ablations would help the reader interpret the results.

Response: Our scaling experiments suggest that the model is already near saturation at the current the model scale and context size. We offer two possible explanations in the Discussion section: first, alignment-based models typically require far fewer parameters to achieve strong performance, as illustrated by AlphaFold2 being substantially smaller than ESMFold, likely because explicit homology information simplifies the learning task; second, as you noted, the highly fragmented nature of alignment data restricts effective use of larger contexts. Still, it is an interesting hypothesis that architectural innovation could potentially unlock further scaling capacity. We have added a sentence in the Discussion section to acknowledge this:

"*Exploring how to leverage larger context sizes with WGA data is a promising direction for future research. Further advancement in the design of model architecture or the alignment data structure is likely necessary.*"

6 — Practicality and computational considerations

Requiring WGAs or MSAs at inference time poses a serious barrier to adoption. In practice, generating and maintaining genome alignments across hundreds of species is computationally expensive and technically complex. Users would need access to large alignment datasets and consistent reference versions, which limits usability—especially for new genomes or clinical pipelines.

This issue has also been observed in protein modeling: MSA-Transformer outperformed single-sequence models like ESM, yet most users adopted ESM because it is simpler and faster to use. Similarly, recent versions of AlphaFold have deliberately moved away from MSAs for practicality. A discussion of these precedents and their implications for GPN-Star would be valuable.

Response: We agree with the reviewer that handling alignment data can be cumbersome for users. Below, we clarify our rationale for this design choice and outline the measures we have taken to alleviate potential usability issues:

1. Our primary objective is to achieve the best possible performance in functional constraint prediction. Alignment data provides explicit evolutionary information that is crucial for state-of-the-art performance. This parallels the field of protein structure prediction, where explicit homology signals (via MSAs or retrieval) remain key components of recent leading models such as AlphaFold 2/3, Boltz, Dayhoff, Protriever, and Profluent-E1. While AlphaFold 3 indeed intends to reduce reliance on MSAs, the fact that the MSA module is not entirely removed underscores that evolutionary context is still essential for

maximal accuracy, a level of performance that single-sequence models like ESMFold have yet to match.
2. To eliminate the need for users to handle alignment datasets or configure inference pipelines, we are actively working on generating genome-wide predictions for all the models and will release the precomputed scores upon publication.
3. We have established collaborations with the UCSC Genome Browser and IGVF to host our model predictions on their public repositories. We are also committed to updating these scores as new alignment data and assemblies become available.
4. Finally, we fully acknowledge the promise of single-sequence gLMs. We selected an alignment-based approach to prioritize variant effect prediction accuracy. We have added a discussion to provide an outlook of the future synthesis of the two approaches.

"*On the other hand, single-sequence gLMs offer greater flexibility and applicability to a broad range of tasks, and can naturally leverage large context and process indels and structural variants. We envision that future work can bridge these paradigms potentially through flexible homology retrieval mechanisms, allowing models to dynamically incorporate evolutionary information without relying on rigid alignment structures.*"

An experiment training with MSAs but removing them at inference could be particularly informative. If performance remains strong, this would suggest that alignments help primarily during training (by accelerating learning) rather than being strictly required for prediction. Such a finding would make GPN-Star far more practical.

Response: We have done this experiment previously, and unfortunately the model performance drops significantly if the alignment data is not available during inference. We believe that the availability of multi-species information during inference is key to the good performance of GPN-Star. On a similar note, in the ESMFold paper, the authors performed an ablation on AlphaFold2 by removing the MSA during inference, which also resulted in a much worse performance (Lin et al., 2023, *Science*).

In addition, comparisons of computational cost should control for model size and version. Stating that GPN-Star is "more efficient" than NT or Evo is misleading if the models differ drastically in objective and parameter count. Providing normalized training times (e.g., hours per billion tokens or FLOPs per variant) would allow a fairer assessment.

Response: Please refer to our response to the last comment in bullet point 2. In summary, we do not intend to claim that our model is more efficient from a purely architectural perspective. Given that alignment-based models and single-sequence models operate on fundamentally different data types, we think that a direct comparison of architectural efficiency is not meaningful. As described above, we have refined our statement to be more precise, rather than claiming our model is "more efficient."

Finally, from a usability perspective, the need to choose among three separate models trained at different evolutionary depths (vertebrate, mammal, primate) is impractical. The authors could

consider unifying these within a single scalable framework or providing clear guidance on model selection for end users.

Response: This has been addressed in the second comment in bullet point 1.

Overall assessment

The manuscript presents a strong and technically competent piece of work that meaningfully extends the authors' previous GPN-MSA framework. The idea of leveraging phylogenetic information for genome modeling is sound and well-motivated, and the reported results are impressive across many benchmarks. However, the advance is primarily incremental, and several aspects—particularly architectural justification, fairness of comparisons, and practical usability—require further clarification before the paper reaches Nature standards.
Improving the clarity of presentation, strengthening the benchmarking fairness, and expanding the scope of evaluation (especially regarding generality and tissue specificity) would substantially enhance the paper's impact.

Figure presentation. Figures summarizing model comparisons (notably Fig. 2) should be redesigned for consistency and visual polish. Axis labels, fonts, and color schemes currently vary between panels, giving an under-finished impression.

Response: Thank you for the suggestion. We have now unified all the fonts used in Figure 2 and 3 to *Dejavu Sans*. Regarding the color scheme, we would like to clarify that we use different shadings to denote models trained at different evolutionary timescales. For example, there are three levels of shading of the blue colors used to denote GPN-Star: the darkest one denotes the vertebrate model, the lightest one denotes the primate model, and the middle one denotes the mammal model. We follow this color scheme consistently in all figures we present in this paper.

Reviewer #5

In this paper, Ye*, Benagas* et al. propose GPN-Star, a new efficient and powerful algorithm to estimate evolutionary constraint. The method leverages Genomic language models and explicitly accounts for confounders such as mutation rate and repeat elements. They provide 3 estimates across vertebrates, mammals and primates. These scores are evaluated through rigorous analyses using an impressive number of disease datasets, spanning rare or common variants, and coding and non-coding variants. GPN-Star is shown to significantly outperform existing evolutionary constraint scores (i.e., phastcons and phylop) as well as other methods predicting deleterious effects.

I am overall very impressed by the results of GPN-Star, although I do not have the expertise to fully understand the methodology. PhyloP and PhastCons scores have been widely used in genetics but have been developed more than a decade ago (2005 and 2010). I anticipate GPN-Star scores to become the gold standard for constraint scores and to be widely used to prioritize disease variants.
I have nevertheless some concerns on the manuscript.

Response: Thank you for your encouraging assessment of our work and for your constructive feedback. Your framing – that PhyloP and PhastCons have served the community well for over two decades, but that the field is ready for a more powerful successor – captures precisely the motivation behind GPN-Star. We are gratified that the results are compelling even to a reader who approaches this work primarily from the genetics rather than the methods side, as this is exactly the audience we most hope to serve. We have carefully incorporated all your suggestions, adding several new analyses and revising the manuscript accordingly. Please find our detailed point-by-point responses to your specific comments below.

My main critic is the limited biological and evolutionary insights presented in this paper. The main conclusion outcome from this paper is that the timescale of the constraint score matters when prioritizing variants. However, this observation was already described by the Zoonomia consortium in Sullivan et al. 2023 Science, who shown that primate constraint scores were more informative than mammal constraint scores in heritability analyses of non-coding variants in GWAS, while mammal constraint scores were more informative than primate constraint scores in analyses of coding and/or low-frequency variants.

Response: Thank you for raising this concern. While we did not intentionally downplay the significance of the Sullivan et al. 2023 study (we have cited it multiple times in the paper, as it is inspirational to our work), we acknowledge that it is not emphasized sufficiently. We are aware that this may not be new for evolutionary biologists, and our intention is to bring this to the attention of the machine learning community, where this issue is often overlooked. We have added a sentence in the corresponding paragraph in the Results to clearly acknowledge the previous observations:

"*Similar timescale-dependent performance patterns have been reported in a previous study \cite{sullivan2023leveraging} with PhyloP and PhastCons. We confirm that this dependency is not unique to classical phylogenetic models but also governs the performance of gLMs across a broader range of predictive tasks.*"

Also we have rephrased the relevant sentence in the Discussion:

"*A central insight from our results is the strong dependence of gLM-learned constraint on the evolutionary timescale represented in the training data, thereby corroborating and extending the observations reported by Sullivan et al.\cite{sullivan2023leveraging}.*"

Clear examples of disease variants and disease genes prioritized by GPN-Star but not by other constraint scores would be a plus.

Response: Thank you for your suggestion. We have carefully investigated those cases in our benchmarks where GPN-Star correctly classifies the variant while phyloP and phastCons fail, and reported three representative cases (Extended Data Figures. 7, 8, 9) with existing experimental evidence about the regulatory mechanisms. Primarily, GPN-Star's advantage comes from the ability to effectively use the sequence context. These variants do not show strong site-wise conservation, but reside in functionally important elements with distinct sequence context. GPN-Star effectively leverages this information to make more accurate predictions, whereas the traditional conservation scores are unable to do so. Additionally, the fact that GPN-Star outperforms phyloP and phastCons at every variant effect prediction benchmarks have already demonstrated the presence of systematic improvements.
As a representative example, we highlight a case from OMIM (chrX:48824894 A>T, causing X-linked chondrodysplasia). This is a 3' UTR variant of the gene *HDAC6* that disrupts a miRNA binding site (miR-433), experimentally validated to increase mRNA stability and its abundance (Simon et al., 2010, *Human Molecular Genetics*). The genomic locus does not show strong conservation, and consequently phyloP and phastCons produce near-neutral scores; GPN-Star, in constrast, correctly identifies it as deleterious. Nucleotide dependency analysis shows that GPN-Star learns the miRNA binding site as a functional element, as well as the strong dependency between it and the polyadenylation signal, consistent with the known regulatory mechanism.

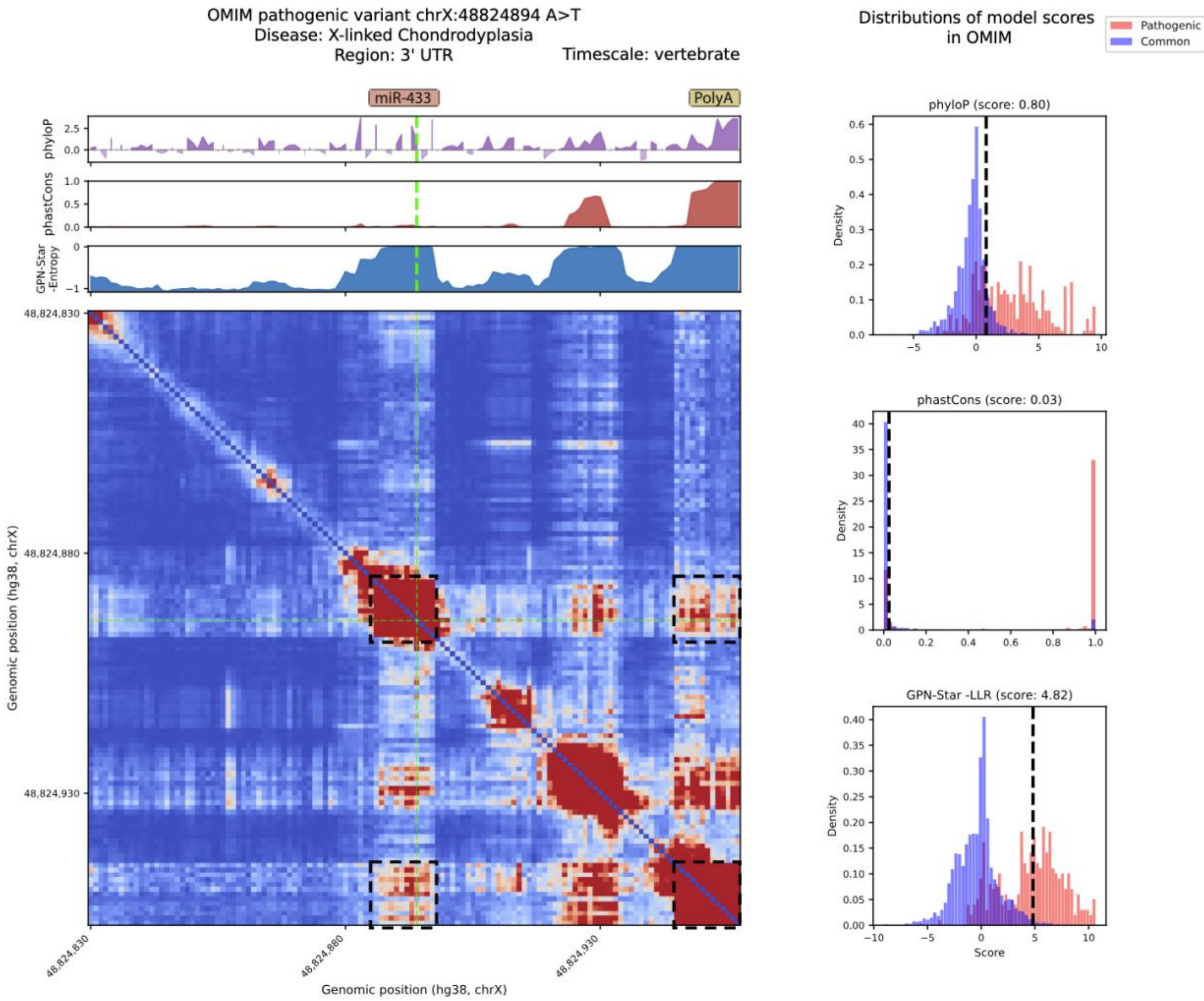

Extended Data Figure 7: Case study on a pathogenic variant in 3' UTR at the vertebrate evolutionary timescale. This variant is correctly classified as deleterious by GPN-Star while PhyloP and PhastCons produce neutral scores (right column). The left column shows the nucleotide dependency map, as well as PhyloP, PhastCons, and GPN-Star prediction tracks in the genomic window chrX:48824830-48824958 centered around the variant (green line), overlaid with functional elements identified in an experimental study. The variant is located in a miR-433 binding site and disrupts the seed sequence, thereby increasing the mRNA stability. Nucleotide dependency map by GPN-Star identifies the miRNA binding site as a functionally important element, as well as its interdependency with the polyadenylation signal.

We also present a 5' UTR variant that disrupts the Kozak sequence, and a promoter variant that disrupts the initiator element, both experimentally validated in previous studies.

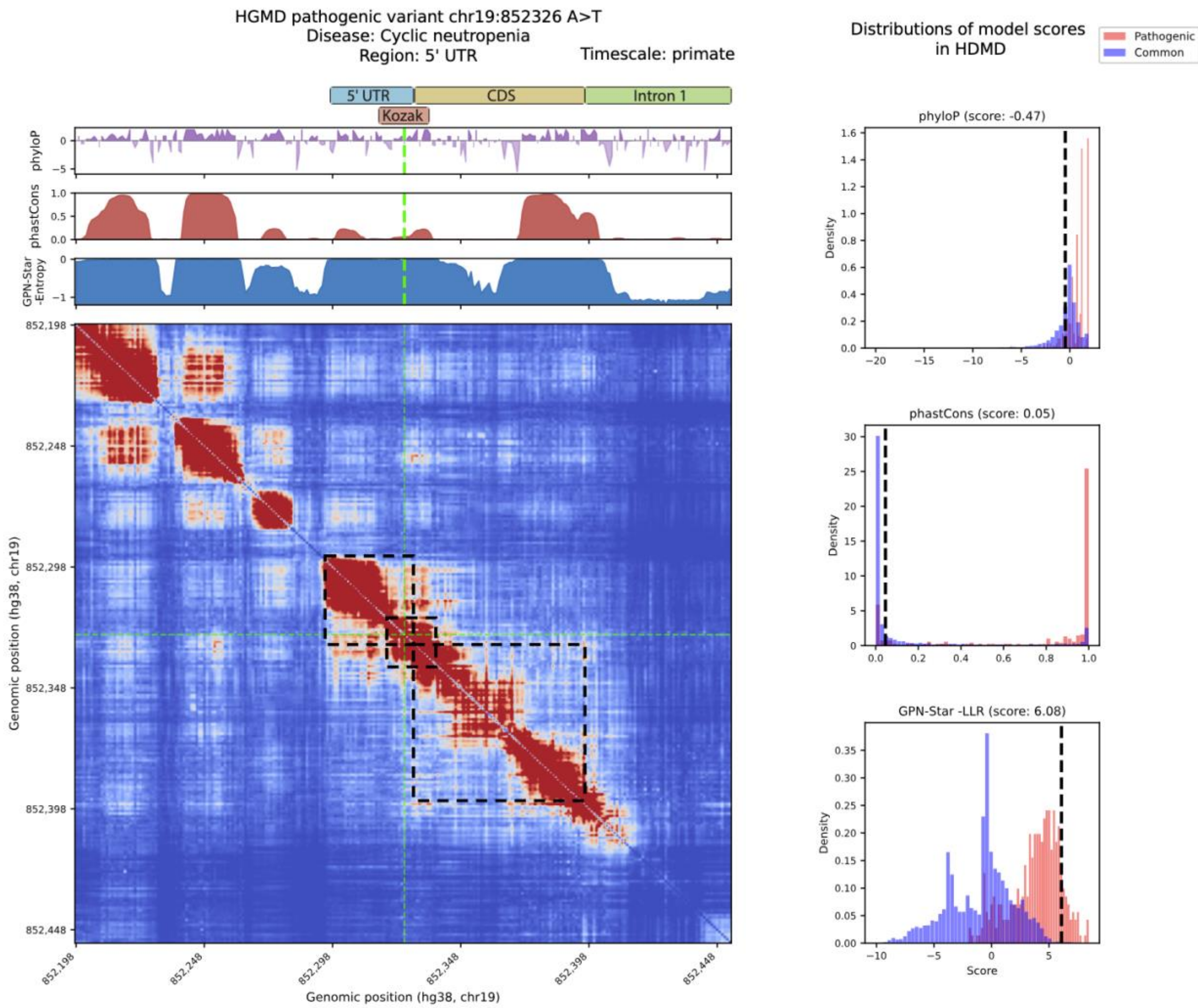

Extended Data Figure 8: Case study on a pathogenic variant in 5' UTR at the primate evolutionary timescale. This variant is correctly classified as deleterious by GPN-Star while PhyloP and PhastCons produce neutral scores (right column). The left column shows the nucleotide dependency map, as well as PhyloP, PhastCons, and GPN-Star prediction tracks in the genomic window chr19:852198-852454 centered around the variant (green line), overlaid with functional elements identified in an experimental study. The variant is located in the Kozak sequence and disrupts the critical purine at position −3. Nucleotide dependency map by GPN-Star identifies the Kozak sequence as a functionally important element, as well as its dependencies with the surrounding 5' UTR and the initial CDS region.

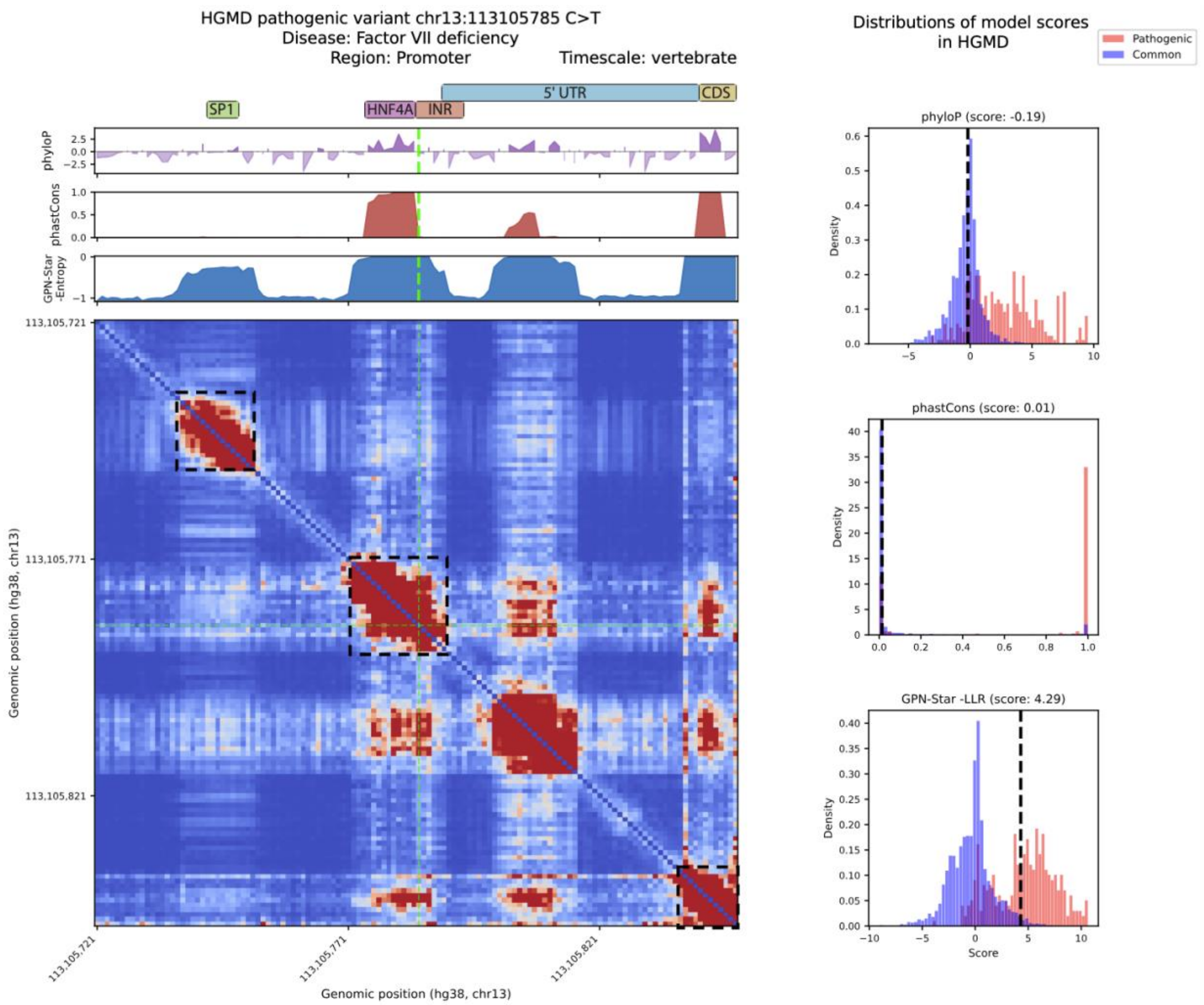

Extended Data Figure 9: Case study on a pathogenic variant in promoter region at the vertebrate evolutionary timescale. This variant is correctly classified as deleterious by GPN-Star while PhyloP and PhastCons produce neutral scores.(right column). The left column shows the nucleotide dependency map, as well as PhyloP, PhastCons, and GPN-Star prediction tracks in the genomic window chr13:113105721-113105977 centered around the variant (green line), overlaid with functional elements identified in an experimental study. The variant is located at the boundary of an HNF4A binding site and the initiator element. Nucleotide dependency map by GPN-Star identifies the HNF4A binding site and the initiator element as functionally important elements, as well as their dependencies with a 5' UTR element and the initial CDS region.

Similarly, leveraging evolutionary constraint to prioritize genetic variants is not new. I would cite recent works from the Zoonomia consortium and from Illumina (e.g. Kuderna et al. 2024 Nature) in the first paragraph of the Introduction.

Response: We have added a sentence and the citations in the first paragraph of the Introduction as suggested.

"*More recently, large-scale comparative genomics studies have systematically quantified evolutionary constraints across the genomes of hundreds of species \cite{sullivan2023leveraging,kuderna2024identification}*."

The GitHub page associated to GPN-Star (https://github.com/songlab-cal/gpn) is not well documented. In addition, prediction scores are not available yet. For a method paper focusing a generating a new tool and new scores for the community, this lack of usability and accessibility is a weakness.

Response: We have improved the documentation of our GitHub repository. We recognize that the precomputed prediction scores for genome-wide variants would be a valuable resource to the community. We have been actively working on computing these scores, and, as stated in the Data Availability section, we commit to making them publicly available upon publication. We have also established collaborations with the UCSC Genome Browser and IGVF Consortium to host our model scores on their platforms, which we believe will further improve the accessibility of GPN-Star.

I have also some technical questions and presentation suggestions:
- What is the recommended GPN-Star to prioritize variants in GWAS? GPN-Star (M) outperforms GPN-Star (P) in GWAS fine-mapped variants, but GPN-Star (P) outperforms GPN-Star (M) in heritability analyses.

Response: Thank you for noting this discrepancy. We have also been curious and working to understand it. Fine-mapping is known to preferentially identify causal variants with larger effect sizes over the smaller ones, given the limited statistical power. By contrast, the heritability enrichment analysis takes into account variants across the full range of effect sizes. Consequently, fine-mapped variants are more enriched for large-effect size variants relative to those covered in the S-LDSC analyses. Consistent with our discussion of evolutionary timescales, the mammal model trained on a longer timescale has an advantage in this setting. However, empirically verifying this hypothesis is difficult, as the precise causal variants with smaller effect sizes included in S-LDSC cannot be directly identified. As an approximation, we have compared the effect size distributions between putatively causal variants from fine-mapping and the top 0.1% variants identified by GPN-Star in S-LDSC. We found that the overall effect sizes of fine-mapped variants are substantially larger than those used in the S-LDSC set.

Here we show the distribution of effect sizes among different sets of variants, summarized across different traits in the S-LDSC GWAS datasets by taking the mean (left panel) or max (right panel) Z^2 across traits. "All" (the purple curve) denotes all regression variants in S-LDSC. "GPN-Star-V top 0.1%" (the green curve) denotes regression variants in the top 0.1% GPN-Star (V) annotation; the blue and orange curves follow a similar convention. The red curve denotes all variants overlapped with the fine-mapped high PIP (>0.9) variant set. We think this results serve as indirect evidence supporting our hypothesis. However, because this relies on an approximation rather than the exact set of causal variants, we have opted to provide this analysis here for your reference rather than adding it to the main manuscript.

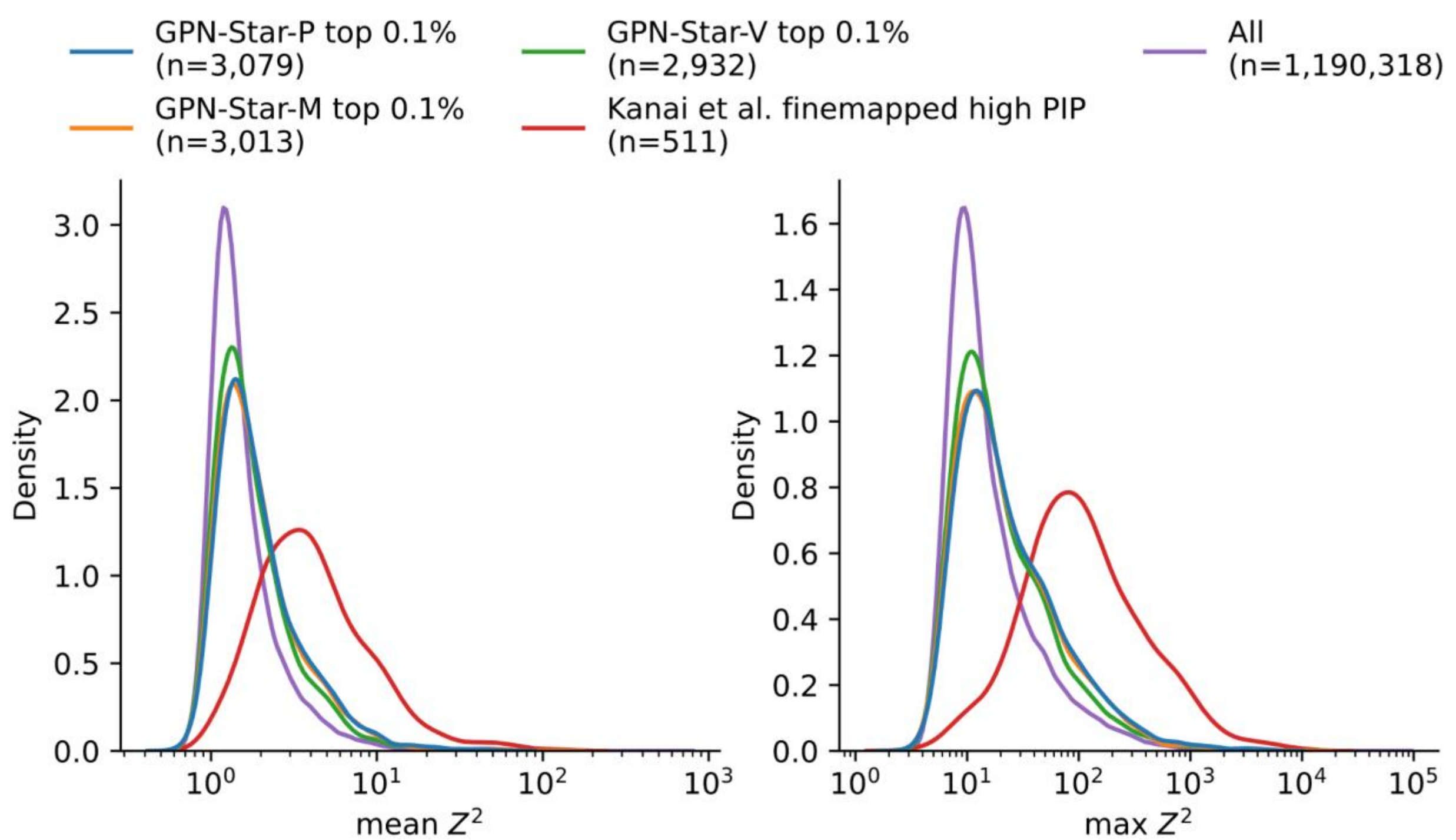

Additionally, the fine-mapped variant set is relatively small and may not be representative of the broader genome-wide pattern; we therefore consider the S-LDSC results more informative for assessing model performance.

For model selection, we always recommend a data-driven approach to transform, select, or weight the model scores. For example, statistical genetics methods typically allow using multiple variant scores, whose importances will be determined by the statistical framework. We have added a paragraph in the Discussion on anticipated model usage.

"*We anticipate GPN-Star predictions to be used in ways analogous to classical conservation scores. The model scores carry a specific interpretation: they reflect evolutionary constraint at particular timescales rather than serving as general-purpose variant effect predictions. When interpreting individual variants, we recommend choosing an appropriate timescale based on the patterns described above. For integrating or selecting among models within predictive or discovery workflows, we recommend data-driven approaches that learn appropriate weights and transformations from task-specific data, as we demonstrated with DeepRVAT in our rare variant association testing analysis.*"

If one just wants to focus on one timescale, we would recommend following the patterns observed in the heritability analysis results for analyzing GWAS data, as they covered a wider range of variants. It is recommended to choose the mammal model for less polygenic traits and the primate model for more polygenic traits, following our analysis shown in Figure 3E.

- Fig 2, 3 & 6: The authors should consider using 95% confidence intervals instead of standard to better represent the uncertainty in the estimates and to avoid potentially overselling the significance of the observed differences.

Response: Thank you for the suggestion. We now plot error bars as 95% confidence intervals for all estimates obtained via bootstrapping in Figures. 2, 6 and all the related Supplementary Figs., as we agree it is the more rigorous approach. We decided to retain standard errors in the S-LDSC heritability analysis plots to remain consistent with the established norm in the field since the original S-LDSC paper (Finucane et al., 2018, *Nat Genet*).

- From my personal experience, a large proportion of the genome (at least 1%) saturate at a phastcons score of 1 for mammals and primates. I am thus wondering how the authors selected the top 0.1% of phastcons variants?

Response: We did find variants with tied phastCons scores at the top percentiles, which we resolved by random sampling among tied variatns.We note the following:

1. The inability of phastCons to resolve rankings among its top-scoring variants is a known limitation. GPN-Star demonstrates that finer-grained variant ranking at this scale yields meaningful performance benefits.
2. The proportion of variants with phastCons scores of 1.0 is very low in our analysis (e.g., 0.18% for the primate score), likely because the S-LDSC analysis predominantly includes common variants, which tend to reside in less constrained loci. PhastCons score saturation is therefore not a material issue in our results.
3. As shown in Figure 3D, GPN-Star still clearly outperforms phastCons at higher percentiles.

This is an important point that worth noting. We have added a sentence in the Methods section to clarify this.

"*Sometimes, the number of variants with tied PhastCons scores saturated at 1.0 exceeds the number of variants selected at the 0.1% quantile. In those cases, we resolved rankings via random sampling.*"

- Fig 2G: The authors should clarify how the controls have been selected.

Response: We have added more details for processing each benchmark dataset. For the GWAS fine-mapping dataset, we have added the description in Methods:

"*GWAS fine-mapping [48, 104]: for non-coding variants, we downloaded processed complex traits dataset from TraitGym [9]. Molecular consequences of variants were obtained with Ensembl VEP annotator [102] with the --most_severe flag. Each positive variant was matched with nine control variants matched by chromosome, consequence, distance to transcription start site (TSS), LD score and MAF. We processed coding variants from the same source [48,*

104], but only matched MAF, similar to GeneticsGym [105]. The goal is to classify variants with high vs. low posterior inclusion probability (PIP) and the metric is the AUPRC."

- Fig 2J: Could the authors label V, M and P on the Figure?

Response: We apologize for the confusion. Every three points in the same color on this plot represent three replicates of DeepRVAT training with different random initializations and otherwise identical settings. Each DeepRVAT+GPN-Star replicate uses predictions from all three GPN-Star models, and DeepRVAT is trained to learn appropriate featureweights during training. To avoid potential misunderstanding, we have added a clarifying sentence to the figure caption.

"Every three points with the same color represent three models with the same inputs trained with different random initializations."

- The link section for phastcons (P) is https://cgl.gi.ucsc.edu/data/cactus/zoonomia-2021-track-hub/hg38/phyloPPrimates.bigWig. - is it a typo, or do Zoonomia PhastCons scores have been released in a file called phyloP?

Response: Yes, this is a mislabeled file. You may refer to the documentation of this track: https://www.google.com/url?q=https://cgl.gi.ucsc.edu/data/cactus/zoonomia-2021-track-hub/hg38/phyloPPrimates.html&sa=D&source=docs&ust=1769808923228384&usg=AOvVaw0yDMLuAJuCJahW25-QVQ1K.

Here, the track is described as provding phastCons scores, while the filename reads phyloP. The score range and distribution are also consistent with phastCons rather than phyloP.

Comments on code: The GitHub page associated to GPN-Star (https://github.com/songlab-cal/gpn) is not well documented. In addition, prediction scores are not available yet. For a method paper focusing a generating a new tool and new scores for the community, this lack of usability and accessibility is a weakness.

Response: This has been addressed in an earlier comment.

References:

1. Avsec, Žiga, et al. "Advancing regulatory variant effect prediction with AlphaGenome." *Nature* 649.8099 (2026): 1206-1218.
2. Benegas, Gonzalo, et al. "A DNA language model based on multispecies alignment predicts the effects of genome-wide variants." *Nature Biotechnology* 43.12 (2025): 1960-1965.
3. Benegas, Gonzalo, Gökcen Eraslan, and Yun S. Song. "Benchmarking dna sequence models for causal regulatory variant prediction in human genetics." *bioRxiv* (2025).
4. Brixi, Garyk, et al. "Genome modelling and design across all domains of life with Evo 2." *Nature* (2026): 1-13.
5. Davison, Anthony Christopher, and David Victor Hinkley. Bootstrap methods and their application. No. 1. *Cambridge university press*, 1997.
6. Fabiha, Tabassum, et al. "A consensus variant-to-function score to functionally prioritize variants for disease." *bioRxiv* (2024): 2024-11.
7. Finucane, Hilary K., et al. "Heritability enrichment of specifically expressed genes identifies disease-relevant tissues and cell types." *Nature genetics* 50.4 (2018): 621-629.
8. Lin, Zeming, et al. "Evolutionary-scale prediction of atomic-level protein structure with a language model." *Science* 379.6637 (2023): 1123-1130.
9. Linder, Johannes, et al. "Predicting RNA-seq coverage from DNA sequence as a unifying model of gene regulation." *Nature Genetics* 57.4 (2025): 949-961.
10. Simon, Delphine, et al. "A mutation in the 3′-UTR of the HDAC6 gene abolishing the post-transcriptional regulation mediated by hsa-miR-433 is linked to a new form of dominant X-linked chondrodysplasia." *Human molecular genetics* 19.10 (2010): 2015-2027.
11. Srivastava, Divyanshi, et al. "Borzoi-informed fine mapping improves causal variant prioritization in complex trait GWAS." *bioRxiv* (2025): 2025-07.
12. Sullivan, Patrick F., et al. "Leveraging base-pair mammalian constraint to understand genetic variation and human disease." *Science* 380.6643 (2023): eabn2937.

Point-by-Point Response to Reviews

Manuscript ID: 2025-09-23375B
Title: Predicting functional constraints across evolutionary timescales with phylogeny-informed genomic language models

We thank the editor and all reviewers again for their insightful and constructive feedback. Please find below our point-by-point responses to the remaining comments.

Referees' comments:

Referee #1 (Remarks to the Author):

We believe the paper is improved and now provides a more complete picture of the conceptual and applicable advances of the modelling approach. We consider the conceptual and technical contributions presented in this manuscript to be timely, innovative, and important. The broader discovery impact remains to be seen; however, we believe this will be a model broadly embraced by the community, and we therefore endorse the publication of the current version.

Referee #1 (Remarks on code availability):

Codebase appropriately annotated and reusable by the community.

Thank you for your endorsement and insightful comments, which helped to improve our manuscript substantially.

Referee #2 (Remarks to the Author):

I co-reviewed this manuscript with one of the reviewers who provided the listed reports.

Referee #3 (Remarks to the Author):

The authors have performed a comprehensive review of the manuscript and addressed most points satisfactorily. The points below require final consideration:

Thank you for your constructive comments. Please find below our response to your remaining points.

2 — Benchmarking and comparison to existing models

This point was not addressed: ”Second, the baseline comparisons are not entirely fair or up to date. GPN-Star leverages WGAs, whereas most competing models (e.g., Nucleotide

Transformer, Evo, Enformer) operate on single sequences. This gives GPN-Star access to substantially more information, which should be clearly acknowledged."

Thank you for pointing this out. We have made several edits to our manuscript to clarify this distinction.

In the Discussion, we have rephrased the following sentence that discusses single-sequence vs. alignment-based models.

"*GPN-Star achieves state-of-the-art performance in functional constraint prediction with substantially smaller model and context sizes compared to existing single-sequence gLMs. This effectiveness likely stems from the explicit homology information provided by the alignment, which is not readily available to single-sequence models.*"

In the Results section, where we first introduce the models, we now further emphasize the alignment-based vs. single-sequence nature of the compared models.

"We compared our models with major genome-wide variant effect predictors, including classical evolutionary methods (PhyloP, PhastCons) that also use whole-genome alignment, the ensemble model CADD, recent single-sequence gLMs (Nucleotide Transformer 2.5B multispecies and Evo 2 40B models), and GPN-MSA."

"Notably, the sequence-to-function models performed substantially worse than alignment-based gLMs and phylogenetic models on this task, consistent with recent findings."

4 — Beyond variant-effect prediction

The authors performed classification analysis of different genomic regions with GPN-Star embeddings to quantitatively evaluate the capacity of our model to learn functional elements. However, there is no comparison to other genomics models. This should be done for completeness and a full picture on the quality of GPN-Star embeddings.

In the first revision, we did not include a comparison with other models for the following reasons:

1. The embedding visualization in Figure 4A was included as a qualitative illustration of what GPN-Star has learned internally, not as a benchmark of embedding quality or a claim of superiority over other models (nowhere in our manuscript do we make such a claim). The core contribution of GPN-Star is variant effect prediction, and we have comprehensively evaluated the model on this primary task against a comprehensive set of competing methods throughout the paper.
2. We agree that benchmarking GPN-Star's embeddings against those of other genomic models in a scholarly manner is a scientifically relevant task, but it would require a separate, dedicated transfer-learning study. The utility of learned embeddings is highly task-dependent; for example, embeddings that perform well at distinguishing genomic

region types may perform poorly at predicting splicing or transcription factor binding. A rigorous comparison would require careful selection of diverse downstream tasks, appropriate probing or fine-tuning protocols, and accommodating different embedding and context sizes, representing a substantial body of work and computational cost that is beyond the scope of this study.

3. We also note that while cross-species genome annotation is a scientifically important application for gLMs, within-human genomic region classification is somewhat limited and not biologically interesting as a standalone benchmark task for these embeddings.

We are concerned that readers might overinterpret the results of a limited comparative analysis. For this reason, we prefer to avoid making quantitative statements about the relative performance of embeddings derived from different models.

While we maintain that a comprehensive comparison falls outside the primary scope of our work, we nevertheless want to be responsive to your feedback. To that end, we have performed a preliminary analysis of genomic region classification with the Evo 2 7B and NT v1 2.5B MS models using the same dataset. The results are shown in the figure below.

Specifically, when evaluated using the same 256bp context size as GPN-Star (M) (NT used 258bp due to the 6-mer tokenization), both Evo 2 and NT performed worse than GPN-Star across all categories. When evaluated using their native context sizes (8192 bp for Evo 2 and ~6kb for NT), however, Evo 2 moderately outperforms GPN-Star in most classes, and NT performs on par with GPN-Star. Echoing our discussion in the previous round of revision, these results suggest that context length plays a major role in this task. More broadly, they illustrate why a rigorous comparison between models requires evaluation across multiple tasks and settings to ensure a fair and meaningful assessment.

For these reasons, we chose not to include this analysis in the manuscript. However, because our response to reviews will be publicly available, we present these results here in the interest of transparency and to make the analysis available to the community.

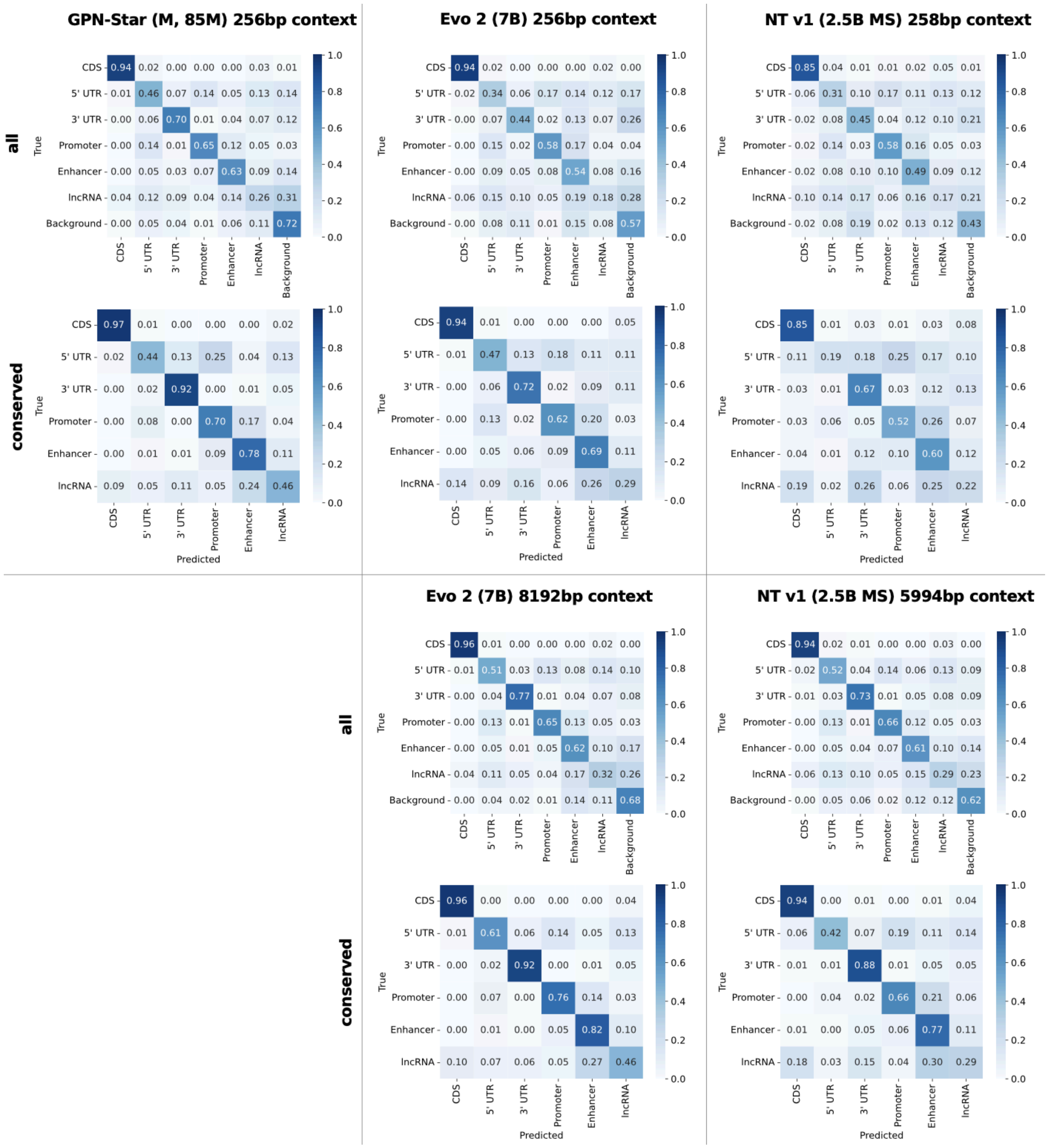

6 — Practicality and computational considerations

The authors mention that in their experiments they observed a drop in model performance if the alignment data is not available during inference. This result should be briefly mentioned in the manuscript as it's a useful insight for the community.

We have added a sentence in the Discussion section to acknowledge this point.

"*GPN-Star requires the availability of the full alignment data when making predictions. Omitting alignment data at inference time can lead to a significant degradation in performance.*"

Other comments

As discussed by different reviewers, it is very important that the authors provide genome-wide predictions for all the models and the precomputed variant scores upon publication to facilitate the use of the model by the community.

We fully recognize the importance of releasing genome-wide predictions and will ensure that they are available upon publication.

Referee #4 (Remarks to the Author):

I co-reviewed this manuscript with one of the reviewers who provided the listed reports.

Referee #5 (Remarks to the Author):

The authors have addressed most of my previous comments satisfactorily.

My main concern was the limited biological and evolutionary insight. While this aspect remains somewhat limited, I consider it acceptable given the primary contribution of the manuscript, namely the development of improved constraint scores. The addition of three case studies is valuable and convincing, illustrating cases where GPN-Star identifies variants as deleterious while PhyloP and PhastCons assign near-neutral scores.

I have one remaining request. The conceptual distinction between GPN-Star and classical conservation scores (PhyloP/PhastCons) is not explicit, and clarifying this point would substantially improve interpretability.
I suggest including a schematic figure illustrating contrasting scenarios, for example:
(1) bases predicted as constrained by GPN-Star but not by PhyloP/PhastCons
(2) bases predicted as constrained by PhyloP/PhastCons but not by GPN-Star
In case (1), is the signal driven by weak or noisy conservation across species, or by sequence-level constraints (e.g. motifs or context) that are not captured by site-wise conservation?
In case (2), does this reflect mutation-rate effects, alignment or phylogenetic artifacts, or regions that are evolutionarily conserved but not strongly supported by sequence context?
Making these distinctions explicit would strengthen the manuscript and help position GPN-Star relative to established conservation metrics.

Thank you for your helpful comments. As you suggested, we have added a schematic figure illustrating the two aforementioned scenarios in which GPN-Star produces more context-aware constraint predictions than classical conservation scores. In the figure, Variant 2 corresponds to scenario 1, while Variant 3 corresponds to scenario 2.

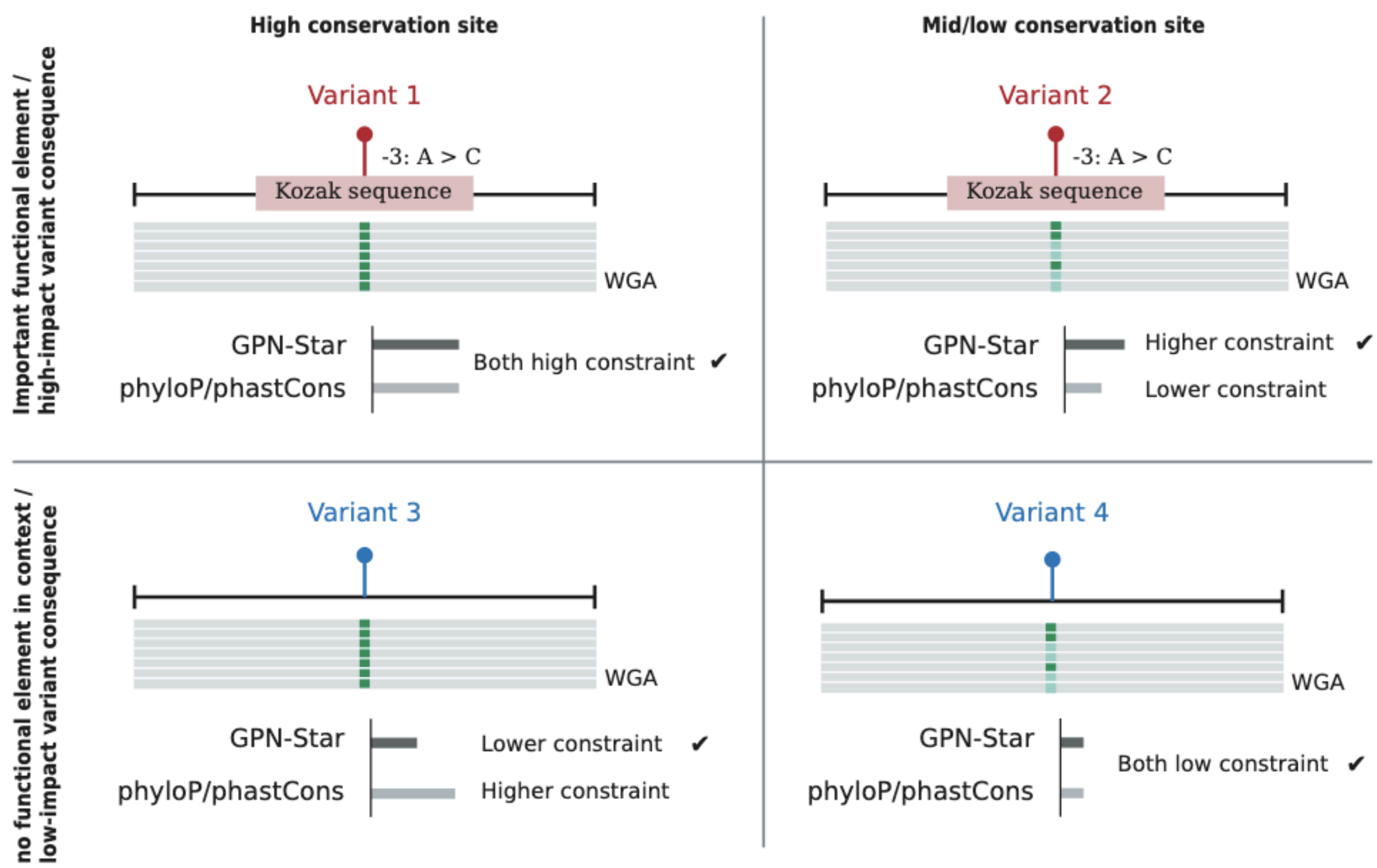

Extended Data Figure 9: Schematics illustrating representative scenarios in which GPN-Star yields more context-aware constraint predictions than traditional conservation scores. Variants 1 and 2 correspond to high-impact, pathogenic variants, whereas variants 3 and 4 correspond to putatively low-impact, benign variants. Variant 1 lies within a functionally important element and at a strongly conserved site according to the alignment; consequently, both GPN-Star and conservation-based metrics make high constraint predictions. Variant 4 is located outside functional elements and at a weakly conserved site; as such, both GPN-Star and conservation scores produce low constraint predictions. Variant 2 occurs at a weakly conserved site but falls within a functionally important element and has a highly disruptive molecular consequence. In this setting, GPN-Star makes a higher constraint prediction than traditional conservation scores. An empirical instance of this pattern is provided in Extended Data Figure 7. By contrast, Variant 3 occurs at a strongly conserved site but lacks surrounding functional elements or has low-impact molecular consequences. In such cases, GPN-Star yields lower constraint predictions than the traditional conservation scores. This behavior is illustrated by the distribution of predicted scores at perfectly conserved sites in Supplementary Figure 18.

Scenario 1 / Variant 2 is empirically illustrated by the case studies presented in Extended Data Figures 6–8, particularly Extended Data Figure 7. To provide empirical support for Scenario 2/ Variant 3, we performed an additional analysis comparing the distributions of GPN-Star, phyloP, and phastCons scores across different variant classes and genomic sites for which alignment columns exhibit perfect conservation.

As shown in the new Supplementary Figure 18 (included below), GPN-Star assigns lower constraint scores to lower-impact coding variant classes and to background (putatively non-functional) intergenic and intronic regions than to enhancer regions. In contrast, phyloP and phastCons assign uniformly high constraint scores to all perfectly conserved sites. These results suggest that GPN-Star can distinguish sites with different functional relevance even when they exhibit identical conservation patterns in the alignment.

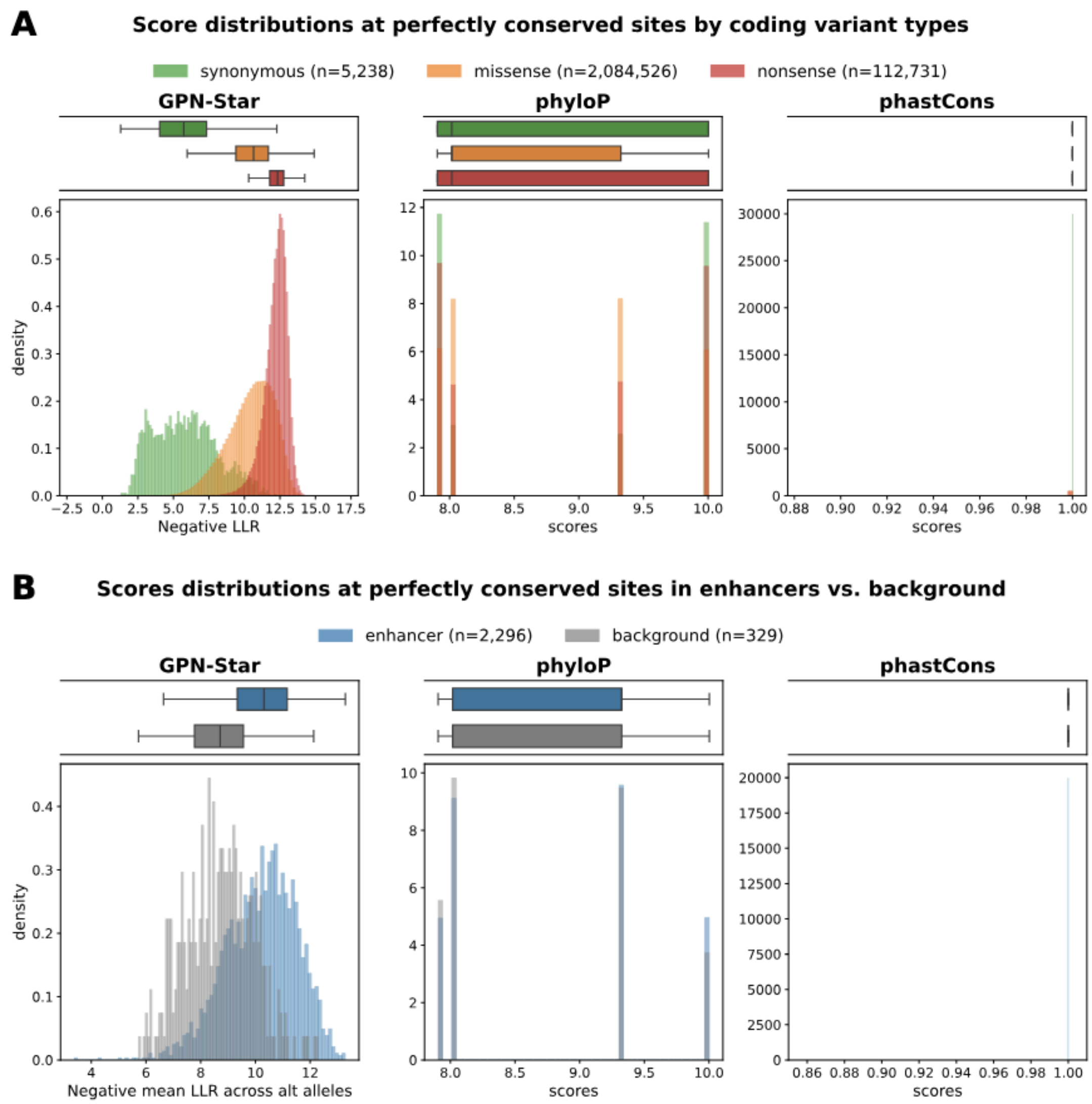

Supplementary Figure 18: Comparison of score distributions generated by GPN-Star, phyloP, and phastCons for different variant types at perfectly conserved sites in the human genome. Perfect conservation is defined as the presence of a single, gap-free nucleotide across all species in the multiz100way alignment, and all scores are computed with respect to this same alignment. (A) Nonsense, missense, and synonymous single-nucleotide variants. (B) Enhancer versus background sites. Experimentally validated enhancer annotations are obtained from the VISTA Enhancer Browser, and background sites are selected from as non-N genomic regions that exclude all exons, ENCODE V4 cCREs, and RepeatMasker repeats. The four discrete phyloP score values observed at these perfectly conserved sites arise from nucleotide-specific neutral substitution rates for the four reference bases.
